# Selective CO_2_ uptake mimics dissolution in highly fluorinated non-porous crystalline materials

**DOI:** 10.1038/s41557-025-01943-4

**Published:** 2025-10-14

**Authors:** Iñigo J. Vitórica-Yrezábal, Craig A. McAnally, Matthew P. Snelgrove, Mark R. Warren, Adrian H. Hill, Stephen P. Thompson, Martin Quinn, Sam Mottley, Stephen Mottley, Ashleigh J. Fletcher, Lee Brammer

**Affiliations:** 1 Department of Chemistry, University of Sheffield, Sheffield, UK; 2 School of Natural Science, University of Manchester, Manchester, UK; 3 Department of Chemical and Process Engineering, University of Strathclyde, Glasgow, UK; 4 Diamond Light Source, Harwell Science and Innovation Campus, Didcot, UK; 5 The European Synchrotron Radiation Facility, Grenoble, France; 6 Present Address: Rigaku Europe SE, Neu-Isenburg, Germany; 7 Present Address: Johnson Matthey, Chilton, UK

**Keywords:** Metal-organic frameworks, Supramolecular chemistry, Solid-state chemistry

## Abstract

Separation of CO_2_ from gas mixtures is important in applications such as CH_4_ gas purification and blue hydrogen production. Here we report selective CO_2_ uptake by a family of flexible silver coordination polymers (AgCPs) that are ostensibly non-porous but exhibit latent porosity to CO_2_ above a gate pressure, through a mechanism akin to dissolution in fluoroalkanes. The CO_2_ sorption properties are rationally modified by changing the perfluoroalkyl chain length of the constituent perfluorocarboxylate ligands. The AgCPs do not take up CH_4_ owing to failure of the dissolution mechanism, consistent with alkane–perfluoroalkane immiscibility. In situ single-crystal and powder X-ray diffraction enable direct visualization of the CO_2_ molecule binding domains. These techniques also reveal associated structural changes in the AgCPs and confirm the gating mechanism of CO_2_ uptake. The combination of perfluoroalkylcarboxylate ligands with the flexible silver(I) coordination sphere generates highly fluorinated but mobile regions of the crystals that play an integral role in the selective uptake of CO_2_ over CH_4_.

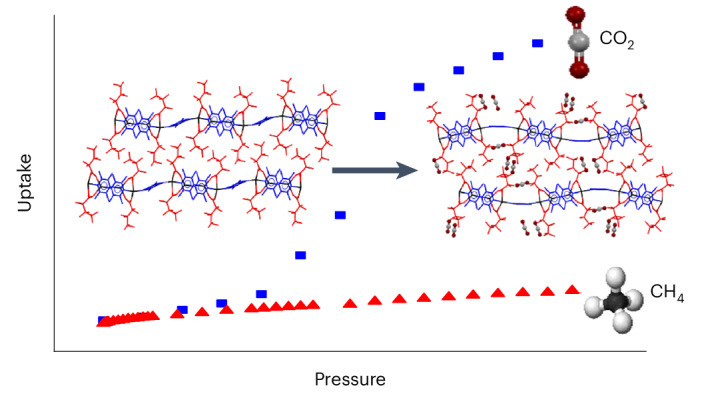

## Abstract (web summary)

Separation of CO_2_ from gas mixtures is a major application focus for porous materials. Now it has been shown that fluorinated non-porous crystalline materials can uptake CO_2_ via mobile perfluoroalkyl regions, a process resembling the dissolution of CO_2_ in perfluoroalkanes, while CH_4_ uptake is hindered. In situ X-ray diffraction data provide insight into the sorption process.

## Main

The design and synthesis of new materials for applications in molecular separations has received increasing attention in the past two decades^1–3^. In particular, materials capable of selectively separating industrially relevant mixtures have been widely studied, as most manufactured chemicals are produced as mixtures that require further purification^3^. Separation and purification often rely on energy-intensive methods such as distillation, crystallization, chemisorption (amine scrubbers for CO_2_) or evaporation, processes that account for 10–15% of the global energy consumption^1^. With predictions of a threefold increase in energy demand by 2050^2^, the development of more efficient purification materials and technologies is urgently needed. In this context, porous materials such as zeolites^4^, activated carbon^5^ and covalent organic frameworks^6^ have been extensively studied as adsorbents. Due to their modularity, however, coordination polymers (CPs) and metal–organic frameworks (MOFs) provide particularly broad scope for design and tunability as energy-efficient sorbents for molecular separations^7^. These crystalline porous materials have been classified from the first generation to the fourth generation based on the evolution of their structural features and dynamic response to stimuli^8,9^: first-generation MOFs can accommodate guest molecules, but lose their structural integrity upon guest removal; second-generation MOFs retain their structures upon guest insertion and removal, exhibiting permanent porosity; third-generation MOFs, also known as flexible or soft MOFs, show reversible structural transformations upon exposure to external stimuli; and fourth-generation materials are able to modify their pore size and chemistry, having self-switching pores under the influence of an external stimulus. Whereas first- and second-generation MOFs are well established, third- and fourth-generation flexible MOFs remain far less common due to design and synthesis challenges and the identification of dynamic behaviour in such materials. Second-generation MOFs typically exhibit type I adsorption isotherms, where the gas sorption and desorption processes often take place at low gas pressures and may lie outside the operationally relevant range for industrial plants, thus decreasing their working capacity^10,11^. Third- and fourth-generation MOFs have desirable F-type sorption profiles as a consequence of their flexibility^12,13^, although crystallographic documentation of guest transport in these flexible materials is rare^10,14,15^ as single crystals often deteriorate due to strain caused by the accompanying structural transformations. The typical stepped sorption profile exhibited by fourth-generation materials arises from the structural conversion between closed-pore and open-pore forms. A distinctive pressure threshold for switching (gate pressure) characterizes the sorption profile, below which the material exhibits minimal gas sorption. Such flexible materials represent a potential opportunity for gas storage by offering higher working capacities for the more volatile gases (O_2_, H_2_, CH_4_ or C_2_H_2_) for which high-pressure storage is not feasible^10^. The gas separation properties of fourth-generation CPs and MOFs remain underexplored due to the novelty and rarity of these materials^16^. Nevertheless, they are attracting attention as their stepped adsorption profiles can provide high adsorption selectivity^10,13^. This is especially relevant when only a single gas in a mixture induces the structural transformation that enables sorption.

Previously, we have reported the synthesis and dynamic behaviour of crystalline one-dimensional silver CPs (AgCPs), [Ag_4_(O_2_C(CF_2_)_*m*_CF_3_)_4_(TMP)_3_] (*m* = 2 (**1**) and 3 (**2**), TMP = 2,3,5,6-tetramethylpyrazine), the crystal structures of which lack defined voids or channels normally associated with porosity^17–19^. Here, we describe the CO_2_ and CH_4_ gas adsorption properties and associated structural transformations of the homologous series of five non-porous isoreticular AgCPs [Ag_4_(O_2_C(CF_2_)_*m*_CF_3_)_4_(TMP)_3_] (*m* = 2 (**1**), 3 (**2**), 4 (**3**), 5 (**4**) and 6 (**5**)). We show that these materials enable the adsorption, transport and encapsulation of CO_2_ molecules in a process akin to dissolution in liquids, but occurring only above gate pressures that are dependent upon the length of the perfluoroalkyl groups of the carboxylate ligands. By contrast, CH_4_ adsorption is prevented, enabling excellent CO_2_/CH_4_ separation characteristics.

## Results and discussion

### Crystal structures of AgCPs 1–5

**Fig. 1:**
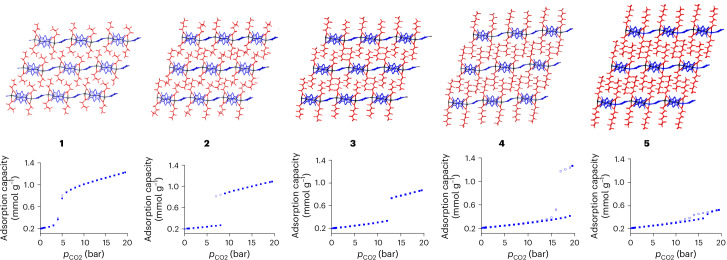
Crystal structures and CO_2_ sorption isotherms for compounds **1**–**5**. Top: crystal structures of AgCPs **1**–**5**, showing polymeric zig-zag tapes extending horizontally via alternating single and double TMP bridges between pairs of Ag(I) centres. Interdigitation of perfluoroalkyl groups creates fluoroalkyl regions between the CPs. Perfluorocarboxylates are shown in red, TMP ligands in blue and silver atoms in black. Bottom: CO_2_ adsorption isotherms at 273 K for AgCPs **1**–**5**; solid squares represent adsorption, and open squares represent desorption.

The crystal structures of AgCPs **1**–**5** comprise pairs of silver(I) ions bridged by pairs of perfluorocarboxylate ligands forming di-silver units Ag_2_(O_2_C(CF_2_)_*m*_CF_3_)_2_, which are linked by pairs of TMP ligands resulting in Ag_4_(O_2_C(CF_2_)_*m*_CF_3_)_4_(TMP)_2_ units (*m* = 2–6). These tetramer units are further linked by single-ligand TMP bridges to form a polymeric zig-zag tape (**1**–**5**). The individual polymers assemble in a rod-like distorted hexagonal packing motif wherein the fluoroalkyl groups project orthogonal to the AgCP tape direction and form fluoroalkyl layers through interdigitation with neighbouring polymers (Fig. 1 and Supplementary Figs. 11–21). The fluoroalkyl groups exhibit some disorder in the crystal structures, suggesting inefficiency in packing and a degree of mobility as a consequence of the weak dispersion interactions between them, resembling fluoroalkane liquids and amorphous polymers^20,21^.

### CO_2_ and CH_4_ sorption by AgCPs 1–5

**Fig. 2:**
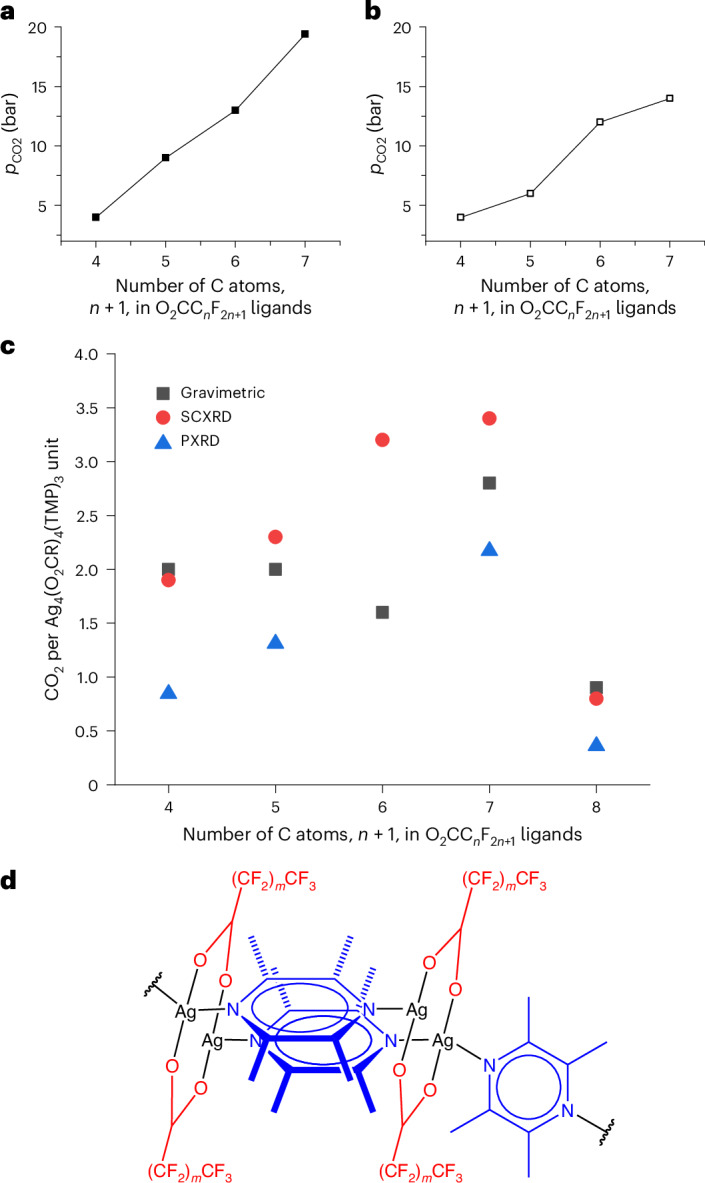
CO_2_ uptake behaviour as a function of perfluoroalkyl chain length. **a**,**b**, The CO_2_ pressure threshold at which adsorption (**a**) and desorption (**b**) is observed at 273 K, plotted versus the number of carbons in the perfluorocarboxylate ligands for AgCPs **1**–**4**. **c**, The number of CO_2_ molecules per AgCP FU estimated from gravimetric sorption (black squares, 273 K), from refined CO_2_ molecule occupancy from SCXRD (red circles, 200–232 K) and from volume increase at initial gate opening from PXRD (blue triangles, 298 K). Data are plotted versus the number of carbons of the perfluorocarboxylate ligands of **1**–**5**. **d**, FU, [Ag_4_(CO_2_(CF_2_)_*m*_CF_3_)_4_(TMP)_3_] (*m* = 2 (**1**), 3 (**2**), 4 (**3**), 5 (**4**) and 6 (**5**)). Atom colours are as in Fig. 1.

**Extended Data Fig. 1:**
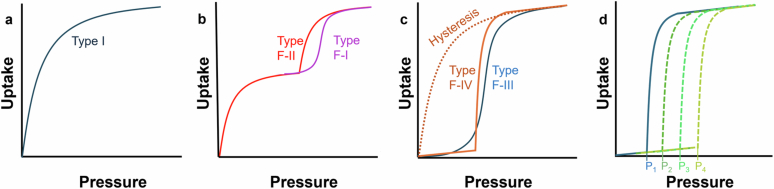
Schematic representation of the gas uptake in rigid and flexible porous materials. **a**, Type I isotherm is typical of a rigid 2nd generation MOF; **b**, Type F-I (gradual in purple) and F-II (abrupt in red) isotherms are typical of a flexible 3rd generation MOF involved in a transformation from open to more open pores; **c**, Type F-III (gradual, blue) and F-IV (abrupt, orange) isotherms and hysteresis in the desorption process (orange dotted) are typical of a flexible 4th generation MOF; and **d**, Type F-IV isotherms are typical of a 4^th^ generation MOFs in which rational modification of the structure induces a gradual change in the gate-pressure threshold. (Adapted from ref. ^13^).

CO_2_ adsorption isotherms were measured for AgCPs 1–5 at 273 K (Fig. 1). These type F-III (for **1** and **5**) and F-IV (for **2**–**4**) adsorption isotherms (Extended Data Fig. 1) illustrate that the gas uptake is gated, and diffraction studies show the gate is accompanied by a structural change from non-porous to guest-containing structure (vide infra; Supplementary Section 4). There is a monotonic trend of increasing gate pressure with increasing perfluoroalkyl chain length (Fig. 2a,b) resulting in gate-opening pressures of approximately 3 bar for **1**, 8 bar for **2**, 12 bar for **3** and 19 bar for **4**. CP **5** does not appear to continue this trend within our experimentally accessible pressure range (*p* ≤ 19.5 bar; *p*/*p*_0_ ≤ 0.54 at 273 K) for the isotherm measurements and exhibits a gate at 16 bar, although involving a much smaller adsorption step than those for **1**–**4**. This smaller lower-pressure step for **5** could also represent a small initial gate opening that precedes a larger, higher-pressure step (that is, *p* > 19.5 bar) more comparable to the gating processes observed for **1**–**4**, a supposition supported by in situ powder X-ray diffraction (PXRD) studies (vide infra).

Maximum CO_2_ adsorption capacities (19.5 bar, 273 K; Fig. 2c) revealed the uptake of 2.0 CO_2_ molecules per formula unit (FU) for **1** (1.19 mmol g^−1^) and **2** (1.03 mmol g^−1^), 1.6 CO_2_ per FU for **3** (0.78 mmol g^−1^), 2.8 CO_2_ per FU for **4** (1.24 mmol g^−1^) and only 0.9 CO_2_ per FU for **5** (0.37 mmol g^−1^). A comprehensive interpretation of the adsorption trends will be developed through consideration of these results alongside those of crystallographic studies (Fig. 2c, vide infra). The CO_2_ isotherms for **1**–**3** and **5** were also measured at 253 K and for **4** at 258 and 263 K and show modest increases in adsorption capacity relative to 273 K (Supplementary Figs. 118–133). Interestingly, the isotherms for **4** at 258 K and 263 K reveal that the step in the isotherm now splits into two steps, with temperature-dependent onset pressures (Supplementary Fig. 130), suggesting, as implied by the study of **5**, that adsorption of CO_2_ in these perfluoroalkylated materials can be a multistep process, dependent upon perfluoroalkyl chain length and adsorption conditions (temperature, *T*, and pressure, *p*).

The CH_4_ adsorption isotherms measured for AgCPs **1** and **2** at 273 K (Supplementary Figs. 127 and 128) are a marked contrast to the CO_2_ isotherms. These type III isotherms, characteristic of nonporous materials, confirm negligible CH_4_ adsorption by **1** and **2** in the pressure range evaluated. The non-porous nature of these materials is also confirmed by N_2_ adsorption isotherms (Supplementary Fig. 117), which show negligible gas uptake.

### CO_2_ and CH_4_ gas sorption studied by X-ray diffraction

Initial synchrotron PXRD measurements under vacuum identified previously established forms^17–19^ of **1** (polymorph **1**_**B**_^**HT**^), **2** (polymorph **2**^**LT**^), **4** and **5** by Pawley fitting^22^ of the patterns. Data for **3** suggested a new, unknown polymorph and prevented further analysis of this material. For **1**, **2**, **4** and **5**, a series of PXRD patterns were obtained after equilibration at sequentially incremented CO_2_ gas pressures. Pawley fitting allowed unit cell parameter determination (Supplementary Tables 12 and 15–17) and established that gated increases in volume per FU (*V*/*Z*) occur in all cases, sometimes accompanied by changes in translational symmetry, but consistent with adsorption of CO_2_.

Adsorption behaviour might be expected to resemble that documented by gravimetric adsorption measurements, but not match exactly as conditions (*T* and *p*) are different. Volume increases (Δ*V*/*Z*) provide estimates of CO_2_ adsorption^23,24^, immediately after the gate opening pressures, of 0.75–0.94 CO_2_ per FU for **1** (gate at 4.2 < *p* < 9.8 bar), 1.23–1.39 CO_2_ per FU for **2** (gate at 17.6 < *p* < 19.1 bar) and 1.93–2.41 CO_2_ per FU for **4** (gate at 24.4 < *p* < 30.0 bar). These estimates are based upon CO_2_ molecular volumes of 30.61 Å^3^ (estimated by Gavezzotti^23^) and 38.3 Å^3^ (estimated by van Heerden and Barbour^24^) as lower and upper bounds, respectively, and assume a 50% occupancy by CO_2_ of the additional volume available^24^. The implied trend is one of increasing adsorption (CO_2_ per FU) and increasing gate pressure with increasing perfluoroalkyl chain length. Comparing gating at 298 K (PXRD estimates) with gravimetric measurements (253–273 K) indicates that relative onset pressures accord well (*p*/*p*_0_: 0.09 at 253 K and 273 K versus 0.07–0.17 at 298 K for **1**; 0.23–0.24 at 253 K and 273 K versus 0.30–0.32 at 298 K for **2**; 0.34–0.35 at 253 K and 273 K for **3**; 0.45–0.63 at 258 K versus 0.52–0.59 at 263 K versus 0.53 at 273 K versus 0.42–0.51 at 298 K for **4**). PXRD studies of **5** indicate only a small volume increase (Δ*V*/*Z*) at *p* < 48.4 bar (estimated uptake 0.32–0.40 CO_2_ per FU), at which point a high-pressure gating occurs leading to a larger change in volume (estimated overall uptake 3.38–4.23 CO_2_ per FU). This behaviour is consistent with that observed in gravimetric adsorption measurements for **5**, which were limited to *p* < 19.5 bar, but suggests greater capacity is accessible at higher pressures.

Room-temperature and low-temperature synchrotron PXRD studies under CH_4_ gas pressure revealed negligible changes in unit cell parameters in the range of 1–25 bar for **1** (1–18 bar for **5**). The absence of structural changes suggests that CH_4_ is not adsorbed by AgCPs **1** and **5**, consistent with gravimetric adsorption measurements.

**Fig. 3:**
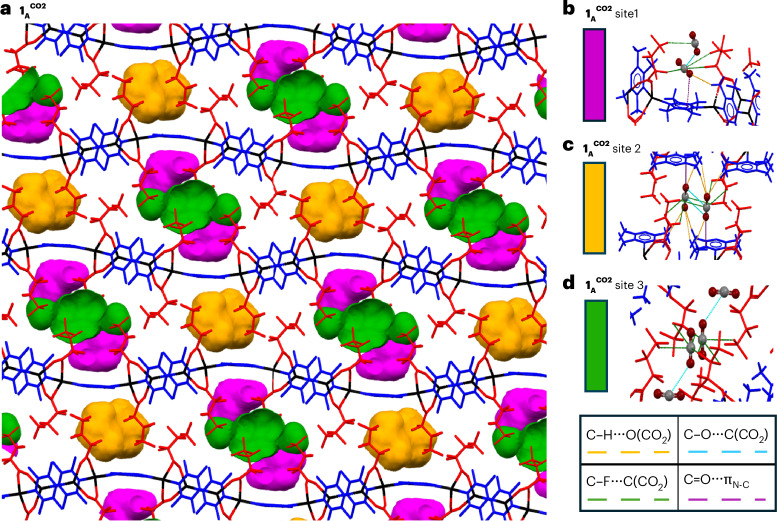
Crystal structure of CP **1**_**A**_^**CO2**^ at 232 K and 10 bar CO_2_, showing locations of CO_2_ sites and interactions with their surroundings. **a**, The three sites for adsorbed CO_2_ molecules are shown as site 1 (pink), located in region 1, and sites 2 (orange) and 3 (green), which are located in region 2. Specific CO_2_ intermolecular interactions are shown in **b**–**d**. **b**, Site 1 (central CO_2_) with neighbouring site 3 (peripheral CO_2_). **c**, Site 2, which contains an inversion-related pair of CO_2_ molecules. **d**, Site 3, which contains a single CO_2_ molecule (central) disordered over an inversion centre (both components shown) and interacting with CO_2_ molecules in neighbouring site 1 (peripheral). Atom colours are as in Fig. 1 with CO_2_ oxygen in dark red and CO_2_ carbon in grey. C=O∙·∙π_N–C_(TMP) interactions are shown as dashed purple lines, C–H···O(CO_2_) hydrogen bonds as dashed orange lines, C–F···C(CO_2_) interactions as dashed green lines and C=O···C(CO_2_) interactions in light blue. See Extended Data Fig. 2 for intermolecular interaction distances. See Supplementary Figs. 25–34 for a depiction of CO_2_ sites for AgCPs **2**–**5**.

Single-crystal X-ray diffraction (SCXRD) studies were conducted to more detailed information on the mechanism and binding sites for CO_2_ adsorption (see, gas cell/rig configuration in Supplementary Section 2 and data in Supplementary Section 3). After initial measurements under vacuum, a sequence of full crystal structure determinations were conducted under a 10-bar CO_2_ atmosphere at different temperatures, beginning at room temperature and ending at 200 K (232 K for **1**). This approach increases the relative pressure (*p*/*p*_0_) of CO_2_ while also reducing the thermal motion of the AgCP host and the CO_2_ molecules, enabling the crystallographic localization of the adsorbed CO_2_ and estimation of the adsorption capacity (Supplementary Section 3.3 and Supplementary Figs. 23 and 34). The guest-containing AgCPs **1**^**CO2**^–**4**^**CO2**^ at the lowest temperatures studied show a trend of increasing amount of CO_2_ molecules per FU (1.9, **1**^**CO2**^; 2.3, **2**^**CO2**^; 3.2, **3**^**CO2**^; and 3.4, **4**^**CO2**^) distributed across three sites (**1**^**CO2**^; Fig. 3) or four sites (**2**^**CO2**^–**4**^**CO2**^; Supplementary Figs. 26, 29, 30 and 32) per FU based upon refined occupancies for crystallographically located CO_2_ molecules. The structure of **5**^**CO2**^, however, exhibits much lower uptake (0.8 CO_2_ per FU located at a single site). The amounts of adsorbed CO_2_ determined by the three methods are of similar magnitude, despite the different approaches used, suggesting consistent adsorption behaviour.

The crystal structures of the guest-containing AgCPs **1**^**CO2**^–**5**^**CO2**^ show two chemically distinctive regions where CO_2_ gas molecules are located. Sites in the first region lie close to the single-bridge TMP ligands (for example, for **1**^**CO2**^, site 1, pink; Fig. 3), although some interaction with proximate CF_2_ groups is often present, while sites in the second region (for example, for **1**^**CO2**^, site 2, orange; site 3, green; Fig. 3) are predominantly associated with the interdigitated perfluoroalkyl ligands. Gated uptake of CO_2_ uptake is evident again, as an initial temperature reduction leads to a contraction in volume (*V*/*Z*). This suggests little or no CO_2_ uptake until a temperature is reached at which the relative pressure (*p*/*p*_0_) permits gate opening, resulting in an increase in volume and allowing CO_2_ molecule(s) to be modelled crystallographically (**1**^**CO2**^, 253 K; **2**^**CO2**^, 215 K; **3**^**CO2**^, 230 K; **4**^**CO2**^, 240 K). Given the different SCXRD approach (that is, isobaric rather than isothermal), quantitative comparisons with gating behaviour from the gravimetric adsorption and PXRD studies cannot be made, but it is important to recognize that the gating observed across all experiments is qualitatively consistent and provides an assurance of the behaviour. For **5**^**CO2**^, CO_2_ could be modelled only at 200 K, but there was no clear evidence of a volume increase as presumably any such increase (for the much smaller CO_2_ uptake) is more than offset by the volume contraction upon cooling.

In all cases (**1**^**CO2**^–**5**^**CO2**^), CO_2_ preferentially populates the sites in region 1 first, with increasing population of the sites in region 2 observed as the temperature is decreased, forming a number of short interactions of CO_2_ with neighbouring atoms of the AgCPs (Supplementary Figs. 24, 26, 29, 30, 32 and 34). CO_2_ molecules in region 1 form C–H···O(CO_2_) hydrogen bonds (with the methyl groups of TMP ligands), CO_2_∙∙∙TMP interactions (parallel π_CO2_···π_N-C_ and T-shaped C=O···π_N-C_), offset antiparallel (CO∙∙∙CO) dimers with carboxylate groups and C–F∙∙∙C(CO_2_) interactions. A π_CO2_···π_N–C_ interaction between CO_2_ and aromatic rings has been previously reported by several groups^25^ and has been described as a mixture between π–π dispersive forces and an electrostatic interaction between the electronegative aromatic π-cloud and the electropositive carbon on the CO_2_. The C=O···π_N–C_(TMP) interaction involves electron-poor regions of the conjugated ring, resembling that of anion–π interactions^26^, whereas the CO∙∙∙CO offset dimers resemble those reported for organic carbonyl groups^27^ and for self-interactions between pairs of CO_2_ molecules^28^. CO_2_ molecules in region 2 also form C=O···π_N–C_ interactions with TMP ligands but predominantly form C–F∙∙∙C(CO_2_) interactions. The latter can be described as electrostatic wherein the fluorines interact with the electropositive carbon of CO_2_ (that is, C–F^δ−^···C^δ+^), although the nature of this interaction has been debated for years, particularly in the context of explaining the high solubility of CO_2_ in perfluoroalkanes^29^.

**Extended Data Fig. 3:**
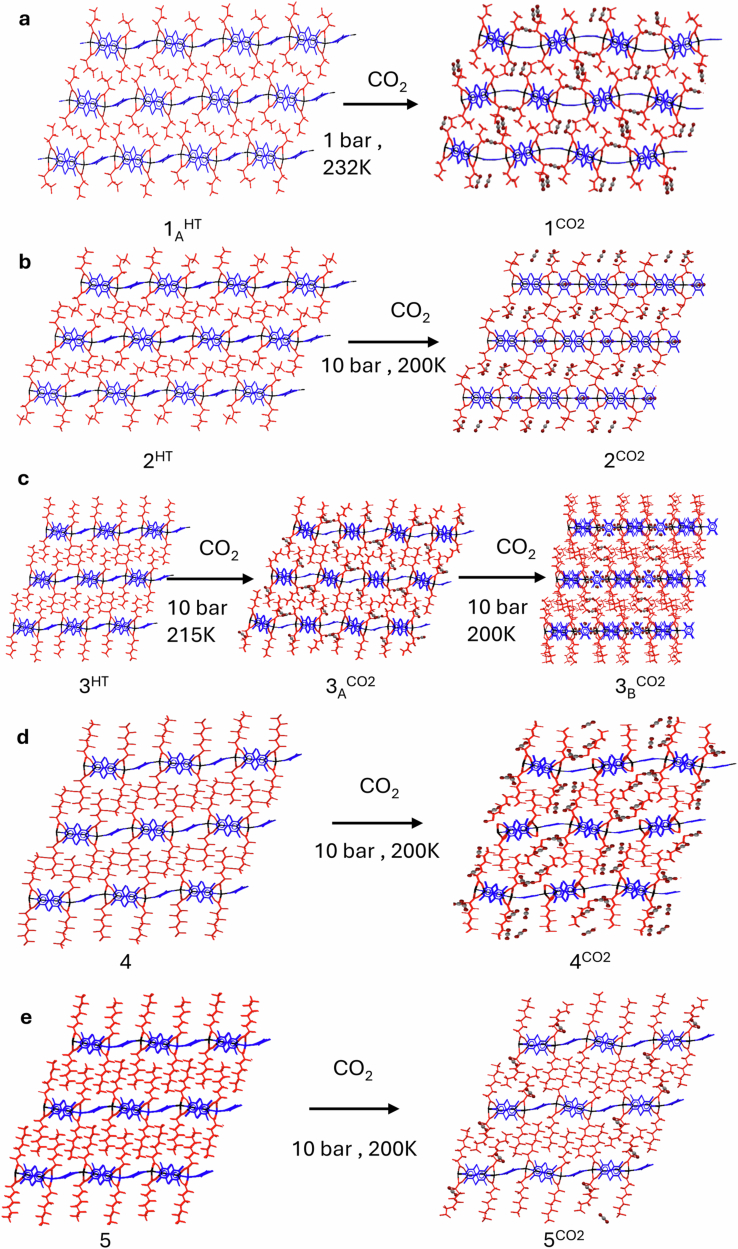
Detailed comparison of crystal structures of AgCPs 1-5 before and after CO_2_ adsorption. **a**, **1**_**A**_^**HT**^ → **1**^**CO2**^; **b**, **2**^**HT**^ → **2**^**CO2**^; **c**, **3**^**HT**^ → **3**_**A**_^**CO2**^ → **3**_**B**_^**CO2**^; **d**, **4** → **4**^**CO2**^; and **e**, **5** → **5**^**CO2**^. These comparisons illustrate the movement/rotation of the coordination polymers that accommodates the CO_2_ uptake, including conformational changes of the perfluoroalkyl groups, reorientation of TMP ligands and flexibility in the Ag(I) coordination spheres. This provides an illustration of the structural changes associated with the CO_2_ sorption process and mechanistic insight into the nonporous-to-porous transitions. Colour coding for coordination polymers is as used in Fig. 1 with CO_2_ oxygen in dark red and CO_2_ carbon in grey.

The crystal structures also reveal that the one-dimensional AgCPs **1**–**5** rearrange their structure to accommodate the CO_2_ guest molecules (Extended Data Fig. 3). Not only are the single-bridge TMP ligands able to reorient by rotation about the longitudinal N···N vector, but the flexible coordination geometry readily accessible by the d^10^ Ag(I) ions allows the single-bridging TMP ligands to pivot around the silver centres, generating pockets of space where CO_2_ molecules are accommodated (see Supplementary Section 3.3 for full descriptions). Such structural flexibility also enables the formation of several polymorphs for AgCPs **1**–**3** and **3**^**CO2**^ (conversion between **3**_**A**_^**CO2**^ and **3**_**B**_^**CO2**^) through end-to-end rotation of the TMP ligand. Indeed, we have previously exploited this process via more extensive heating to remove the single-bridge TMP ligand (for AgCPs **1** and **2**) and enable a cross-linking solid-state pathway to new two-dimensional AgCPs^17–19^.

### Dissolving CO_2_ and CO_2_/CH_4_ separation

**Fig. 4:**
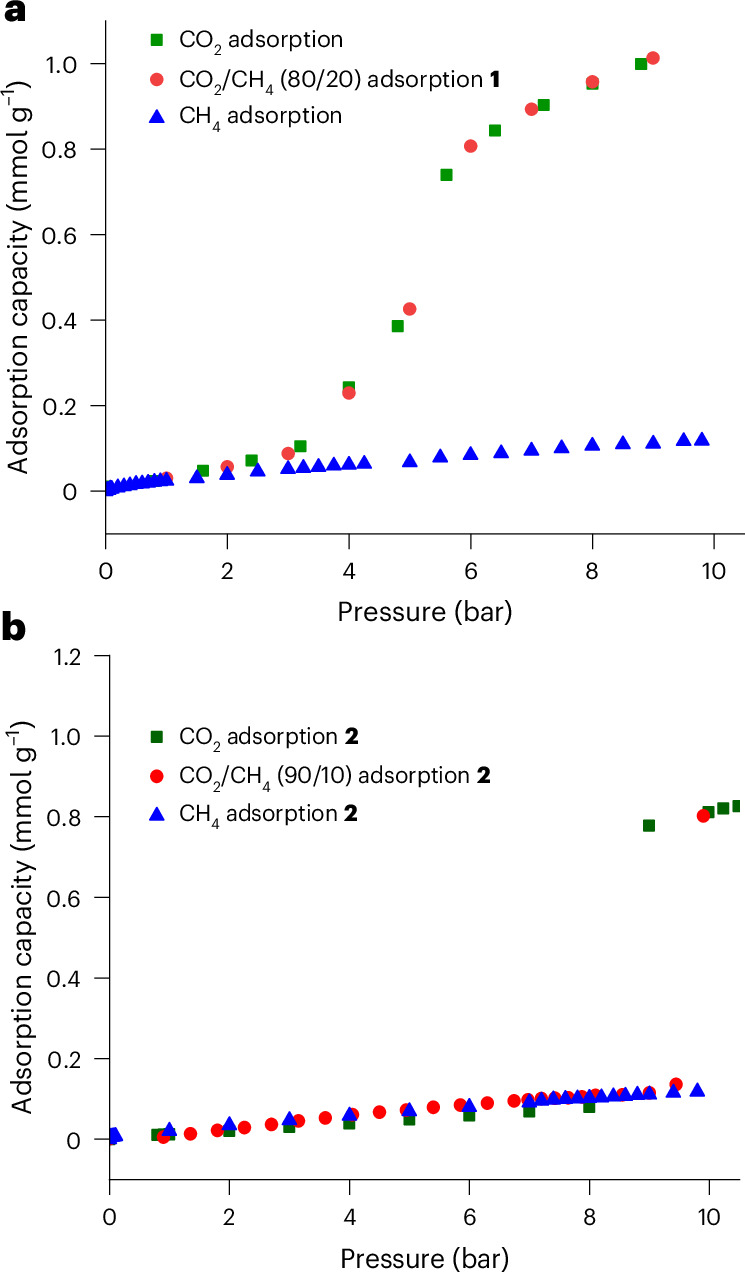
Adsorption isotherms of CH_4_, CO_2_ and CO_2_/CH_4_ mixtures for AgCPs at 273 K. **a**, Isotherms for **1. b**, Isotherms for **2**. Red circles represent CO_2_/CH_4_ competitive adsorption data for **1** (80/20) and **2** (90/10). Green squares represent single-gas CO_2_ adsorption, and blue triangles represent single-gas CH_4_ adsorption.

The combination of gravimetric adsorption data and diffraction studies has demonstrated the gated CO_2_ adsorption by the highly fluorinated, nominally non-porous AgCPs **1**–**5**, in contrast to the absence of CH_4_ adsorption (established in studies of **1**, **2** and **5**). These observations led us to investigate the separation of CO_2_ from CH_4_ by the AgCP materials. Compounds **1** and **2** were selected to explore this potential through measurement of adsorption isotherms of 80/20 and 90/10 CO_2_/CH_4_ gas mixtures for **1** and **2**, respectively. A total pressure range of 0 < *p* < 10 bar at 273 K was used to ensure that the gate pressures determined for the single-component CO_2_ adsorption isotherms were exceeded by the partial pressure of CO_2_ used. When plotted as a function of CO_2_ partial pressure (Fig. 4), the CO_2_/CH_4_ mixed gas isotherms are very similar to the single component CO_2_ isotherms, showing gated adsorption at similar gate pressures and the same overall adsorption capacities. This confirms that AgCPs **1** and **2** preferentially adsorb CO_2_ over CH_4_ at high pressures with a selectivity close to 100% in the pressure range measured.

The adsorption of CO_2_ and high selectivity of CO_2_ over CH_4_ exhibited in AgCPs **1**–**5** more closely resembles the dissolution of CO_2_ in perfluoroalkane solvents. The CO_2_ adsorption properties shown by AgCPs **1**–**5** are better described by scaled particle theory, which predicts that the solubility of a solute in a liquid is primarily influenced by the work required to generate the cavities that accommodate the solute^30^. The interdigitated, but mobile, perfluoroalkyl layers present in these materials (Fig. 1) provide a pathway for incursion and thus adsorption of CO_2_ by the AgCPs (Extended Data Fig. 3). This is consistent with the observation of conformational flexibility of the perfluoroalkyl chains that leads to polymorphism in these materials as well as enabling (transient) cavities to accommodate and transport CO_2_ molecules. Consequently, it is reasonable to attribute the gating behaviour of the present materials during CO_2_ adsorption to the need to overcome dispersion forces between the interdigitated perfluoroalkyl chains, which require greater CO_2_ pressures to overcome greater dispersion forces between the longer chains but can lead to greater overall adsorption capacity. These assertions are supported by such dispersion forces between perfluoroalkyl chains giving rise to the monotonic trend in melting points and boiling points of perfluoroalkanes^31^. The ability to adjust the gate pressure and adsorption capacity by changing the perfluoroalkyl chain length in this isoreticular series of AgCPs suggests a platform for tuning adsorption properties in these or related materials. Rational control of the pressure threshold in such type F-III or F-IV (stepped) isotherms is extremely rare, but highly desirable in designing new materials for gas separation at high pressures^10,13^. The orientational flexibility of the single-bridge TMP ligands, facilitated by the ease of deformation of the Ag(I) coordination environments, plays an important role in providing CO_2_ binding sites in region 1, but C–F∙∙∙C(CO_2_) interactions are evident in CO_2_ binding sites in both region 1 and region 2, and are most prevalent in the latter. The nature of the observed CO_2_ binding sites is consistent with the high CO_2_ solubility in perfluoroalkanes^32,33^, and the corresponding high solubility of small fluoroalkanes in liquid or supercritical CO_2_ (ref. ^34^) is well established. Indeed, the CO_2_–fluoroalkyl attractive interaction has been the subject of a number of experimental^29,35^ and computational^36^ studies, although the precise nature of the interaction remains an area of discussion^29^. Interactions between fluorine atoms and CO_2_ molecules have been exploited previously to enhance CO_2_ uptake in porous molecular materials^37^ and most notably in MOFs, such as the SIFSIX^38^ series of MOFs in which the frameworks are pillared by SiF_6_^2−^, TiF_6_^2−^ or related fluoroanions and project fluorine atoms into open pores, enabling binding of CO_2_ molecules by, for example, Si–F^δ−^···C^δ+^ interactions. Fluorinated organic ligands within MOFs have also been explored^39–47^, predominantly involving fluorinated aromatic linker ligands, including the observation of C_Ar_–F^δ−^···C^δ+^ interactions. Unlike AgCPs **1**–**5**, however, these MOFs exhibit permanent porosity that provides a direct pathway for CO_2_ uptake. More closely related to the present study are examples of postsynthetic attachment of perfluoroalkylcarboxylates to the Zr_6_ nodes of the MOF NU-1000, DUT-67 and MOF-808^45–47^. The perfluoroalkyl chains project into the cavities of the MOFs and are found to improve water stability (DUT-67) and enhance CO_2_ uptake and CO_2_ adsorption relative to N_2_ (NU-1000) and to CH_4_ (MOF-808). Computer simulations (NU-1000) also suggest that CO_2_ binding sites lie close to the Zr_6_ nodes and in the vicinity of the perfluoroalkyl groups, although specific C–F∙∙∙CO_2_ interactions are not invoked^45^. Most closely related to our approach is the study by Kitagawa and coworkers, which examines gas adsorption and the resulting structural change in two-dimensional CPs with pendant perfluoroalkyl groups, although CO_2_/CH_4_ separation is not explored^48^.

Turning to the potential for separation of CO_2_ from CH_4_, the negligible CH_4_ uptake by AgCPs **1**–**5** can be attributed to the unfavourable interactions between perfluoroalkanes and alkanes^49^, which are immiscible as liquids. The same effect is found in the solutions of small hydrocarbon gases in perfluorocarbon media, where a reduction in solubility is found compared with the solubility in analogous hydrocarbon media^50^.

The crystal structures provide accurate characterization of CO_2_ molecules interacting with perfluoroalkyl chains in which the interactions with CO_2_ are well defined. The structural insight provided therefore will be of value to further investigations of fluorinated polymers in CO_2_ adsorption and separation applications. The behaviour of AgCP **5** will require further investigation as PXRD studies suggest a gate pressure beyond the experimental limitations of our gravimetric adsorption studies is required to enable a larger CO_2_ uptake, although very small uptakes were determined within the lower accessible pressure range (*p*(CO_2_) < 20 bar at 273 K). The nature of the hystereses observed, most notably for **2** and **4**, is also an area in which further investigation would be informative.In this proof-of-principle study, we have not extensively investigated the recyclability of the materials, but initial observations are encouraging. Thus, the PXRD studies of CO_2_ adsorption by AgCPs **1**, **2**, **4** and **5** show a return to their original state after desorption, based upon unit cell dimensions (Supplementary Tables 14–17). The potential for recyclability is further reinforced for AgCP **1** by the CO_2_ adsorption and 80/20 CO_2_/CH_4_ adsorption studies (Fig. 4a), which were conducted sequentially on the same sample and exhibit the same adsorption isotherms. Overall, the crystalline nature of **1**–**5** has permitted crystallographic identification of the CO_2_ binding sites within the mobile perfluoroalkyl regions of the crystals, enabling identification of a dissolution-like process for CO_2_ uptake in a non-porous crystalline solid.

## Conclusions

CO_2_ sorption has been demonstrated by a family of highly fluorinated AgCPs **1**–**5** despite the absence of pores or channels in the crystal structures and contrasts with adsorption by highly porous fluorinated MOFs (vide supra), in which there has been considerable interest^44^. Gas uptake in **1**–**5** proceeds through a gate opening mechanism, resulting in type F-III or F-IV CO_2_ adsorption isotherms, in which the gas pressure threshold depends directly on the length of the perfluorocarboxylate chains that interdigitate to form perfluoroalkyl layers within the crystals. Gravimetric gas adsorption studies are complemented by in situ PXRD and SCXRD studies to provide structural and mechanistic insight into the sorption process.

Gas adsorption by nonporous crystals^51,52^, perhaps better described as crystals with latent porosity^53^, is known, but has received limited investigation compared with that in permanently porous materials. Gas transport in solids with latent porosity requires molecular mobility to aid transport. This is accomplished in the AgCPs by torsional flexibility of the perfluoroalkyl chains and flexibility along the CP backbone facilitated by non-directional coordination demands of the Ag(I) centres, enabling a process akin to dissolution of CO_2_ in perfluoroalkane solvents or perfluorocarbon polymers. SCXRD studies reveal the location of the CO_2_ molecule binding sites and identify interactions between CO_2_ molecules, with the single-bridge aromatic TMP ligands and prominently with the perfluoroalkyl chains via C–F^δ−^···C^δ+^ interactions.

Separation of CO_2_ from CH_4_ in gas mixtures with high selectivity at high pressure has been demonstrated and is reinforced by single-component CH_4_ adsorption studies that result in negligible uptake (type III isotherms, that is, non-wetting adsorbate) or volume changes in complementary diffraction studies. These relatively densely packed, but latently porous, materials suggest that perfluoroalkyl regions may be suitable to exploit for gas separations including tunability of gated sorption. The approach in this study has used complementary techniques to provide valuable insight into the nature of the sorption phenomena and has relevance beyond crystalline materials. It is hoped this will spur interest in similar new approaches to gas sorption and separation.

## Methods

### Materials

All reagents were purchased from Aldrich or Alfa Aesar. High-purity carbon dioxide and methane gases were supplied by BOC and used as received. CO_2_ sorption measurements were made using an Intelligent Gravimetric Analyser model 003 supplied by Hiden Isochema Ltd. Elemental analyses were conducted by the Elemental Analysis Service in the Department of Chemistry at the University of Sheffield.

### Synthesis

A homologous series of isoreticular AgCPs [Ag_4_(CO_2_(CF_2_)_*m*_CF_3_)_4_(TMP)_3_(ROH)_2_]∙*x*CH_2_Cl_2_ (*m* = 2 (**1-MeOH**), 3 (**2-EtOH**), 4 (**3-EtOH**), 5 (**4-MeOH**) and 6 (**5-MeOH**); ROH = methanol or ethanol; *x* = 0 or 1 (**5-MeOH** only); Extended Data Table 1) has been synthesized as large colourless needle crystals by a slow liquid diffusion process at 278 K. Alcohol-free AgCPs [Ag_4_(CO_2_(CF_2_)_*m*_CF_3_)_4_(TMP)_3_] (*m* = 2 (**1**), 3 (**2**), 4 (**3**), 5 (**4**) and 6 (**5**)) were obtained upon mild heating of compounds **1-ROH**–**5-ROH**. These materials were prepared following a similar procedure to those reported in ref. ^17^.

#### Synthesis of [Ag_4_(O_2_C(CF_2_)_2_CF_3_)_4_(TMP)_3_(MeOH)_2_] (1-MeOH)

Silver(I) heptafluorobutanoate (96 mg, 0.30 mmol) was dissolved in methanol (1.5 ml) and carefully layered on a solution of TMP (30 mg, 0.220 mmol) in dichloromethane (DCM) solution (1 ml). Diffusion between layers at 5 °C afforded a mixture of colourless needle and plate crystals. Separation of the needle crystals under the microscope resulted in 81% yield within 3 days. Calc.: C, 28.72; H, 2.52; N, 4.78%; found C, 28.59; H, 2.14; N, 4.91%.

#### Synthesis of [Ag_4_(O_2_C(CF_2_)_2_CF_3_)_4_(TMP)_3_] (1)

Compound **1** (in polymorphic form **1**_**A**_^**HT**^) is best synthesized by release of alcohol from **1-MeOH**. **1-MeOH** (100 mg, 0.0569 mmol) was placed in an open vial at room temperature for a week to permit MeOH vapour release. White crystals of **1**_**A**_^**HT**^ formed quantitatively. Calc.: C, 28.38; H, 2.12; N, 4.96%. Found: C, 28.17; H, 2.23; N, 4.72%. The phase purity and identity of the polymorph was confirmed by Rietveld refinement of powder X-ray diffraction data.

#### Synthesis of [Ag(O_2_C(CF_2_)_3_CF_3_)]

Silver(I) carbonate (372 mg, 1.35 mmol) was partially dissolved in 25 ml of methanol. Perfluoropentanoic acid (0.42 ml, 2.7 mmol) was added dropwise with a syringe to the methanol solution. The reaction mixture was stirred until the entire methanol was evaporated, affording 902 mg of white powder in 90% yield Calc.: C, 18.04; H, 0; N, 0%. Found: 18.34; H, 0; N, 0.21%.

#### Synthesis of [Ag_4_(O_2_C(CF_2_)_3_CF_3_)_4_(TMP)_3_(EtOH)_2_] (2-EtOH)

Silver(I) nonafluoropentanoate (100 mg, 0.29 mmol) was dissolved in ethanol (1.5 ml) and carefully layered on a solution of TMP (26 mg, 0.19 mmol) in DCM solution (1 ml). Diffusion between layers at 5 °C afforded colourless needles and plate crystals within 3 days. The presence of CP [Ag_4_(O_2_C(CF_2_)_3_CF_3_)_4_(TMP)_2_]_*n*_ as an impurity was confirmed by Rietveld refinement of powder X-ray diffraction data. Separation between colourless needles and plates enabled purification of **2-EtOH**.

#### Synthesis of [Ag_4_(O_2_C(CF_2_)_3_CF_3_)_4_(TMP)_3_] (2)

Compound **2** is best synthesized by the release of ethanol from **2-EtOH** CP. Crystals of **2-EtOH** (100 mg, 0.0503 mmol) that were previously selected under the microscope were placed in an open vial in an oven at 60 °C for 3 h to permit ethanol vapour release. White crystals of **2** formed quantitatively. Calc.: C, 27.93; H, 1.92; N, 4.44%. Found: C, 28.17; H, 2.23; N, 4.72%.

#### Synthesis of [Ag(O_2_C(CF_2_)_4_CF_3_)]

Silver(I) carbonate (328 mg, 1.2 mmol) was partially dissolved in 25 ml of methanol. Perfluorohexanoic acid (0.42 ml, 2.37 mmol) was added dropwise with a syringe to the methanol solution. The reaction mixture was stirred until all methanol was evaporated, affording 847 mg of white powder in 75% yield Calc.: C, 17.10; H, 0; N, 0%. Found: C, 17.05; H, 0; N, 0.24 %.

#### Synthesis of [Ag_4_(O_2_C(CF_2_)_4_CF_3_)_4_(TMP)_3_(EtOH)_2_] (3-EtOH)

Silver(I) undecafluorohexanoate (100 mg, 0.24 mmol) was dissolved in ethanol (1.5 ml) and carefully layered on a solution of TMP (24 mg, 0.18 mmol) in DCM solution (1 ml). Diffusion between layers at 5 °C afforded colourless needles and plates. Separation of the needle crystals under the microscope resulted in 72% yield within 3 days. Calc.: C, 28.53; H, 2.22; N, 3.85%. Found: C, 28.04; H, 1.89; N, 3.71 %.

#### Synthesis of [Ag_4_(O_2_C(CF_2_)_4_CF_3_)_4_(TMP)_3_] (3)

Compound **3** is best synthesized by release of the alcohol molecule from **3-EtOH** CP. Crystals of **3-EtOH** (100 mg, 0.0457 mmol) were placed in an open vial in an oven for 3 h at 60 °C to permit EtOH vapour release. White crystals of **3** formed quantitatively. Calc.: C, 27.56; H, 1.73; N, 4.02%. Found: C, 27.65; H, 1.42; N, 3.91 %.

#### Synthesis of [Ag(O_2_C(CF_2_)_5_CF_3_)]

Silver(I) carbonate (292 mg, 1.1 mmol) was partially dissolved in 25 ml of methanol. Perfluoroheptanoic acid (771 mg, 2.12 mmol) was added and stirred until all methanol was evaporated, affording 843 mg of white powder in 84% yield. Calc.: C, 17.83; H, 0; N, 0%. Found: C, 17.71; H, 0; N, 0%.

#### Synthesis of [Ag_4_(O_2_C(CF_2_)_5_CF_3_)_4_(TMP)_3_(MeOH)_2_] (4-MeOH)

Silver(I) tridecafluoroheptanoate (100 mg, 0.21 mmol) was dissolved in methanol (1.5 ml) and carefully layered on a solution of TMP (21 mg, 0.16 mmol) in DCM solution (1 ml). Diffusion between layers at 5 °C afforded colourless needles and plates. Separation of the needle crystals under the microscope resulted in 86% yield within 3 days. Calc.: C, 27.52; H, 1.88; N, 3.57%. Found: C, 27.77; H, 2.04; N, 3.75%.

#### Synthesis of [Ag_4_(O_2_C(CF_2_)_4_CF_3_)_4_(TMP)_3_] (4)

Compound **4** is best synthesized by the release of the methanol molecules from **4-MeOH**. Crystals of **4-MeOH** (100 mg, 0.0436 mmol) were placed in an open vial in an oven for 3 h at 60 °C to permit MeOH vapour release. White crystals of **4** formed quantitatively. Calc.: C, 27.25; H, 1.58; N, 3.67%. Found: C, 27.27; H, 1.16; N, 3.58 %.

#### Synthesis of [Ag(O_2_C(CF_2_)_6_CF_3_)]

Silver(I) carbonate (264 mg, 0.96 mmol) was partially dissolved in 25 ml of methanol. Perfluorooctanoic acid (790 mg, 1.91 mmol) was added and stirred until all methanol was evaporated, affording 962 mg of white powder in 96% yield. Calc.: C, 18.42; H, 0; N, 0%. Found: C, 18.16; H, 0; N, 0.43%.

#### Synthesis of [Ag_4_(O_2_C(CF_2_)_6_CF_3_)_4_(TMP)_3_(MeOH)_2_]·CH_2_Cl_2_ (5-MeOH)

Silver(I) perfluorooctanoate (100 mg, 0.19 mmol) was dissolved in methanol (1.5 ml) and carefully layered on a solution of TMP (18 mg, 0.13 mmol) in DCM solution (1 ml). Diffusion between layers at 5 °C afforded colourless needles and plates. Separation of the needle crystals under the microscope resulted in 86% yield within 3 days. Calc.: C, 26.83; H, 1.75; N, 3.18%. Found: C, 26.79; H, 1.29; N, 3.02 %

#### Synthesis of [Ag_4_(O_2_C(CF_2_)_4_CF_3_)_4_(TMP)_3_] (5)

Compound **5** is best synthesized by the release of ethanol molecules from **5-MeOH**. Crystals of **5-MeOH** (100 mg, 0.0386 mmol) were placed in an open vial in an oven for 3 h at 60 °C to permit MeOH vapour release. White crystals of **5** formed quantitatively. Calc.: C, 26.99; H, 1.46; N, 3.37%. Found: C, 26.79; H, 1.29; N, 3.23 %.

### SCXRD

#### Crystal structure determination and refinement

X-ray data were processed and reduced using APEX2 or CrysAlisPro programs and corrected for absorption using empirical methods (SADABS^54^ or Scale3 Abspack) based upon symmetry-equivalent reflections combined with measurements at different azimuthal angles. All crystal structures were solved and refined against all *F*^2^ values using OLEX2^55^ and/or SHELX^56^. A summary of the data collection and structure refinement information is provided in Supplementary Tables 1–11. Non-hydrogen atoms were refined anisotropically where possible, whereas hydrogen atoms were placed in calculated positions, refined using idealized geometries (riding model) and assigned fixed isotropic displacement parameters. In many of the structure determinations, fluoroalkyl chains are described as disordered over two orientations with carbon and fluorine atoms modelled using isotropic displacement parameters. Distance restraints (SAME, SADI and DFIX commands in SHELX) were applied to C–F and C–C bonds. Atomic displacement parameters of atoms in the perfluoroalkyl chains were restrained using a rigid-body approach by applying SHELX RIGU commands and restrained to be similar in magnitudes using SHELX SIMU commands.

#### SCXRD during gas sorption

A series of SCXRD experiments have been undertaken using a gas rig and a gas cell on a dual-source Rigaku Synergy FR-X rotating anode diffractometer (Supplementary Section 2) and at beamline I19 at Diamond Light Source^57^, to investigate the adsorption of CO_2_ gas molecules in single crystals of **1**–**5**. After initial crystal structure determinations, each crystal was exposed to 10 bar CO_2_ pressure. A sequence of data collections was undertaken on crystals of **1**–**5** whereby the temperature was progressively decreased to 200 K (232 K for **1**). In the crystal structure models, CO_2_ molecules were located from difference electron density maps and refined as rigid bodies with unconstrained occupancies. Isotropic displacement parameters for atoms of the CO_2_ molecules were constrained to *U*_iso_ = 0.2 Å^2^.

Data collected for compound **1** under CO_2_ atmospheres (**1**^**CO2**^) resulted in a low completeness (~91%) as the strongest reflections collected were treated as detector overloads and omitted from refinements. Data collected for compound **2**^**CO2**^ (200 K, 10 bar CO_2_), **3**_**B**_^**CO2**^ (200 K, 10 bar CO_2_) and **4**^**CO2**^ (200 K, 10 bar CO_2_) had lower resolutions than other data (*d*_min_ ≈ 1 Ǻ). Crystal data are summarized in Supplementary Tables 1–9

### PXRD during gas sorption

The structural changes associated with CO_2_ gas sorption by compounds **1**, **2**, **4** and **5**, and CH_4_ gas sorption by compounds **1** and **5** were monitored in situ by powder X-ray diffraction at beamline ID31^58,59^ at the European Synchrotron Radiation Source (ESRF) using a nine-channel multi-analyser crystal detector and at beamline I11 at the Diamond Light Source using a wide-angle (90°) position-sensitive detector comprising 18 Mythen-2 modules^60,61^. For data collected using the position-sensitive detector, a pair of scans related by a 0.25° detector offset were collected for each measurement to account for gaps between detector modules. All data were collected at room temperature for studies of CO_2_ adsorption, whereas for CH_4_ adsorption data were collected at both room temperature and 180 K. Thirty-minute intervals between measurements were used to allow for equilibration of the structure along the capillary at each pressure. A sample of colourless microcrystalline **1**, **2**, **4** and **5**, synthesized as previously described, was lightly ground using an agate pestle and mortar and loaded into a 0.8-mm quartz capillary. The open end of the capillary was attached via tightened Swagelok compression fittings to the stainless-steel tubing of a gas handling rig that was further connected via a series of taps to a lecture bottle of CO_2_ or CH_4_ gas, a turbomolecular pump and an exhaust vent. The rig, including the capillary, was first evacuated taking care not to disturb the powder in the capillary.

For **1**, PXRD scans were collected (−2.5 ≤ 2*θ* ≤ 12.5°) at beamline ID31 (*λ* = 0.39998 Å) at ESRF using a scan speed of 6° min^−1^, during which the capillary was oscillated about its axis through an angle of approximately 150°. One sequence of scans was collected at room temperature (*p*_CO2_: 0, 1.01, 4.21, 9.71, 19.71, 50.23, 19.51, 10.05, 4.31 and 1.13 bar; *p*_CH4_: 0, 19.71, 42.00, 18.35, 8.81, 4.31 and 1.13 bar). In a second study (CH_4_ only), PXRD patterns were obtained at 180 K for CH_4_ pressures of 2, 5 and 25 bar. For CP **2**, 10-s PXRD scans were measured at beamline I11 (*λ* = 0.826741(1) Å) at Diamond Light Source, during which the capillary was oscillated about its axis through an angle of approximately 35°. All data were collected at room temperature (*p*_CO2_: 0, 1.19, 4.35, 5.15, 6.78, 7.68, 8.75, 11.07, 12.37, 15.52, 17.58, 19.16, 22.18, 49.23, 22.28, 18.55, 16.62, 14.33, 12.55, 10.33, 7.92, 5.05 and 1.4 bar). For CP **4**, 10-s PXRD scans were measured at beamline I11(*λ* = 0.826179(1) Å) at Diamond Light Source, during which the capillary was oscillated about its axis through an angle of approximately 35°. All data were collected at room temperature (*p*_CO2_: 0, 1.13, 10.25, 14.54, 19.99, 24.40, 30.01, 49.90, 28.60, 25.05, 20.10, 10.31 and 1.04 bar). For CP **5**, 10-s PXRD scans were measured at beamline I11(*λ* = 0.826179(1) Å) at Diamond Light Source, during which the capillary was oscillated about its axis through an angle of approximately 35°. All data were collected at room temperature (*p*_CO2_: 0, 1.02, 9.89, 14.89, 20.17, 24.24, 28.99, 38.53, 48.37, 49.41, 20.71, 15.60, 9.72 and 1.04 bar). In a separate experiment, data were collected at room temperature (295 K) and 180 K, each in a sequence of CH_4_ gas pressures, (295 K, air; 180 K, *p*_CH4_: 4.99, 9.63, 14.24, 18.63, 14.19, 10.14, 5.23 and 1.05 bar). All powder patterns were indexed and unit cell values were determined through Pawley refinement using TOPAS^62–64^.

### Volumetric and gravimetric gas sorption

Volumetric adsorption measurements were undertaken using a Micromeritics ASAP2420 Accelerated Surface Area and Porosimetry System. The samples were degassed under vacuum overnight before analysis, performed using nitrogen adsorption at 77 K (99.99% nitrogen adsorbate). A total of 49 points were taken on the adsorption branch and 30 on the desorption branch. Gravimetric adsorption measurements were recorded using an Intelligent Gravimetric Analyser model 003 supplied by Hiden Isochema Ltd. The balance and pressure control system of the instrument are fully thermostatted to 0.1 K and the microbalance has a weighing resolution of 0.2 μg. Samples (**1**–**5**) were outgassed until they reached a constant mass, at a pressure of <10^−6^ mbar at 273 K. During measurements, the pressure of the gas was gradually increased over ~15 s avoiding disruption to the microbalance. Pressure control used a 0–2 MPa pressure transducer, with an accuracy of 0.02 MPa. The pressure was maintained at the set point by active computer control. The mass uptake was measured as a function of time and the approach to equilibrium monitored in real time with a computer algorithm. After equilibrium was established, the pressure of gas in the system was increased to the next set pressure value and the subsequent uptake was measured until equilibrium was re-established. Pressure steps in the range of 0–20 bar were used to obtain CO_2_ isotherms for all compounds at 273 K. Additional CO_2_ isotherms were measured for samples **1**–**3** and **5** at 253 K and for **4** at 258 K and 263 K. Pressure steps in the range of 0–10 bar were used to obtain CH_4_ isotherms for compounds **1** and **2** at 273 K. Further mixed gas isotherms (90/10 or 80/20 CO_2_/CH_4_) were measured (0–10 bar) for compounds **1** and **2** at 273 K to investigate selectivity between the two gases. During gas adsorption, the sample temperature was maintained using a thermostirrer and was constantly monitored throughout the duration of the experiment. All measurements were made using task-specific software, supplied by Hiden Isochema Ltd.

## Online content

Any methods, additional references, Nature Portfolio reporting summaries, source data, extended data, supplementary information, acknowledgements, peer review information; details of author contributions and competing interests; and statements of data and code availability are available at 10.1038/s41557-025-01943-4.

## Supplementary information

Supplementary Information Supplementary Sections 1–8, including Supplementary discussion, Figs. 1–135, Tables 1–19 and References 1–13.

## Figures and Tables

**Extended Data Fig. 2:**
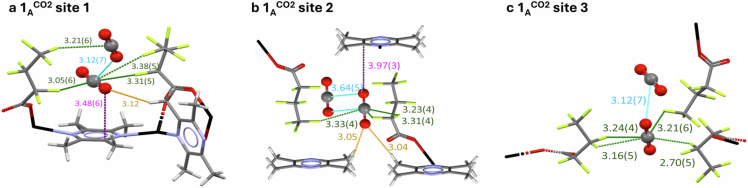
Detailed representation of the interactions between CO_2_ and coordination polymer 1_A_^CO2^. **a**, Interactions of CO_2_ at site 1; **b**, Interactions of CO_2_ at site 2; and **c**, Interactions of CO_2_ at site 3. All are shown here with symmetry-equivalent interactions (presented in Fig. 3) removed to simply the representation and are annotated with pertinent intermolecular contact distances (Å). Colour code for coordination polymer: Ag in black, C in grey, N in blue, F in green, H in white, O in red. Colour code for dashed lines that represent intermolecular interactions: C = O ∙ · ∙ π_N-C_(TMP) interactions shown as purple dashed lines, C − H···O(CO_2_) hydrogen bonds as orange dashed lines, C − F···C(CO_2_) interactions as green dashed lines and C = O ···C(CO_2_) interactions in light blue.

## Data Availability

All of the relevant data that support the findings of this research are available within the article and its Supplementary Information. Crystallographic data for crystal structures reported in the article are available free of charge from the Cambridge Crystallographic Data Centre (https://www.ccdc.cam.ac.uk/structures) under deposition numbers CCDC 903747 (**1-MeOH**), 903751 (**1**_**A**_^**HT**^), 1044595 (**1**_**A**_^**LT**^), 1044597 (**1**_**B**_^**HT**^) 1044596 (**1**_**B**_^**LT**^), 2329010 (**1**_**A**_^**HT**^ at 298 K, 2 bar CO_2_), 2329012 (**1**^**CO2**^ at 253 K, 10 bar CO_2_), 2329013 (**1**^**CO2**^ at 232 K, 10 bar CO_2_), 654182 (**2-EtOH**), 654183 (**2**^**LT**^), 2329019 (**2**^**HT**^ at 298 K, vacuum), 2329018 (**2**^**HT**^ at 273 K, 10 bar CO_2_), 2329017 (**2**^**HT**^ at 240 K, 10 bar CO_2_), 2329015 (**2**^**CO2**^ at 215 K, 10 bar CO_2_), 2329016 (**2**^**CO2**^ at 200 K, 10 bar CO_2_), 2329020 (**2**^**HT**^ at 298 K, vacuum_2), 2329007 (**3-EtOH**), 2329008 (**3**^**LT**^), 2329025 (**3**^**HT**^ at 343 K, vacuum), 2329022 (**3**^**HT**^ at 298 K, 10 bar CO_2_), 2329024 (**3**^**HT**^ at 250 K, 10 bar CO_2_), 2329023 (**3**_**A**_^**CO2**^ at 230 K, 10 bar CO_2_), 2329026 (**3**_**A**_^**CO2**^ at 215 K, 10 bar CO_2_), 2329027 (**3**_**B**_^**CO2**^ at 200 K, 10 bar CO_2_), 2329021 (**3**^**HT**^ at 298 K, 1 bar CO_2_), 2329006 (**4-MeOH**), 2329028 (**4** at 273 K, vacuum), 2329032 (**4** at 273 K, 10 bar CO_2_), 2329034 (**4**^**CO2**^ at 240 K, 10 bar CO_2_), 2329029 (**4**^**CO2**^ at 230 K, 10 bar CO_2_), 2329033 (**4**^**CO2**^ at 215 K, 10 bar CO_2_), 2329031 (**4** at 273 K, vacuum_2), 2329030 (**4**^**CO2**^ at 200 K, 10 bar CO_2_), 2329009 (**5-MeOH**), 2329038 (**5** at 295 K, vacuum), 2329037 (**5** at 295 K, 10 bar CO_2_), 2329036 (**5** at 250 K, 10 bar CO_2_), 2329035 (**5** at 230 K, 10 bar CO_2_) and 2329039 (**5**^**CO2**^ at 200 K, 10 bar CO_2_). Experimental datasets for adsorption experiments are available via the University of Strathclyde Data repository at 10.15129/e8d86890-f353-4357-afce-aa2007f8a866. Experimental datasets for PXRD studies are available via the University of Sheffield ORDA data repository at 10.15131/shef.data.29620916.

## Extended data

**Extended Data Table 1:** Chemical formulae of compounds

Compound abbreviation	Chemical formula
**1-MeOH**	[Ag_4_(O_2_C(CF_2_)_2_CF_3_)_4_(TMP)_3_(MeOH)_2_]
**1**	[Ag_4_(O_2_C(CF_2_)_2_CF_3_)_4_(TMP)_3_]
**1** ^**CO2**^	[Ag_4_(O_2_C(CF_2_)_2_CF_3_)_4_(TMP)_3_]∙*x*CO_2_
**2-EtOH**	[Ag_4_(O_2_C(CF_2_)_3_CF_3_)_4_(TMP)_3_(EtOH)_2_]
**2**	[Ag_4_(O_2_C(CF_2_)_3_CF_3_)_4_(TMP)_3_]
**2** ^**CO2**^	[Ag_4_(O_2_C(CF_2_)_3_CF_3_)_4_(TMP)_3_]∙*x*CO_2_
**3-EtOH**	[Ag_4_(O_2_C(CF_2_)_4_CF_3_)_4_(TMP)_3_(EtOH)_2_]
**3**	[Ag_4_(O_2_C(CF_2_)_4_CF_3_)_4_(TMP)_3_]
**3** ^**CO2**^	[Ag_4_(O_2_C(CF_2_)_4_CF_3_)_4_(TMP)_3_]∙*x*CO_2_
**4-MeOH**	[Ag_4_(O_2_C(CF_2_)_5_CF_3_)_4_(TMP)_3_(MeOH)2]
**4**	[Ag_4_(O_2_C(CF_2_)_5_CF_3_)_4_(TMP)_3_]
**4** ^**CO2**^	[Ag_4_(O_2_C(CF_2_)_5_CF_3_)_4_(TMP)_3_]∙*x*CO_2_
**5-MeOH**	[Ag_4_(O_2_C(CF_2_)_6_CF_3_)_4_(TMP)_3_(MeOH)_2_]∙CH_2_Cl_2_
**5**	[Ag_4_(O_2_C(CF_2_)_6_CF_3_)_4_(TMP)_3_]
**5** ^**CO2**^	[Ag_4_(O_2_C(CF_2_)_6_CF_3_)_4_(TMP)_3_]∙*x*CO_2_
