# Supplementary material for: Selective CO2 uptake mimics dissolution in highly fluorinated non-porous crystalline materials
Source: Nat Chem. 2025 Oct 14;17(11):1705–11. doi: 10.1038/s41557-025-01943-4 (PMC12580317; doi:10.1038/s41557-025-01943-4)
Supplement: Supplementary file 1 — Supplementary Sections 1–8, including Supplementary discussion, Figs. 1–135, Tables 1–19 and References 1–13. [file 41557_2025_1943_MOESM1_ESM.pdf]

nature chemistry

Supplementary information https://doi.org/10.1038/s41557-025-01943-4

Selective CO_2 uptake mimics dissolution in highly fluorinated non-porous crystalline materials

In the format provided by the authors and unedited

Table of Contents

1. Synthesis of coordination polymers 1-ROH−5-ROH and 1−5 ....S2

2. Gas rig and gas cell S3

2.1 Gas rig S3

2.2 Gas cell S6

3. Single-crystal X-ray diffraction S10

3.1 Data collection, structure solution and refinement and crystal data S10

3.2 Crystal structures and solid-vapour reaction manifold in coordination polymers 1-5-ROH and 1-5 ....S20

3.3 CO_2 adsorption in coordination polymers 1-5 studied by single-crystal X-ray diffraction (SCXRD) S31

4. *In situ* powder X-ray diffraction studies of coordination polymers 1, 2, 4 and 5 under exposure to CO_2 and CH_4 gas pressure S47

4.1 *In situ* PXRD studies of coordination polymer 1 exposure to CO_2 and CH_4 gas pressure S47

4.2 *In situ* PXRD study of coordination polymer 2 exposure to CO_2 gas pressure S56

4.3 *In situ* PXRD study of coordination polymer 4 exposure to CO_2 gas pressure S66

4.4 *In situ* PXRD study of coordination polymer 5 exposure to CO_2 and CH_4 gas pressure S72

5. Gas sorption for coordination polymers 1-5 ....S81

5.1 N_2 adsorption isotherms for coordination polymers 1, 2, 4 and 5 ....S81

5.2 CO_2 and CH_4 adsorption isotherms for coordination polymers 1-5 ....S82

6. CheckCIF A and B alerts........................S92

7. Displacement ellipsoid plots for single-crystal structures........................S93

8. References........................S106

1. Synthesis of coordination polymers 1-ROH−5-ROH and 1−5.

General. All reagents were purchased from Aldrich, or Alfa Aesar. High-purity carbon dioxide and methane gases supplied by BOC and used as received. CO_2 sorption measurements were made using an Intelligent Gravimetric Analyser (IGA) model 003 supplied by Hiden Isochema Ltd. Elemental analyses were conducted by the Elemental Analysis Service in the Department of Chemistry at University of Sheffield.

Synthesis of $[Ag_4(O_2C(CF_2)_2CF_3)_4(TMP)_3(MeOH)_2]$ (1-MeOH). Silver(I) heptafluorobutanoate (96 mg, 0.30 mmol) was dissolved in methanol (1.5 mL) and carefully layered on a solution of 2,3,4,5-tetramethylpyrazine (TMP) (30 mg, 0.220 mmol) in dichloromethane (DCM) solution (1 mL). Diffusion between layers at 5 °C afforded colourless needles and plate crystals. Separation of needles under a microscope resulted in 81% yield within 3 days. Anal. Calc. ($C_{42}H_{44}F_{28}N_6O_{10}Ag_4$): C, 28.72; H, 2.52; N, 4.78%; found C, 28.59; H, 2.14; N, 4.91%.

Synthesis of $[Ag_4(O_2C(CF_2)_2CF_3)_4(TMP)_3]$ (1). Compound **1** (in polymorphic form $\mathbf{1_A}^{\mathbf{HT}}$) is best synthesized by release of alcohol from **1-MeOH**. **1-MeOH** (100 mg, 0.0569 mmol) was placed in an open vial at room temperature for a week to permit MeOH vapour release. White crystals of $\mathbf{1_A}^{\mathbf{HT}}$ formed quantitatively. Calc.: C, 28.38; H, 2.12; N, 4.96%. Found: C, 28.17; H, 2.23; N, 4.72%. The phase purity and identity of the polymorph was confirmed by Rietveld refinement of X-ray powder diffraction data.

Synthesis of $[Ag(O_2C(CF_2)_3CF_3)]$. Silver(I) carbonate (372 mg, 1.35 mmol) was partially dissolved in 25 ml of methanol. Perfluoropentanoic acid (0,42 ml, 2.7 mmol) was added dropwise with a syringe to the methanol solution. The reaction mixture was stirred until the entire methanol was evaporated, affording 902 mg of white powder in 90% yield Calc.: C, 18.04; H, 0; N, 0%. Found: 18.34; H, 0; N, 0.21%.

Synthesis of $[Ag_4(O_2C(CF_2)_3CF_3)_4(TMP)_3(EtOH)_2]$ (2-EtOH). Silver(I) nonafluoropentanoate (100 mg, 0.29 mmol) was dissolved in ethanol (1.5 mL) and carefully layered on a solution of TMP (26 mg, 0.19 mmol) in DCM solution (1 mL). Diffusion between layers at 5 °C afforded colourless needles and plate crystals within 3 days. The presence of coordination polymer $[Ag_4(O_2C(CF_2)_3CF_3)_4(TMP)_2]_n$ as impurity was confirmed by Rietveld refinement of X-ray powder diffraction data. The separation of needle and plate crystals resulted in the purification of **2-EtOH** compound.

Synthesis of $[Ag_4(O_2C(CF_2)_3CF_3)_4(TMP)_3]$ (2). Compound **2** is best synthesized by the release of ethanol from **2-EtOH** coordination polymer. Crystals of **2-EtOH** (100 mg, 0.0503 mmol), that were previously selected under the microscope, were placed in an open vial in an oven at 60 °C for 3 hours to permit ethanol vapour release. White crystals of **2** formed quantitatively. Calc.: C, 27.93; H, 1.92; N, 4.44%. Found: C, 28.17; H, 2.23; N, 4.72%.

Synthesis of $[Ag(O_2C(CF_2)_4CF_3)]$. Silver(I) carbonate (328 mg, 1.2 mmol) was partially dissolved in 25 ml of methanol. Perfluorohexanoic acid (0.42 ml, 2.37 mmol) was added dropwise with a syringe to the methanol solution. The reaction mixture was stirred until all methanol was evaporated, affording 847 mg of white powder in 75% yield Calc.: C, 17.10; H, 0; N, 0%. Found: C, 17.05; H, 0;N, 0.24 %.

Synthesis of $[Ag_4(O_2C(CF_2)_4CF_3)_4(TMP)_3(EtOH)_2]$ (3-EtOH). Silver(I) undecafluorohexanoate (100 mg, 0.24 mmol) was dissolved in ethanol (1.5 mL) and carefully layered on a solution of TMP (24 mg, 0.18 mmol) in DCM solution (1 mL). Diffusion between layers at 5 °C afforded colourless needles and plate crystals. Separation of needle and plate crystals resulted in 72% yield within 3 days. Calc.: C, 28.53; H, 2.21; N, 3.85%. Found: C, 28.04; H, 1.89; N, 3.71 %.

Synthesis of [$Ag_4(O_2C(CF_2)_4CF_3)_4(TMP)_3$] (3). Compound **3** is best synthesized by release of the alcohol molecule from **3-EtOH** coordination polymer. Crystals of **3-EtOH** (100 mg, 0.0457 mmol) were placed in an open vial in an oven for 3 hours at 60° C to permit EtOH vapour release. White crystals of **3** formed quantitatively. Calc.: C, 27.56; H, 1.73; N, 4.02%. Found: C, 27.65; H, 1.42; N, 3.91 %.

Synthesis of [$Ag(O_2C(CF_2)_5CF_3)$]. Silver(I) carbonate (292 mg, 1.1 mmol) was partially dissolved in 25 ml of methanol. Perfluoroheptanoic acid (771 mg, 2.12 mmol) was added and stirred until all methanol was evaporated, affording 843 mg of white powder in 84% yield. Calc.: C, 17.83; H, 0; N, 0%. Found: C, 17.71; H, 0; N, 0%.

Synthesis of [$Ag_4(O_2C(CF_2)_5CF_3)_4(TMP)_3(MeOH)_2$] (4-MeOH). Silver(I) tridecafluoroheptanoate (100 mg, 0.21 mmol) was dissolved in methanol (1.5 mL) and carefully layered on a solution of TMP (21 mg, 0.16 mmol) in DCM solution (1 mL). Diffusion between layers at 5 °C afforded colourless needles and plate crystals. Separation of the needle crystals resulted in 86% yield within 3 days. Calc.: C, 27.52; H, 1.88; N, 3.57%. Found: C, 27.77; H, 2.04; N, 3.75%.

Synthesis of [$Ag_4(O_2C(CF_2)_4CF_3)_4(TMP)_3$] (4). Compound **4** is best synthesized by the release of the methanol molecules from **4-MeOH**. Crystals of **4-MeOH** (100 mg, 0.0436 mmol) were placed in an open vial in an oven for 3 hours at 60° C to permit MeOH vapour release. White crystals of **4** formed quantitatively. Calc.: C, 27.25; H, 1.58; N, 3.67%. Found: C, 27.27; H, 1.16; N, 3.58 %.

Synthesis of [$Ag(O_2C(CF_2)_6CF_3)$]. Silver(I) carbonate (264 mg, 0.96 mmol) was partially dissolved in 25 ml of methanol. Perfluorooctanoic acid (790 mg, 1.91 mmol) was added dropwise with a syringe. The reaction mixture was stirred until all methanol was evaporated, affording 962 mg of white powder in 96% yield Calc.: C, 18.42; H, 0; N, 0%. Found: C, 18.16; H, 0; N, 0.43%.

Synthesis of [$Ag_4(O_2C(CF_2)_6CF_3)_4(TMP)_3(MeOH)_2$]·$CH_2Cl_2$ (5-MeOH). Silver(I) perfluorooctanoate (100 mg, 0.19 mmol) was dissolved in methanol (1.5 mL) and carefully layered on a solution of TMP (18 mg, 0.13 mmol) in DCM solution (1 mL). Diffusion between layers at 5 °C afforded colourless needles and plate crystals. Separation of colourless needles resulted in 86% yield within 3 days. Calc.: C, 26.83; H, 1.75; N, 3.18%. Found: C, 26.79; H, 1.29; N, 3.02 %

Synthesis of [$Ag_4(O_2C(CF_2)_4CF_3)_4(TMP)_3$] (5). Compound **5** is best synthesized by the release of ethanol molecules from **5-MeOH**. Crystals of **5-MeOH** (100 mg, 0.0386 mmol) were placed in an open vial in an oven for 3 hours at 60° C to permit MeOH vapour release. White crystals of **5** formed quantitatively. Calc.: C, 26.99; H, 1.46; N, 3.37%. Found: C, 26.79; H, 1.29; N, 3.23 %

2. Gas rig and gas cell

2.1 Gas rig

A gas rig was designed and constructed for use in conjunction with a gas cell in single-crystal X-ray diffraction experiments under gas atmospheres at University of Manchester (UoM). The design was inspired by the gas rig reported by Hill[S1] and deployed at beamline ID31 (now ID22) at the European Synchrotron Radiation Source (ESRF). Supplementary Figure 1 shows the UoM gas rig mounted on a trolley. The rig comprises 9 pneumatic valves (Swagelok SS-HBV51-C) and two Brooks GF40 mass flow controllers connected by ¼ inch Swagelok VCR to form a rigid structure capable of working under vacuum and to pressures of 100 bar (all parts except for the mass flow controllers are safety rated to >200 bar).

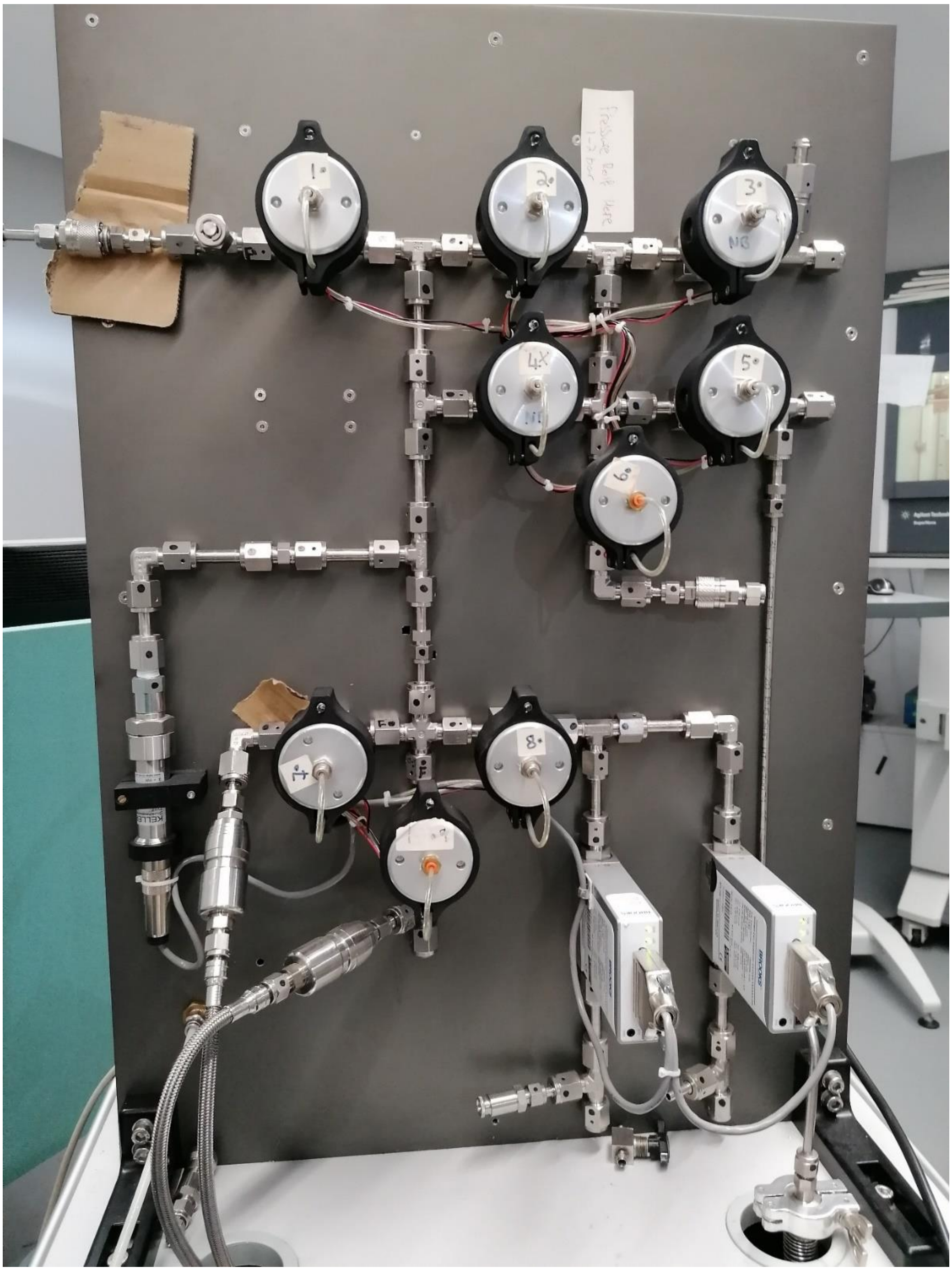

Supplementary Figure 1. The UoM gas rig mounted on a trolley in the XRD lab of the Chemistry Department at the University of Manchester

The instrument is capable of operating in two different working modes using dynamic or static gas pressures. The rig can be divided into 4 different sections identified by their functions: (1) the inlet section is formed by valves 7-9 and two gas flow controllers. Valves 7 and 9 controls the amount of static gas pressure that is introduced into the main body of the rig. Two mass flow controllers linked to a pneumatic valve (valve 8) allow exposure of the sample to a dynamic gas mixture up to 10 bar. The pressure control section comprises valves 2 and 4, and a pressure gauge. Valves 2 and 4 are used to finely tune the desired pressure by removing large (valve 4) or small (valve 2) excesses of overdosed gas. The ¼-inch VCR connector of valve 2 contains a 0.5 µm filter to slow the gas flow, which allows

greater control over the amount of gas evacuated. (3) The exhaust and vacuum comprise valves 3, 5 and 6. The exhaust is controlled by valve 3, while valve 5 is directly connected to a Pfeifer HiCube vacuum station, which contains a rotary and a turbo pump. Valve 6 is used to evacuate the gas/solvent from a second sample, while the main experiment is taking place (valve 6 remains closed if either valve 2 and/or 4 are open). (4) The sample section is controlled by valve 1 and is formed by a mixture of quick release connectors and PTFE flexible tubing linking the gas rig to the gas cell. PTFE tubing was chosen due to its high flexibility, which is required to operate on a standard diffractometer. Unfortunately, the PTFE-ferrule (stainless steel) contact cannot hold pressures above 14 bar, which defines the pressure limit of the gas rig-cell set up. Synchrotron beamlines as Diamond Light Source I19, I11 and ESRF ID22 have larger diffractometers with stronger Φ and ω goniometer motors able to operate gas rig-cells using less flexible and more robust tubes of materials such PEEK or stainless steel, allowing larger operating gas pressures.

The pneumatically controlled Swagelok bellows-sealed valves are triggered by house compressed air distributed via an SMC manifold and solenoid actuators. Communication to the manifold is effected through a modular control box. The gas rig is controlled by a windows PC responsible for running the control software. See Supplementary Figure 2 for a block diagram of the control electronics.

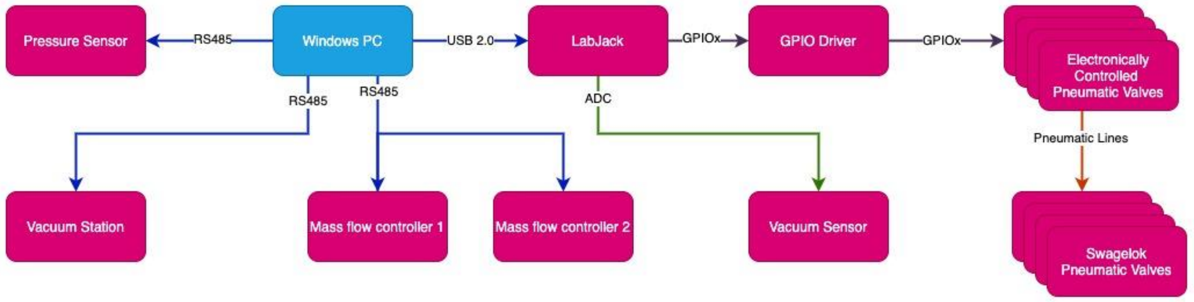

Supplementary Figure 2. Block diagram of the control electronics for the UoM gas rig.

The control software written in Qt 5.10 (C++14) and is available on GitHub https://github.com/MU-Electronics/GasRig. It is cross-platform tested on Windows, MacOS & Linux allowing for easy development and a future move to an embedded system on a chip (SoC), as this would replace the Windows PC & LabJack unit. The GUI was designed using Qt's user interface markup language QML, allowing the GUI to render via the GPU, thereby reducing the load on the CPU, which is important for an embedded SOC. The control element of the software architecture is based around many small-state machines controlling relevant functionality. This allows additional automated experiments to be plugged together with basic C++ development skills.

The state machine *Pressurise* allows the developer to set pressures from 10^{-6} to 100 bar. The accuracy of the end pressure is determined by the largest value, 0.2% of the desired end pressure or 30 mbar. If the pressure is below 1 bar , however, the tolerance is 5 mbar. This accuracy is achieved by using dynamic valve pulse timings dependant on the delta of the previous pressure increase, and the valve timing used to achieve that delta. All control of hardware via the PC is done either by USB or RS485 connections. It was decided to keep the RS485 devices on separate busses to reduce the impact of bus lockup, although this does not include the flow controllers which share a bus. As these controllers are from the same manufacturer and can be isolated from the rig via valve 8, this was deemed less of a risk.

As the gas rig is capable of controlling pressures from $10^{-6} - 100$ bar there are two pressure sensors. The first is a Keller PAA-33X sensor capable of reading pressures from 0.1 – 100 bar. The Keller PAA-

33X pressure gauge does not provide accurate pressures below 0.1 bar, but will not be damaged at such low pressures, and has a maximum overpressure of 200 bar. A Pfeiffer HiCube 80 sensor mounted on top of the vacuum station reads from $10^{-6} - 0.1$ bar.

The LabJack is responsible for controlling the solenoid pneumatic actuators, which then actuate the larger Swagelok bellow valves. The LabJack's general purpose input/output (GPIO) does not have the drive capability required by the actuators, hence a small printed circuit board (PCB) with a ULN2003 transistor array was designed to buffer the signals. The LabJack was also used to read the vacuum pressure value via its analogue-to-digital convertor (ADC).

2.2 Gas cell

Supplementary Figure 3 shows the gas cell developed at the University of Manchester mounted on a standard Huber goniometer head for use on a four-circle goniometer. The gas cell can be divided in 5 different sections (Supplementary Figures 4-9) that could be fabricated in any mechanical workshop.

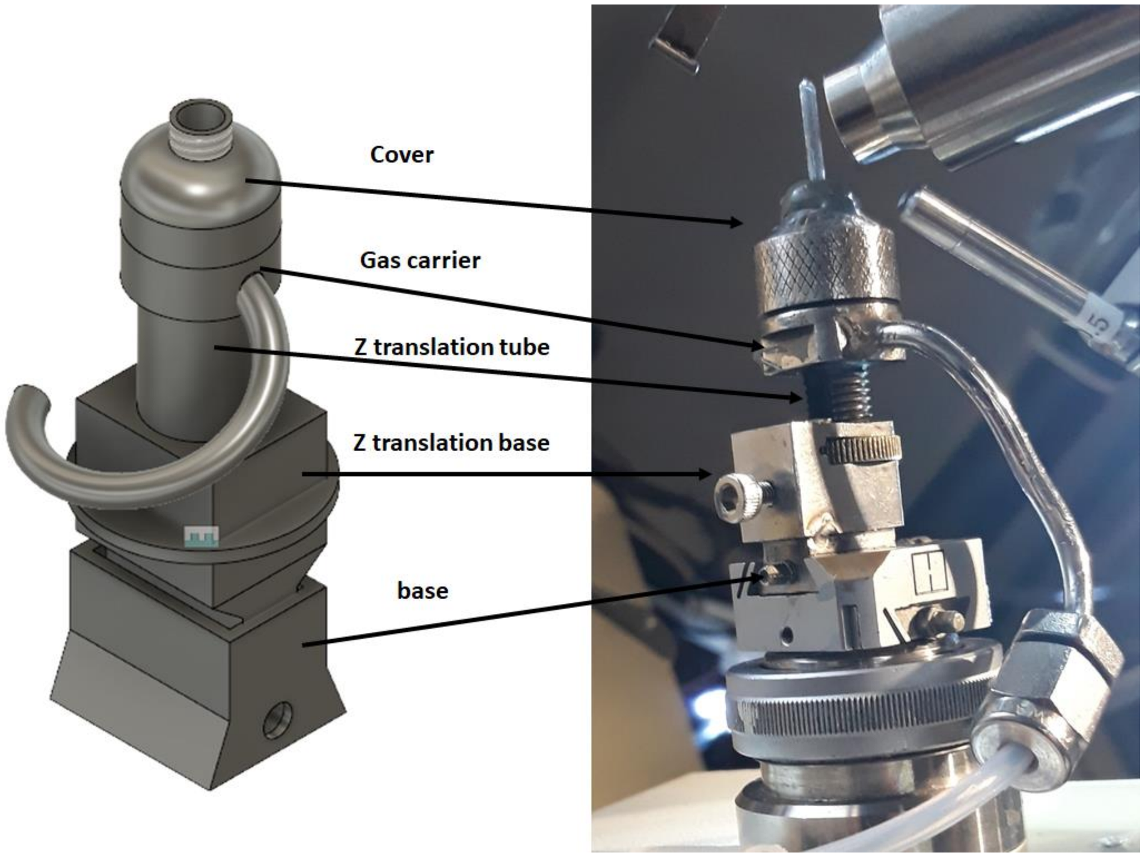

Supplementary Figure 3. Schematic of gas cell (left) and gas cell mounted on goniometer of the diffractometer (right).

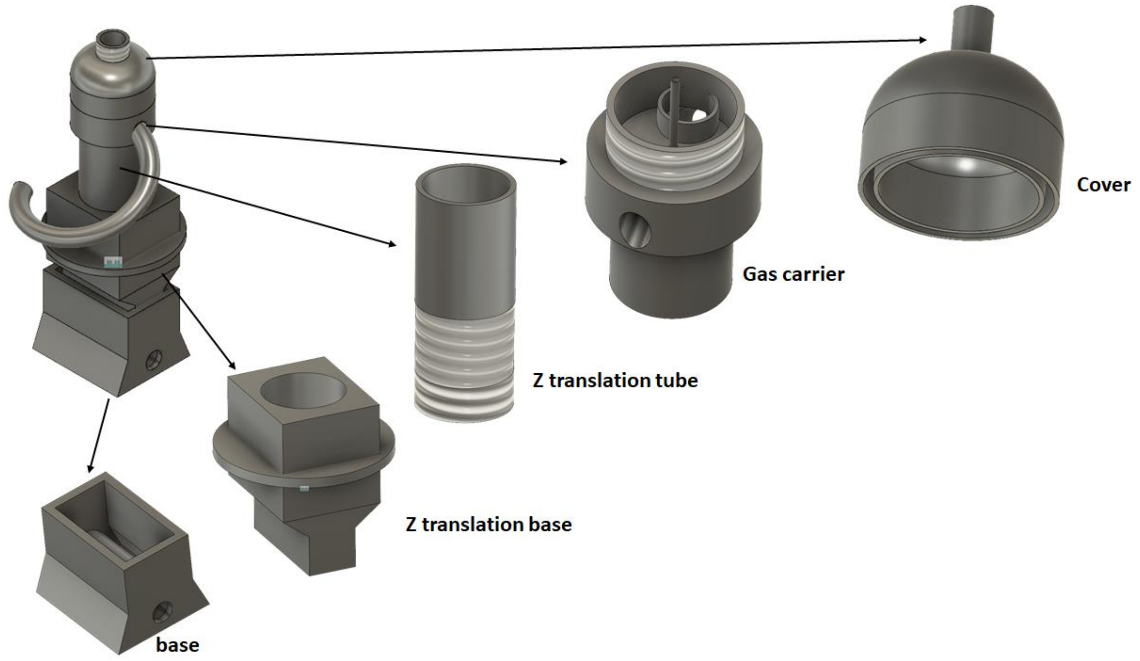

Supplementary Figure 4. Schematic of 5 different sections of the gas.

2.2.1 The base

The base, shown in Supplementary Figure 4, is the link between the gas cell and the Huber goniometer head and replaces the *z*-translation section of the Huber goniometer head (the threaded screw is retained). It should fit the goniometer head *xy*-translation section impeding any unwanted lateral movement.

The rectangular top section of the base should be hollow and 4 cm deep, so that the base of the z-translation base can be attached.

The base contains two interlinked holes with different sizes. The size of the channel should be the same as the larger hole (3.5 mm diameter). The channel must contain a thread which matches the original thread of the threaded screw of the Huber goniometer head.

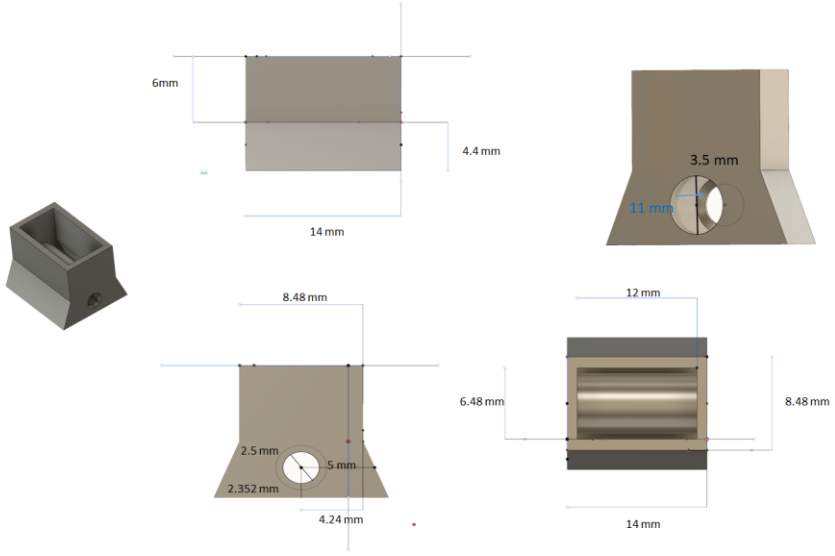

Supplementary Figure 5. Schematic showing the dimensions of the base of the gas cell.

2.2.2 The *z*-translation base

The *z*-translation base is shown in Supplementary Figure 6. The bottom of the *z*-translation base should match the hollow top of the base. The rectangular section at the top of the *z*-translation base contains a cylindrical channel, which will contain the *z*-translation tube. At the top of this channel is placed a threaded washer (inner diameter 8 mm, outer diameter < 11 mm which will be used to control the movement in the *z*-axis of the *z*-translation tube. A screw could be added in the structure in order to fix the position of the *z*-translation tube once the crystal has been centred in the X-ray beam on the diffractometer.

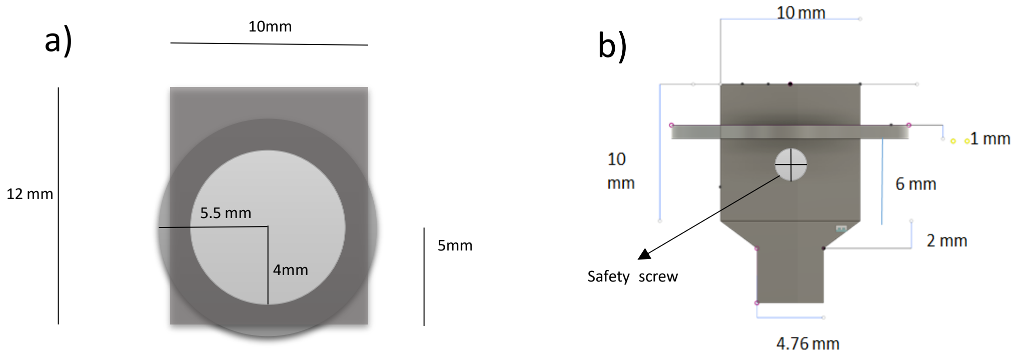

Supplementary Figure 6. Schematic showing the dimensions of the *z*-translation base of the gas cell.

2.2.3 The *z*-translation tube

The *z*-translation tube is shown in Supplementary Figure 7. The aim to the *z*-translation tube is to enable the gas carrier section to move along the *z*-axis. This is needed for centring of the crystal in the X-ray beam. It comprises two main sections:

- The threaded section is solid and fits the channel of the *z*-translation base. The thread has to match the thread of the washer that will control the movement of the tube along the z-axis.

▪ The non-threaded section is hollow, so the gas carrier section can be attached to it.

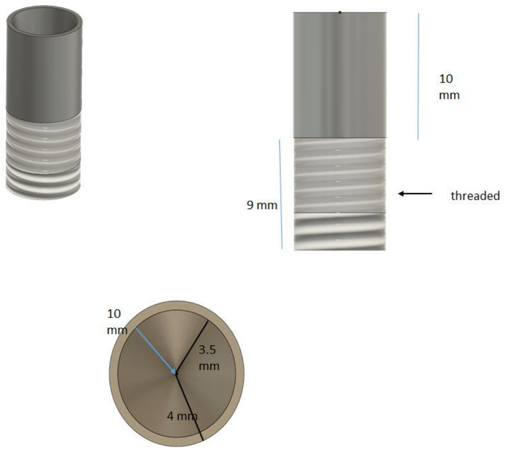

Supplementary Figure 7. Schematic showing the dimensions of the *z*-translation tube of the gas cell.

2.2.4 The gas carrier

Probably this is the most complicated part of the gas cell is the gas carrier section, shown in Supplementary Figure 8. This section acts as a gas chamber before the gas contacts the crystal. The cylindrical base must match the channel dimensions of the *z*-translation tube. Alternatively, the top smooth part of the *z*-translation can be cut and the threaded section can be welded on the base of the gas carrier. Two different versions of this section can be built depending on whether the gas cell is used under static or dynamic pressure. For dynamic pressure experiments the gas carrier should contain a gas inlet hole and an exit hole inside a cylinder. The cylinder around the exit hole is required to allow a better gas transmission to the sample. For static pressure experiments, only the inlet hole is required. At the top of the section a circular thread with an insertion for an O-ring must be built to seal the chamber.

A hollow cylinder must be placed at the exact centre of the top section to introduce the crystal mount (glass fibre or mitogen mount). Ideally, the copper MiTeGen sample holder shown in Supplementary Figure 8 could be used to hold the crystal pin or glass fibre.

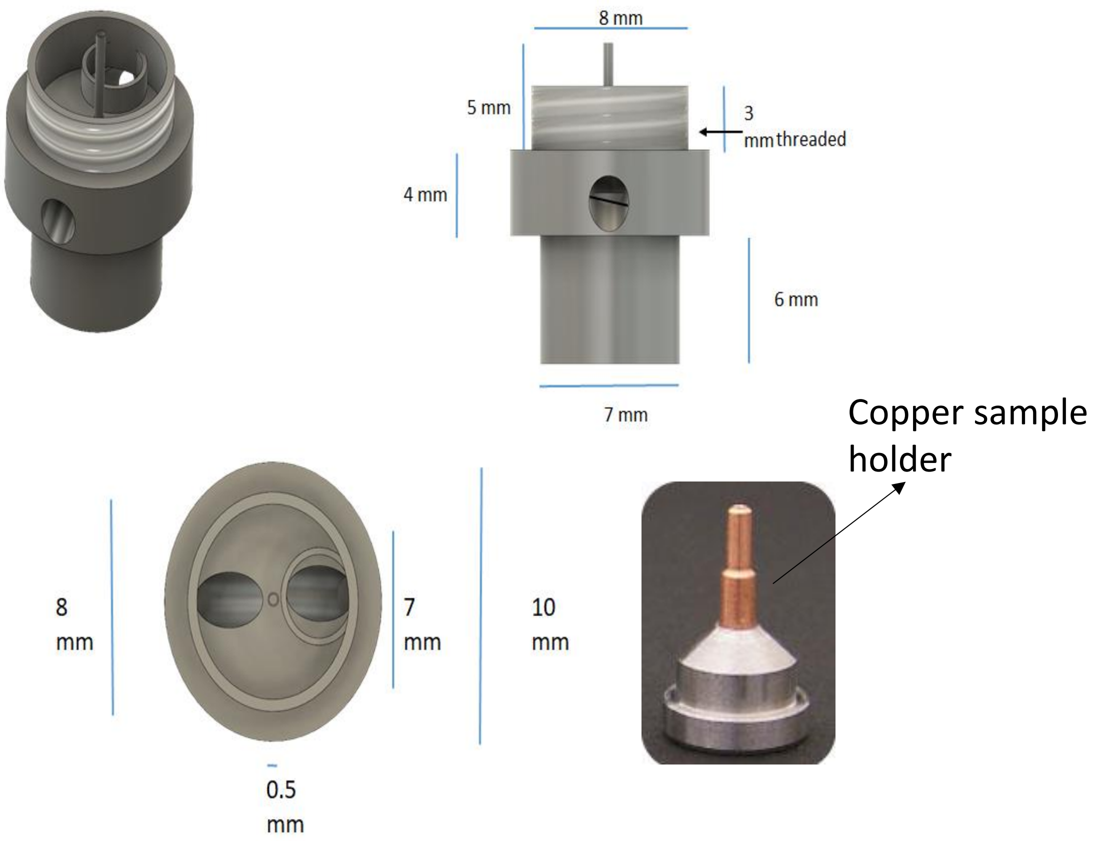

Supplementary Figure 8. Schematic showing the dimensions of the gas carrier section of the gas cell.

2.2.5 The cover

The cover is used to seal the gas carrier section and is shown in Supplementary Figure 9. The interior must be threaded to match the thread of the gas carrier.

At the top of the cover is formed into a hollow cylinder into which will be glued to a 1-3 mm quartz capillary. The diameter of the quartz capillary will determine the attenuation of the beam (larger capillaries – more attenuation), and the difficulty in threading the crystal (and its attached fibre or mitogen mount) through the capillary (smaller capillaries – more difficult).

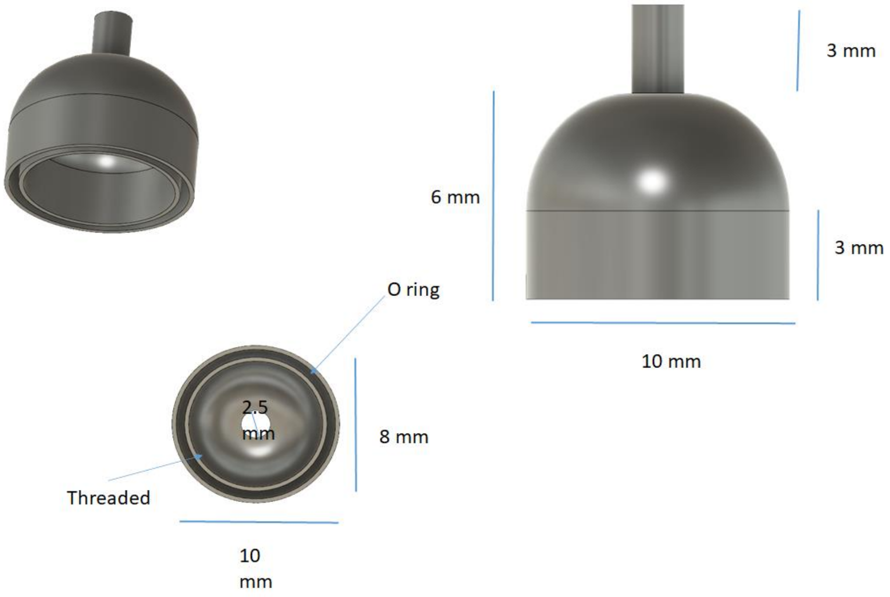

Supplementary Figure 9. Schematic showing the dimensions of the cover of the gas cell.

2.2.6 The pipe

A metal tube is welded to the inlet whole of the gas carrier section. It is recommended that the metal pipe spirals downwards around the gas cell. A male or female connector must be welded at the end of the tube. The direction of the spiral (chirality) should be chosen depending on the preferred φ and ω rotation directions of the goniometer. The location of the gas rig relative to the diffractometer should also be considered before defining the metallic tube direction.

PTFE tubing was selected to connect the gas cell and the gas rig. The nature of this material forming the tubing will depend on the goniometer size and strength of the φ and ω goniometer motors. As noted earlier, stronger and less flexible tubing (e.g. stainless steel) could be used in conjunction with stringer motors than are in place on typical laboratory X-ray diffractometers.

3. Single-crystal X-ray diffraction

3.1 Data collection, structure solution and refinement and crystal data

3.1.1 Coordination polymers 1−5 and 1−5-ROH (ROH = MeOH or EtOH)

Using crystals directly synthesized from solution, X-ray data were collected at 298 K or 150 K using Mo-Kα radiation for compound **1-MeOH** (CCDC 903747), **1** (polymorph $\mathbf{1_A}^{\mathbf{HT}}$ (CCDC 903751)), $\mathbf{1_A}^{\mathbf{LT}}$ (CCDC 1044595), $\mathbf{1_B}^{\mathbf{HT}}$ (CCDC 1044597) and $\mathbf{1_B}^{\mathbf{LT}}$ (CCDC 1044596), **2-EtOH** (CCDC 654182), **2** (polymorph $\mathbf{2}^{\mathbf{LT}}$ (CCDC 654183)**)**, **3-EtOH**, **3** (polymorphs $\mathbf{3}^{\mathbf{HT}}$ and $\mathbf{3}^{\mathbf{LT}}$), **4-MeOH**, **4**, **5-MeOH** and **5** on a Bruker SMART APEX II diffractometer equipped with an Oxford Cryosystems nitrogen flow gas system.

3.1.2 Coordination polymers 1−5 during CO_2 gas uptake

CO_2 gas uptake in coordination polymer 1. X-ray diffraction data were collected on a crystal of **1** at beamline I19, Diamond Light Source (λ = 0.6889(2) Å), using the gas rig and gas cell set up provided by the beamline.[S2]

1_A^{HT} (RT, 2 bar) → 1_A^{CO2} (253 K, 10 bar) → 1_A^{CO2} (232 K, 10 bar). A partial data set was collected at room temperature and under vacuum confirming the structure of **1_A^{HT}** (high-temperature polymorph A)[S3] by unit cell determination. The CO_2 pressure was increased to 2 bar and a full data set was collected, confirming the presence of coordination polymer **1_A^{HT}**. The CO_2 pressure was then increased to 10 bar and the temperature decreased to 253 K and 232 K. Full data sets were collected at each temperature, documenting an increase in the gas adsorbed in the resultant **1_A^{CO2}** structure consistent with the increased relative pressure (P/P_0) of CO_2 at the reduced temperatures.

CO_2 gas uptake in coordination polymers 2-5. Data were collected for crystals of **2-5** on a Rigaku Synergy FR-X rotating anode diffractometer (Mo Kα = 0.71073 Å) at University of Manchester using the gas rig and gas cell described in Section 2.

2^{HT} (298 K, vacuum) →2^{HT} (273 K, 10 bar) → 2^{HT} (240 K, 10 bar) → 2^{CO2} (215 K, 10 bar) → 2^{CO2} (200 K, 10 bar) → 2^{HT} (298 K, vacuum). A full X-ray data set on a crystal of coordination polymer **2** was collected at 298 K under vacuum, confirming the presence of coordination polymer **2^{HT}** (high-temperature polymorph).[S4] Then, the CO_2 pressure was increased to 10 bar and a full data set was collected at 273 K and 240 K. No structural change in **2^{HT}** was observed. The temperature was further decreased to 215 K and 200 K and full data sets were collected at 10 bar CO_2 pressure, confirming the presence of new polymorph **2^{CO2}** at both temperatures. Then, the temperature was raised to 298 K and a full data set was collected under vacuum confirming the presence of **2^{HT}** and the reversibility of the process.

3-EtOH (298 K, air) →3^{HT} (343 K, vacuum) → 3^{HT}(298 K, 10 bar) → 3^{HT}(250 K, 10 bar) → 3_A^{CO2} (230 K, 10 bar) → 3_A^{CO2} (215 K, 10 bar) → 3_B^{CO2} (200 K, 10 bar) → 3^{HT} (298 K, 1 bar). A partial data set was collected on **3-EtOH** at 298 K, confirming the presence of **3-EtOH** through unit cell measurement. Then, the crystal was heated at 434 K under vacuum for 30 minutes and a data set was collected confirming the release of the coordinated ethanol molecules and the formation of **3^{HT}**, the high-temperature polymorph of the alcohol-free coordination polymer. The CO_2 pressure in the gas cell was increased to 10 bar and the temperature was decreased to 298 and 250 K, and full data sets were collected after each step, confirming the presence of **3^{HT}**. Then, the temperature was decreased to 230 and 215 K and full data sets were collected under 10 bar CO_2 pressure, confirming the presence of CO_2 guest containing coordination polymer **3_A^{CO2}**. The temperature was set to 200 K and a partial data set was collected under 10 bar CO_2, confirming the formation of a new polymorph of the CO_2-containing coordination polymer, **3_B^{CO2}**. Finally, the temperature was raised to 298 K and a full data set was collected under 1 bar CO_2 pressure, confirming the presence of **3^{HT}** and the reversibility of the process.

4(273 K, vacuum) →4(273 K, 10 bar) → 4^{CO2}(240 K, 10 bar) → 4^{CO2} (230 K, 10 bar) → 4^{CO2} (215 K, 10 bar) A full data set on a crystal of coordination polymer **4** was collected at 273 K under vacuum, confirming the presence of coordination polymer **4** after removal of MeOH. Then, the CO_2 pressure was increased to 10 bar and a full data set was collected at 273 K, with no substantial structural change in **4** observed. The temperature was further decreased sequentially to 240, 230 and 215 K and a full data set was collected under 10 bar CO_2 pressure at each temperature, confirming the presence of a new CO_2-containing coordination polymer **4^{CO2}**. A measurement after return to ambient pressure and temperature was not possible as the crystal became detached in the gas cell after the 215 K measurement.

4(273 K, vacuum) →4^{CO2}(200 K, 10 bar). A full data set on a second crystal of coordination polymer **4** was collected at 273 K under vacuum, confirming the presence of coordination polymer **4**. Then, the CO_2 pressure was increased to 10 bar and the temperature decreased to 200 K, and a full data set was collected, confirming the uptake of CO_2 and the formation of coordination polymer $\mathbf{4^{CO2}}$.

5(298 K, vacuum) → 5 (295 K, 10 bar) → 5 (250 K, 10 bar) → 5 (230 K, 10 bar) → $\mathbf{5^{CO2}}$ (200 K, 10 bar). A full data set on a crystal of coordination polymer **5** was collected at 298 K under vacuum, confirming the presence of coordination polymer **5** after loss of MeOH and CH_2Cl_2. Then, the CO_2 pressure was increased to 10 bar and a full data set was collected sequentially at temperatures of 295, 250 and 230 K with no substantial change in the structure of **5** observed. The temperature was further decreased to 200 K and a full data set was collected under 10 bar CO_2 pressure, confirming the presence of a new CO_2-containing coordination polymer $\mathbf{5^{CO2}}$.

Crystal structure determinations and refinements. X-Ray data were processed and reduced using APEX2 or CrysAlisPro programs and corrected for absorption using empirical methods (SADABS[S5] or Scale3 Abspack) based upon symmetry-equivalent reflections combined with measurements at different azimuthal angles. All crystal structures were solved and refined against all F^2 values using OLEX2[S6] and/or SHELX.[S7] A summary of the data collection and structure refinement information is provided in Supplementary Tables 1-11. Displacement ellipsoid plots for all new crystal structures are presented in Supplementary Table 19 (Section 7). Non-hydrogen atoms were refined anisotropically where possible, whereas hydrogen atoms were placed in calculated positions, refined using idealized geometries (riding model) and assigned fixed isotropic displacement parameters. In many of the structure determinations, fluoroalkyl chains are described as disordered over two orientations with carbon and fluorine atoms modelled using isotropic displacement parameters. Distance restraints (SAME, SADI and DFIX commands in SHELX) were applied to C−F and C−C bonds. Atomic displacement parameters of atoms in the perfluoroalkyl chains were restrained using a rigid-body approach by applying SHELX RIGU commands and restrained to be similar in magnitudes using SHELX SIMU commands. CO_2 molecules were modelled as rigid bodies with atomic position and occupancy of the rigid body refined. Isotropic displacement parameters for atoms of the CO_2 molecules were constrained to $U_{iso} = 0.2$ Å^2.

Data collected for compound **1** under CO_2 atmospheres resulted in a low completeness (~91 %) as the strongest reflections collected were treated as detector overloads and omitted from refinements. Data collected for compound $\mathbf{2^{CO2}}$ (200 K, 10 bar CO_2), $\mathbf{3_B^{CO2}}$ (200 K, 10 bar CO_2) and $\mathbf{4^{CO2}}$ (200 K, 10 bar CO_2) had lower resolutions than other data ($d_{min} \approx 1$ Å). These crystal data are summarised in Supplementary Tables 1-9

Crystallographic data have been deposited in the CCDC (CCDC 654182, 654183, 903747, 903751, 1044595, 1044596, 1044597, 2329006-2329039). See Supplementary Section 7 for details.

Supplementary Table 1. Crystal data for $\mathbf{1_A^{HT}}$ **(298 K, 2 bar)**, $\mathbf{1^{CO2}}$ **(253 K, 10 bar)** and $\mathbf{1^{CO2}}$ **(232 K, 10 bar)**

Identification code	**1_A^{HT} (298 K, 2 bar)**	**1^{CO2} (253 K, 10 bar)**	**1^{CO2} (232 K, 10 bar)**
Empirical formula	$C_{40}H_{36}Ag_4F_{28}N_6O_8$	$C_{41.56}H_{36}Ag_4F_{28}N_6O_{11.12}$	$C_{41.91}H_{36}Ag_4F_{28}N_6O_{11.81}$
Formula weight	1692.23	1760.80	1776.06
Temperature/K	298	253	232
Crystal system	triclinic	triclinic	triclinic
Space group	P-1	P-1	P-1
a/Å	8.5428(5)	8.4972(3)	8.4633(5)
b/Å	12.9026(13)	16.4885(7)	16.5444(6)
c/Å	14.8894(10)	22.8257(9)	22.7144(7)
α/°	112.854(8)	76.687(3)	77.118(3)
β/°	90.217(5)	79.916(3)	79.657(4)
γ/°	108.808(7)	77.806(3)	78.599(4)
Volume/Å^3	1415.8(2)	3014.8(2)	3008.6(3)
Z	1	2	2
ρ_{calc} (g/cm^3)	1.985	1.940	1.961
μ/mm^{-1}	1.396	1.318	1.323
F(000)	822.0	1713.0	1728.0
Crystal size/mm^3	0.2 × 0.1 × 0.09	0.2 × 0.1 × 0.09	0.2 × 0.1 × 0.09
Radiation (λ/ Å)	synchrotron (λ = 0.6889)	synchrotron (λ = 0.6889)	synchrotron (λ = 0.6889)
2Θ range for data collection/°	4.964 to 49.036	3.356 to 49.038	3.958 to 49.036
Index ranges	-10 ≤ h ≤ 8, -15 ≤ k ≤ 15, -17 ≤ l ≤ 17	-10 ≤ h ≤ 8, -19 ≤ k ≤ 19, -27 ≤ l ≤ 27	-10 ≤ h ≤ 9, -19 ≤ k ≤ 19, -27 ≤ l ≤ 27
Reflections collected	17349	36903	37498
Independent reflections	4744 [R_{int} = 0.0609, R_{sigma} = 0.0479]	10034 [R_{int} = 0.1735, R_{sigma} = 0.2204]	10053 [R_{int} = 0.0764, R_{sigma} = 0.0653]
Data/restraints/parameters	4744/683/576	10034/741/1115	10053/883/1112
Goodness-of-fit on F^2	1.074	1.170	1.035
Final R indices [I ≥ 2σ(I)]	R_1 = 0.0975, wR_2 = 0.2697	R_1 = 0.1303, wR_2 = 0.3114	R_1 = 0.1016, wR_2 = 0.2698
Final R indices [all data]	R_1 = 0.1161, wR_2 = 0.2920	R_1 = 0.1640, wR_2 = 0.3378	R_1 = 0.1427, wR_2 = 0.3156
Largest diff. peak/hole / e Å^{-3}	1.11/-0.95	1.21/-1.19	1.00/-1.03

Supplementary Table 2. Crystal data for $\mathbf{2^{HT}}$**(298 K, vacuum)**, $\mathbf{2^{HT}}$**(273 K, 10 bar)** and $\mathbf{2^{HT}}$**(240 K, 10 bar)**

Identification code	**2^{HT}(298 K, vacuum)**	**2^{HT}(273 K, 10 bar)**	**2^{HT}(240 K, 10 bar)**
Empirical formula	$C_{44}H_{36}Ag_4F_{36}N_6O_8$	$C_{44}H_{36}Ag_4F_{36}N_6O_8$	$C_{44}H_{36}Ag_4F_{36}N_6O_8$
Formula weight	1892.27	1892.27	1892.27
Temperature/K	293.62(10)	272.94(11)	240.00(10)
Crystal system	triclinic	triclinic	triclinic
Space group	P-1	P-1	P-1
a/Å	8.6651(6)	8.6288(6)	8.7078(8)
b/Å	14.0420(9)	13.9877(9)	14.1117(16)
c/Å	14.9335(11)	14.8614(10)	14.9915(13)
α/°	113.375(7)	113.486(6)	113.565(10)
β/°	91.090(6)	91.271(5)	91.549(7)
γ/°	106.235(6)	106.270(6)	106.321(9)
Volume/Å^3	1583.9(2)	1560.89(19)	1600.0(3)
Z	1	1	1
ρ_{calc}g/cm^3	1.984	2.013	1.964

μ/mm^{-1}	1.376	1.397	1.362
F(000)	918.0	918.0	918.0
Crystal size/mm^3	0.3 × 0.2 × 0.2	0.3 × 0.2 × 0.2	0.3 × 0.2 × 0.2
Radiation	MoKα (λ = 0.71073)	MoKα (λ = 0.71073)	MoKα (λ = 0.71073)
2Θ range for data collection/°	3.326 to 50.698	3.346 to 50.698	3.322 to 50.7
Index ranges	-10 ≤ h ≤ 10, -16 ≤ k ≤ 16, -17 ≤ l ≤ 17	-10 ≤ h ≤ 10, -16 ≤ k ≤ 16, -17 ≤ l ≤ 17	-10 ≤ h ≤ 10, -17 ≤ k ≤ 17, -18 ≤ l ≤ 18
Reflections collected	13995	14397	14040
Independent reflections	5724 [R_{int} = 0.0311, R_{sigma} = 0.0390]	5667 [R_{int} = 0.0231, R_{sigma} = 0.0288]	5795 [R_{int} = 0.0299, R_{sigma} = 0.0367]
Data/restraints/parameters	5724/633/684	5667/633/684	5795/639/684
Goodness-of-fit on F^2	1.030	1.029	1.046
Final R indexes [I>=2σ (I)]	R_1 = 0.0895, wR_2 = 0.2517	R_1 = 0.0683, wR_2 = 0.1966	R_1 = 0.0795, wR_2 = 0.2163
Final R indexes [all data]	R_1 = 0.1428, wR_2 = 0.3059	R_1 = 0.1013, wR_2 = 0.2393	R_1 = 0.1251, wR_2 = 0.2584
Largest diff. peak/hole / e Å^{-3}	1.07/-0.87	0.87/-0.88	0.89/-0.85

Supplementary Table 3. Crystal data for **2^{CO2} (215 K, 10 bar)**, **2^{CO2} (200 K, 10 bar)** and **2^{HT}(298 K, vacuum)_2**

Identification code	**2^{CO2} (215 K, 10 bar)**	**2^{CO2} (200 K, 10 bar)**	**2^{HT}(298 K, vacuum)_2**
Empirical formula	$C_{45.99}H_{36}Ag_4F_{36}N_6O_{11.98}$	$C_{46.32}H_{36}Ag_4F_{36}N_6O_{12.64}$	$C_{44}H_{36}Ag_4F_{36}N_6O_8$
Formula weight	1979.85	1994.37	1892.27
Temperature/K	215.00(10)	200.00(10)	298.00(10)
Crystal system	triclinic	triclinic	triclinic
Space group	P-1	P-1	P-1
a/Å	8.5006(9)	8.4943(9)	8.6684(9)
b/Å	14.6828(14)	14.6540(14)	14.0722(19)
c/Å	14.9263(16)	14.8956(16)	14.9999(18)
α/°	67.254(10)	67.125(9)	113.386(12)
β/°	84.173(9)	84.210(9)	91.082(9)
γ/°	74.504(9)	74.726(9)	106.182(11)
Volume/Å^3	1655.6(3)	1648.0(3)	1595.4(4)
Z	1	1	1
ρ_{calc}g/cm^3	1.986	2.010	1.970
μ/mm^{-1}	1.326	1.334	1.366
F(000)	962.0	969.0	918.0
Crystal size/mm^3	0.3 × 0.2 × 0.2	0.3 × 0.2 × 0.2	0.3 × 0.2 × 0.2
Radiation	MoKα (λ = 0.71073)	MoKα (λ = 0.71073)	MoKα (λ = 0.71073)
2Θ range for data collection/°	3.106 to 50.696	3.11 to 50.698	3.318 to 50.696
Index ranges	-10 ≤ h ≤ 10, -17 ≤ k ≤ 17, -17 ≤ l ≤ 17	-10 ≤ h ≤ 10, -17 ≤ k ≤ 17, -17 ≤ l ≤ 17	-10 ≤ h ≤ 10, -16 ≤ k ≤ 16, -18 ≤ l ≤ 18
Reflections collected	14114	14206	13943
Independent reflections	5981 [R_{int} = 0.0302, R_{sigma} = 0.0432]	5984 [R_{int} = 0.0322, R_{sigma} = 0.0388]	5774 [R_{int} = 0.0343, R_{sigma} = 0.0493]
Data/restraints/parameters	5981/779/706	5984/779/709	5774/639/684
Goodness-of-fit on F^2	1.042	1.038	1.023
Final R indexes [I > 2σ(I)]	R_1 = 0.0792, wR_2 = 0.2048	R_1 = 0.0819, wR_2 = 0.2192	R_1 = 0.0897, wR_2 = 0.2474
Final R indexes [all data]	R_1 = 0.1274, wR_2 = 0.2591	R_1 = 0.1094, wR_2 = 0.2524	R_1 = 0.1568, wR_2 = 0.3236
Largest diff. peak/hole / e Å^{-3}	1.67/-1.14	1.70/-1.09	1.06/-0.83

Supplementary Table 4. Crystal data for **3-EtOH**, **3^{LT}** and **3^{HT}(343 K, vacuum)**

Identification code	**3-EtOH**	**3^{LT}**	**3^{HT}(343 K, vacuum)**
Empirical formula	$C_{52}H_{48}Ag_4F_{44}N_6O_{10}$	$C_{48}H_{36}Ag_4F_{44}N_6O_8$	$C_{48}H_{36}Ag_4F_{44}N_6O_8$
Formula weight	2184.44	2092.31	2092.31
Temperature/K	100.00	100.15	343.01(10)
Crystal system	triclinic	triclinic	triclinic
Space group	P-1	P-1	P-1
a/Å	15.1647(5)	16.3790(10)	8.6818(6)
b/Å	15.6672(5)	19.8947(13)	14.9986(12)
c/Å	17.6814(6)	27.1182(17)	15.1451(13)
α/°	70.770(2)	74.700(3)	108.618(8)
β/°	76.685(2)	83.910(4)	105.898(7)
γ/°	65.454(2)	76.128(3)	90.325(6)
Volume/$Å^3$	3586.6(2)	8266.2(9)	1788.0(3)
Z	2	5	1
$\rho_{calc}g/cm^3$	2.023	2.102	1.943
μ/mm^{-1}	1.248	1.346	1.245
F(000)	2132.0	5070.0	1014.0
Crystal size/mm^3	0.51 × 0.21 × 0.05	0.42 × 0.39 × 0.14	0.3 × 0.3 × 0.3
Radiation	MoKα (λ = 0.71073)	MoKα (λ = 0.71073)	MoKα (λ = 0.71073)
2Θ range for data collection/°	3.31 to 55.052	2.176 to 50.7	2.966 to 50.69
Index ranges	-19 ≤ h ≤ 19, -20 ≤ k ≤ 20, -22 ≤ l ≤ 22	-19 ≤ h ≤ 15, -23 ≤ k ≤ 23, -32 ≤ l ≤ 32	-10 ≤ h ≤ 10, -18 ≤ k ≤ 18, -18 ≤ l ≤ 18
Reflections collected	80667	112577	17079
Independent reflections	16433 [R_{int} = 0.0275, R_{sigma} = 0.0219]	29987 [R_{int} = 0.0793, R_{sigma} = 0.1120]	6532 [R_{int} = 0.0241, R_{sigma} = 0.0384]
Data/restraints/parameters	16433/4/1065	29987/39/2495	6532/984/792
Goodness-of-fit on F^2	1.013	1.017	1.150
Final R indexes [I > 2σ(I)]	R_1 = 0.0288, wR_2 = 0.0781	R_1 = 0.0641, wR_2 = 0.1071	R_1 = 0.0910, wR_2 = 0.2782
Final R indexes [all data]	R_1 = 0.0389, wR_2 = 0.0871	R_1 = 0.1610, wR_2 = 0.1407	R_1 = 0.1536, wR_2 = 0.3427
Largest diff. peak/hole / e $Å^{-3}$	0.81/-0.74	1.15/-0.90	0.90/-0.85

Supplementary Table 5. Crystal data for **3^{HT}** (298 K, 10 bar CO_2), **3^{HT}** (250 K, 10 bar CO_2) and **3_A^{CO2}** (230 K, 10 bar CO_2)

Identification code	**3^{HT}(298 K, 10 bar)**	**3^{HT}(250 K, 10 bar)**	**3_A^{CO2} (230 K, 10 bar)**
Empirical formula	$C_{48}H_{36}Ag_4F_{44}N_6O_8$	$C_{48}H_{36}Ag_4F_{44}N_6O_8$	$C_{49.05}H_{36}Ag_4F_{44}N_6O_{10.09}$
Formula weight	2092.31	2092.31	2138.30
Temperature/K	298.00(10)	250.01(10)	230.00(10)
Crystal system	triclinic	triclinic	triclinic
Space group	P-1	P-1	P-1
a/Å	8.6395(6)	8.5880(5)	8.5794(6)
b/Å	14.9070(10)	14.7837(12)	16.7345(9)
c/Å	15.1845(11)	15.4028(11)	25.8830(12)
α/°	109.141(7)	111.907(7)	85.166(4)
β/°	105.676(6)	103.937(6)	82.634(5)
γ/°	90.537(5)	90.746(6)	76.815(5)
Volume/$Å^3$	1768.4(2)	1749.4(2)	3582.4(4)
Z	1	1	2

ρ_{calc}g/cm^3	1.965	1.986	1.982
μ/mm^{-1}	1.259	1.272	1.247
F(000)	1014.0	1014.0	2074.0
Crystal size/mm^3	0.3 × 0.3 × 0.3	0.3 × 0.3 × 0.3	0.3 × 0.3 × 0.3
Radiation	MoKα (λ = 0.71073)	MoKα (λ = 0.71073)	MoKα (λ = 0.71073)
2Θ range for data collection/°	2.966 to 50.694	2.956 to 50.696	3.178 to 50.7
Index ranges	-10 ≤ h ≤ 10, -17 ≤ k ≤ 17, -18 ≤ l ≤ 18	-10 ≤ h ≤ 10, -17 ≤ k ≤ 17, -18 ≤ l ≤ 18	-10 ≤ h ≤ 10, -20 ≤ k ≤ 20, -31 ≤ l ≤ 31
Reflections collected	16863	16701	34592
Independent reflections	6454 [R_{int} = 0.0257, R_{sigma} = 0.0426]	6392 [R_{int} = 0.0300, R_{sigma} = 0.0418]	13077 [R_{int} = 0.0327, R_{sigma} = 0.0511]
Data/restraints/parameters	6454/977/792	6392/992/792	13077/1754/1624
Goodness-of-fit on F^2	1.152	1.019	1.018
Final R indexes [I > 2σ(I)]	R_1 = 0.0944, wR_2 = 0.2837	R_1 = 0.0755, wR_2 = 0.2148	R_1 = 0.0814, wR_2 = 0.2223
Final R indexes [all data]	R_1 = 0.1533, wR_2 = 0.3397	R_1 = 0.1175, wR_2 = 0.2612	R_1 = 0.1274, wR_2 = 0.2575
Largest diff. peak/hole / e Å^{-3}	1.13/-0.85	1.20/-0.82	1.14/-1.07

Supplementary Table 6. Crystal data for $\mathbf{3_A^{CO2}}$ (215 K, 10 bar CO_2), $\mathbf{3_B^{CO2}}$ (200 K, 10 bar CO_2) and $\mathbf{3^{HT}}$(298 K, 1 bar CO_2)

Identification code	**3_A^{CO2} (215 K, 10 bar)**	**3_B^{CO2} (200 K, 10 bar)**	**3^{HT}(298 K, 1bar)**
Empirical formula	$C_{49.28}H_{36}Ag_4F_{44}N_6O_{10.57}$	$C_{51.23}H_{36}Ag_4F_{44}N_6O_{14.46}$	$C_{48}H_{36}Ag_4F_{44}N_6O_8$
Formula weight	2148.72	2234.38	2092.31
Temperature/K	215.00(10)	199.97(10)	298.05(14)
Crystal system	triclinic	triclinic	triclinic
Space group	P-1	P-1	P-1
a/Å	8.5914(6)	15.7442(16)	8.6547(7)
b/Å	16.8159(11)	15.9795(14)	14.9510(12)
c/Å	25.9859(12)	16.3094(16)	15.1393(15)
α/°	85.165(5)	90.980(7)	108.789(8)
β/°	82.758(5)	103.568(9)	105.814(8)
γ/°	76.706(6)	103.389(8)	90.531(7)
Volume/Å^3	3618.6(4)	3869.2(7)	1774.2(3)
Z	2	2	1
ρ_{calc}g/cm^3	1.972	1.918	1.958
μ/mm^{-1}	1.236	1.163	1.255
F(000)	2084.0	2170.0	1014.0
Crystal size/mm^3	0.3 × 0.3 × 0.3	0.3 × 0.3 × 0.3	0.3 × 0.3 × 0.3
Radiation	Mo Kα (λ = 0.71073)	MoKα (λ = 0.71073)	MoKα (λ = 0.71073)
2Θ range for data collection/°	3.164 to 50.7	3.816 to 41.624	2.97 to 50.698
Index ranges	-10 ≤ h ≤ 10, -20 ≤ k ≤ 20, -31 ≤ l ≤ 31	-15 ≤ h ≤ 15, -14 ≤ k ≤ 15, -16 ≤ l ≤ 16	-10 ≤ h ≤ 10, -17 ≤ k ≤ 18, -18 ≤ l ≤ 18
Reflections collected	34352	8181	16714
Independent reflections	13193 [R_{int} = 0.0491, R_{sigma} = 0.0729]	6444 [R_{int} = 0.0218, R_{sigma} = 0.0644]	6477 [R_{int} = 0.0360, R_{sigma} = 0.0585]
Data/restraints/parameters	13193/1815/1622	6444/320/790	6477/978/792
Goodness-of-fit on F^2	1.081	1.045	1.179
Final R indexes [I>=2σ (I)]	R_1 = 0.0853, wR_2 = 0.2263	R_1 = 0.0876, wR_2 = 0.2339	R_1 = 0.1043, wR_2 = 0.2999
Final R indexes [all data]	R_1 = 0.1394, wR_2 = 0.2592	R_1 = 0.1391, wR_2 = 0.2779	R_1 = 0.1839, wR_2 = 0.3714

Largest diff. peak/hole / e Å^{-3}	0.85/-0.85	1.02/-0.62	1.29/-0.86

Supplementary Table 7. Crystal data for **4-MeOH**, **4** (273 K, vacuum) and **4** (273 K, 10 bar CO_2)

Identification code	**4-MeOH**	**4 (273 K, vacuum)**	**4 (273 K, 10 bar)**
Empirical formula	$C_{54}H_{44}Ag_4F_{52}N_6O_{10}$	$C_{52}H_{36}Ag_4F_{52}N_6O_8$	$C_{52}H_{36}Ag_4F_{52}N_6O_8$
Formula weight	2356.43	2292.35	2292.35
Temperature/K	100.15	273.00(10)	273.01(10)
Crystal system	triclinic	triclinic	triclinic
Space group	P-1	P-1	P-1
a/Å	8.8229(7)	8.6052(4)	8.5653(8)
b/Å	15.1912(12)	14.6635(8)	14.6070(13)
c/Å	16.3445(13)	16.8351(8)	16.7926(13)
α/°	106.536(4)	111.876(5)	111.975(8)
β/°	100.472(3)	103.701(4)	103.649(7)
γ/°	103.925(3)	91.235(4)	91.201(7)
Volume/Å^3	1963.2(3)	1900.98(18)	1879.3(3)
Z	1	1	1
ρ_{calc}g/cm^3	1.993	2.002	2.025
μ/mm^{-1}	1.162	1.195	1.209
F(000)	1146.0	1110.0	1110.0
Crystal size/mm^3	0.55 × 0.4 × 0.37	0.5 × 0.3 × 0.25	0.5 × 0.3 × 0.25
Radiation	MoKα (λ = 0.71073)	MoKα (λ = 0.71073)	MoKα (λ = 0.71073)
2Θ range for data collection/°	2.698 to 50.7	3.016 to 50.692	3.028 to 50.7
Index ranges	-10 ≤ h ≤ 10, -18 ≤ k ≤ 18, -19 ≤ l ≤ 19	-10 ≤ h ≤ 10, -17 ≤ k ≤ 17, -20 ≤ l ≤ 20	-9 ≤ h ≤ 10, -17 ≤ k ≤ 17, -20 ≤ l ≤ 20
Reflections collected	25951	13238	12726
Independent reflections	7191 [R_{int} = 0.0650, R_{sigma} = 0.0567]	6638 [R_{int} = 0.0216, R_{sigma} = 0.0305]	6534 [R_{int} = 0.0309, R_{sigma} = 0.0450]
Data/restraints/parameters	7191/123/541	6638/404/640	6534/406/640
Goodness-of-fit on F^2	1.060	1.009	1.041
Final R indexes [I > 2σ(I)]	R_1 = 0.0556, wR_2 = 0.1435	R_1 = 0.0738, wR_2 = 0.2110	R_1 = 0.0869, wR_2 = 0.2346
Final R indexes [all data]	R_1 = 0.0620, wR_2 = 0.1484	R_1 = 0.1013, wR_2 = 0.2489	R_1 = 0.1299, wR_2 = 0.2735
Largest diff. peak/hole / e Å^{-3}	1.27/-1.57	0.96/-0.67	0.91/-0.59

Supplementary Table 8. Crystal data for **4^{CO2}** (240 K, 10 bar CO_2), **4^{CO2}** (230 K, 10 bar CO_2) and **4^{CO2}** (215 K, 10 bar CO_2)

Identification code	**4^{CO2} (240 K, 10 bar)**	**4^{CO2} (230 K, 10 bar)**	**4^{CO2} (215 K, 10 bar)**
Empirical formula	$C_{52.52}H_{36}Ag_4F_{52}N_6O_{9.04}$	$C_{52.5}H_{36}Ag_4F_{52}N_6O_9$	$C_{52.63}H_{36}Ag_4F_{52}N_6O_{9.26}$
Formula weight	2315.31	2314.35	2319.99
Temperature/K	240.00(10)	230.00(10)	214.99(10)
Crystal system	triclinic	triclinic	triclinic
Space group	P-1	P-1	P-1
a/Å	8.6188(8)	8.6062(8)	8.6042(6)
b/Å	16.9261(11)	16.9194(11)	16.9456(9)
c/Å	27.2565(19)	27.1917(19)	27.1711(17)
α/°	95.774(5)	95.793(6)	95.650(5)
β/°	97.972(6)	97.934(6)	97.756(6)
γ/°	103.082(7)	103.075(7)	103.063(5)

Volume/Å^3	3799.5(5)	3783.8(5)	3789.5(4)
Z	2	2	2
ρ_{calc}g/cm^3	2.024	2.031	2.033
μ/mm^{-1}	1.198	1.203	1.201
F(000)	2243.0	2242.0	2248.0
Crystal size/mm^3	0.5 × 0.3 × 0.25	0.5 × 0.3 × 0.25	0.5 × 0.3 × 0.25
Radiation	MoKα (λ = 0.71073)	MoKα (λ = 0.71073)	MoKα (λ = 0.71073)
2Θ range for data collection/°	3.046 to 58.182	3.054 to 50.698	3.052 to 50.696
Index ranges	-11 ≤ h ≤ 11, -23 ≤ k ≤ 22, -34 ≤ l ≤ 35	-10 ≤ h ≤ 10, -20 ≤ k ≤ 20, -32 ≤ l ≤ 32	-10 ≤ h ≤ 10, -20 ≤ k ≤ 20, -32 ≤ l ≤ 32
Reflections collected	29903	25643	25936
Independent reflections	16054 [R_{int} = 0.0630, R_{sigma} = 0.0947]	13221 [R_{int} = 0.0791, R_{sigma} = 0.0986]	13179 [R_{int} = 0.0442, R_{sigma} = 0.0632]
Data/restraints/parameters	16054/2115/1826	13221/2099/1811	13179/2449/1826
Goodness-of-fit on F^2	1.036	1.533	1.101
Final R indexes [I > 2σ(I)]	R_1 = 0.1317, wR_2 = 0.3631	R_1 = 0.1355, wR_2 = 0.3989	R_1 = 0.1227, wR_2 = 0.2500
Final R indexes [all data]	R_1 = 0.1931, wR_2 = 0.4084	R_1 = 0.1856, wR_2 = 0.4409	R_1 = 0.1600, wR_2 = 0.2673
Largest diff. peak/hole / e Å^{-3}	2.59/-1.56	3.10/-1.41	1.54/-1.75

Supplementary Table 9. Crystal data for **4** (273 K, vacuum)_2 and **4^{CO2}** (200 K, 10 bar CO_2)

Identification code	**4 (273 K, vacuum)_2**	**4^{CO2} (200 K, 10 bar)**
Empirical formula	$C_{52}H_{36}Ag_4F_{52}N_6O_8$	$C_{55.37}H_{36}Ag_4F_{52}N_6O_{14.73}$
Formula weight	2292.35	2440.36
Temperature/K	273.0(3)	199(1)
Crystal system	triclinic	triclinic
Space group	P-1	P-1
a/Å	8.6192(5)	8.5117(10)
b/Å	14.7080(10)	18.6972(19)
c/Å	16.8734(10)	26.352(2)
α/°	111.883(6)	82.007(7)
β/°	103.734(5)	80.974(8)
γ/°	91.216(5)	81.666(9)
Volume/Å^3	1913.8(2)	4069.0(7)
Z	1	2
ρ_{calc}g/cm^3	1.989	1.992
μ/mm^{-1}	1.187	1.129
F(000)	1110.0	2368.0
Crystal size/mm^3	0.45 × 0.25 × 0.2	0.45 × 0.25 × 0.2
Radiation	MoKα (λ = 0.71073)	Mo Kα (λ = 0.71073)
2Θ range for data collection/°	3.164 to 50.694	3.152 to 41.63
Index ranges	-10 ≤ h ≤ 10, -17 ≤ k ≤ 16, -20 ≤ l ≤ 20	-8 ≤ h ≤ 8, -18 ≤ k ≤ 18, -26 ≤ l ≤ 26
Reflections collected	17848	24000
Independent reflections	6994 [R_{int} = 0.0235, R_{sigma} = 0.0328]	8499 [R_{int} = 0.0911, R_{sigma} = 0.0972]
Data/restraints/parameters	6994/1142/900	8499/537/1035
Goodness-of-fit on F^2	1.024	1.123
Final R indexes [I > 2σ(I)]	R_1 = 0.0694, wR_2 = 0.2055	R_1 = 0.1259, wR_2 = 0.2862
Final R indexes [all data]	R_1 = 0.0933, wR_2 = 0.2343	R_1 = 0.1584, wR_2 = 0.3080
Largest diff. peak/hole / e Å^{-3}	0.96/-0.65	1.86/-0.90

Supplementary Table 10. Crystal data for **5-MeOH**, **5** (295 K, vacuum) and **5** (295 K, 10 bar CO_2)

Identification code	**5-MeOH**	**5 (295 K, vacuum)**	**5 (295 K, 10 bar)**
Empirical formula	$C_{59}H_{46}Ag_4Cl_2F_{60}N_6O_{10}$	$C_{56}H_{36}Ag_4F_{60}N_6O_8$	$C_{56}H_{36}Ag_4F_{60}N_6O_8$
Formula weight	2641.40	2492.39	2492.39
Temperature/K	100.15	294.15(10)	294.17(10)
Crystal system	triclinic	triclinic	triclinic
Space group	P-1	P-1	P-1
a/Å	15.4784(9)	8.6352(7)	8.6397(4)
b/Å	17.7752(11)	14.7234(14)	14.7490(7)
c/Å	17.7958(11)	17.9907(18)	18.0091(9)
α/°	77.042(3)	110.791(9)	110.867(4)
β/°	69.915(3)	101.849(7)	101.856(4)
γ/°	67.614(3)	90.529(7)	90.534(4)
Volume/$Å^3$	4227.2(5)	2084.4(4)	2090.03(18)
Z	2	1	1
$\rho_{calc}g/cm^3$	2.075	1.986	1.980
μ/mm^{-1}	1.166	1.112	1.109
F(000)	2568.0	1206.0	1206.0
Crystal size/mm^3	0.3 × 0.25 × 0.25	0.25 × 0.2 × 0.2	0.25 × 0.2 × 0.2
Radiation	MoKα (λ = 0.71073)	MoKα (λ = 0.71073)	MoKα (λ = 0.71073)
2Θ range for data collection/°	2.45 to 50.7	2.97 to 50.698	2.968 to 50.7
Index ranges	$-18 \leq h \leq 18, -21 \leq k \leq 21, -21 \leq l \leq 21$	$-10 \leq h \leq 10, -17 \leq k \leq 17, -21 \leq l \leq 21$	$-10 \leq h \leq 10, -17 \leq k \leq 17, -21 \leq l \leq 21$
Reflections collected	80876	19955	19876
Independent reflections	15479 [R_{int} = 0.0773, R_{sigma} = 0.0546]	7621 [R_{int} = 0.0193, R_{sigma} = 0.0267]	7643 [R_{int} = 0.0206, R_{sigma} = 0.0264]
Data/restraints/parameters	15479/282/1198	7621/1365/1008	7643/1366/1008
Goodness-of-fit on F^2	1.071	1.416	0.999
Final R indexes [I > 2σ(I)]	R_1 = 0.0534, wR_2 = 0.1413	R_1 = 0.0899, wR_2 = 0.2897	R_1 = 0.0768, wR_2 = 0.2302
Final R indexes [all data]	R_1 = 0.0670, wR_2 = 0.1535	R_1 = 0.1150, wR_2 = 0.3340	R_1 = 0.1038, wR_2 = 0.2800
Largest diff. peak/hole / e $Å^{-3}$	2.04/-1.16	1.64/-1.10	1.18/-1.08

Supplementary Table 11. Crystal data for **5** (250 K, 10 bar CO_2), **5** (230 K, 10 bar CO_2) and $\mathbf{5^{CO2}}$ (200 K, 10 bar)

Identification code	**5 (250 K, 10 bar)**	**5 (230 K, 10 bar)**	$\mathbf{5^{CO2}}$ **(200 K, 10 bar)**
Empirical formula	$C_{28}H_{18}Ag_2F_{30}N_3O_4$	$C_{56}H_{36}Ag_4F_{60}N_6O_8$	$C_{56.76}H_{36}Ag_4F_{60.35}N_6O_{9.52}$
Formula weight	2492.39	2492.39	2532.48
Temperature/K	250.01(10)	230.00(10)	199.9(5)
Crystal system	triclinic	triclinic	triclinic
Space group	P-1	P-1	P-1
a/Å	8.5951(4)	8.6146(7)	8.5763(4)
b/Å	14.6921(6)	14.7423(13)	18.0371(9)
c/Å	18.0362(8)	18.1319(15)	27.7126(13)
α/°	111.689(4)	112.340(8)	76.843(4)
β/°	101.546(4)	101.272(8)	81.288(4)
γ/°	90.684(3)	90.886(7)	79.331(4)
Volume/$Å^3$	2064.29(16)	2078.6(3)	4075.4(4)

Z	1	1	2
ρ_{calc}g/cm^3	2.005	1.991	2.064
μ/mm^{-1}	1.123	1.115	1.141
F(000)	1206.0	1206.0	2452.0
Crystal size/mm^3	0.25 × 0.2 × 0.2	0.25 × 0.2 × 0.2	0.25 × 0.2 × 0.2
Radiation	MoKα (λ = 0.71073)	MoKα (λ = 0.71073)	MoKα (λ = 0.71073)
2Θ range for data collection/°	2.996 to 50.696	3.002 to 50.698	4.204 to 50.698
Index ranges	$-10 \le h \le 10$, $-17 \le k \le 17$, $-21 \le l \le 21$	$-10 \le h \le 10$, $-17 \le k \le 17$, $-21 \le l \le 21$	$-10 \le h \le 10$, $-21 \le k \le 21$, $-33 \le l \le 33$
Reflections collected	19515	18582	38928
Independent reflections	7541 [R_{int} = 0.0198, R_{sigma} = 0.0263]	7569 [R_{int} = 0.0502, R_{sigma} = 0.0524]	14892 [R_{int} = 0.0334, R_{sigma} = 0.0425]
Data/restraints/parameters	7541/1360/1008	7569/1328/1008	14892/2084/1844
Goodness-of-fit on F^2	1.028	1.031	1.172
Final R indexes [I > 2σ(I)]	R_1 = 0.0797, wR_2 = 0.2333	R_1 = 0.0920, wR_2 = 0.2688	R_1 = 0.1152, wR_2 = 0.2560
Final R indexes [all data]	R_1 = 0.1049, wR_2 = 0.2720	R_1 = 0.1250, wR_2 = 0.3080	R_1 = 0.1432, wR_2 = 0.2703
Largest diff. peak/hole / e Å^{-3}	1.26/-1.06	2.21/-0.89	1.37/-0.98

3.2 Crystal structures and solid-vapour reaction manifold in coordination polymers 1-5-ROH and 1-5.

A series of closely related coordination polymers $[Ag_4(CO_2(CF_2)_mCF_3)_4(TMP)_3(ROH)_2]\cdot xCH_2Cl_2$ (m = 2 (**1-MeOH**), 3 (**2-EtOH**), 4 (**3-EtOH**), 5 (**4-MeOH**) and 6 (**5-MeOH**); ROH = methanol and ethanol; x = 0 and 1(**5-MeOH** only)) have been synthesized as large colourless needle crystals by slow diffusion of a dichloromethane solution of 2,3,5,6-tetramethylpyrizine (TMP) into an alcoholic solution of $Ag(CO_2(CF_2)_mCF_3)$ (m = 2, 3, 4, 5 or 6) at 278 K. In this family of crystal structures, pairs of silver ions are bridged by pairs of perfluorocarboxylate ligands. One ligand directly bridges both silver atoms, while the second ligand adopts an asymmetric coordination mode where it chelates one silver ion and hydrogen bonds to a coordinated alcohol molecule via an O–H···O hydrogen bond. The resulting disilver units $Ag_2(O_2C(CF_2)_mCF_3)_2(ROH)$ (m = 2-6; ROH = methanol or ethanol) are further linked by pairs of TMP ligands resulting in silver tetramer units $Ag_4(O_2C(CF_2)_mCF_3)_4(ROH)_2(TMP)_2$ (m =2-6; ROH = methanol or ethanol). The tetramer silver units are linked into polymeric zig-zag tapes via additional single-bridge TMP ligands. Alcohol-containing coordination polymers assemble in a rod-like distorted hexagonal packing motif with the perfluoroalkyl groups of neighbouring polymers forming an interdigitated perfluoroalkyl layer (e.g. see Figure 2). The perfluoroalkyl groups are disordered in the crystal structure, suggesting some degree of mobility as consequence of the weak dispersion interactions formed. The fluoroalkyl groups can also adopt two conformations (*gauche* or *anti*) around the C_n-C_{n+1} (n = 2-4) bonds. **1-MeOH** coordination adopts a 1:1 ratio of *gauche*/*anti* conformations, whereas for **2-EtOH**, **3-EtOH**, **4-MeOH** and **5-MeOH** a fully *anti* conformation is observed. Coordination polymer **5-MeOH** also has disordered non-coordinated CH_2Cl_2 molecules (one per formula unit) situated in the (010) planes formed by the interdigitated perfluorocarboxylate ligands. The synthesis of coordination polymers **1-MeOH** and **2-EtOH** present small amount of colourless plate crystals of 2D coordination polymers **1** and **2**, $[Ag_4(O_2C(CF_2)_mCF_3)_4(TMP)_2]$ (m = 2 and 3, respectively). Purification of coordination polymers **1-MeOH** and **2-EtOH** was achieved by separation of the colourless plate crystals of 2D coordination polymer $[Ag_4(O_2C(CF_2)_mCF_3)_4TMP_2]_n$ (m = 2 and 3) under a microscope.

In an earlier series of reports, we have shown the reversible uptake and release of the coordinated alcohol molecules in **1-MeOH** and **2-EtOH**.[S3-S4] Three separate crystallographic experiments have now

been undertaken to remove the coordinated alcohol from **3-EtOH**, **4-MeOH** and **5-MeOH** and the solvated CH_2Cl_2 from the latter. Release of the coordinated alcohol was achieved upon mild heating without losing crystallinity. Single crystals of **3-EtOH**, **4-MeOH** and **5-MeOH** were first structurally characterized at 100 K. Then, the crystals were heated at 340 K for 1 hr, under vacuum for **3-EtOH** and in air for **4-MeOH** and **5-MeOH**, after which, and based on repeated measurement of the unit cell, the crystals had transformed into coordination polymers **3** (polymorph $\mathbf{3}^{\mathbf{HT}}$), **4** and **5**, respectively. Then, the crystals were cooled to room temperature, a full data set was measured and crystal structure determination confirmed the presence of the alcohol-free coordination polymers.

The crystal structure of the closely related coordination polymers $[Ag_4(CO_2(CF_2)_mCF_3)_4(TMP)_3]$ (m = 4 ($\mathbf{3}^{\mathbf{HT}}$), 5 (**4**) and 6 (**5**)) confirms the intramolecular ligand substitution at alternate silver metal centres, where the coordinated alcohol molecule is replaced by the oxygen atom of a now fully bridging carboxylate $CF_3(CF_2)_mCO_2^-$ (m = 4 ($\mathbf{3}^{\mathbf{HT}}$), 5 (**4**) and 6 (**5**)) ligand (Supplementary Figure 10). The process involves the cleavage and formation of covalent bonds as well as the cleavage of hydrogen bonds. The plane formed by the $Ag_4(CO_2(CF_2)_mCF_3)_4(TMP)_2$ (m = 4 ($\mathbf{3}^{\mathbf{HT}}$), 5 (**4**) and 6 (**5**)) tetramer units are perfectly parallel respect to each other, and form a dihedral angle of 34.1(3) ° $\mathbf{3}^{\mathbf{HT}}$, 37.24(19)° **4** and 36.7(2)° **5** with the plane formed by the single-bridge TMP ligand. The fluoroalkyl chains adopt an *anti* conformation for $\mathbf{3}^{\mathbf{HT}}$, **4** and **5**. Then, crystals were cooled to 100 K and through measurement of unit cell it was confirmed the presence of coordination polymer **4** and **5**, while coordination polymer $\mathbf{3}^{\mathbf{HT}}$ went through a phase transformation to form the low temperature $\mathbf{3}^{\mathbf{LT}}$ polymorph. Overall reduction of the $\mathbf{3}^{\mathbf{LT}}$ symmetry results in the presence of 3.5 $[Ag_4(CO_2(CF_2)_4CF_3)_4(TMP)_3]$ molecules in the asymmetric unit as consequence of the slight twist in the dihedral angle between the $Ag_4(CO_2(CF_2)_4CF_3)_4(TMP)_2$ tetramers. The structure of the fluoroalkyl chains changed from a 100% *anti* to a 1:1 *gauche*/*anti* conformation ratio.

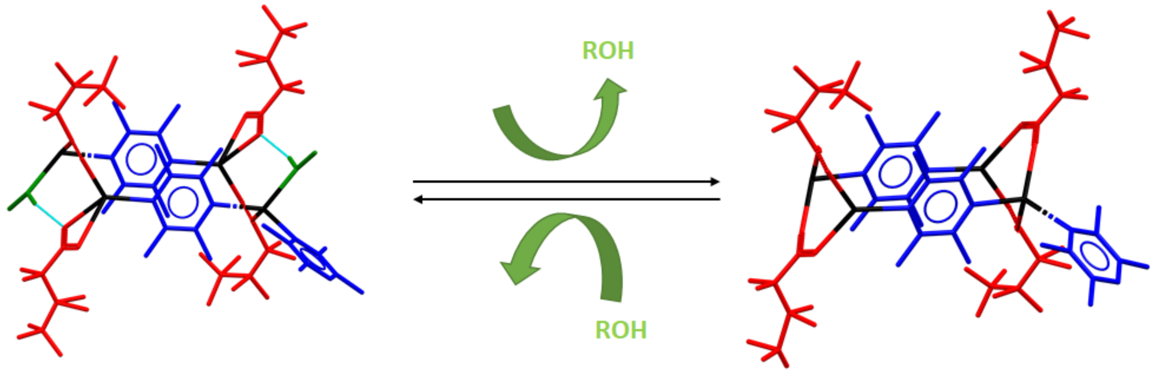

Supplementary Figure 10. Reversible uptake and release of coordinated methanol molecule in coordination polymers **1-MeOH** and **1**, showing corresponding changes in coordination mode of half of the carboxylate ligands between chelating (**1-MeOH**) and bridging (**1**). Silver atoms are represented in black, heptafluorobutanoate in red, TMP in blue and MeOH in green.

Alcohol-free coordination polymers **1**($\mathbf{1_A}^{\mathbf{HT}}$, $\mathbf{1_A}^{\mathbf{LT}}$, $\mathbf{1_B}^{\mathbf{HT}}$ and $\mathbf{1_B}^{\mathbf{LT}}$), **2** ($\mathbf{2}^{\mathbf{HT}}$ and $\mathbf{2}^{\mathbf{LT}}$) and **3** ($\mathbf{3}^{\mathbf{HT}}$ and $\mathbf{3}^{\mathbf{LT}}$) include high-temperature (HT) and low-temperature (LT) polymorphs, which differ in the conformation of the fluoroalkyl groups (*anti*/*gauche*) about one of their alkyl C−C bonds, and result in a reduction in symmetry for the LT forms of **1-3** relative to the HT forms. Additionally, polymorphs of coordination polymer **1** can be further subdivided into two classifications (A or B) depending on the relative arrangement of neighbouring 1D coordination polymer tapes.[S3] The polymorphism in coordination polymers **1-3** arises from the conformational flexibility shown in the fluoroalkyl groups (bent (*gauche*) and linear (*anti*), and flexible coordination geometry around Ag−N bonds, which allows pivotal rotation

of the single-bridge TMP ligand about its N···N axis, as shown in the change of the dihedral angle of the planes formed by the single-bridge TMP ligand and the Ag_4 plane of the $Ag_4(CO_2(CF_2)_mCF_3)_4(TMP)_2$ (m = 2, 3, 4, 5 and 6) tetramer units. The crystal structures of the alcohol-containing and alcohol-free coordination polymers are shown in Supplementary Figures 11-22.

The reversibility of the alcohol release process was previously reported by our group for coordination polymers **1** and **2**,[S3-S4] wherein methanol, ethanol and isopropanol were taken up by **1** in a single-crystal-to-single-crystal manner, and ethanol was taken up by **2** as polycrystalline sample. The reversibility of the alcohol uptake by coordination polymers **3-5** has not been investigated but it could be assumed that coordination polymers **3-5** follow similar behaviour to that of compounds **1** and **2**.

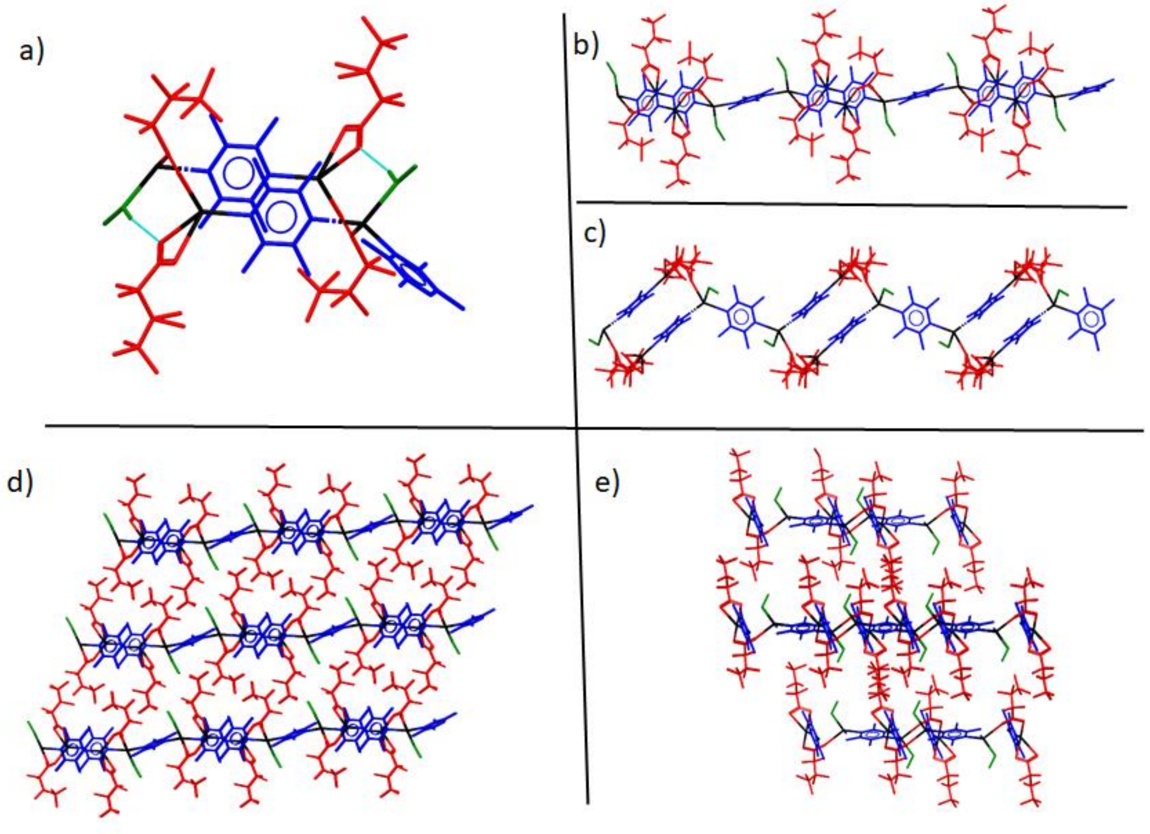

Supplementary Figure 11. Crystal structure of **1-MeOH** showing: (a) formula unit; (b) lateral view of the 1D coordination polymer; (c) top view of the 1D coordination polymer; (d) lateral view of the packing of the 1D coordination polymers; (e) hexagonal packing motif of the 1D coordination polymers. Silver atoms are represented in black, perfluorocarboxylates in red, TMP in blue, alcohol molecules in green and hydrogen bonds shown as blue dashed lines.

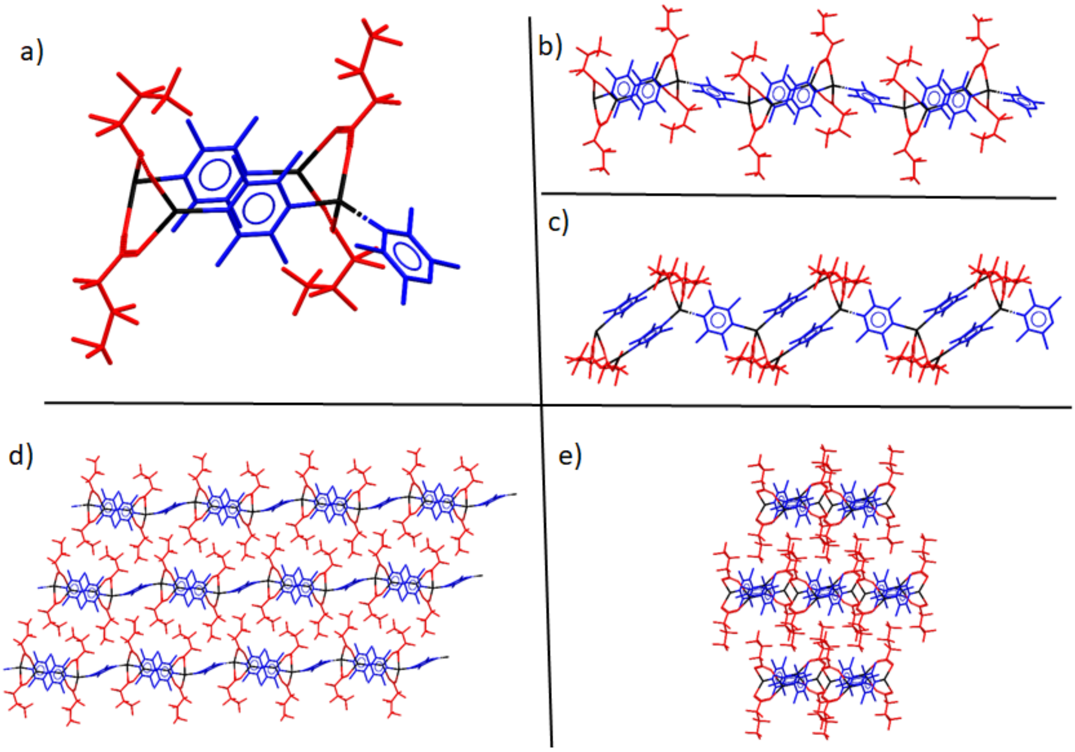

Supplementary Figure 12. Crystal structure of **1** (polymorph $\mathbf{1_A}^{\mathbf{HT}}$) showing: (a) formula unit; (b) lateral view of the 1D coordination polymer; (c) top view of the 1D coordination polymer; (d) lateral view of the packing of the 1D coordination polymers; (e) hexagonal packing motif of the 1D coordination polymers. Colour coding as in Supplementary Figure 11.

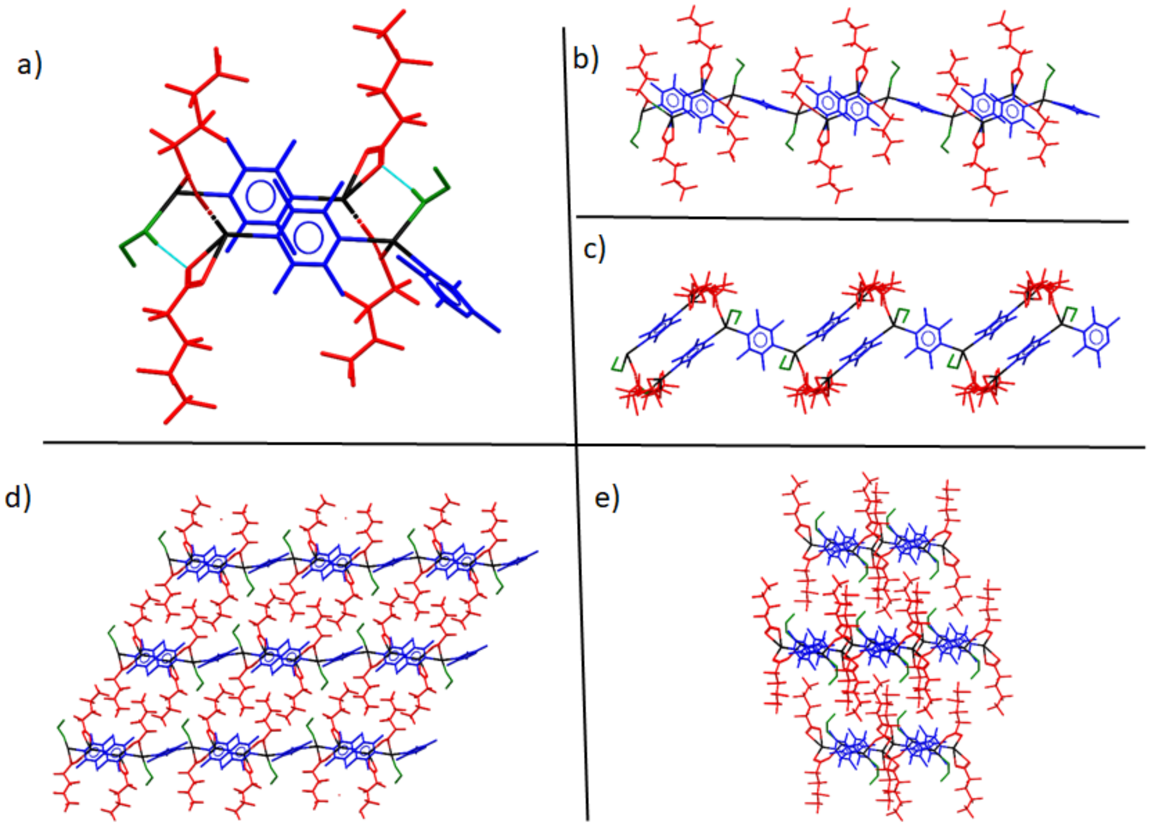

Supplementary Figure 13. Crystal structure of **2-EtOH** showing: (a) formula unit; (b) lateral view of the 1D coordination polymer; (c) top view of the 1D coordination polymer; (d) lateral view of the packing of the 1D

coordination polymers; (e) hexagonal packing motif of the 1D coordination polymers. Colour coding as in Supplementary Figure 11.

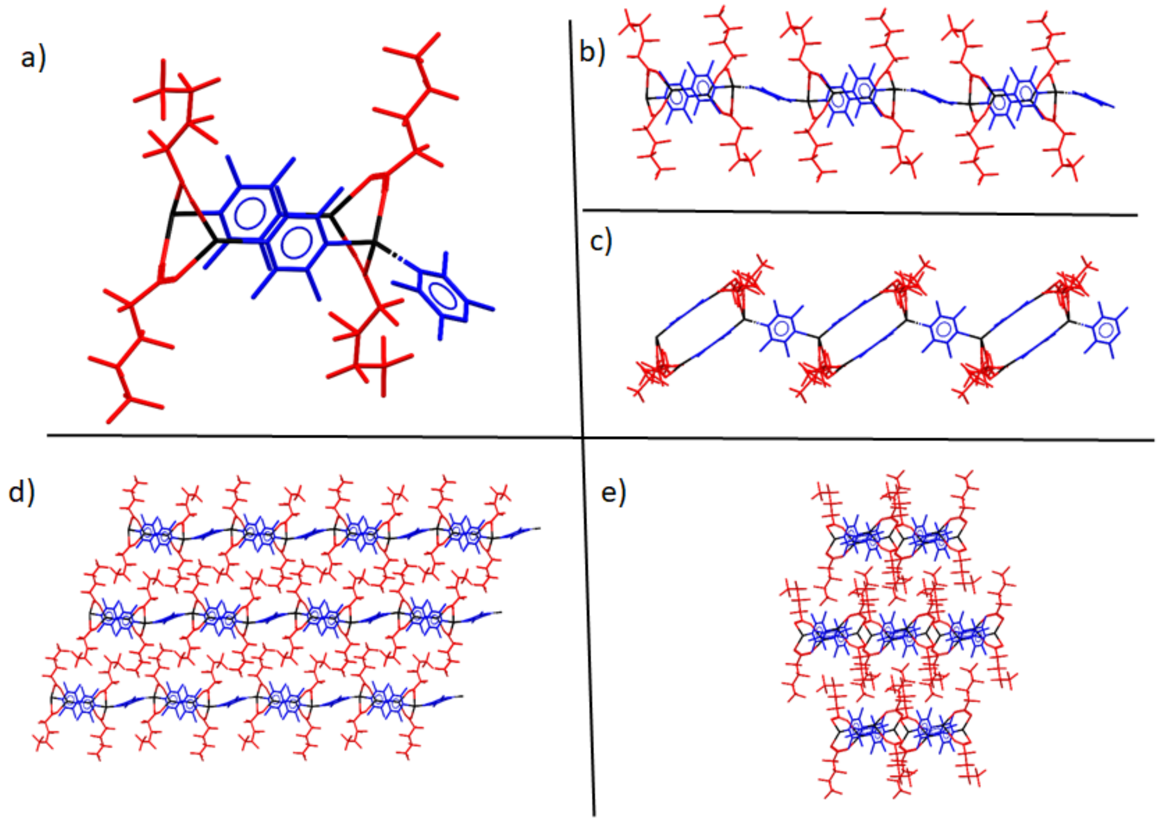

Supplementary Figure 14. Crystal structure of **2** (polymorph $\mathbf{2^{HT}}$) showing: (a) formula unit; (b) lateral view of the 1D coordination polymer; (c) top view of the 1D coordination polymer; (d) lateral view of the packing of the 1D coordination polymers; (e) hexagonal packing motif of the 1D-coordination polymers. Colour coding as in Supplementary Figure 11.

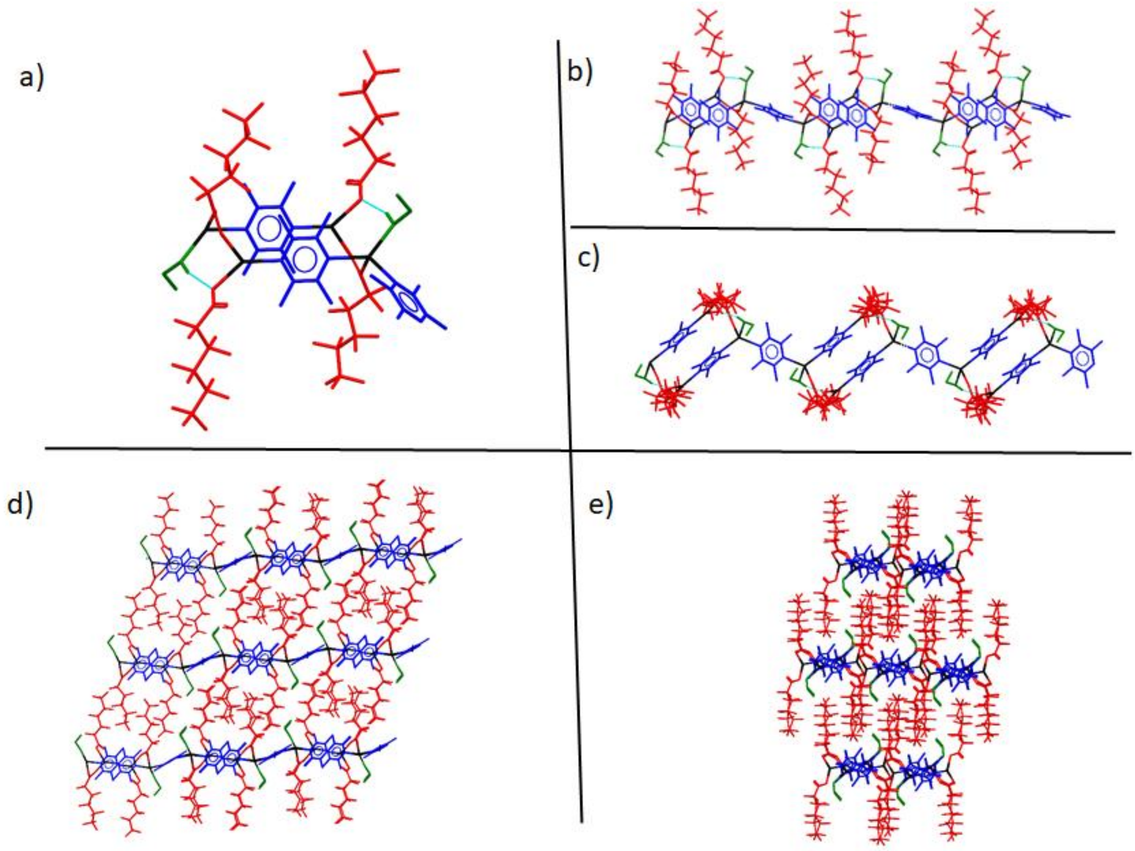

Supplementary Figure 15. Crystal structure of **3-EtOH** showing: (a) formula unit; (b) lateral view of the 1D coordination polymer; (c) top view of the 1D coordination polymer; (d) lateral view of the packing of the 1D coordination polymers; (e) hexagonal packing motif of the 1D coordination polymers. Colour coding as in Supplementary Figure 11.

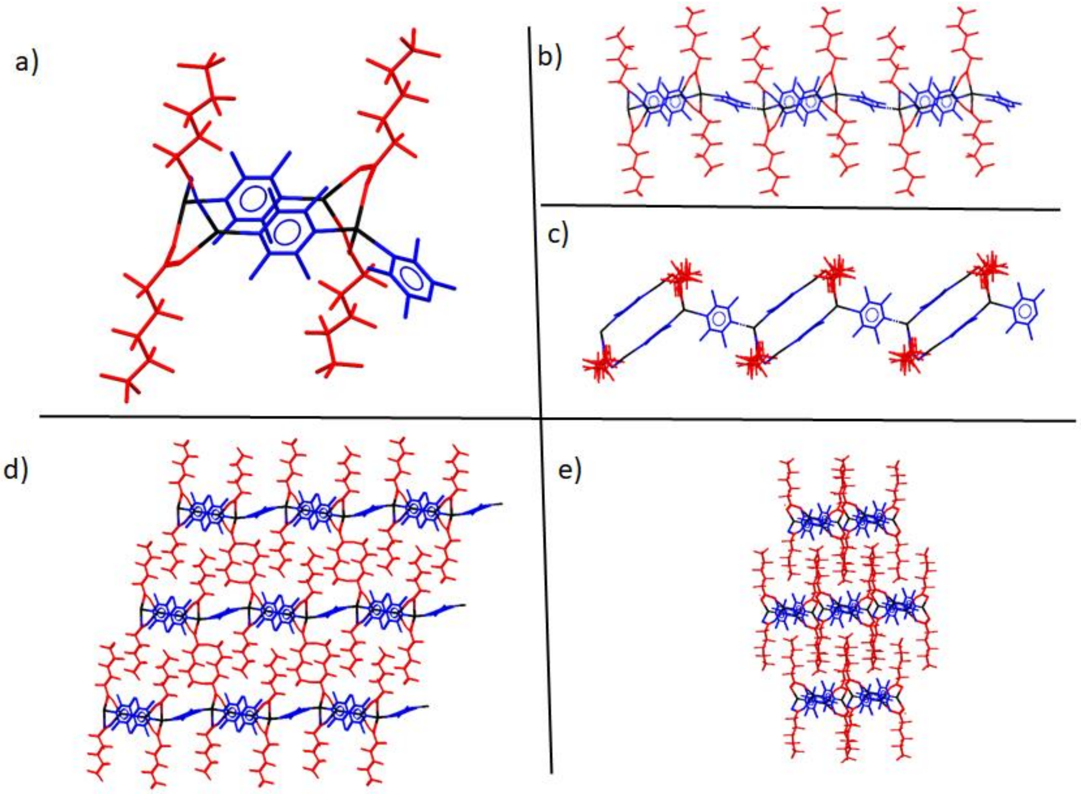

Supplementary Figure 16. Crystal structure of **3** (polymorph $\mathbf{3^{HT}}$) showing: (a) formula unit; (b) lateral view of the 1D coordination polymer; (c) top view of the 1D coordination polymer; (d) lateral view of the packing of the 1D coordination polymers; (e) hexagonal packing motif of the 1D-coordination polymers. Colour coding as in Supplementary Figure 11.

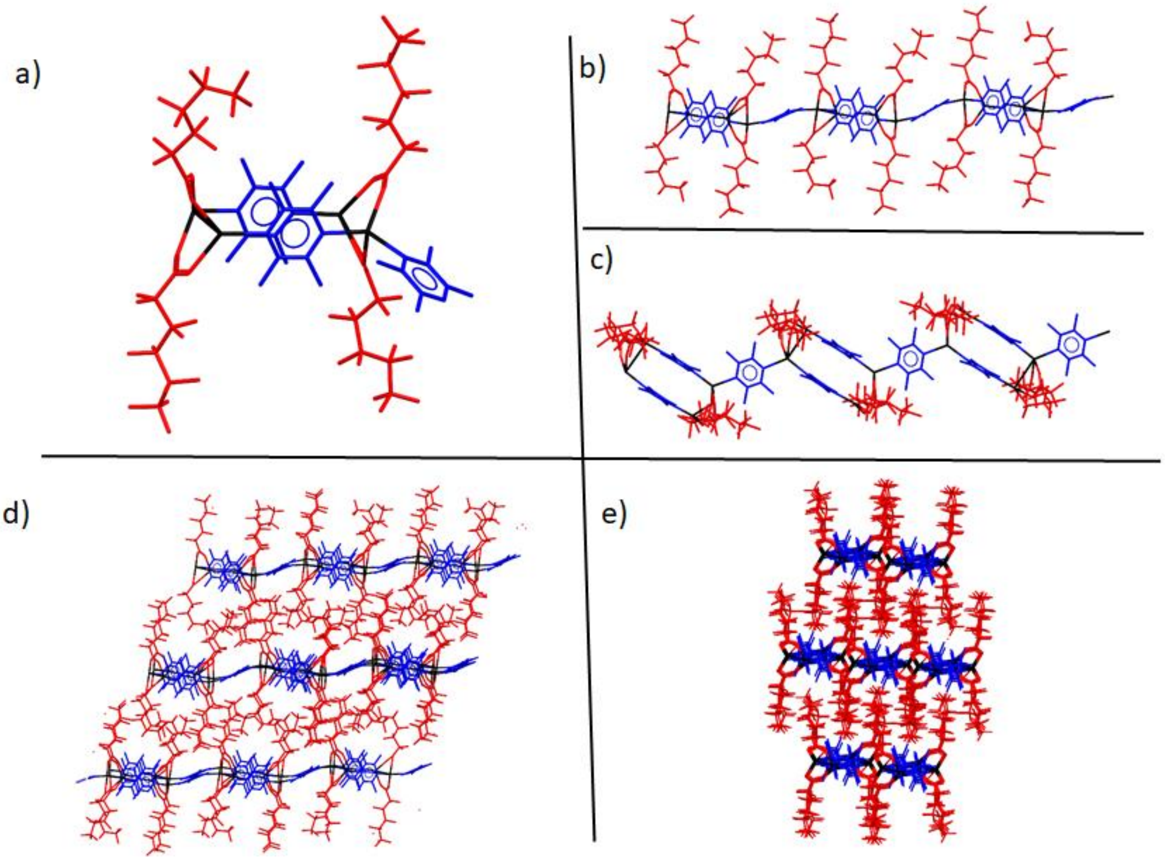

Supplementary Figure 17. Crystal structure of **3** (polymorph $\mathbf{3^{LT}}$) showing: (a) formula unit; (b) lateral view of the 1D coordination polymer; (c) top view of the 1D coordination polymer; (d) lateral view of the packing of the 1D coordination polymers; (e) hexagonal packing motif of the 1D-coordination polymers. Colour coding as in Supplementary Figure 11.

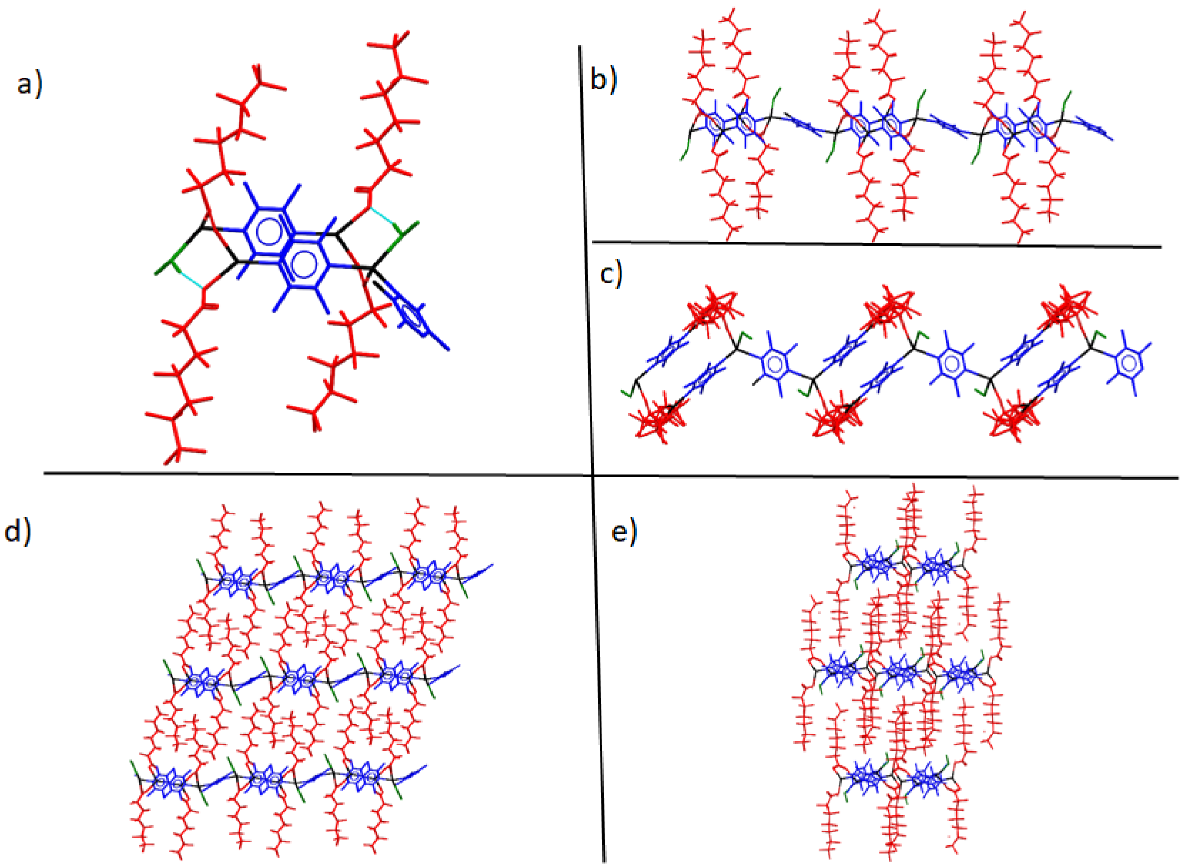

Supplementary Figure 18. Crystal structure of **4-MeOH** showing: (a) formula unit; (b) lateral view of the 1D coordination polymer; (c) top view of the 1D coordination polymer; (d) lateral view of the packing of the 1D coordination polymers; (e) hexagonal packing motif of the 1D-coordination polymers. Colour coding as in Supplementary Figure 11.

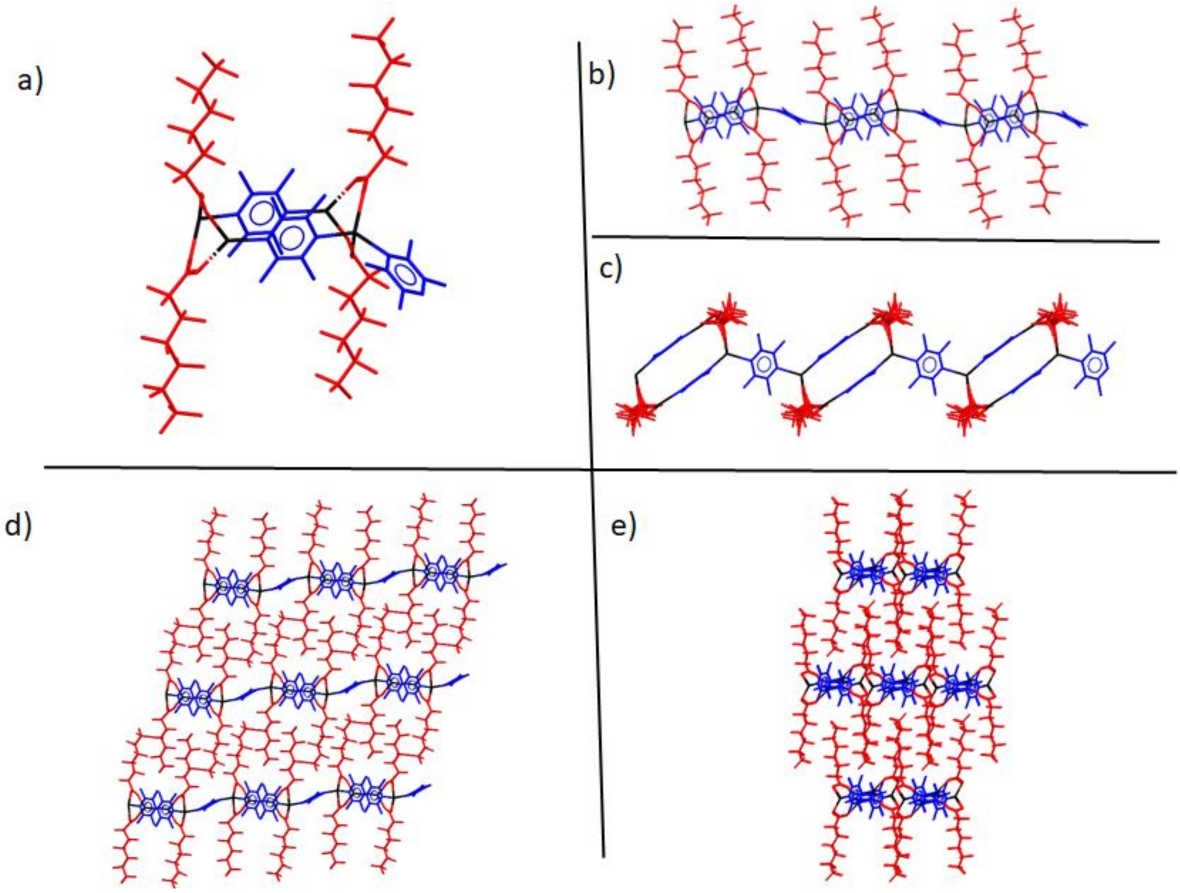

Supplementary Figure 19. Crystal structure of **4** showing: (a) formula unit; (b) lateral view of the 1D coordination polymer; (c) top view of the 1D coordination polymer; (d) lateral view of the packing of the 1D coordination polymers; (e) hexagonal packing motif of the 1D-coordination polymers. Colour coding as in Supplementary Figure 11.

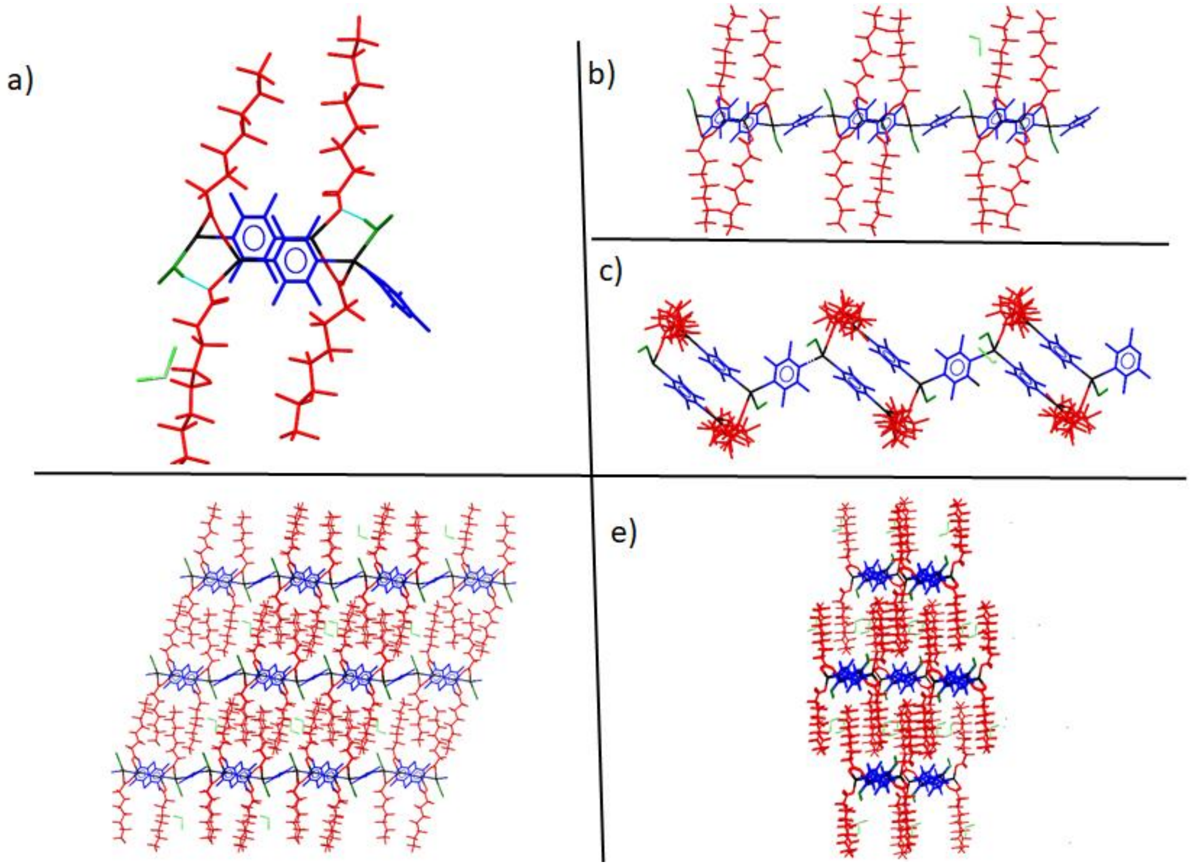

Supplementary Figure 20. Crystal structure of **5-MeOH** showing: (a) formula unit; (b) lateral view of the 1D coordination polymer; (c) top view of the 1D coordination polymer; (d) lateral view of the packing of the 1D coordination polymers; € hexagonal packing motif of the 1D-coordination polymers. Colour coding as in Supplementary Figure 11 with CH_2Cl_2 molecule shown in light green.

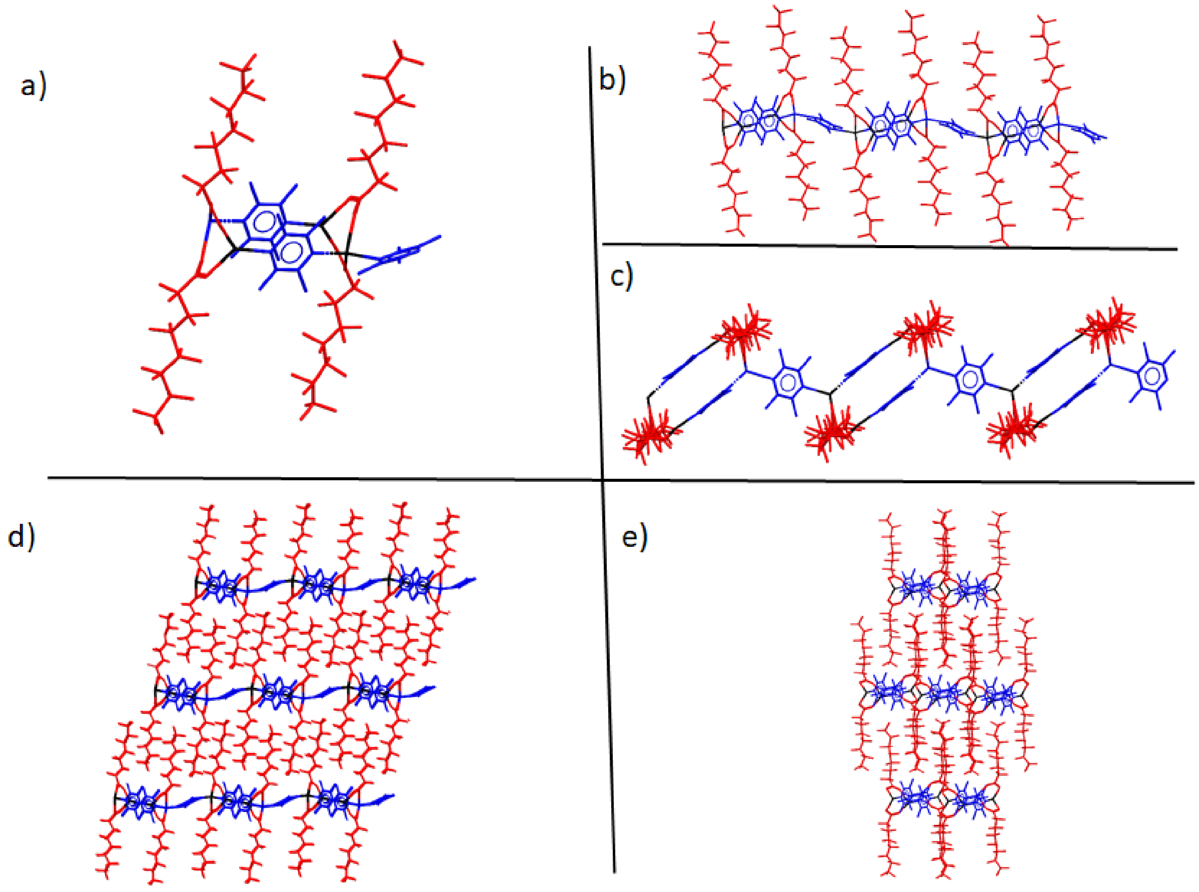

Supplementary Figure 21. Crystal structure of **5** showing (a) formula unit; (b) lateral view of the 1D coordination polymer; (c) top view of the 1D coordination polymer; (d) lateral view of the packing of the 1D coordination polymers; (e) hexagonal packing motif of the 1D-coordination polymers. Colour coding as in Supplementary Figure 11.

3.3 CO_2 adsorption in coordination polymers 1-5 studied by single-crystal X-ray diffraction (SCXRD). A series of SCXRD experiments have been undertaken using a gas rig and a gas cell on a dual-source Rigaku Synergy FR-X rotating anode diffractometer (see Section 2) and at beamline I19 at Diamond Light Source,[S2] to investigate the adsorption of CO_2 gas molecules in single crystals of **1-5**. Experimental conditions of the gravimetric adsorption experiments cannot be exactly replicated in the SCXRD experiments due to instrumentation set-up limitations, including gas pressure range and temperature control inside the capillary of the gas cell (Supplementary Figure 22). In the final crystal structure models, CO_2 molecules were located from difference electron density maps and refined as rigid bodies with unconstrained occupancies. CO_2 molecules are located in sites in two regions (e.g. Figure 3a). The first region lies close to the single-bridge TMP ligands, although some interaction with proximate CF_2 groups is often present, while sites in the second region are better classified as being predominantly associated with the interdigitated perfluoroalkyl ligands.

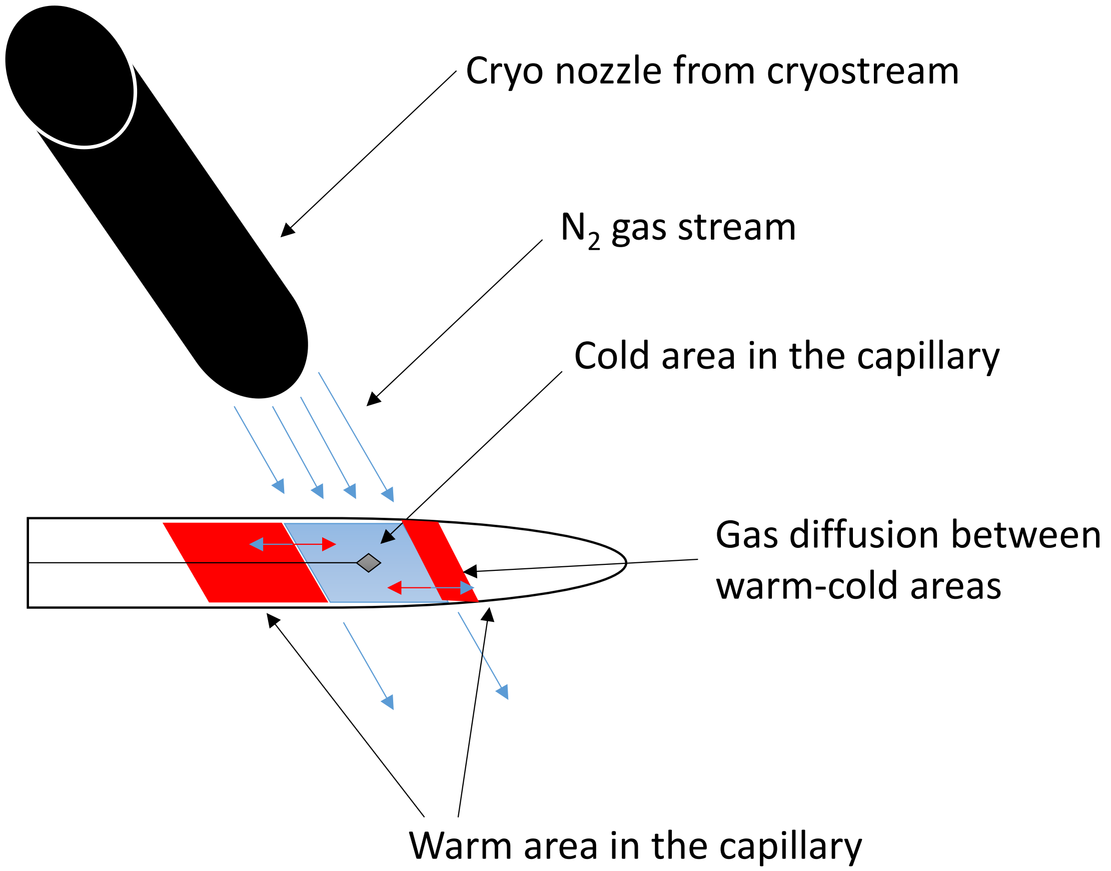

Supplementary Figure 22. Scheme illustrating gas diffusion between warm and cold areas in the capillary of the gas cell

Coordination polymer **1** (polymorph $\mathbf{1_A}^{\mathbf{HT}}$) was structurally characterized at 2 bar CO_2 pressure and room temperature and showed no evidence of CO_2 uptake. The CO_2 pressure was increased to 10 bar and the temperature decreased to 253 K, where crystal structure determination revealed a modified form of coordination polymer $\mathbf{1_A}^{\mathbf{HT}}$ ($[Ag_4(O_2C(CF_2)_2CF_3)_4(TMP)_3]\cdot 1.5CO_2$, denoted $\mathbf{1_A}^{\mathbf{CO2}}$) with a total of 1.5(1) molecules of CO_2 per formula unit, situated in two crystallographically independent locations. The structural change is accompanied by an approximate doubling in unit cell volume through a change in translational symmetry, but includes an increase in volume per formula unit (*V*/*Z*). At site 1 (region 1), a CO_2 molecule with an occupancy of 0.9(1) forms (O=C=O)$\pi\cdots\pi_{N\text{-}C}$ interactions ($O_{CO2}\cdots N_{TMP}$ 3.64(5) Å) with the single-bridge TMP ligand, multiple C−F···C_{CO_2} contacts (3.05(6)−3.38(5) Å) and weak C−H···O hydrogen bonds with the neighbouring methyl groups. At site 2 (region 2), a second CO_2 molecule with occupancy 0.6(1) was found in the (01−1) plane occupied by the interdigitated perfluorocarboxylates, forming an offset parallel dimer with its symmetry-generated (through an inversion centre) CO_2 molecule (C···O separation 3.64(6) Å), multiple C−F···C contacts (3.23(3)−3.33(4) Å), two C−H···O hydrogen bonds with the neighbouring TMP methyl group and forming a O=C=O$\cdots\pi_{N\text{-}C}$ interaction ($O_{CO2}\cdots C_{TMP}$ 3.97(3) Å) with the single-bridge TMP ligand. The temperature was further decreased to 232 K and a data set was collected at 10 bar CO_2 pressure, resulting in a further increase in *V*/*Z*. The new crystal structure of $\mathbf{1_A}^{\mathbf{CO2}}$ now $[Ag_4(O_2C(CF_2)_2CF_3)_4(TMP)_3]\cdot 1.9CO_2$ (Supplementary Figures 23 and 24), presented 1.9(1) CO_2 molecules per formula unit in three distinct crystallographic sites. Two of the CO_2 sites were equivalent to those preciously described. The new CO_2 location (site 3) was also found in the (01−1) plane (i.e.

region 2) within the interdigitated perfluorocarboxylates. The CO_2 molecule was found to be disordered over an inversion centre with an occupancy of 0.2(1), forming multiple C···F contacts (2.70(5)−3.17(4) Å). The structure of coordination polymer **1** was rearranged from that adopted by $\mathbf{1_A}^{\mathbf{HT}}$ to create enough space to accommodate the CO_2 molecules. The angle between the equatorial planes formed by the silver atoms of adjacent $Ag_4(TMP)_2$ tetramers were modified from perfectly parallel in the initial $\mathbf{1_A}^{\mathbf{HT}}$ to a dihedral angle of 24.59(4) ° (253 K) and 27.72(3) ° (232 K) in $\mathbf{1_A}^{\mathbf{CO2}}$. Also, the dihedral angle between the equatorial plane of the $Ag_4(TMP)_2$ tetramers and the plane formed by the single-bridge TMP units was reduced from 34.8(3) ° in coordination polymer $\mathbf{1_A}^{\mathbf{HT}}$ to 6.2(3) ° and 18.4(3) ° at 253 K and 12.0(3) ° and 15.8(3) ° at 232 K in $\mathbf{1_A}^{\mathbf{CO2}}$.

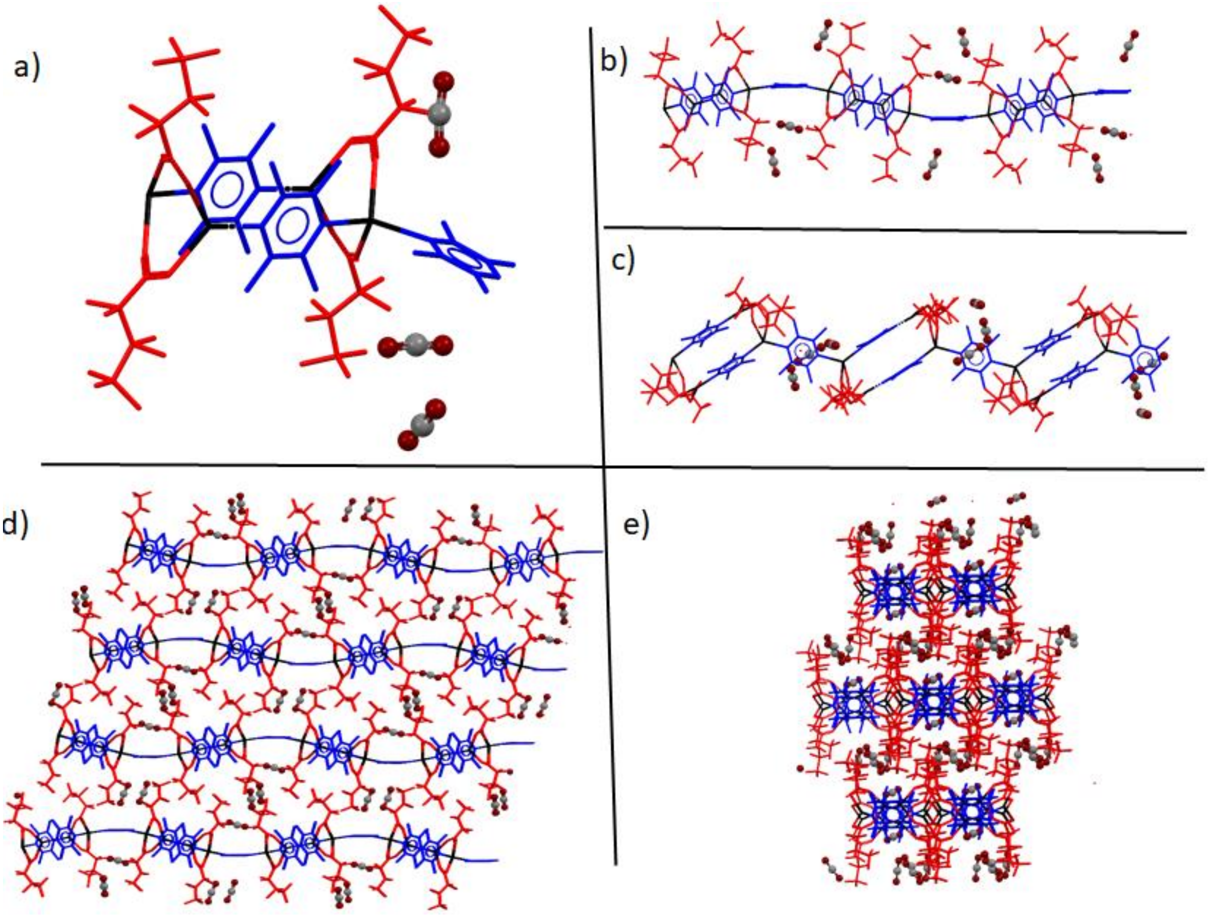

Supplementary Figure 23. Crystal structure of $\mathbf{1_A}^{\mathbf{CO2}}$ at 232 K and 10 bar CO_2, showing (a) formula unit; (b) lateral view of the 1D coordination polymer; (c) top view of the 1D coordination polymer; (d) lateral view of the packing of the 1D coordination polymers; (e) hexagonal packing motif of the 1D-coordination polymers. Colour coding as in Supplementary Figure 11. CO_2 molecules are represented with oxygen atoms in dark red and carbon in grey.

a) $\mathbf{1_A}^{\mathbf{CO2}}$ **site 1**

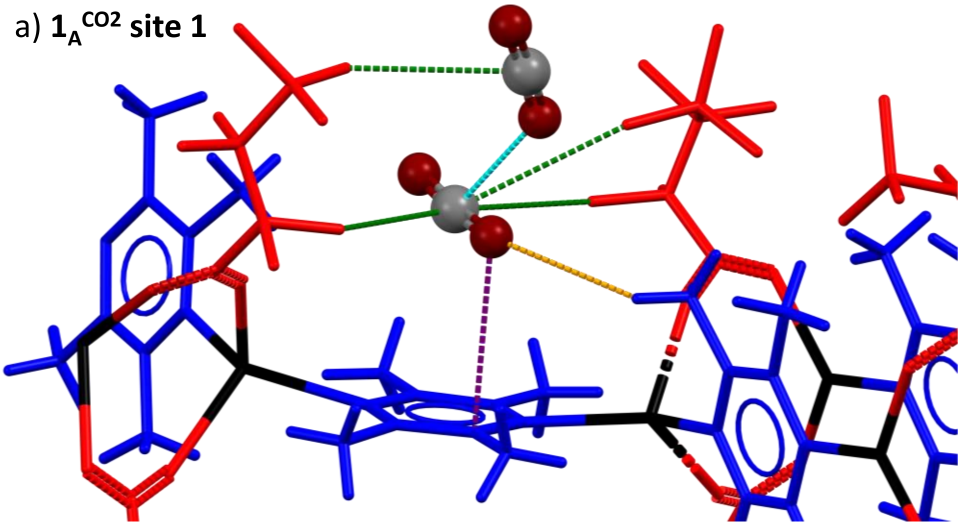

b) $\mathbf{1_A}^{CO2}$ site 2

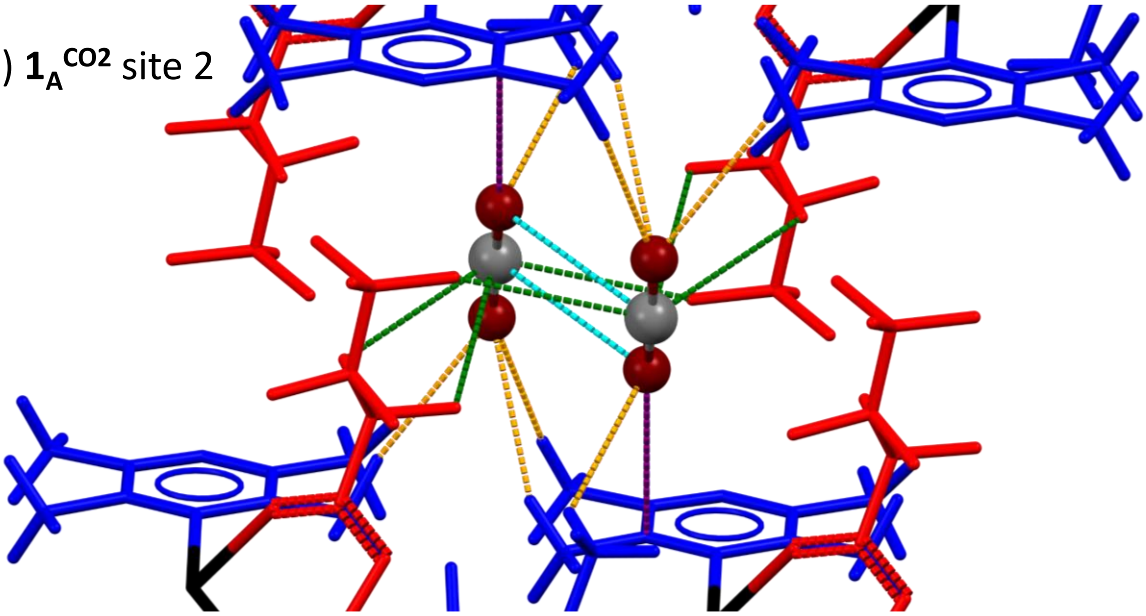

c) $\mathbf{1}_A^{CO2}$ site 3

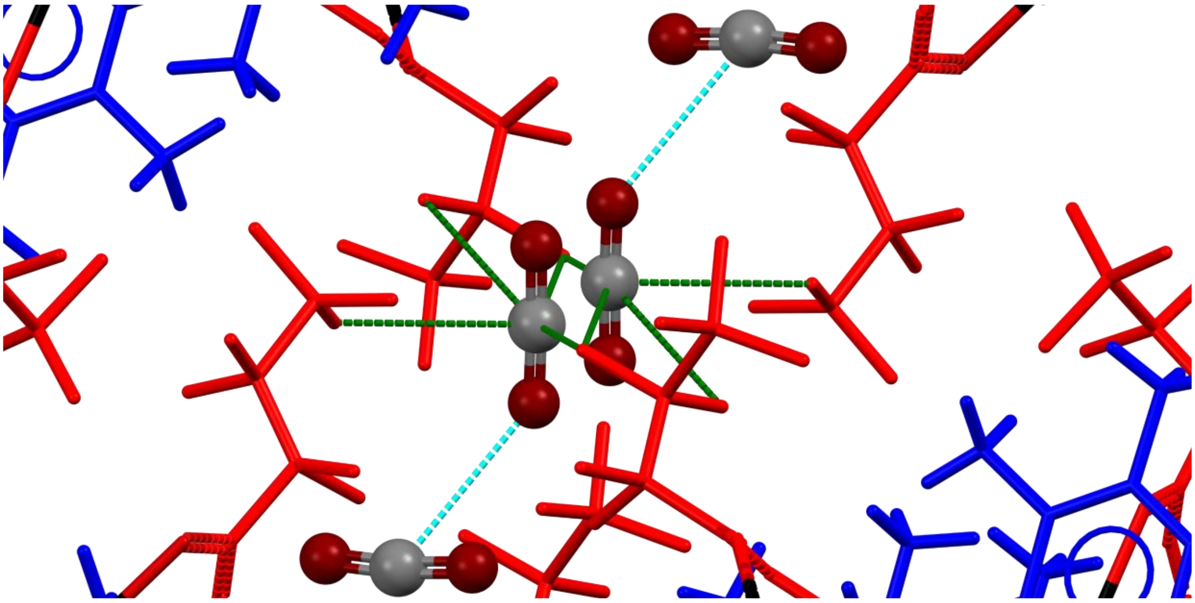

Supplementary Figure 24. Crystal structure of $\mathbf{1}_A^{CO2}$ at 232 K and 10 bar CO_2, showing the CO_2 interaction with the coordination polymer at (a) at site 1 (central) with neighbouring site 3 (peripheral); (b) at site 2, which contains an inversion-related pair of CO_2 molecules, and (c) at site 3, which also contains a single CO_2 molecule (central) disordered over an inversion centre (both components shown) and interacting with CO_2 molecules in neighbouring site 1 (peripheral). Colour coding as in Supplementary Figure 23, $CO_2\cdots\pi$ (TMP) shown as purple dashed lines, (TMP)C−H···O(CO_2) hydrogen bonds as orange dashed lines, C−F···C(CO_2) interactions as green dashed lines and (CO_2)C···O(CO_2) interactions in light blue.

Coordination polymer **2** (high-temperature polymorph $\mathbf{2}^{HT}$) was structurally characterized under vacuum at 298 K. the CO_2 pressure was increased to 10 bar and the temperature was decreased first to 273 K and then to 240 K. Crystal structure determinations at both temperatures showed no significant structural changes in coordination polymer $\mathbf{2}^{HT}$, although reduction in volume (V/Z) from 298 K to 273 K followed by increase in volume at 240 K, suggests some CO_2 uptake during the second temperature reduction, albeit insufficient to enable crystallographic detection. Upon further reduction in temperature to 215 K, however, the crystal structure revealed a modified form of coordination polymer **2** ($\mathbf{2}^{CO2}$, $[Ag_4(O_2C(CF_2)_3CF_3)_4(TMP)_3]\cdot 2CO_2$) with 2.0(1) CO_2 molecules per formula unit in two different crystallographic positions. Site 1 is fully occupied by a CO_2 molecule that forms (O=C=O)$\pi\cdots\pi_{N\text{-}C}$ interactions ((CO_2)O···C(TMP) 3.19(3) Å) with the single-bridge TMP ligand, an offset parallel dimer with the C−O atom pair of a carboxylate moiety (C···O 3.32(3) Å) and two C−F···C(CO_2) contacts (3.00(5)−3.07(7) Å). The CO_2 molecule is situated in the plane (010) formed by the four silver atoms of the $Ag_4(TMP)_2$ tetramers (region 1) and lies very close to an inversion centre, about which it is disordered in two (half-occupancy) positions. Site 2 lies in the (010) plane occupied by the interdigitated perfluorocarboxylate chains (region 2). There are two such symmetry-related sites per formula unit in which CO_2 molecules have an occupancy of 0.5(1). These CO_2 molecules form multiple C−F···C(CO_2) contacts (2.67(4)−3.22(4) Å). After a further decrease in temperature to 200 K, the crystal structure of $\mathbf{2}^{CO2}$ (200 K, 10 bar CO_2, Supplementary Figures 25 and 26) was isostructural to that previously described at 215 K. The structure shows a slight increase in occupancy of the CO_2 molecule (from 0.5(1) to 0.7(1)) located in site 2, resulting in 2.3(1) CO_2 molecules per formula unit. In order to accommodate

the CO_2 guest molecules (on cooling to 215 K and 200 K), the structure of coordination polymer **2** undergoes a rearrangement. The dihedral angle between the equatorial plane formed by the four silver atoms of the $Ag_4(TMP)_2$ tetramers and the plane formed by the single-bridge TMP units changed from 34.1(2) ° in coordination polymer $\mathbf{2^{HT}}$ to 90.5(4) ° in coordination polymer $\mathbf{2^{CO2}}$. Also, the orientation of the $Ag_4(TMP)_2$ tetramers respect the (010) plane changed from forming a dihedral angle of 11.3 ° in $\mathbf{2^{HT}}$ to 0.2 ° in $\mathbf{2^{CO2}}$. Finally, the temperature was raised to room temperature and the sample placed under vacuum. A full data set enabled crystal structure determination, confirming the presence of coordination polymer $\mathbf{2^{HT}}$, with a similar unit cell volume to the starting value, and the reversibility of the gas uptake.

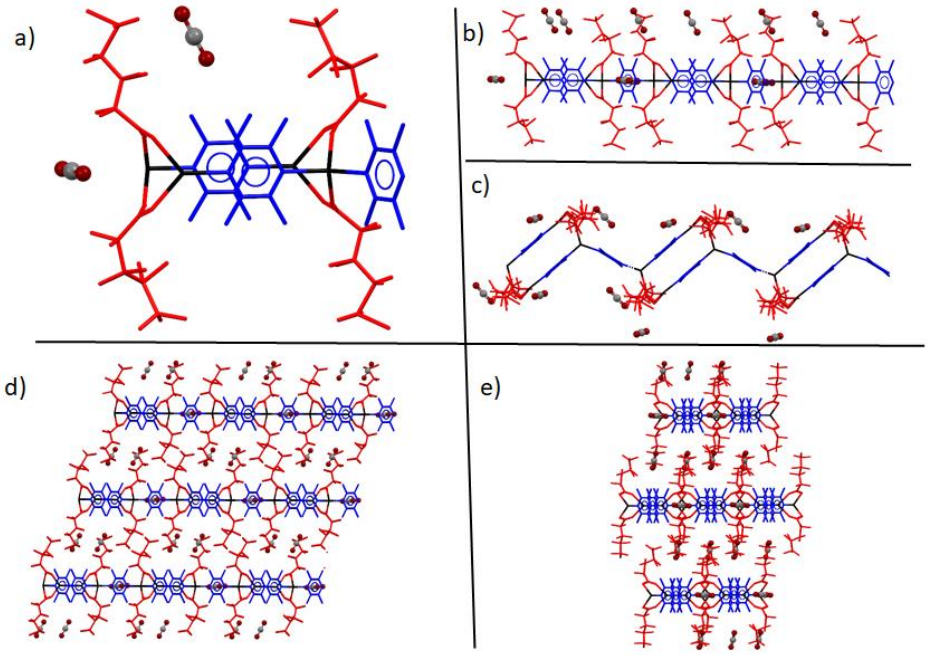

Supplementary Figure 25. Crystal structure of $\mathbf{2^{CO2}}$ (200 K, 10 bar CO_2), showing (a) formula unit; (b) lateral view of the 1D coordination polymer; (c) top view of the 1D coordination polymer; (d) lateral view of the packing of the 1D coordination polymers; (e) hexagonal packing motif of the 1D-coordination polymers. Colour coding as in Supplementary Figure 23.

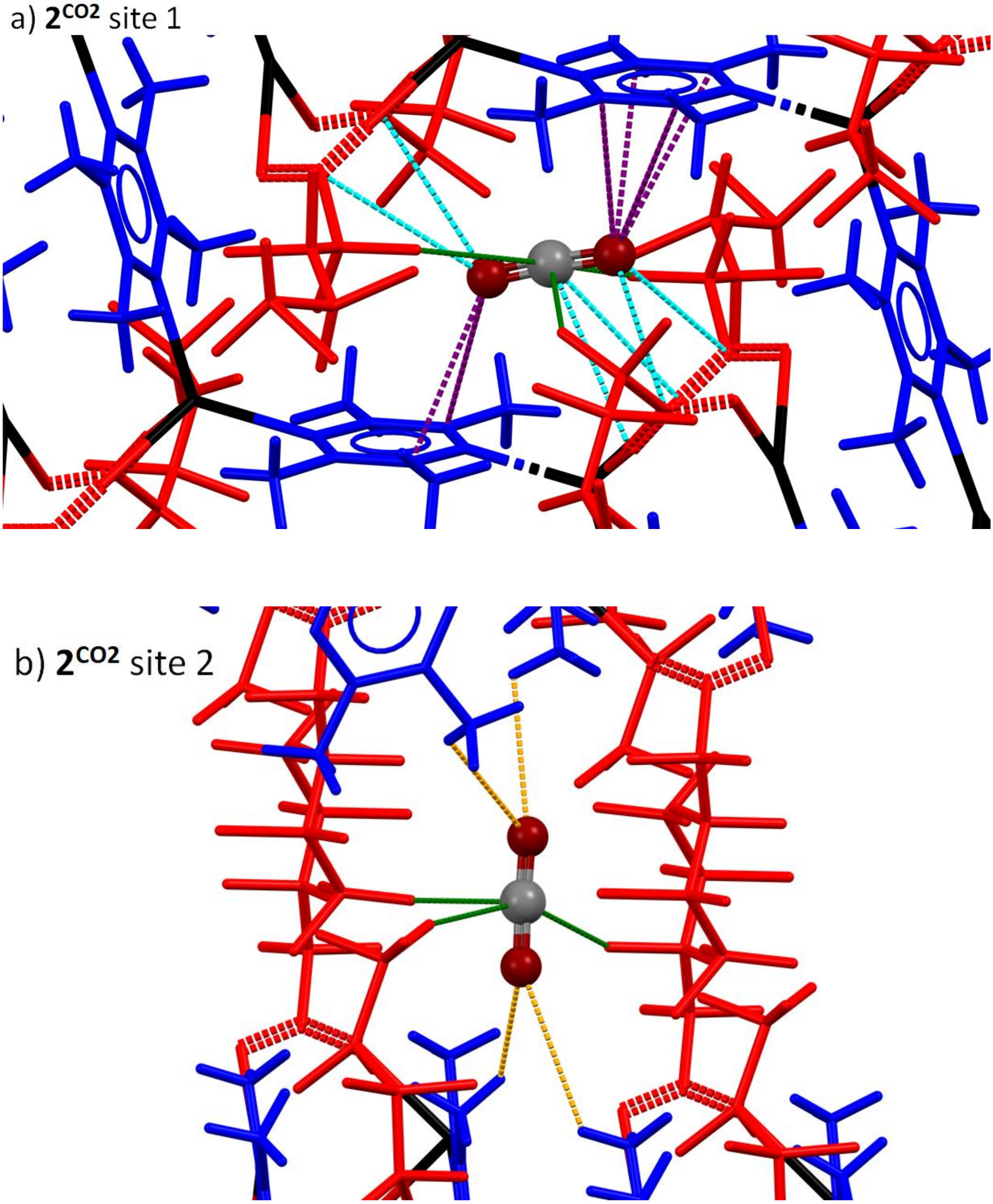

Supplementary Figure 26. Crystal structure of $\mathbf{2_A}^{\mathbf{CO2}}$ (200 K, 10bar CO_2), showing the CO_2 interaction with the coordination polymer at (a) site 1 and (b) site 2. Colour coding as in Supplementary Figure 23, $CO_2\cdots\pi$(TMP) shown as purple dashed lines, (TMP)C−H···O(CO_2) hydrogen bonds as orange dashed lines, C−F···C(CO_2) interactions as green dashed lines and (CO_2)C···O(CO_2) (both carboxylate and CO_2 gas groups) interactions in light blue.

Coordination polymer **3** (polymorph $\mathbf{3}^{\mathbf{HT}}$) was structurally characterized under vacuum at 343 K. The CO_2 pressure was increased to 10 bar and full data sets were collected at 293 K and 250 K. Crystal

structure determinations showed no substantial structural change in 3^{HT} and the associated decrease in volume (*V*/*Z*) suggests little of no CO_2 uptake The temperature was further decreased to 230 K, enabling characterization of a modified form of coordination polymer **3** (3_A^{CO2}) with 1.0(1) CO_2 molecules per formula unit alongside an increase in volume (*V*/*Z*), consistent with gated CO_2 uptake. CO_2 molecules were located in two crystallographically independent sites. In site 1 (region 1), with occupancy 0.9(1), the CO_2 molecule forms (O=C=O)$\pi\cdots\pi_{N\text{-}C}$ interactions ($O_{CO2}\cdots N_{TMP}$ 3.32(3) Å) with the single-bridge TMP ligand, multiple C−F···C(CO_2) contacts (2.73(4)-3.11(5) Å) and a weak C−H···O(CO_2) hydrogen bond with a neighbouring methyl group. A second, low-occupancy CO_2 site in region 2 (occupancy 0.1(1)) was found. The CO_2 molecule in this site interacts with its local environment via an $O{=}C{=}O^{\delta-}\cdots\pi_{N\text{-}C}$ interaction ($O_{CO2}\cdots C_{TMP}$ 2.95(4) Å) involving the single-bridge TMP ligand. The temperature was further reduced to 215 K. The X-ray crystal structure of coordination polymer 3_A^{CO2} at 215 K (Supplementary Figures 27 and 29) is isostructural with that at 230 K, but with increased CO_2 content, now $[Ag_4(O_2C(CF_2)_4CF_3)_4(TMP)_3]\cdot 1.3CO_2$. The fully occupied site contains CO_2 molecules disordered over two orientations (0.6:0.4(1) occupancies). The first orientation forms (O=C=O)$\pi\cdots\pi_{N\text{-}C}$ interactions ($O_{CO2}\cdots N_{TMP}$ 3.31(3) Å) with the single-bridge TMP ligand, C−H···O hydrogen bonds with the methyl groups and C−F···C(CO_2) contacts (2.73(4)−3.11(5) Å). The second orientation forms only multiple C−F···C(CO_2) contacts (3.18(5)−3.24(5) Å). The second CO_2 site is partially occupied (0.3(1)) by a CO_2 molecule that forms $O{=}C{=}O^{\delta-}\cdots\pi_{N\text{-}C}$ interactions ($O_{CO2}\cdots C_{TMP}$ 2.98(3) Å) with a single-bridge TMP ligand and forms multiple C−F···C(CO_2) contacts (3.10(4)−3.21(8) Å). After decreasing the temperature to 200 K a further full data set was collected at 10 bar CO_2. The resulting crystal structure is a different conformationally modified form of coordination polymer **3** (3_B^{CO2}, $[Ag_4(O_2C(CF_2)_4CF_3)_4(TMP)_3]\cdot 3.2CO_2$) with 3.2(1) CO_2 molecules per formula unit located across four crystallographically independent sites (Supplementary Figures 28 and 30). Sites 1-3 lie in region 1. Site 1 is fully occupied and the CO_2 molecule forms C−H···O(CO_2) hydrogen bonds with neighbouring methyl groups, (O=C=O)$\pi\cdots\pi_{N\text{-}C}$ interactions ($O_{CO2}\cdots N_{TMP}$ 3.49(4) Å) with the single-bridge TMP ligand, two different offset parallel dimers with the C-O atom pair of a carboxylate ligand and an adjacent CO_2 molecule (site 3), with C···O interaction distances of 3.23(3) and 3.1(1) Å, respectively. The CO_2 molecule in site 2 (occupancy 0.8(1)) forms C−H···O(CO_2) hydrogen bonds with neighbouring methyl groups, and offset parallel dimers with a C−O atom pair of an adjacent CO_2 molecule (site 3), with C···O interaction distances of 3.2(2) and 3.1(1) Å, respectively. The CO_2 molecule in site 3 (occupancy 0.8(1)) forms C−H···O(CO_2) weak hydrogen bonds with the methyl groups, multiple C−F···C(CO_2) contacts (3.07(5)−3.09(4) Å) and two different offset parallel dimers with the CO_2 molecules in sites 1 and 2. Site 4 has a CO_2 molecule (occupancy 0.5(1)) located in the (100) plane occupied by the interpenetrated perfluorocarboxylate ligands (region 2), and forms multiple C−F···C(CO_2) contacts (2.65(8)−3.12(4) Å) and parallel dimers with a symmetry-equivalent CO_2 molecule (C···O interaction distances 3.77(8) Å). The crystal structure of 3_B^{CO2} comprises two crystallographically independent but chemically identical coordination polymers. The planes formed by all carbon atoms of the perfluorocarboxylates of the tetramer ($Ag_4(CO_2(CF_2)_4CF_3)_4TMP_2$) units of the crystallographically independent coordination polymers are related by a dihedral angle of 19.5 ° (Supplementary Figure 28f). To accommodate the guest CO_2 molecules, coordination polymers 3_A^{CO2} and 3_B^{CO2} rearrange their structure relative to 3^{HT}. The dihedral angle between the equatorial (Ag_4) plane of the $Ag_4(TMP)_2$ unit and the mean plane of the single-bridge TMP ligand changes from 34.1(3) ° in coordination polymer 3^{HT} to 21.0(2) ° and 33.1(2) ° in coordination polymer 3_A^{CO2} (215 K) and 103.7(7) ° and 78.3(7) ° in coordination polymer 3_B^{CO2} (200 K). Furthermore, the dihedral angle between the equatorial planes of adjacent $Ag_4(TMP)_2$ tetramer units within each polymer change from 0 ° (*i.e.*, strictly parallel consistent with symmetry equivalence) in coordination polymer 3^{HT} to 12.38(3) ° in coordination polymer 3_A^{CO2} (215 K), but return to 0 ° in coordination polymer 3_B^{CO2} (200 K). Finally, the temperature was raised to room temperature and a full data set was collected under vacuum,

confirming the return of the crystal structure to that of coordination polymer $\mathbf{3^{HT}}$ (with a return in volume consistence with an absence of CO_2) , thereby verifying the reversibility of the gas uptake process.

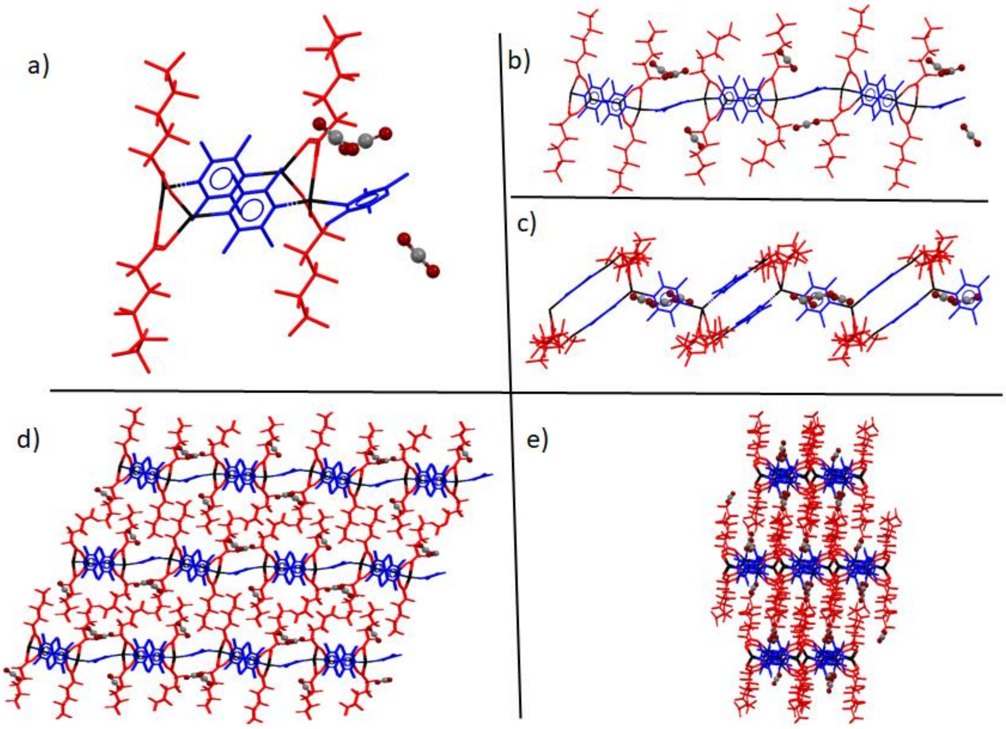

Supplementary Figure 27. Crystal structure of $\mathbf{3_A}^{CO2}$ (215 K, 10 bar CO_2), showing (a) formula unit; (b) lateral view of the 1D coordination polymer; (c) top view of the 1D coordination polymer; (d) lateral view of the packing of the 1D coordination polymers; (e) hexagonal packing motif of the 1D-coordination polymers. Colour coding as in Supplementary Figure 23.

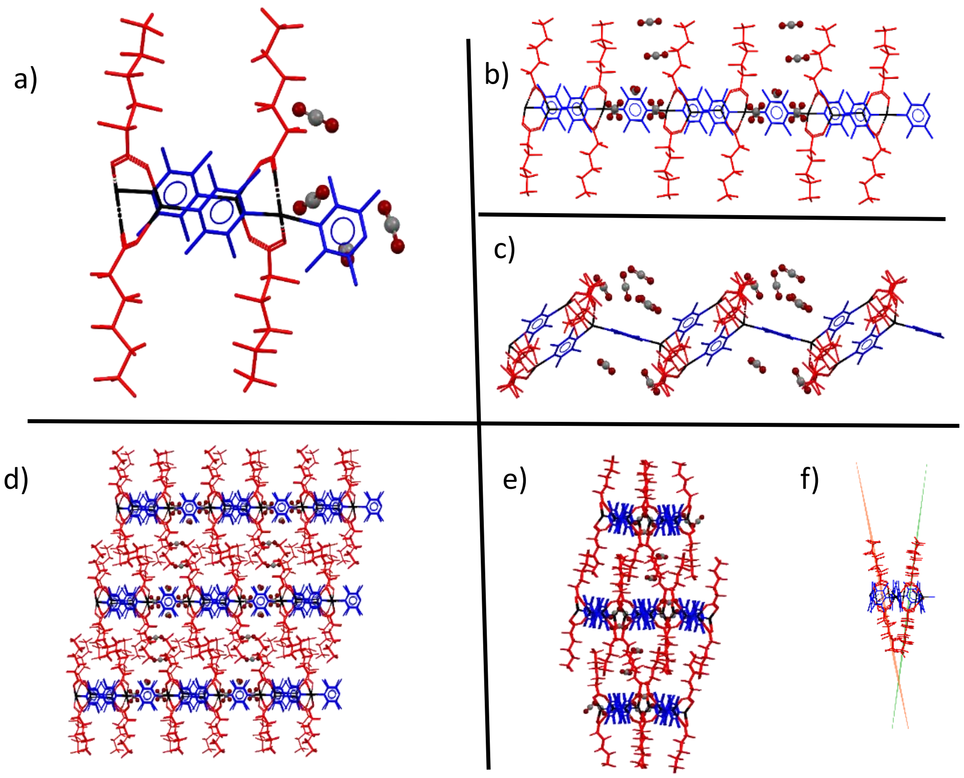

Supplementary Figure 28. Crystal structure of $\mathbf{3_B}^{CO2}$ (200 K, 10 bar CO_2), showing (a) formula unit; (b) lateral view of the 1D coordination polymer; (c) top view of the 1D coordination polymer; (d) lateral view of the packing of the 1D coordination polymers; (e) hexagonal packing motif of the 1D-coordination polymers; (f) representation of the torsion angle between crystallographically independent ($Ag_4(CO_2(CF_2)_4CF_3)_4TMP_2$) units. Colour coding as in Supplementary Figure 23.

a) $\mathbf{3_A}^{CO2}$ site 1 (disorder component 1)

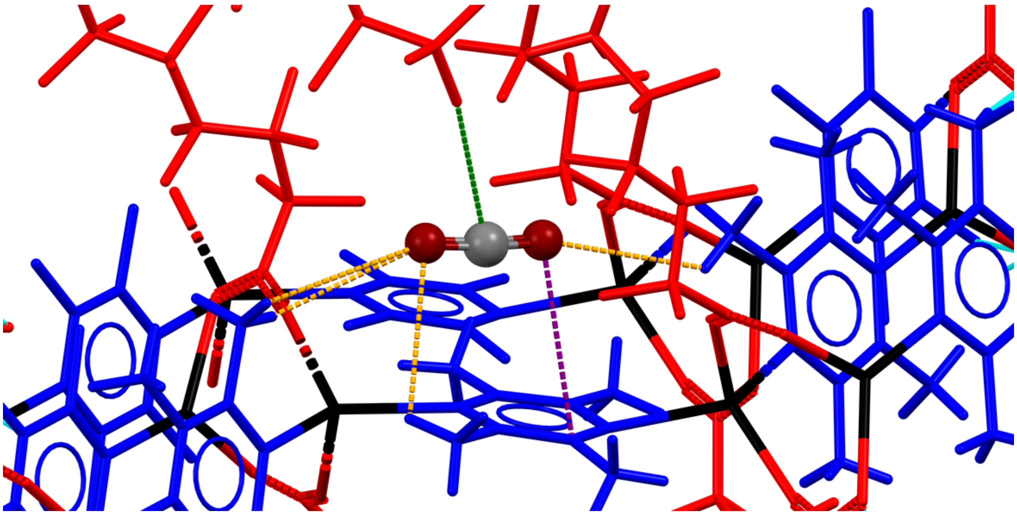

b) $\mathbf{3}_{A}^{CO2}$ site 1 (disorder component 2)

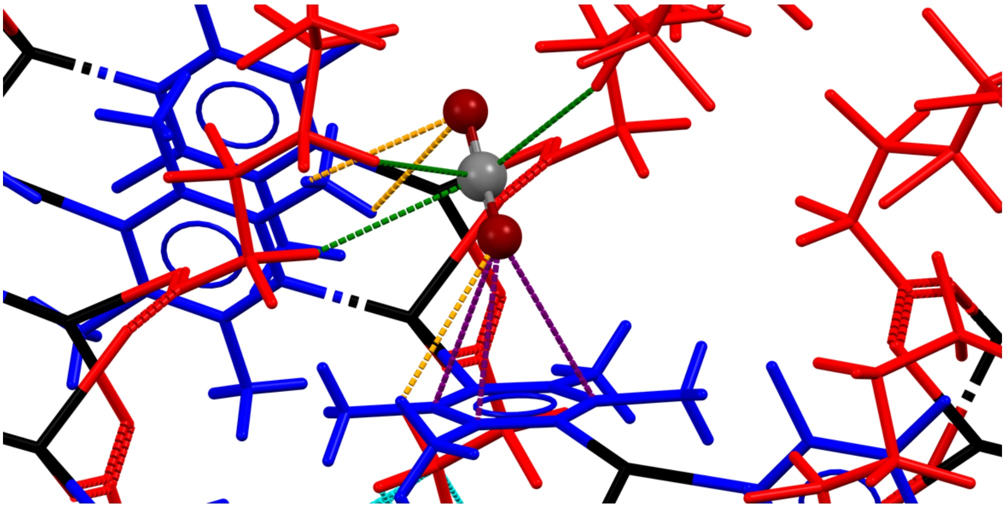

c) $\mathbf{3}_{A}^{CO2}$ site 2

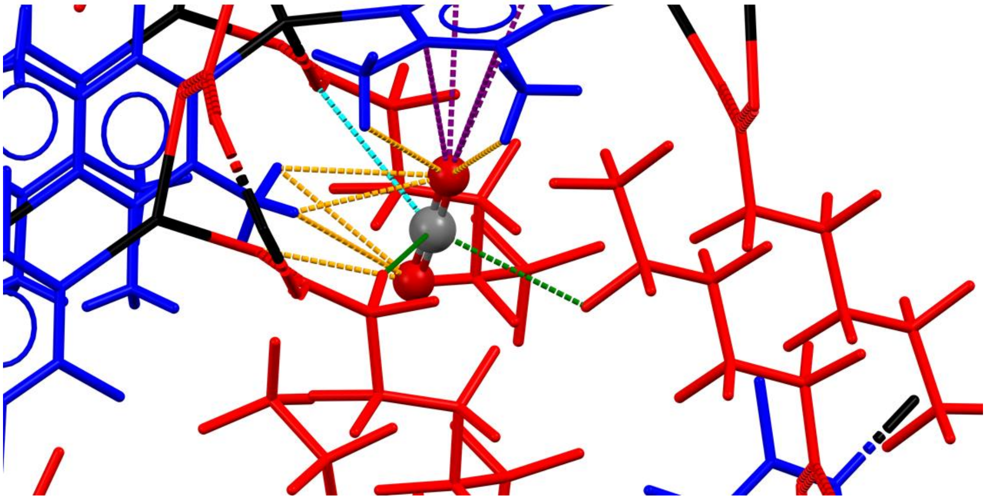

Supplementary Figure 29. Crystal structure of $\mathbf{3}_{A}^{CO2}$ (215 K, 10 bar CO_2), showing the CO_2 interaction with the coordination polymer at (a) site 1, orientation 1; (b) site 1, orientation 2; (c) site 2. Colour coding as in Supplementary Figure 23, $CO_2\cdots\pi$ (TMP) shown as purple dashed lines, (TMP)C−H···O(CO_2) hydrogen bonds as orange dashed lines, C−F···C(CO_2) interactions as green dashed lines and (CO_2)O···C(CO_2) (both carboxylate and CO_2 gas groups) interactions in light blue.

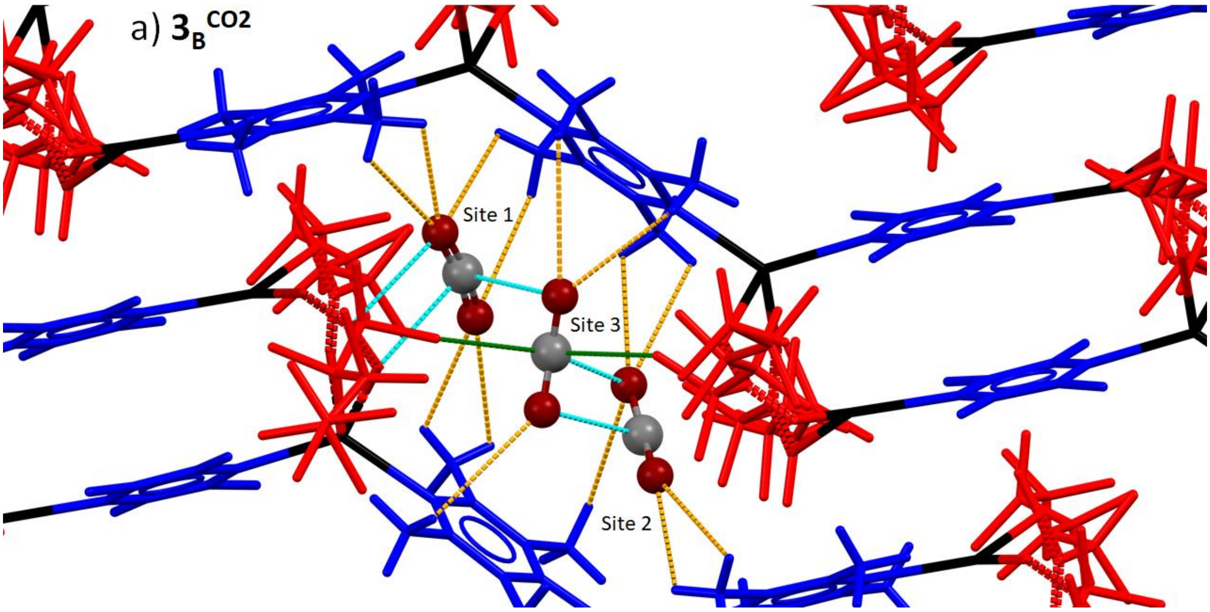

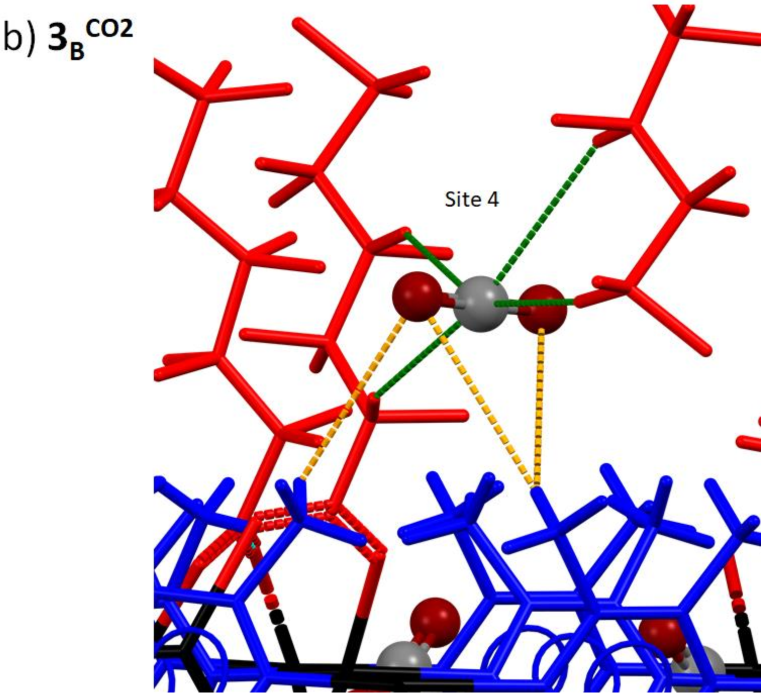

Supplementary Figure 30. Crystal structure of $\mathbf{3_B^{CO2}}$ (200 K, 10 bar CO_2) showing the CO_2 interaction with the coordination polymer at (a) sites 1-3; (b) site 4. Colour coding as in Supplementary Figure 23, $CO_2\cdots\pi$(TMP) as purple dashed lines, (TMP)C−H···O hydrogen bonds as orange dashed lines, C−F···C(CO_2) interactions as green dashed lines and (CO_2)O···C(CO_2) (both carboxylate and CO_2 gas groups) interactions in light blue.

Coordination polymer **4** was structurally characterized under vacuum at 273 K. The CO_2 pressure was increased to 10 bar and crystal structure determination from a full X-ray data set collected at 273 K established an absence of structural changes in coordination polymer **4**, with a reduction in volume (*V*/*Z*) suggesting no CO_2 uptake. The temperature was further decreased to 240 K and a new X-ray data set was collected at 10 bar CO_2 pressure, with an increase in volume (*V*/*Z*) consistent with gate opening and CO_2 uptake. The resulting crystal structure showed formation of a new form of coordination

polymer **4** (denoted **4^{CO2}**, $[Ag_4(O_2C(CF_2)_5CF_3)_4(TMP)_3]\cdot0.5CO_2$) containing 0.5(1) molecules of CO_2 per formula unit. CO_2 molecules are located at a single site (occupancy 0.5(1)) and form $O{=}C{=}O^{\delta-}\cdots\pi_{TMP}$ interactions ($O_{CO2}\cdots N_{TMP}$ 3.05(3) Å) with the single-bridge TMP ligand (region 1). The temperature was further decreased to 230 and 215 K and two data sets were collected at 10 bar CO_2 pressure. The structure of coordination polymer **4^{CO2}** remained unaffected at 230 K and 215 K, changing only in a slight, but not significant, increase in the amount of CO_2 present (0.6(1) per formula unit at 215 K). Using a new crystal of coordination polymer **4**, an X-ray data set was collected after setting the CO_2 pressure to 10 bar and reducing the temperature to 200 K. The crystal structure of coordination polymer **4^{CO2}** was retained, with a markedly increased volume (*V*/*Z*) and now with 3.4(1) molecules of CO_2 per formula unit ($[Ag_4(O_2C(CF_2)_5CF_3)_4(TMP)_3]\cdot3.4CO_2$) located across four crystallographically independent sites (Supplementary Figures 31 and 32). Site 1 is the one already occupied at higher temperatures and now contains one CO_2 molecule disordered over two orientations (54:46(3)% occupancies) in contrast to a single orientation of occupancy 0.6(1) at 215 K. The first orientation forms $(O{=}C{=}O)\pi\cdots\pi_{TMP}$ interactions ($O_{CO2}\cdots C_{TMP}$ (3.03(3) Å) with the single-bridge TMP ligand, a single C−F···C(CO_2) contact (3.60(9) Å) and multiple C−H···O(CO_2) hydrogen bonds with neighbouring methyl groups. The second orientation of the disordered CO_2 molecule in site 1 forms $O{=}C{=}O^{\delta-}\cdots\pi_{N\text{-}C}$ interactions ($O_{CO2}\cdots C_{TMP}$ 3.06(5) Å) with the single-bridge TMP ligand, multiple C−F···C(CO_2) contacts (3.15(17) - 3.50(14) Å) and multiple C−H···O(CO_2) hydrogen bonds with neighbouring methyl groups. Site 2 (in region 2) contains a CO_2 molecule with 0.6(1) occupancy forming C−F···C(CO_2) contacts (2.87(10) − 3.32(8) Å), and a distorted T-shape dimer with a CO_2 molecule in site 1 (1st orientation; (CO_2)O···C(CO_2) 3.23(7) Å). A third site was found in the (022) plane occupied by the interdigitated perfluorocarboxylate ligands (region 2) with a CO_2 molecule occupancy of 0.8(1). The CO_2 molecule forms $O{=}C{=}O^{\delta-}\cdots\pi_{N\text{-}C}$ interactions ($O_{CO2}\cdots N_{TMP}$ 3.60(4) Å) with the single-bridge TMP ligands, multiple C−F···C(CO_2) (3.03(5)−3.35(6) Å), multiple C−H···O(CO_2) hydrogen bonds with neighbouring methyl groups and a distorted T-shape dimer with the CO_2 molecule in site 4 ($C_{CO2}\cdots O_{CO2}$ 3.23(7) Å). Site 4 was also found in the (022) plane occupied by the interdigitated perfluorocarboxylate ligands (region 2) and has a CO_2 molecule with an occupancy of 0.9(1), which forms multiple C−F···C(CO_2) (3.14(16) − 3.27(7) Å), multiple C−H···O(CO_2) hydrogen bonds with neighbouring methyl groups and distorted T-shape dimer with the CO_2 molecule in site 3. To accommodate the guest CO_2 molecules, the coordination polymers in **4^{CO2}** rearrange their structure relative to **4**. The dihedral angle between the Ag_4 equatorial plane of the $Ag_4(TMP)_2$ units and the mean plane formed by the single-bridge TMP units changes from 37.2(2) ° in coordination polymer **4** to 39.4(3) ° and 29.8(3) ° in **4^{CO2}** at 215 K, and 1.5(6)° and 26.1(5) ° in **4^{CO2}** at 200 K. Furthermore, the dihedral angle between the equatorial planes of adjacent $Ag_4(TMP)_2$ units within each individual coordination polymer changes from 0° (*i.e.*, strictly parallel, consistent with being symmetry equivalent) in coordination polymer **4** to 13.07(2) ° in coordination polymer **4^{CO2}** at 215 K and 24.72(6) ° in coordination polymer **4^{CO2}** at 200 K.

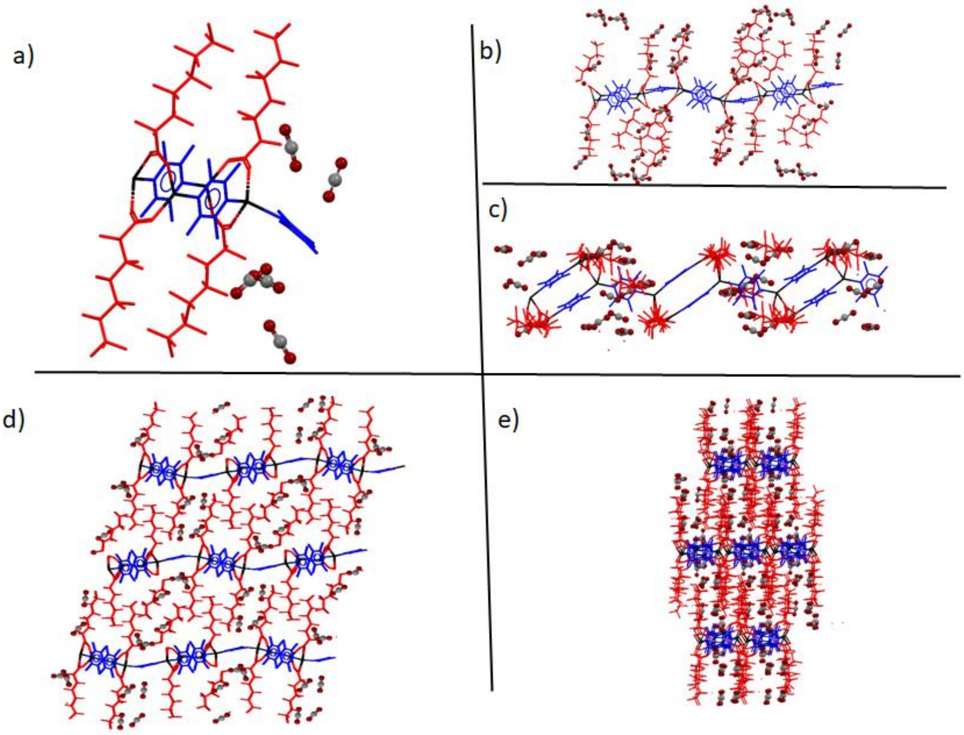

Supplementary Figure 31. Crystal structure of $\mathbf{4}^{CO2}$ (200 K, 10 bar CO_2) showing (a) formula unit; (b) lateral view of the 1D coordination polymer; (c) top view of the 1D coordination polymer; (d) lateral view of the packing of the 1D coordination polymers; (e) hexagonal packing motif of the 1D-coordination polymers. Colour coding as in Supplementary Figure 23.

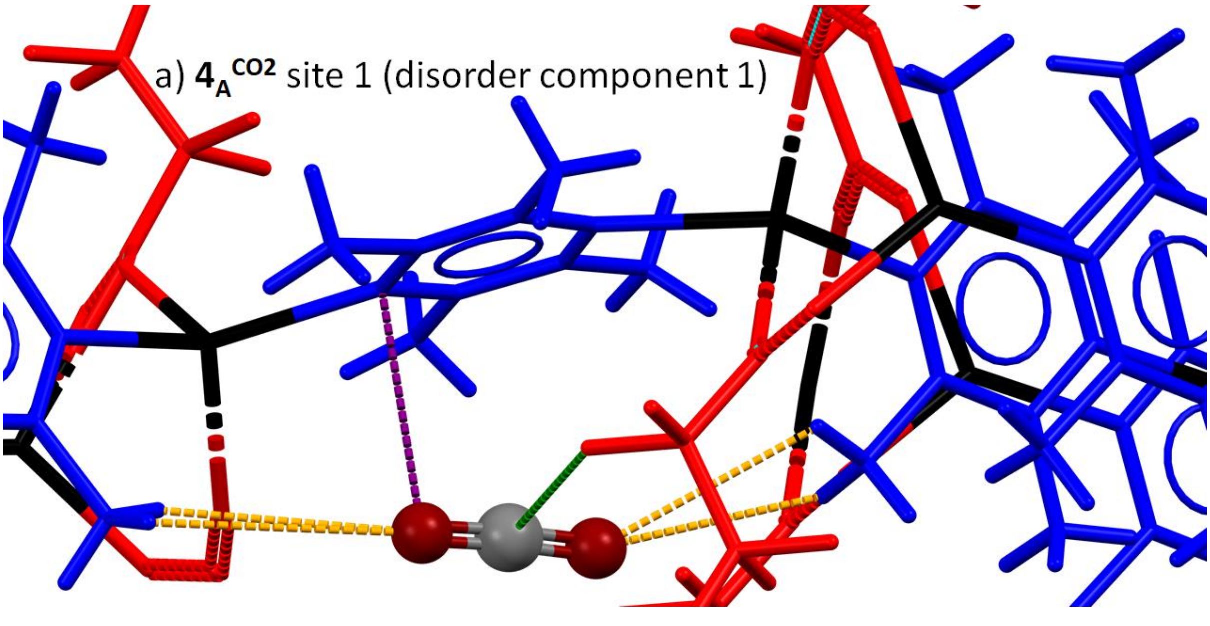

b) **4_A**^{CO2} site 1 (disorder component 2)

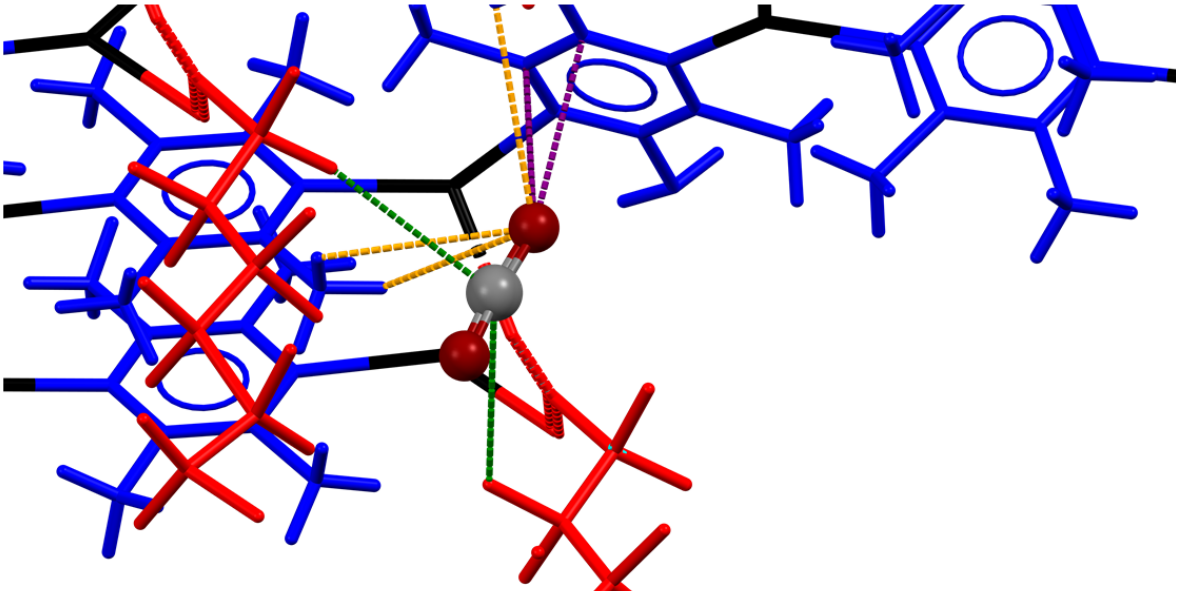

c) **4_A**^{CO2} site 2

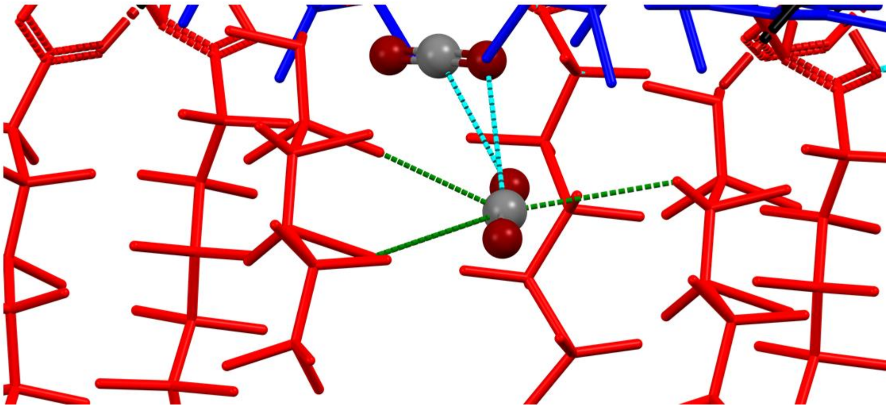

d) **4_A**^{CO2} site 3 and 4

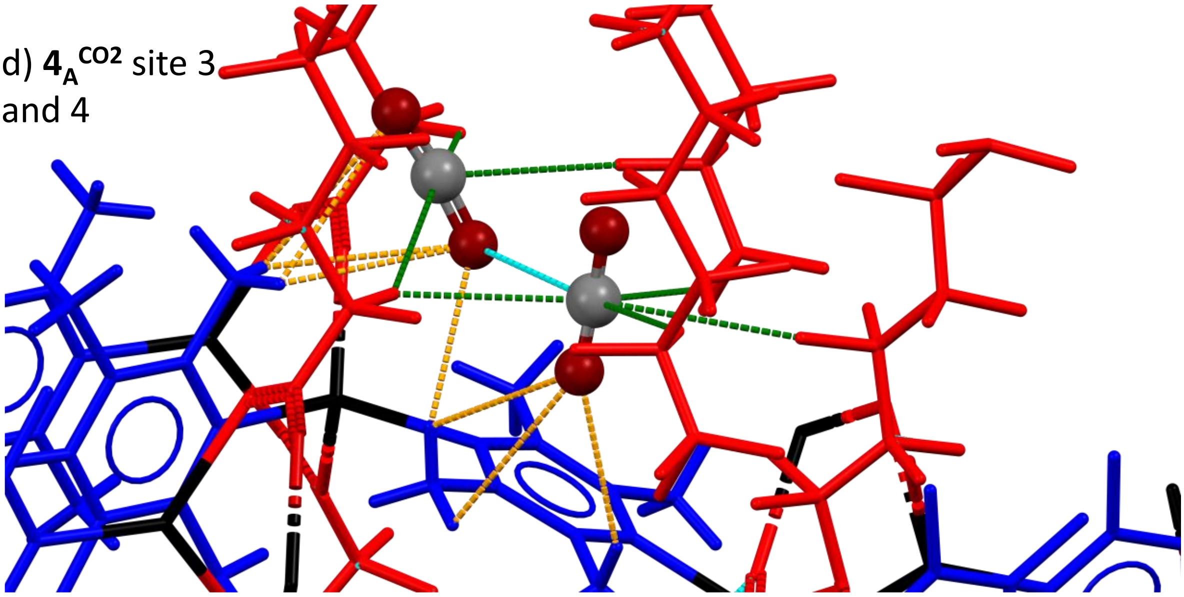

Supplementary Figure 32. Crystal structure of $\mathbf{4^{CO2}}$ (200 K, 10 bar CO_2) showing the CO_2 interaction with the coordination polymer at (a) site 1, orientation 1; (b) site 2, orientation 2; (c) site 2 and (d) site 3 and 4. Colour coding as in Supplementary Figure 23, $CO_2\cdots\pi$(TMP) as purple dashed lines, (TMP)C−H···O(CO_2) hydrogen bonds as orange dashed lines, C−F···C(CO_2) interactions as green dashed lines and (CO_2)C···O(CO_2) (both carboxylate and CO_2 gas groups) interactions in light blue.

Coordination polymer **5** was structurally characterized under vacuum at 298 K. The CO_2 pressure was increased to 10 bar and crystal structure determination from a full data set collected at 298 K established an absence of structural changes in coordination polymer **5**. The temperature was subsequently decreased to 250 and 230 K and crystal structure determinations at both temperatures showed no substantial structural changes. Further reduction in temperature to 200 K and collection of a full data set at 10 bar CO_2 pressure, indicated of a new form of coordination polymer **5** (subsequently denoted $\mathbf{5^{CO2}}$) based initially on determination of unit cell parameters that indicated an approximate unit cell volume doubling, although a reduction in *V*/*Z*. Data were then collected on a new crystal of coordination polymer **5** at 10 bar CO_2 pressure and 200 K, confirming the formation of coordination polymer $\mathbf{5^{CO2}}$ and enabling crystal structure determination, which identified 0.8(1) CO_2 molecules per formula unit. The CO_2 molecules lie in a single site (region 1) which forms (O=C=O)$\pi\cdots\pi_{N\text{-}C}$ interactions ($O_{CO2}\cdots C_{TMP}$ 3.03(2) Å), multiple C−H···O(CO_2) hydrogen bonds with neighbouring methyl groups with the single-bridge TMP ligand, and multiple C−F···C(CO_2) contacts (2.92(5)−3.05(6) Å). The coordination polymers in $\mathbf{5^{CO2}}$ are shown to have rearranged their structure to accommodate the guest CO_2 molecule. The dihedral angle between the equatorial (Ag_4) plane of the $Ag_4(TMP)_2$ tetramers and the mean plane of the single-bridge TMP ligands changed from 36.7(2) ° in coordination polymer **5** to 38.9(3) ° and 29.4(3) ° in coordination polymer $\mathbf{5^{CO2}}$ (200 K, 10 bar CO_2). Furthermore, the dihedral angle between the equatorial planes of the $Ag_4(TMP)_2$ tetramer units changed from 0 ° in coordination polymer **5** to 10.79(2) ° in coordination polymer $\mathbf{5^{CO2}}$ (200 K, 10 bar CO_2).

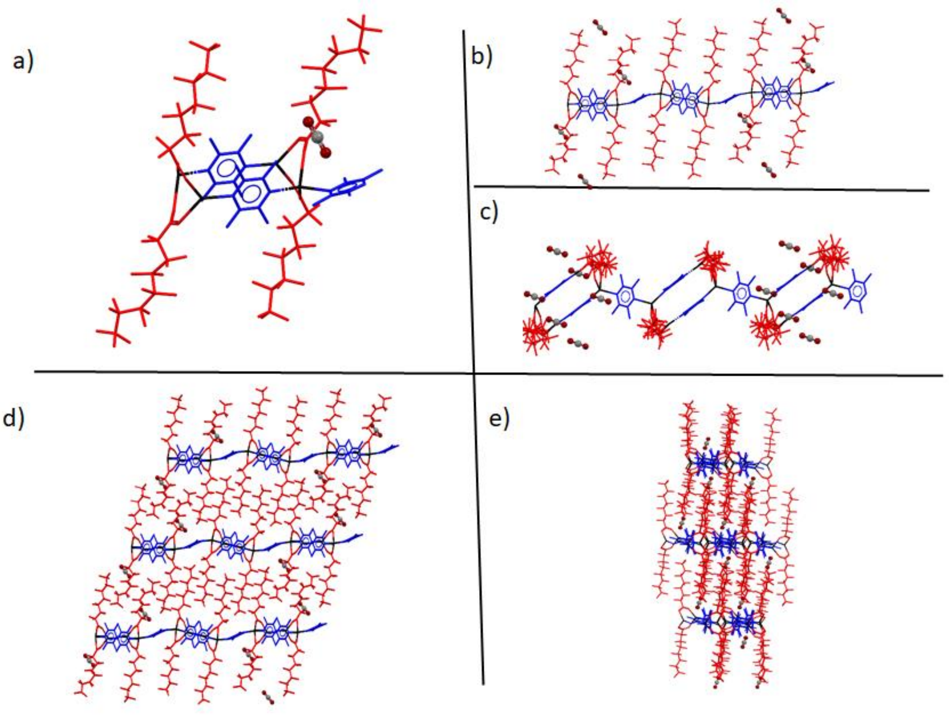

Supplementary Figure 33. Crystal structure of $\mathbf{5^{CO2}}$ (200 K, 10 bar CO_2) showing (a) formula unit; (b) lateral view of the 1D coordination polymer; (c) top view of the 1D coordination polymer; (d) lateral view of the packing of the 1D coordination polymers; (e) hexagonal packing motif of the 1D-coordination polymers. Colour coding as in Supplementary Figure 23.

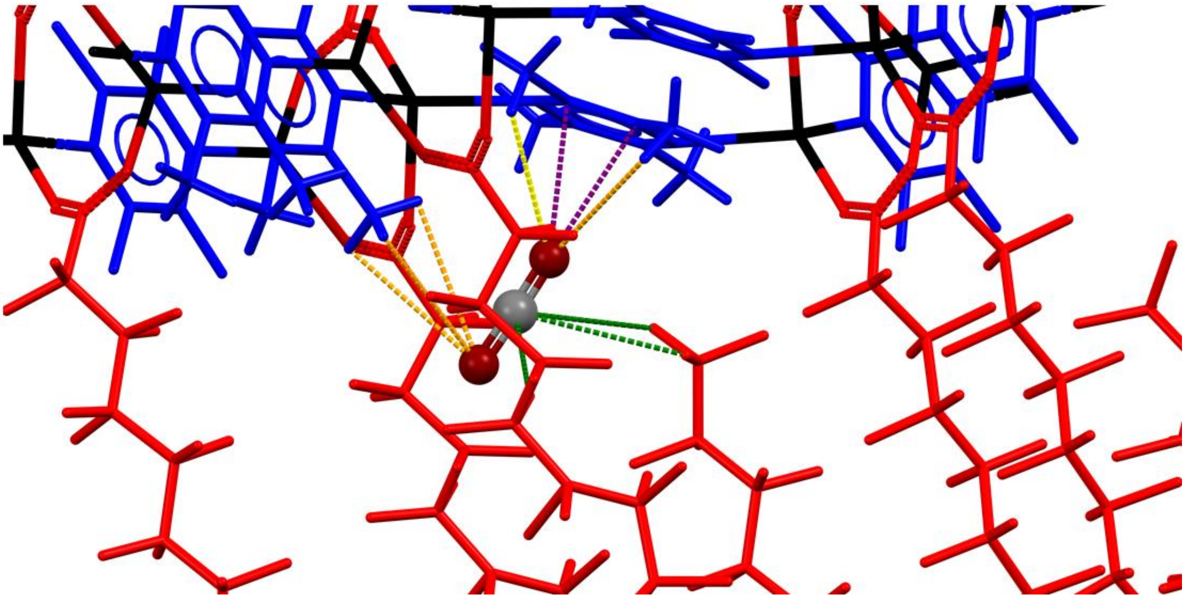

Supplementary Figure 34. Crystal structure of $\mathbf{5^{CO2}}$ (200 K, 10 bar CO_2) showing the CO_2 interaction with the coordination polymer at a single site. Colour code as in Supplementary Figure 23, $CO_2\cdots\pi$(TMP) as purple dashed lines, (TMP)C−H···O hydrogen bonds as orange dashed lines, C−F···C(CO_2) interactions as green dashed lines and (CO_2)O···C(CO_2) (both carboxylate and CO_2 gas groups) interactions in light blue.

4. *In situ* powder X-ray diffraction studies of coordination polymers 1, 2, 4 and 5 under exposure to CO_2 and CH_4 gas pressure

The CO_2 gas sorption by compounds **1**, **2**, **4** and **5**, and CH_4 gas sorption by compounds **1** and **5** were monitored *in situ* by powder X-ray diffraction at beamline ID31 at the European Synchrotron Radiation Source (ESRF) using a 9-channel multi-analyser crystal (MAC) detector and at beamline I11 at the Diamond Light Source using a wide-angle (90 °) position-sensitive detector (PSD) comprising 18 Mythen-2 modules.[S1,S8,S9] For data collected using the PSD, a pair of scans related by a 0.25 ° detector offset were collected for each measurement to account for gaps between detector modules. All data were collected at room temperature for studies of CO_2 adsorption, whereas for CH_4 adsorption data were collected at both room temperature and 180 K. A sample of colourless microcrystalline **1**, **2**, **4** and **5**, synthesised as previously described, was lightly ground using an agate pestle and mortar and loaded into a 0.8 mm quartz capillary. The open end of the capillary was attached via tightened Swagelok® compression fittings to the stainless steel tubing of a gas handling rig that was further connected via a series of taps to a lecture bottle of CO_2 or CH_4 gas, a turbomolecular pump and an exhaust vent. The rig including the capillary was first evacuated taking care not to disturb the powder in the capillary.

4.1 *In situ* PXRD studies of coordination polymer 1 exposure to CO_2 and CH_4 gas pressure

The structural changes associated with CO_2 and CH_4 absorption were explored for coordination polymer **1** using PXRD at ESRF.[S1,S8] Scans were collected ($-2.5 \leq 2\theta \leq 12.5$ °) at a scan speed of 6 ° min^{-1} during which the capillary was oscillated about its axis through an angle of ca. 150 °. One scan was collected at room temperature (295 K) at each of a series of gas pressures in two separate studies (CO_2: 0, 1.01, 4.21, 9.71, 19.71, 50.23, 19.51, 10.05, 4.31 and 1.13 bar; CH_4: 0, 19.71, 42.00, 18.35, 8.81,

4.31, 1.13 bar), with 30 min intervals between measurements for equilibration of the structure along the capillary at each pressure. In the second study (*i.e.* CH_4 atmosphere), the temperature was subsequently reduced to 180 K and PXRD patterns were obtained at CH_4 pressures of 2, 5 and 25 bar. In all cases, the powder pattern was indexed using the *TOPAS* program.[S10] A three-phase refinement was implemented in *TOPAS*, which comprised a Pawley refinement,[S11] using the unit cell parameters of structure of **1** (high-temperature polymorph B, $\mathbf{1_B}^{\mathbf{HT}}$) or $\mathbf{1_B}^{\mathbf{CO2}}$ (depending on the CO_2 gas pressure), and a Rietveld refinement[S12] for the minor impurity phases $[Ag_4(O_2C(CF_2)_2CF_3)_4(TMP)_2]$[S13] and $[Ag(O_2C(CF_2)_2CF_3)(TMP)]$,[S3] using the crystal structures of these compounds as starting models. The model for each structure was refined with a different global isotropic thermal parameter. Unit cell parameters at each CO_2 gas pressure are provided in Supplementary Table 12 and summarized in Supplementary Figure 35. Fits for individual PXRD patterns are provided in Supplementary Figures 36-45. Unit cell parameters at each CH_4 gas pressure measured at room temperature and 180 K are provided in Supplementary Tables 13 and 14, respectively. Fits for individual PXRD patterns are provided in Supplementary Figures 46-52 and 53-55, respectively.

4.1.1 *In situ* PXRD study of coordination polymer 1 exposure to CO_2 gas under pressure

An initial scan on coordination polymer **1** was collected under vacuum and room temperature. Pawley refinement confirmed the presence of coordination polymer **1** (as polymorph $\mathbf{1_B}^{\mathbf{HT}}$, in contrast to $\mathbf{1_A}^{\mathbf{HT}}$ found in SCXRD studies, section 3.2), while traces of 2D coordination polymer $[Ag_4(CO_2(CF_2)_2CF_3)_4(TMP)_2]$ and 1D coordination polymer $[Ag(CO_2(CF_2)_2CF_3)(TMP)]$ were also identified and fitted by Rietveld refinement. Coordination polymers $[Ag_4(CO_2(CF_2)_2CF_3)_4(TMP)_2]$ and $[Ag(CO_2(CF_2)_2CF_3)(TMP)]$ remained unaltered through the entire gas uptake process. The CO_2 pressure was increased to 1.01 bar and subsequently 4.21 bar and a new scan was collected at room temperature at each pressure, with Pawley of the peaks associated with **1** indicating no structural changes in $\mathbf{1_B}^{\mathbf{HT}}$. The CO_2 pressure was further increased to 9.79 bar and a new scan was collected at room temperature. Pawley refinement confirmed the presence of a new form of the gas-containing coordination polymer **1** ($\mathbf{1_B}^{\mathbf{CO2}}$). Unit cell values of coordination polymer $\mathbf{1_B}^{\mathbf{CO2}}$ (Supplementary Table 12) are analogous to the $\mathbf{1_B}^{\mathbf{LT}}$ polymorph,[S3] but with an increase of approx. 2 Å in the *a*- axis, 1 Å in the *c*-axis and 5 ° in the β angle. As a consequence, the unit cell volume of $\mathbf{1_B}^{\mathbf{CO2}}$ is 600 $Å^3$ larger than that of $\mathbf{1_B}^{\mathbf{LT}}$. The structure of $\mathbf{1_B}^{\mathbf{CO2}}$ was not obtained due to insufficient resolution in the patterns and the overlap of reflections caused by the presence of the minor impurity phases. The CO_2 pressure was further increased to 19.7 and 50.2 bar and PXRD scans were collected at room temperature, showing a slight increase in the unit cell volume ($\Delta V \approx 30$ and 78 $Å^3$, respectively) of $\mathbf{1_B}^{\mathbf{CO2}}$ relative to that at p_{CO_2} = 9.79 bar. The reversibility of the process was investigated by decreasing the CO_2 pressure at room temperature. Pawley refinement of scans collected at 19.5 and 10.0 bar showed the presence of $\mathbf{1_B}^{\mathbf{CO2}}$ coordination polymer. On reducing the CO_2 pressure to 4.3 and 1.1 bar, Pawley refinement showed the presence of the $\mathbf{1_B}^{\mathbf{HT}}$ polymorph, confirming the release of the CO_2 guest molecules and the reversibility of the gas uptake process.

Supplementary Table 12. Unit cell parameters from Pawley refinement for compound **1** under CO_2 gas pressure at room temperature

Pressure (bar)	*Compound (Z)*	*a (Å)*	*b (Å)*	*c (Å)*	*α (°)*	*β (°)*	*γ (°)*	*V (Å³)*	*V/Z (Å³)*
10^{-6}	$\mathbf{1_B}^{HT}$(2)	22.6861(7)	8.5473(3)	15.0623(7)	90	100.702(3)	90	2869.8(2)	1434.9(2)
1.01	$\mathbf{1_B}^{HT}$(2)	22.6847(7)	8.5457(4)	15.0585(7)	90	100.682(3)	90	2868.6(3)	1434.3(3)
4.21	$\mathbf{1_B}^{HT}$(2)	22.7021(9)	8.5470(4)	15.0718(9)	90	100.644 (4)	90	2874.1(3)	1437.0(3)
9.79	$\mathbf{1_B}^{CO2}$(4)	25.646(2)	8.5683(6)	30.269(2)	90	116.160(4)	90	5970.2(8)	1492.5(8)
19.71	$\mathbf{1_B}^{CO2}$(4)	25.711(3)	8.5732(8)	30.289(2)	90	115.997(7)	90	6001(1)	1500(1)
50.23	$\mathbf{1_B}^{CO2}$(4)	25.819(1)	8.5785(1)	30.321(2)	90	115.759(4)	90	6048.5(7)	1512.1(7)
19.51	$\mathbf{1_B}^{CO2}$(4)	25.711(2)	8.5724(6)	30.283(2)	90	116.009(5)	90	5998.8(1)	1499.7(1)
10.05	$\mathbf{1_B}^{CO2}$(4)	25.657(1)	8.5684(6)	30.265(1)	90	116.186(4)	90	5970.7(7)	1492.6(7)
4.31	$\mathbf{1_B}^{HT}$(2)	22.6960(9)	8.5432(4)	15.0689(9)	90	100.632(4)	90	2871.6(7)	1435.8(7)
1.13	$\mathbf{1_B}^{HT}$(2)	22.6792(8)	8.5421(4)	15.0586(9)	90	100.671(4)	90	2866.8(7)	1433.4(7)

Overall, the *in-situ* powder X-ray diffraction study shows $\mathbf{1_B}^{HT} \rightarrow \mathbf{1_B}^{CO2}$ transition between 4-10 bar CO_2 pressure (Supplementary Table 12, Supplementary Figure 35). The unit cell volume of $\mathbf{1_B}^{CO2}$ is more than twice that of the initial phase $\mathbf{1_B}^{HT}$ as the asymmetric unit of $\mathbf{1_B}^{CO2}$ contains twice as much of the polymer as that of $\mathbf{1_B}^{HT}$. The expansion due to CO_2 adsorption into the crystals is more effectively quantified by considering the change in volume per formula unit, $\Delta V/Z = 55.5$ Å^3 from $p_{CO2} =$ 4.21 bar to $p_{CO2} = 9.79$ bar and $\Delta V/Z = 19.6$ Å^3 from $p_{CO2} =$ 9.79 bar to $p_{CO2} = 50.23$ bar.

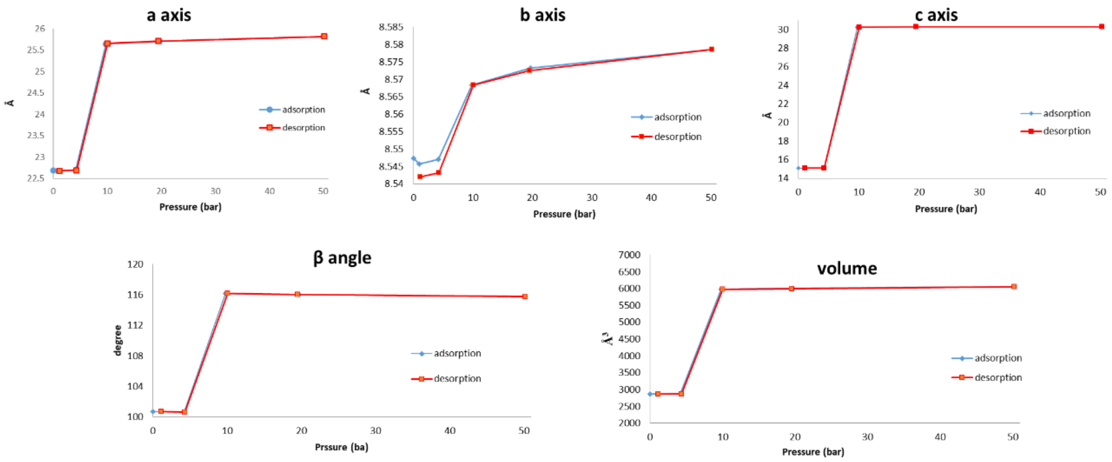

Supplementary Figure 35. Unit cell values from Pawley refinement of PXRD patterns for coordination polymer **1** ($\mathbf{1_B}^{HT}$ at pressures up to 4.5 bar and $\mathbf{1_B}^{CO2}$ at pressures above 9 bar) under CO_2 gas at pressures of 0, 1.05, 4.21, 9.79, 19.71, 50.29, 19.51, 10.05, 4.31 and 1.13 bar at room temperature. Note that there is a reduction in symmetry at $p_{CO2} = 9.79$ bar that results in doubling of *Z*. Changes in volume expressed as changes in *V*/*Z* are provided in Supplementary Table 12.

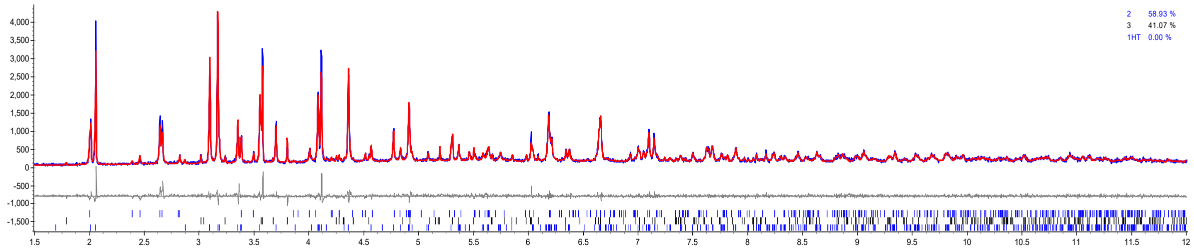

Supplementary Figure 36. Observed (blue) and calculated (red) profiles and difference plot [I_{obs}–I_{calc}] (grey) of the Pawley refinement of Pattern 1 at 10^{-6} mbar ($1.5 \le 2\theta \le 12.0$ °; d_{min} = 1.91 Å) (R_{wp} = 11.632, R_{wp}' = 21.738) for $\mathbf{1_B}^{HT}$. Minor-phase impurities of [$Ag_4(O_2C(CF_2)_2CF_3)_4(TMP)_2$] and [$Ag(O_2C(CF_2)_2CF_3)(TMP)$] that remain constant throughout the study are fitted by Rietveld methods.

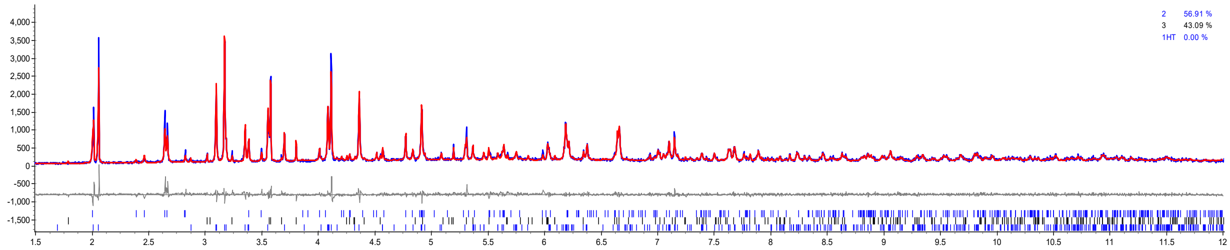

Supplementary Figure 37. Observed (blue) and calculated (red) profiles and difference plot [I_{obs}–I_{calc}] (grey) of the Pawley refinement of Pattern 2 at 1.01 bar CO_2 ($1.5 \le 2\theta \le 12.0$ °; d_{min} = 1.91 Å) (R_{wp} = 12.960, R_{wp}' = 25.043) for $\mathbf{1_B}^{HT}$. Minor-phase impurities of [$Ag_4(O_2C(CF_2)_2CF_3)_4(TMP)_2$] and [$Ag(O_2C(CF_2)_2CF_3)(TMP)$] that remain constant throughout the study are fitted by Rietveld methods.

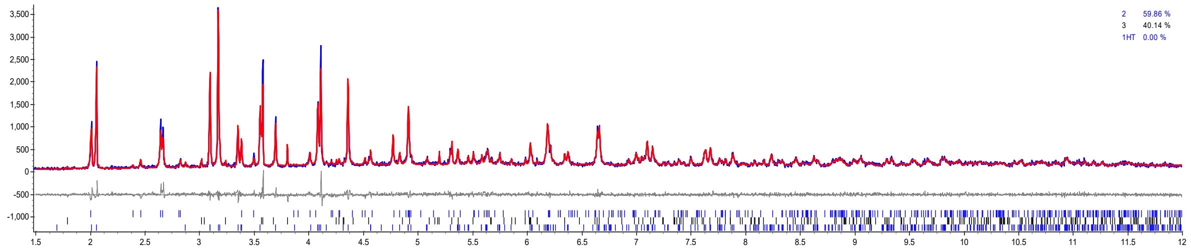

Supplementary Figure 38. Observed (blue) and calculated (red) profiles and difference plot [I_{obs}–I_{calc}] (grey) of the Pawley refinement of Pattern 3 at 4.21 bar CO_2 ($1.5 \le 2\theta \le 12.0$ °; d_{min} = 1.91 Å) (R_{wp} = 11.877, R_{wp}' = 23.290) for $\mathbf{1_B}^{HT}$. Minor-phase impurities of [$Ag_4(O_2C(CF_2)_2CF_3)_4(TMP)_2$] and [$Ag(O_2C(CF_2)_2CF_3)(TMP)$] that remain constant throughout the study are fitted by Rietveld methods.

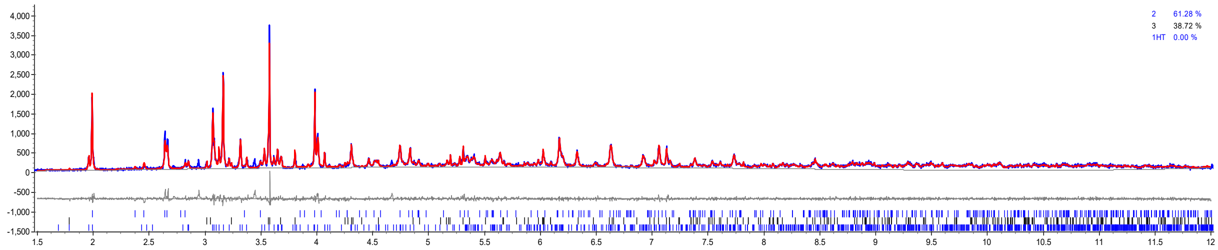

Supplementary Figure 39. Observed (blue) and calculated (red) profiles and difference plot [I_{obs}–I_{calc}] (grey) of the Pawley refinement of Pattern 4 at 9.79 bar CO_2 ($1.5 \le 2\theta \le 12.0$ °; d_{min} = 1.91 Å) (R_{wp} = 12.228, R_{wp}' = 22.848) for $\mathbf{1_B}^{CO2}$. Minor-phase impurities of [$Ag_4(O_2C(CF_2)_2CF_3)_4(TMP)_2$] and [$Ag(O_2C(CF_2)_2CF_3)(TMP)$] that remain constant throughout the study are fitted by Rietveld methods.

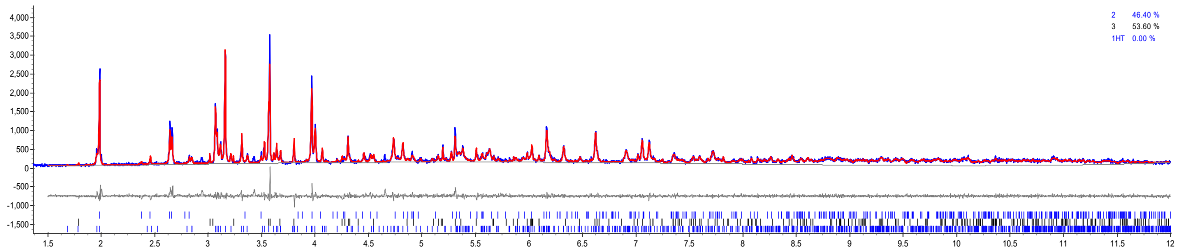

Supplementary Figure 40. Observed (blue) and calculated (red) profiles and difference plot [I_{obs}–I_{calc}] (grey) of the Pawley refinement of Pattern 5 at 19.71 bar CO_2 ($1.5 \leq 2\theta \leq 12.0$ °; d_{min} = 1.91Å) (R_{wp} = 12.843, R_{wp}' = 23.368) for $\mathbf{1}_{\mathbf{B}}^{\mathbf{CO2}}$. Minor-phase impurities of [$Ag_4(O_2C(CF_2)_2CF_3)_4(TMP)_2$] and [$Ag(O_2C(CF_2)_2CF_3)(TMP)$] that remain constant throughout the study are fitted by Rietveld methods.

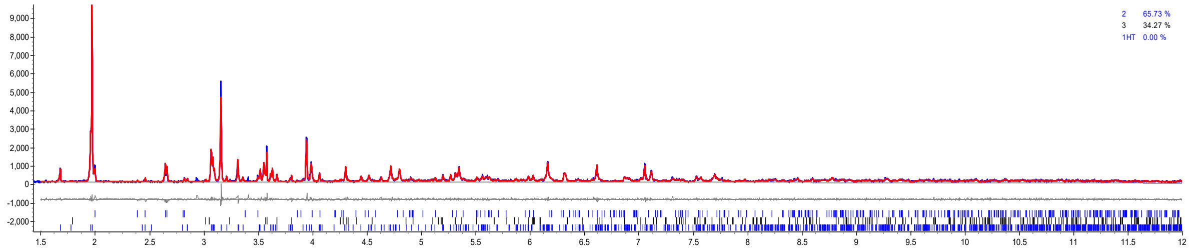

Supplementary Figure 41. Observed (blue) and calculated (red) profiles and difference plot [I_{obs}–I_{calc}] (grey) of the Pawley refinement of Pattern 6 at 50.23 bar CO_2 ($1.5 \leq 2\theta \leq 12.0$ °; d_{min} = 1.91 Å) (R_{wp} = 12.062, R_{wp}' = 22.500) for $\mathbf{1}_{\mathbf{B}}^{\mathbf{CO2}}$. Minor-phase impurities of [$Ag_4(O_2C(CF_2)_2CF_3)_4(TMP)_2$] and [$Ag(O_2C(CF_2)_2CF_3)(TMP)$] that remain constant throughout the study are fitted by Rietveld methods.

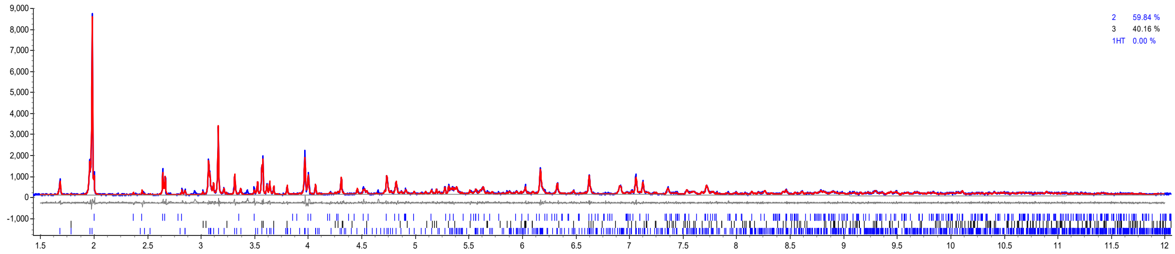

Supplementary Figure 42. Observed (blue) and calculated (red) profiles and difference plot [I_{obs}–I_{calc}] (grey) of the Pawley refinement of Pattern 7 at 19.51 bar CO_2 ($1.5 \leq 2\theta \leq 12.0$ °; d_{min} = 1.91 Å) (R_{wp} = 13.010 , R_{wp}' = 23.757) for $\mathbf{1}_{\mathbf{B}}^{\mathbf{CO2}}$. Minor-phase impurities of [$Ag_4(O_2C(CF_2)_2CF_3)_4(TMP)_2$] and [$Ag(O_2C(CF_2)_2CF_3)(TMP)$] that remain constant throughout the study are fitted by Rietveld methods.

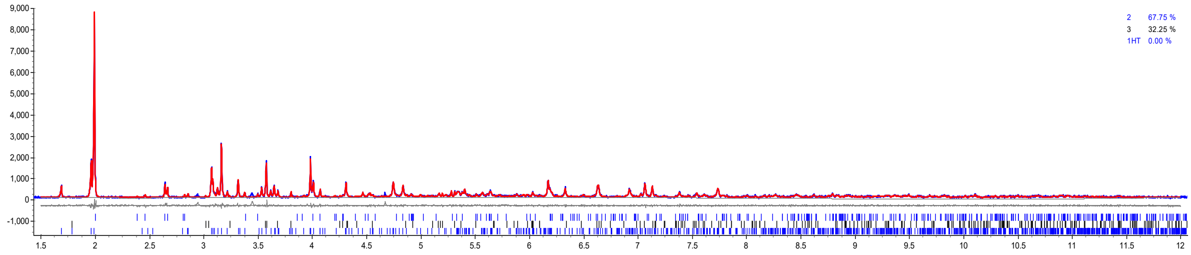

Supplementary Figure 43. Observed (blue) and calculated (red) profiles and difference plot [I_{obs}–I_{calc}] (grey) of the Pawley refinement of Pattern 8 at 10.05 bar CO_2 ($1.5 \leq 2\theta \leq 12.0$ °; d_{min} = 1.91 Å) (R_{wp} = 12.534, R_{wp}' = 22.853) for $\mathbf{1}_{\mathbf{B}}^{\mathbf{CO2}}$. Minor-phase impurities of [$Ag_4(O_2C(CF_2)_2CF_3)_4(TMP)_2$] and [$Ag(O_2C(CF_2)_2CF_3)(TMP)$] that remain constant throughout the study are fitted by Rietveld methods.

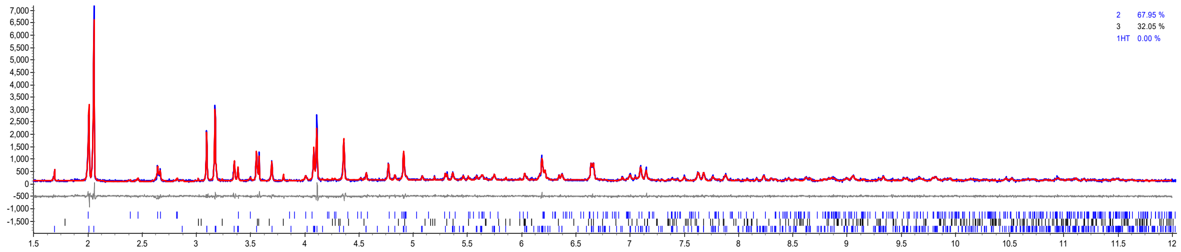

Supplementary Figure 44. Observed (blue) and calculated (red) profiles and difference plot [I_{obs}–I_{calc}] (grey) of the Pawley refinement of Pattern 9 at 4.31 bar CO_2 ($1.5 \leq 2\theta \leq 12.0$ °; d_{min} = 1.91 Å) (R_{wp} = 13.324, R_{wp}' = 25.594) for $\mathbf{1_B}^{\mathbf{HT}}$. Minor-phase impurities of [$Ag_4(O_2C(CF_2)_2CF_3)_4(TMP)_2$] and [$Ag(O_2C(CF_2)_2CF_3)(TMP)$] that remain constant throughout the study are fitted by Rietveld methods.

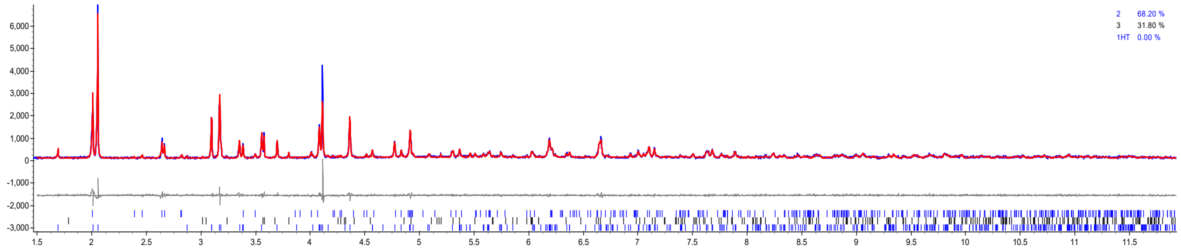

Supplementary Figure 45. Observed (blue) and calculated (red) profiles and difference plot [I_{obs}–I_{calc}] (grey) of the Pawley refinement of Pattern 10 at 1.13 bar CO_2 ($1.5 \leq 2\theta \leq 12.0$ °; d_{min} = 1.91 Å) (R_{wp} = 13.715, R_{wp}' = 25.857) for $\mathbf{1_B}^{\mathbf{HT}}$. Minor-phase impurities of [$Ag_4(O_2C(CF_2)_2CF_3)_4(TMP)_2$] and [$Ag(O_2C(CF_2)_2CF_3)(TMP)$] that remain constant throughout the study are fitted by Rietveld methods.

4.1.2 *In situ* PXRD study of coordination polymer 1 exposure to CH_4 gas under pressure

In a second experiment, CH_4 adsorption was studied for coordination polymer **1** using powder X-ray diffraction. An initial scan was collected under vacuum and room temperature. Pawley refinement confirmed the presence of coordination polymer **1** (polymorph $\mathbf{1_B}^{\mathbf{HT}}$, consistent with initial state prior to *in situ* PXRD study during CO_2 adsorption, section 4.1.1), while traces of 2D coordination polymer [$Ag_4(CO_2(CF_2)_2CF_3)_4(TMP)_2$] and 1D coordination polymer [$Ag(CO_2(CF_2)_2CF_3)(TMP)$] were also identified and fitted by Rietveld refinement. Coordination polymers [$Ag_4(CO_2(CF_2)_2CF_3)_4(TMP)_2$] and [$Ag(CO_2(CF_2)_2CF_3)(TMP)$] remained unaltered through the entire gas uptake process. The CH_4 gas pressure was increased to 19.71 bar and a PXRD pattern obtained. Further scans were then obtained after changes in CH_4 pressures in the sequence 42.00, 18.35, 8.81, 4.31 and 1.13 bar. All scans in this sequence were measured at room temperature. Pawley refinement confirmed the presence of coordination polymer $\mathbf{1_B}^{\mathbf{HT}}$ through the whole pressure range with negligible change in unit cell volume, suggesting that no CH_4 gas up taken up by the material. In order to assess whether CH_4 uptake occurs at higher relative pressures (p/p_0), the temperature was decreased to 180 K and a series of scans were collected sequentially at 1.96, 4.87 and 25.33 bar CH_4 pressure. Pawley refinement confirmed the presence of low-temperature coordination polymer polymorph $\mathbf{1_B}^{\mathbf{LT}}$ through the whole pressure range, again suggesting that no CH_4 gas up taken up by the material. Unit cell parameters at each CH_4 gas pressure measured at room temperature and 180 K are provided in Supplementary Tables 13 and 14, respectively. Fits for individual PXRD patterns are provided in Supplementary Figures 46-52 and 53-55, respectively.

Supplementary Table 13. Unit cell parameters from Pawley refinement for compound **1** under CH_4 gas pressure at room temperature

Pressure (bar)	*Compound*	*a (Å)*	*b (Å)*	*c (Å)*	*α (°)*	*β (°)*	*γ (°)*	*V (Å^3)*
10^{-6}	$\mathbf{1_B}^{\mathbf{HT}}$	22.6811(4)	8.5456(2)	15.0646(4)	90	79.307(2)	90	2869.2(1)
19.71	$\mathbf{1_B}^{\mathbf{HT}}$	22.6821(4)	8.5455(3)	15.0688(7)	90	79.307(3)	90	2870.1(1)
42.00	$\mathbf{1_B}^{\mathbf{HT}}$	22.6809(6)	8.5430(3)	15.0626(6)	90	79.347(2)	90	2868.3(1)
18.35	$\mathbf{1_B}^{\mathbf{HT}}$	22.6811(6)	8.5436(3)	15.0642(7)	90	79.331(3)	90	2868.7(2)
8.81	$\mathbf{1_B}^{\mathbf{HT}}$	22.6798(5)	8.5431(2)	15.0617(5)	90	79.319(2)	90	2867.7(1)
4.31	$\mathbf{1_B}^{\mathbf{HT}}$	22.6810(7)	8.5436(3)	15.0612(4)	90	79.317(3)	90	2867.8(1)
1.13	$\mathbf{1_B}^{\mathbf{HT}}$	22.6798(8)	8.5440(4)	15.0601(3)	90	79.318(4)	90	2867.7(7)

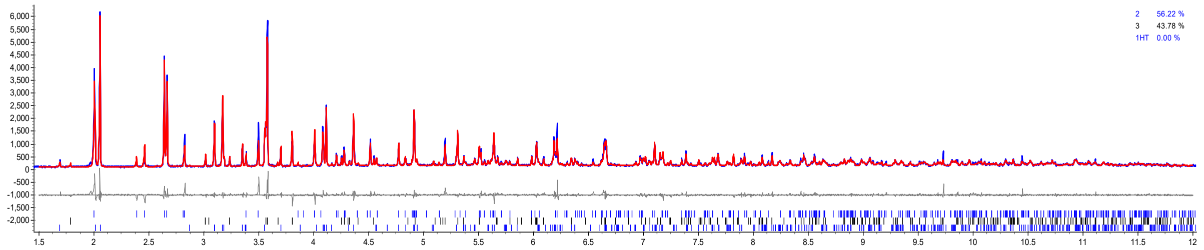

Supplementary Figure 46. Observed (blue) and calculated (red) profiles and difference plot [I_{obs}–I_{calc}] (grey) of the Pawley refinement of Pattern 1 at 10^{-6} mbar and room temperature ($1.5 \leq 2\theta \leq 12.0$ °; d_{min} = 1.91 Å) (R_{wp} = 13.129, R_{wp}' = 23.045) for $\mathbf{1_B}^{\mathbf{HT}}$. Minor-phase impurities of $[Ag_4(O_2C(CF_2)_2CF_3)_4(TMP)_2]$ and $[Ag(O_2C(CF_2)_2CF_3)(TMP)]$ that remain constant throughout the study are fitted by Rietveld methods.

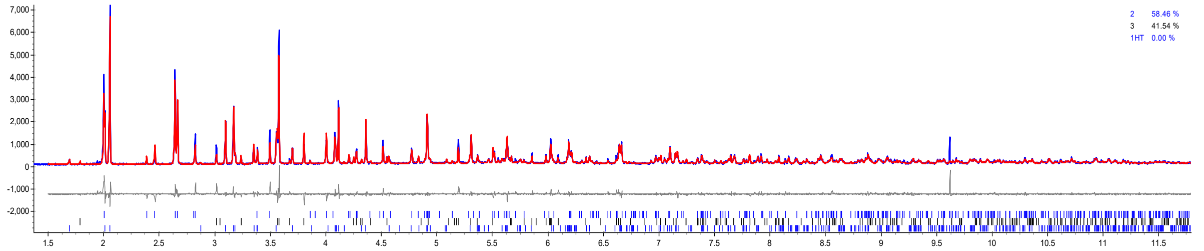

Supplementary Figure 47. Observed (blue) and calculated (red) profiles and difference plot [I_{obs}–I_{calc}] (grey) of the Pawley refinement of Pattern 2 at 19.71 bar CH_4 and room temperature ($1.5 \leq 2\theta \leq 12.0$ °; d_{min} = 1.91 Å) (R_{wp} = 14.222, R_{wp}' = 25.235) for $\mathbf{1_B}^{\mathbf{HT}}$. Minor-phase impurities of $[Ag_4(O_2C(CF_2)_2CF_3)_4(TMP)_2]$ and $[Ag(O_2C(CF_2)_2CF_3)(TMP)]$ that remain constant throughout the study are fitted by Rietveld methods.

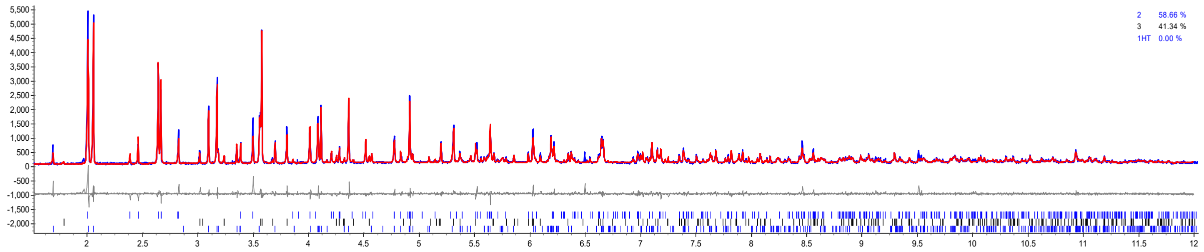

Supplementary Figure 48. Observed (blue) and calculated (red) profiles and difference plot [I_{obs}–I_{calc}] (grey) of the Pawley refinement of Pattern 3 at 42.00 bar CH_4 and room temperature ($1.5 \leq 2\theta \leq 12.0$ °; d_{min} = 1.91 Å) (R_{wp} = 13.411, R_{wp}' = 23.641) for $\mathbf{1_B}^{\mathbf{HT}}$. Minor-phase impurities of [$Ag_4(O_2C(CF_2)_2CF_3)_4(TMP)_2$] and [$Ag(O_2C(CF_2)_2CF_3)(TMP)$] that remain constant throughout the study are fitted by Rietveld methods.

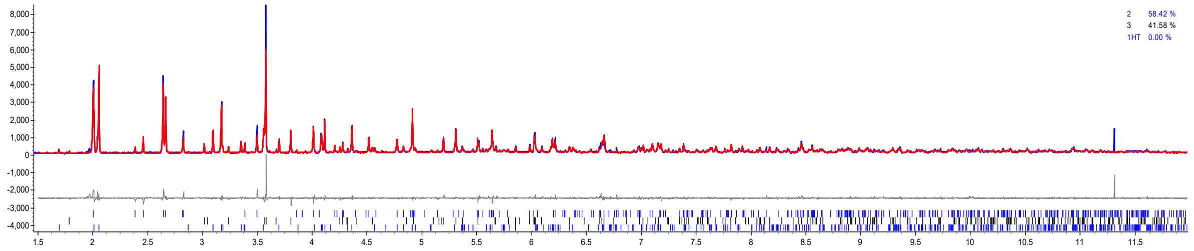

Supplementary Figure 49. Observed (blue) and calculated (red) profiles and difference plot [I_{obs}–I_{calc}] (grey) of the Pawley refinement of Pattern 4 at 18.35 bar CH_4 and room temperature ($1.5 \leq 2\theta \leq 12.0$ °; d_{min} = 1.91 Å) (R_{wp} = 13.637, R_{wp}' = 23.951) for $\mathbf{1_B}^{\mathbf{HT}}$. Minor-phase impurities of [$Ag_4(O_2C(CF_2)_2CF_3)_4(TMP)_2$] and [$Ag(O_2C(CF_2)_2CF_3)(TMP)$] that remain constant throughout the study are fitted by Rietveld methods.

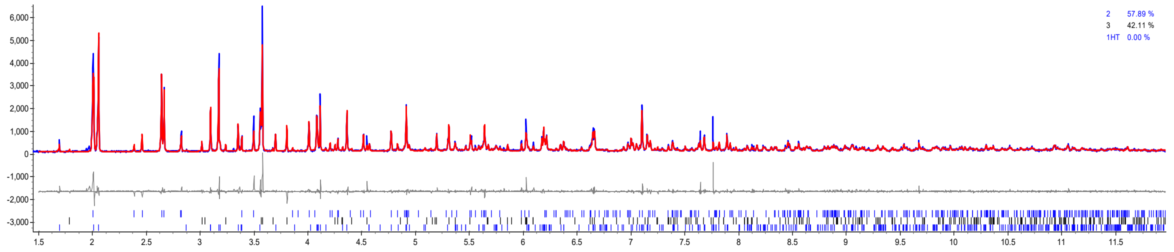

Supplementary Figure 50. Observed (blue) and calculated (red) profiles and difference plot [I_{obs}–I_{calc}] (grey)of the Pawley refinement of Pattern 5 at 8.81 bar CH_4 and room temperature ($1.5 \leq 2\theta \leq 12.0$ °; d_{min} = 1.91 Å) (R_{wp} = 14.686, R_{wp}' = 25.538) for $\mathbf{1_B}^{\mathbf{HT}}$. Minor-phase impurities of [$Ag_4(O_2C(CF_2)_2CF_3)_4(TMP)_2$] and [$Ag(O_2C(CF_2)_2CF_3)(TMP)$] that remain constant throughout the study are fitted by Rietveld methods.

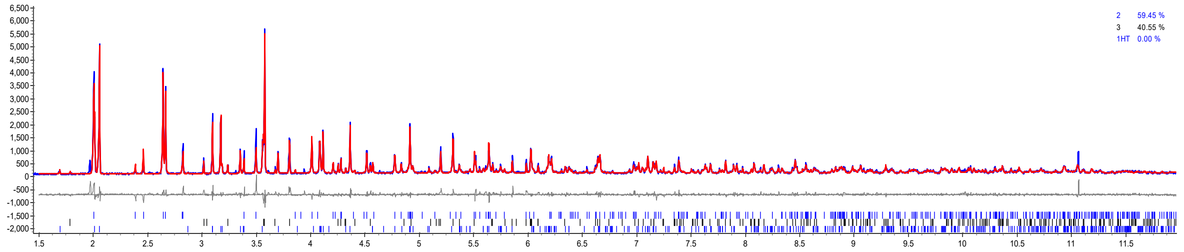

Supplementary Figure 51. Observed (blue) and calculated (red) profiles and difference plot [I_{obs}–I_{calc}] (grey) of the Pawley refinement of Pattern 6 at 4.31 bar CH_4 and room temperature ($1.5 \leq 2\theta \leq 12.0$ °; d_{min} = 1.91 Å) (R_{wp} = 14.452, R_{wp}' = 25.104) for $\mathbf{1_B}^{\mathbf{HT}}$. Minor-phase impurities of [$Ag_4(O_2C(CF_2)_2CF_3)_4(TMP)_2$] and [$Ag(O_2C(CF_2)_2CF_3)(TMP)$] that remain constant throughout the study are fitted by Rietveld methods.

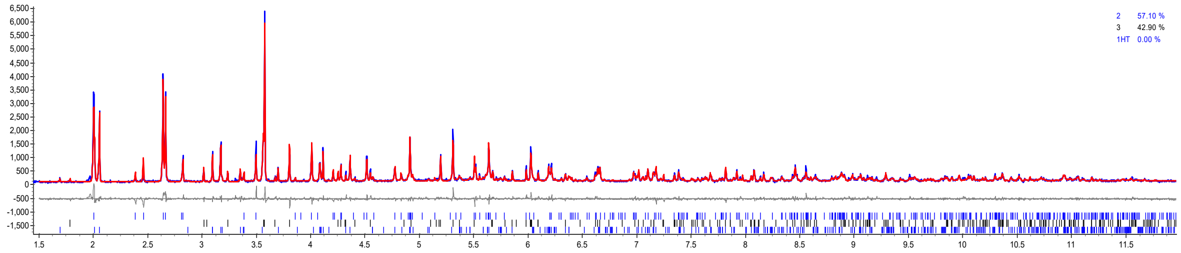

Supplementary Figure 52. Observed (blue) and calculated (red) profiles and difference plot [I_{obs}–I_{calc}] (grey) of the Pawley refinement of Pattern 7 at 1.13 bar CH_4 and room temperature ($1.5 \leq 2\theta \leq 12.0$ °; d_{min} = 1.91 Å) (R_{wp} = 13.287, R_{wp}' = 23.571) for $\mathbf{1_B}^{HT}$. Minor-phase impurities of [$Ag_4(O_2C(CF_2)_2CF_3)_4(TMP)_2$] and [$Ag(O_2C(CF_2)_2CF_3)(TMP)$] that remain constant throughout the study are fitted by Rietveld methods.

Supplementary Table 14. Unit cell parameters from Pawley refinement for compound **1** under CH_4 gas pressure at 180 K

Pressure (bar)	*Compound*	*a (Å)*	*b (Å)*	*c (Å)*	*α (°)*	*β (°)*	*γ (°)*	*V (Å³)*
1.96	$\mathbf{1_B}^{LT}$	28.763(6)	8.457(1)	24.445(5)	90	111.88(1)	90	5518(1)
4.87	$\mathbf{1_B}^{LT}$	28.483(3)	8.482(1)	24.518(3)	90	111.558(7)	90	5509(1)
25.33	$\mathbf{1_B}^{LT}$	28.450(4)	8.473(1)	24.434(3)	90	111.590(6)	90	5477(1)

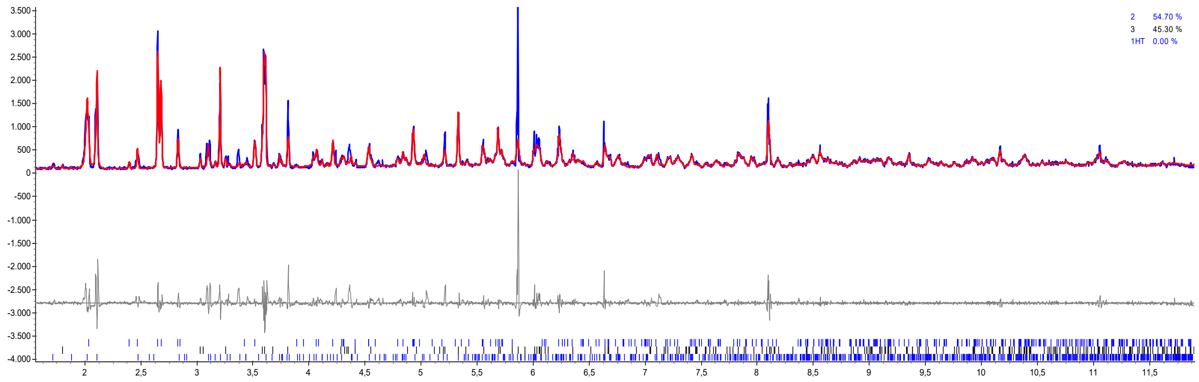

Supplementary Figure 53. Observed (blue) and calculated (red) profiles and difference plot [I_{obs}–I_{calc}] (grey) of the Pawley refinement of Pattern 1 at 1.96 bar CH_4 (T = 180 K) ($1.5 \leq 2\theta \leq 12.0$ °; d_{min} = 1.91 Å) (R_{wp} = 20.329, R_{wp}' = 32.507) for $\mathbf{1_B}^{LT}$. Minor-phase impurities of [$Ag_4(O_2C(CF_2)_2CF_3)_4(TMP)_2$] and [$Ag(O_2C(CF_2)_2CF_3)(TMP)$] that remain constant throughout the study are fitted by Rietveld methods.

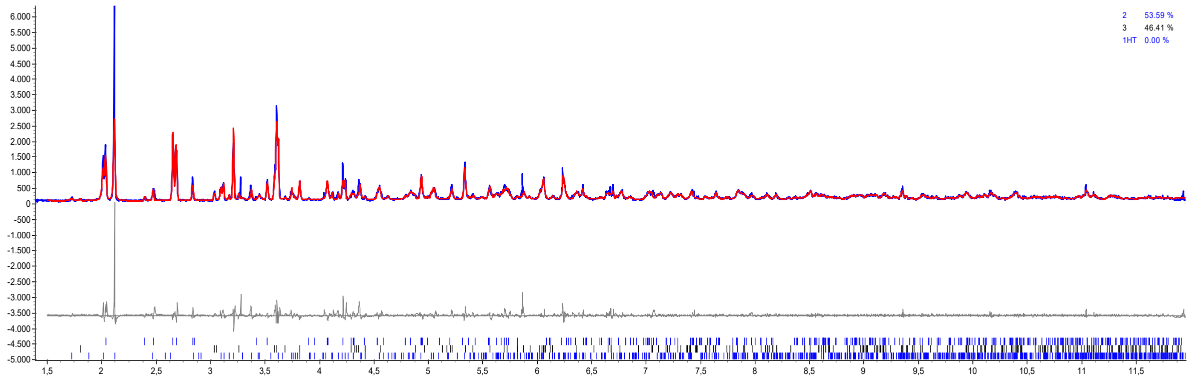

Supplementary Figure 54. Observed (blue) and calculated (red) profiles and difference plot [I_{obs}–I_{calc}] (grey) of the Pawley refinement of Pattern 2 at 4.87 bar CH_4 (T = 180 K) (1.5 ≤ 2θ ≤ 12.0 °; d_{min} = 1.91 Å) (R_{wp} = 16.901, R_{wp}' = 28.685) for $\mathbf{1_B}^{\mathbf{LT}}$. Minor-phase impurities of [$Ag_4(O_2C(CF_2)_2CF_3)_4(TMP)_2$] and [$Ag(O_2C(CF_2)_2CF_3)(TMP)$] that remain constant throughout the study are fitted by Rietveld methods.

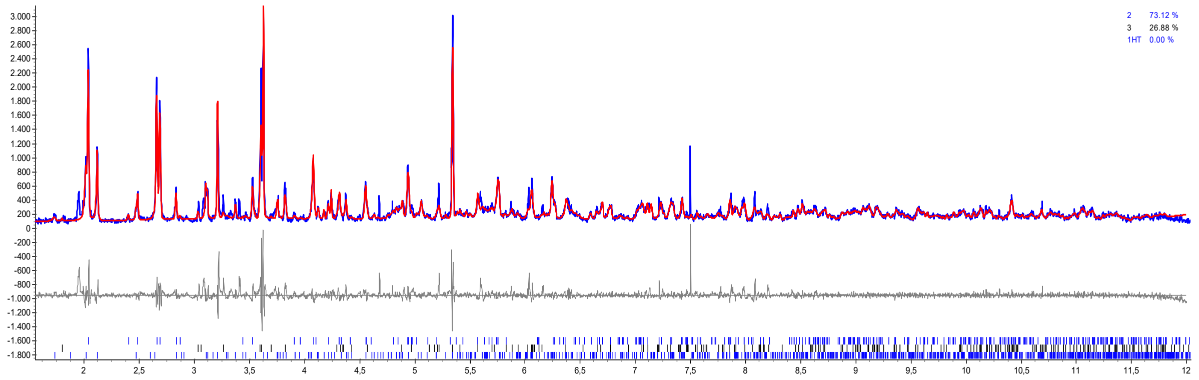

Supplementary Figure 55. Observed (blue) and calculated (red) profiles and difference plot [I_{obs}–I_{calc}] (grey) of the Pawley refinement of Pattern 3 at 25.33 bar CH_4 (T = 180 K) (1.5 ≤ 2θ ≤ 12.0 °; d_{min} = 1.91 Å) (R_{wp} = 18.141, R_{wp}' = 29.032) for $\mathbf{1_B}^{\mathbf{LT}}$. Minor-phase impurities of [$Ag_4(O_2C(CF_2)_2CF_3)_4(TMP)_2$] and [$Ag(O_2C(CF_2)_2CF_3)(TMP)$] that remain constant throughout the study are fitted by Rietveld methods.

4.2 *In situ* PXRD study of coordination polymer 2 exposure to CO_2 gas pressure

The structural changes associated with CO_2 absorption were explored for coordination polymer **2** using PXRD (λ= 0.826741(1) Å) at beamline I11 at Diamond Light Source.[S13] 10s scans were collected using a PSD detector, during which the capillary was oscillated about its axis through an angle of ca. 35 °. All data were collected at room temperature (295 K) at each in a sequence of CO_2 gas pressures (0, 1.19, 4.35, 5.15, 6.78, 7.68, 8.75, 11.07, 12.37, 15.52, 17.58, 19.16, 22.18, 49.23, 22.28, 18.55, 16.62, 14.33, 12.55, 10.33, 7.92, 5.05, 1.4 bar), with 30 minutes intervals in between measurements for equilibration of the structure along the capillary at each pressure. The powder patterns were indexed using *TOPAS*.[S10] A two-phase refinement was implemented in *TOPAS*, which comprised a Pawley refinement[S11] using the unit cell parameters of **2** or $\mathbf{2}^{\mathbf{CO2}}$ (depending on the pressure for the CO_2 gas uptake), and the unit cell parameters of a minor impurity [$Ag_4(O_2C(CF_2)_3CF_3)_4(TMP)_2$].[S4] Unit cell parameters at each gas pressure are provided in Supplementary Table 15 and summarized in Supplementary Figure 56. Fits for individual PXRD patterns are provided in Supplementary Figures 57-79.

An initial PXRD scan on coordination polymer **2** showed some traces of coordination polymer **2-EtOH** and 2D coordination polymer [$Ag_4(CO_2(CF_2)_3CF_3)_4(TMP)_2$]. The capillary was heated for 20 minutes 340 K under vacuum, and it was subsequently cooled down to room temperature. A scan was collected confirming the release of the coordinated ethanol molecules from **2-EtOH** to form **2** (polymorph $\mathbf{2^{HT}}$), with retaining traces of [$Ag_4(CO_2(CF_2)_3CF_3)_4(TMP)_2$] as anticipated. The CO_2 pressure was subsequently increased to 1.19 bar, then 4.35 bar and new scans were collected at room temperature. Pawley refinement showed the presence of the high-temperature polymorph of coordination polymer **2** ($\mathbf{2^{HT}}$). Scans were then collected sequentially at CO_2 pressures of 5.15, 6.78, 7.68, 8.85 and 11.07 bar at room temperature before the collapse of the capillary. Pawley refinement indicated the presence of coordination polymer $\mathbf{2^{HT}}$ and traces of [$Ag_4(CO_2(CF_2)_3CF_3)_4(TMP)_2$] across this CO_2 gas pressure range. A second separate capillary containing coordination polymer **2** ($\mathbf{2^{HT}}$) was set to the sequence of CO_2 pressures 12.37, 15.52 and 17.58 bar and a new set of scans measured for each at room temperature. Pawley refinement confirmed the presence of coordination polymer $\mathbf{2^{HT}}$ and traces of [$Ag_4(CO_2(CF_2)_3CF_3)_4(TMP)_2$] across this range of pressures. The CO_2 pressure was increased to 19.16 bar and a new pattern collected at room temperature. Pawley refinement confirmed the presence of a mixture of coordination polymers $\mathbf{2^{HT}}$, $\mathbf{2^{CO2}}$ and traces of [$Ag_4(CO_2(CF_2)_3CF_3)_4(TMP)_2$]. The CO_2 pressure was gradually increased to 22.18 bar and the new data set collected at room temperature showed the full conversion of coordination polymer $\mathbf{2^{HT}}$ into $\mathbf{2^{CO2}}$, the designation of the latter being made based on the marked increase in unit cell volume relative to $\mathbf{2^{HT}}$ (Supplementary Table 15). No change was observed in unit cell parameters for minor impurity [$Ag_4(CO_2(CF_2)_3CF_3)_4(TMP)_2$]. A final increase in CO_2 pressure to 49.23 bar was made and a pattern was collected at room temperature. Pawley refinement showed the persistence of $\mathbf{2^{CO2}}$ and a minor phase attributed to a new form of 2D coordination polymer [$Ag_4(CO_2(CF_2)_3CF_3)_4(TMP)_2$], presumably due to some absorption of CO_2 at this pressure. A sequence of pressure reductions was then undertaken to explore the reversibility of the processes via CO_2 desorption. A sequence of scans was measured at room temperature at CO_2 pressures of 22.28, 18.55, 16.62, 14.33, 12.55 and 7.92 bar. Pawley refinement of the pattern at 22.28 bar CO_2 showed the reformation of 2D coordination polymer [$Ag_4(CO_2(CF_2)_3CF_3)_4(TMP)_2$] as the minor phase, confirming the reversibility of the phase change. The presence of coordination polymer $\mathbf{2^{CO2}}$ as the major phase was confirmed by Pawley refinement throughout the pressure range until the CO_2 pressure reached 7.92 bar, at which stage both $\mathbf{2^{HT}}$ and $\mathbf{2^{CO2}}$ were observed. Full desorption of the CO_2 gas was confirmed by Pawley refinement of patterns measured at 5.05 and 1.4 bar CO_2 at room temperature, which showed $\mathbf{2^{HT}}$ together with the minor impurity phase of [$Ag_4(CO_2(CF_2)_3CF_3)_4(TMP)_2$].

Supplementary Table 15. Unit cell parameters from Pawley refinement for compound **2** under CO_2 gas pressure at room temperature

Pressure (Bar)	*Compound*	*a (Å)*	*b (Å)*	*c (Å)*	*α (°)*	*β (°)*	*γ (°)*	*V (Å³)*
0	$\mathbf{2^{HT}}$	8.656(1)	14.002(2)	15.023(2)	112.903(3)	90.647(4)	106.366(4)	1594.1(4)
1.19	$\mathbf{2^{HT}}$	8.6428(3)	14.0001(6)	14.9447(8)	113.197(2)	90.963(3)	106.338(4)	1578.2(1)
4.35	$\mathbf{2^{HT}}$	8.6384(3)	13.9774(7)	14.9370(9)	113.140(9)	90.928(3)	106.351(3)	1579.4(1)
5.15	$\mathbf{2^{HT}}$	8.6373(4)	13.9888(7)	14.9517(9)	113.147(3)	90.951(3)	106.356(3)	1577.9(1)
6.78	$\mathbf{2^{HT}}$	8.6368(4)	13.9903(8)	14.9637(9)	113.100(2)	90.942(3)	106.365(4)	1579.1(1)
7.68	$\mathbf{2^{HT}}$	8.6360(3)	13.9897(7)	14.9699(9)	113.074(2)	90.934(3)	106.369(3)	1579.9(1)
8.75	$\mathbf{2^{HT}}$	8.6360(3)	13.990(1)	14.9696(9)	113.074(3)	90.935(3)	106.369(3)	1579.9(1)
11.07	$\mathbf{2^{HT}}$	8.636(1)	13.990(3)	14.990(3)	113.074(3)	90.934(3)	106.369(4)	1579.9(5)
12.37	$\mathbf{2^{HT}}$	8.6497(3)	14.0234(4)	14.970(1)	113.216(3)	90.974(3)	106.281(4)	1585.1(1)
15.52	$\mathbf{2^{HT}}$	8.6493(4)	14.0257(7)	14.9781(9)	113.173(3)	90.960(2)	106.294(3)	1586.6(1)
17.58	$\mathbf{2^{HT}}$	8.6481(3)	14.0263(5)	14.9819(8)	113.132(3)	90.940(2)	106.308(4)	1587.3(1)

19.16	(**2**^{HT} and) **2**$^{\mathrm{CO2}}$	8.481(1)	14.336(1)	15.172(1)	104.698(9)	84.29(1)	105.85(1)	1688.0(3)
22.18	**2**$^{\mathrm{CO2}}$	8.481(1)	14.340(1)	15.179(1)	104.693(8)	84.30(1)	105.76(1)	1690.2(3)
49.23	**2**$^{\mathrm{CO2}}$	8.4889(7)	14.3887(8)	15.2118(8)	104.757(3)	84.478(9)	105.877(6)	1700.5(2)
22.28	**2**$^{\mathrm{CO2}}$	8.4907(7)	14.3534(7)	15.1782(8)	104.705(4)	84.273(9)	105.792(8)	1693.1(2)
18.55	**2**$^{\mathrm{CO2}}$	8.4870(3)	14.3391(5)	15.1697(5)	104.649(3)	84.250(3)	105.777(3)	1690.2(1)
16.62	**2**$^{\mathrm{CO2}}$	8.4849(3)	14.3285(4)	15.1670(5)	104.621(3)	84.241(2)	105.758(3)	1688.7(1)
14.33	**2**$^{\mathrm{CO2}}$	8.4826(3)	14.3166(5)	15.1638(5)	104.590(3)	84.227(3)	105.743(3)	1686.9(1)
12.55	**2**$^{\mathrm{CO2}}$	8.4807(3)	14.3049(5)	15.1603(5)	104.561(3)	84.214(3)	105.731(3)	1685.0(1)
10.33	**2**$^{\mathrm{CO2}}$	8.4773(3)	14.2882(5)	15.1589(6)	104.512(4)	84.202(7)	105.708(3)	1682.8(1)
7.92	(**2**^{HT} and) **2**$^{\mathrm{CO2}}$	8.4710(3)	14.2544(7)	15.1551(6)	104.436(3)	84.188(3)	105.689(4)	1678.0(1)
5.05	**2**^{HT}	8.6434(3)	14.016(8)	14.9812(6)	113.038(4)	90.906(3)	106.389(3)	1585.8(1)
1.4	**2**^{HT}	8.6419(3)	14.0032(7)	14.9781(5)	113.058(3)	90.894(4)	106.410(4)	1583.3(1)

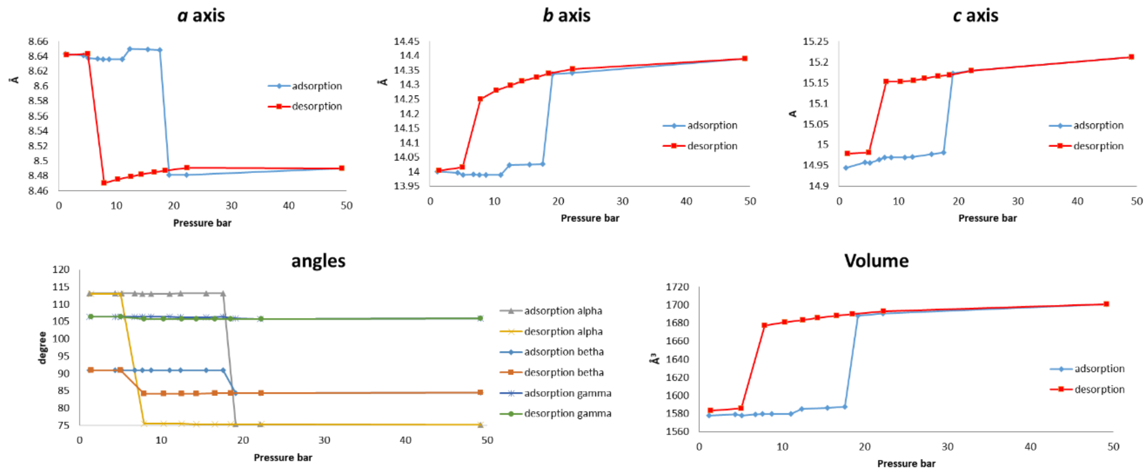

Supplementary Figure 56. Unit cell values from Pawley refinement of PXRD patterns for coordination polymer **2** (**2**^{HT} or **2**$^{\mathrm{CO2}}$ depending on CO_2 gas pressure). PXRD data were collected sequentially at CO_2 gas pressures of 0, 1.19, 4.35, 5.15, 6.78, 7.68, 8.75, 11.07, 12.37, 15.52, 17.58, 19.16, 22.18, 49.23, 22.28, 18.55, 16.62, 14.33, 12.55, 10.33, 7.92, 5.05, 1.4 bar at room temperature.

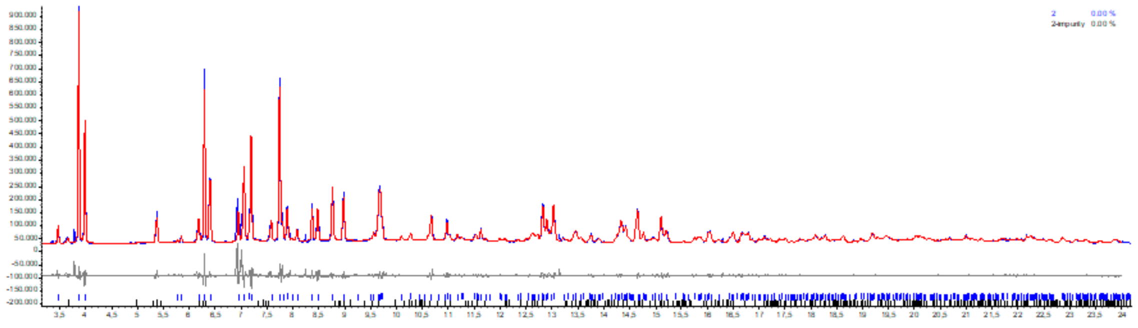

Supplementary Figure 57. Observed (blue) and calculated (red) profiles and difference plot [I_{obs}–I_{calc}] (grey) of the Pawley refinement of Pattern 1 at 10^{-6} bar CO_2 (T = 295 K) ($3.0 \leq 2\theta \leq 24.0$ °; d_{min} = 2.03 Å) (R_{wp} = 5.066, R_{wp}' = 13.617) for **2**^{HT}. Minor-phase impurity phase of [$Ag_4(O_2C(CF_2)_3CF_3)_4(TMP)_2$] is also fitted by Pawley methods.

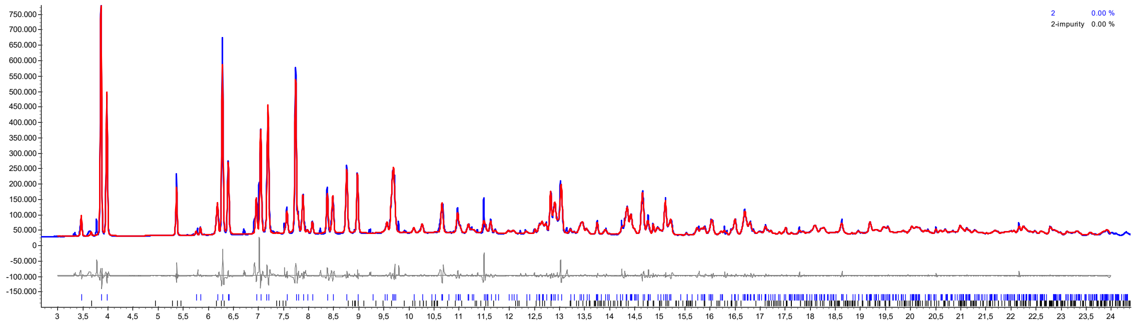

Supplementary Figure 58. Observed (blue) and calculated (red) profiles and difference plot [I_{obs}–I_{calc}] (grey) of the Pawley refinement of Pattern 2 at 1.19 bar CO_2 (T = 295 K) (3.0 ≤ 2θ ≤ 24.0 °; d_{min} = 2.03 Å) (R_{wp} = 7.715, R_{wp}' = 15.689) for **2^{HT}**. Minor-phase impurity phase of [$Ag_4(O_2C(CF_2)_3CF_3)_4(TMP)_2$] is also fitted by Pawley methods.

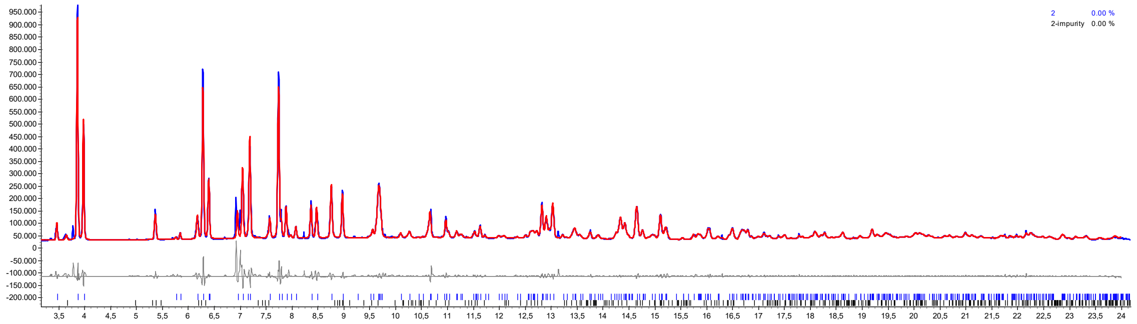

Supplementary Figure 59. Observed (blue) and calculated (red) profiles and difference plot [Iobs–Icalc] (grey) of the Pawley refinement of Pattern 3 at 4.35 bar CO_2 (T = 295 K) (3.0 ≤ 2θ ≤ 24.0 °; d_{min} = 2.03 Å) (R_{wp} = 8.629, R_{wp}' = 17.584) for **2^{HT}**. Minor-phase impurity phase of [$Ag_4(O_2C(CF_2)_3CF_3)_4(TMP)_2$] is also fitted by Pawley methods.

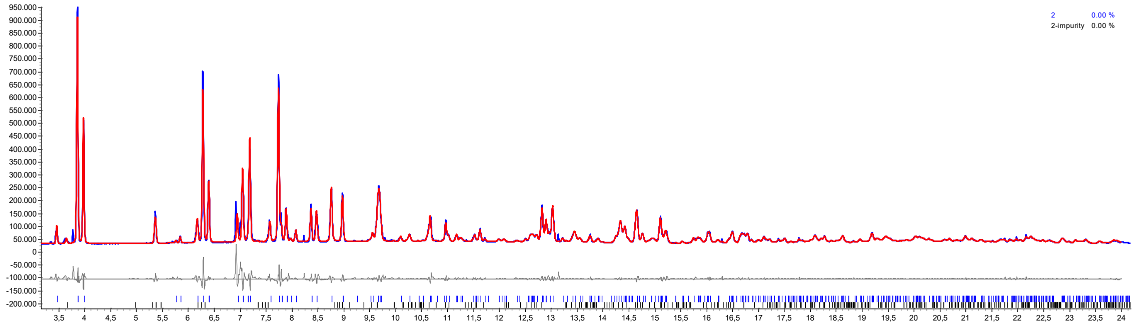

Supplementary Figure 60. Observed (blue) and calculated (red) profiles and difference plot [I_{obs}–I_{calc}] (grey) of the Pawley refinement of Pattern 4 at 5.15 bar CO_2 (T = 295 K) (3.0 ≤ 2θ ≤ 24.0 °; d_{min} = 2.03 Å) (R_{wp} = 7.403, R_{wp}' = 15.181) for **2^{HT}**. Minor-phase impurity phase of [$Ag_4(O_2C(CF_2)_3CF_3)_4(TMP)_2$] is also fitted by Pawley methods.

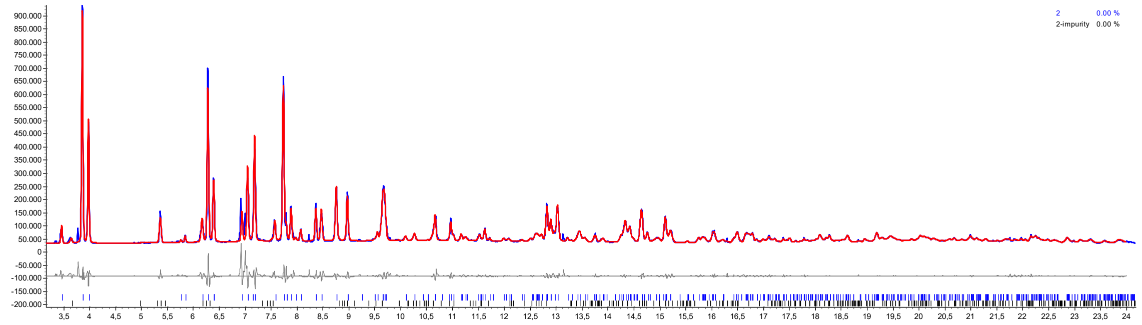

Supplementary Figure 61. Observed (blue) and calculated (red) profiles and difference plot [I_{obs}–I_{calc}] (grey) of the Pawley refinement of Pattern 5 at 6.78 bar CO_2 (T = 295 K) ($3.0 \leq 2\theta \leq 24.0$ °; d_{min} = 2.03 Å) (R_{wp} = 7.474, R_{wp}' = 15.289) for $\mathbf{2}^{\mathbf{HT}}$. Minor-phase impurity phase of [$Ag_4(O_2C(CF_2)_3CF_3)_4(TMP)_2$] is also fitted by Pawley methods.

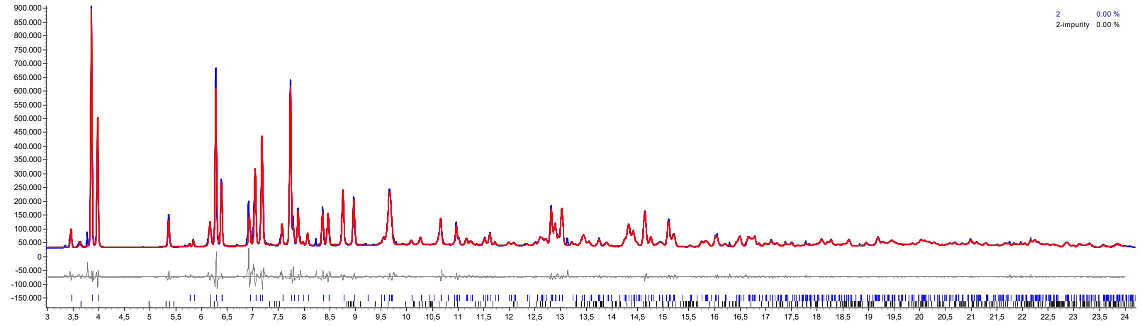

Supplementary Figure 62. Observed (blue) and calculated (red) profiles and difference plot [I_{obs}–I_{calc}] (grey) of the Pawley refinement of Pattern 6 at 7.68 bar CO_2 (T = 295 K) ($3.0 \leq 2\theta \leq 24.0$ °; d_{min} = 2.03 Å) (R_{wp} = 6.865, R_{wp}' = 13.977) for $\mathbf{2}^{\mathbf{HT}}$. Minor-phase impurity phase of [$Ag_4(O_2C(CF_2)_3CF_3)_4(TMP)_2$] is also fitted by Pawley methods.

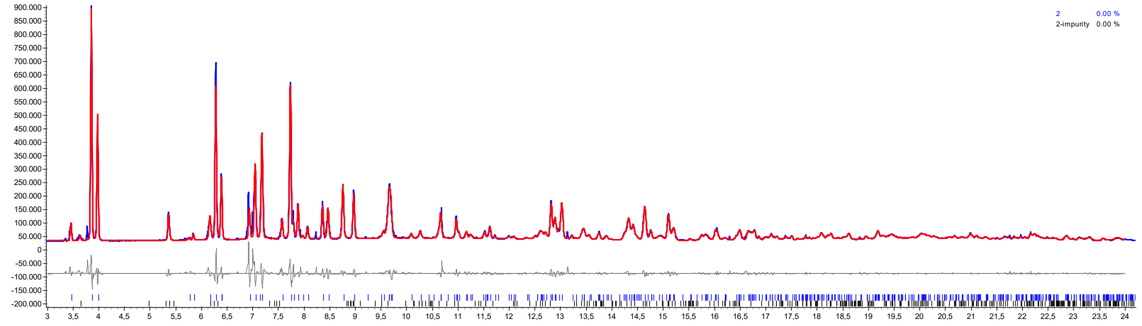

Supplementary Figure 63. Observed (blue) and calculated (red) profiles and difference plot [I_{obs}–I_{calc}] (grey) of the Pawley refinement of Pattern 7 at 8.75 bar CO_2 (T = 295 K) ($3.0 \leq 2\theta \leq 24.0$ °; d_{min} = 2.03 Å) (R_{wp} = 8.075, R_{wp}' = 6.422) for $\mathbf{2}^{\mathbf{HT}}$. Minor-phase impurity phase of [$Ag_4(O_2C(CF_2)_3CF_3)_4(TMP)_2$] is also fitted by Pawley methods.

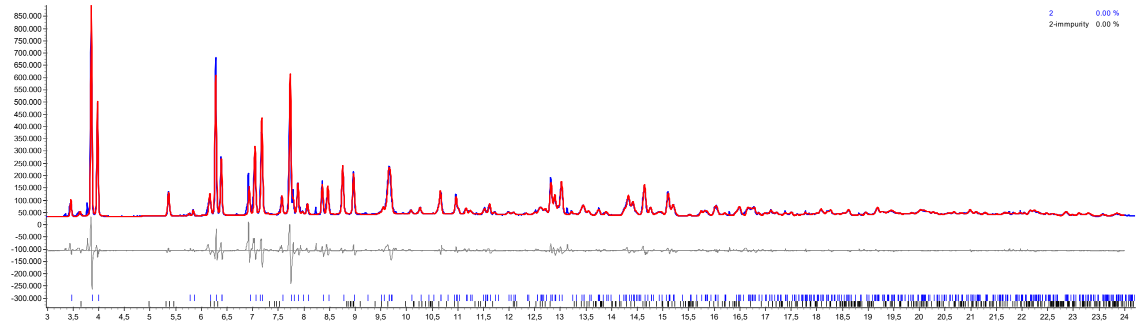

Supplementary Figure 64. Observed (blue) and calculated (red) profiles and difference plot [I_{obs}–I_{calc}] (grey) of the Pawley refinement of Pattern 8 at 11.07 bar CO_2 (T = 295 K) (3.0 ≤ 2θ ≤ 24.0 °; d_{min} = 2.03 Å) (R_{wp} = 9.881, R_{wp}' = 20.253) for $\mathbf{2}^{\mathbf{HT}}$. Minor-phase impurity phase of [$Ag_4(O_2C(CF_2)_3CF_3)_4(TMP)_2$] is also fitted by Pawley methods.

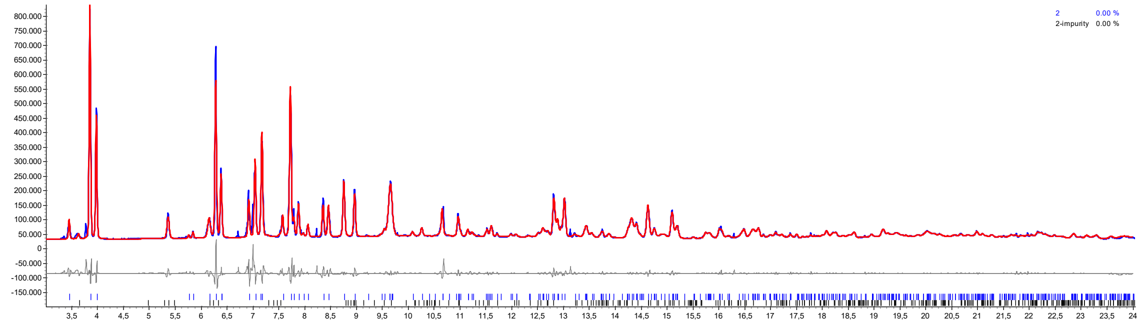

Supplementary Figure 65. Observed (blue) and calculated (red) profiles and difference plot [I_{obs}–I_{calc}] (grey) of the Pawley refinement of Pattern 9 at 12.37 bar CO_2 (T = 295 K) (3.0 ≤ 2θ ≤ 24.0 °; d_{min} = 2.03 Å) (R_{wp} = 7.211, R_{wp}' = 14.815) for $\mathbf{2}^{\mathbf{HT}}$. Minor-phase impurity phase of [$Ag_4(O_2C(CF_2)_3CF_3)_4(TMP)_2$] is also fitted by Pawley methods.

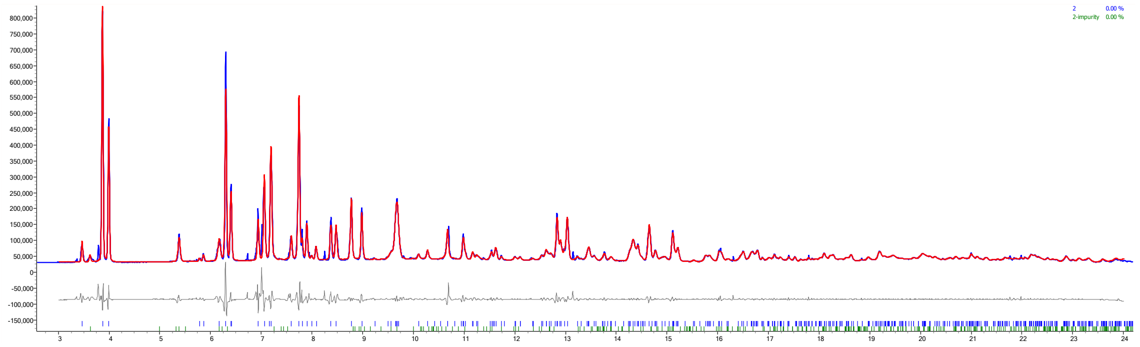

Supplementary Figure 66. Observed (blue) and calculated (red) profiles and difference plot [I_{obs}–I_{calc}] (grey) of the Pawley refinement of Pattern 10 at 15.52 bar CO_2 (T = 295 K) (3.0 ≤ 2θ ≤ 24.0 °; d_{min} = 2.03 Å) (R_{wp} = 7.010 , R_{wp}' = 14.385) for $\mathbf{2}^{\mathbf{HT}}$. Minor-phase impurity phase of [$Ag_4(O_2C(CF_2)_3CF_3)_4(TMP)_2$] is also fitted by Pawley methods.

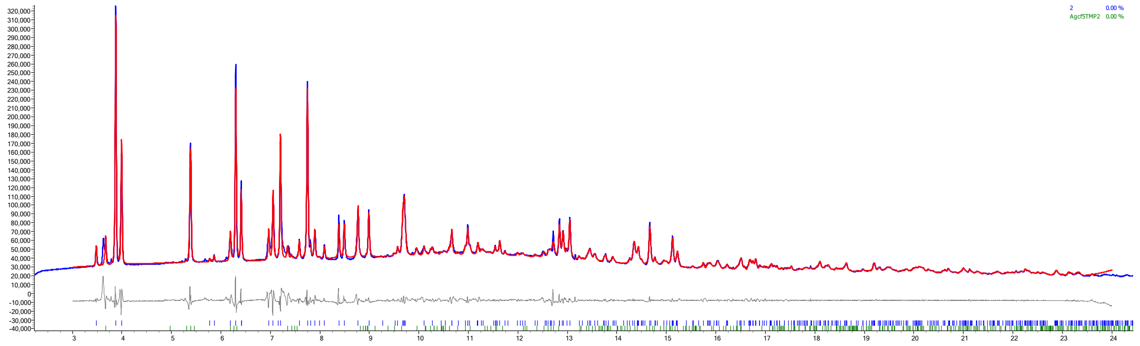

Supplementary Figure 67. Observed (blue) and calculated (red) profiles and difference plot [I_{obs}–I_{calc}] (grey) of the Pawley refinement of Pattern 11 at 17.58 bar CO_2 (T = 295 K) ($3.0 \leq 2\theta \leq 24.0$ °; d_{min} = 2.03 Å) (R_{wp} = 4.564, R_{wp}' = 16.448) for $\mathbf{2}^{\mathbf{HT}}$. Minor-phase impurity phase of [$Ag_4(O_2C(CF_2)_3CF_3)_4(TMP)_2$] is also fitted by Pawley methods.

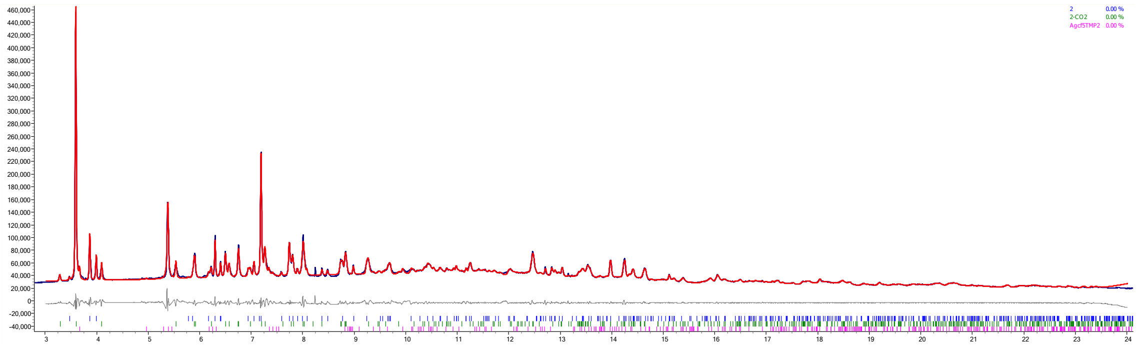

Supplementary Figure 68. Observed (blue) and calculated (red) profiles and difference plot [I_{obs}–I_{calc}] (grey) of the Pawley refinement of Pattern 12 at 19.16 bar CO_2 (T = 295 K) ($3.0 \leq 2\theta \leq 24.0$ °; d_{min} = 2.03 Å) (R_{wp} = 3.625, R_{wp}' = 10.470) for mixed phase of $\mathbf{2}^{\mathbf{HT}}$ and $\mathbf{2}^{\mathbf{CO2}}$. Minor-phase impurity phase of [$Ag_4(O_2C(CF_2)_3CF_3)_4(TMP)_2$] is also fitted by Pawley methods.

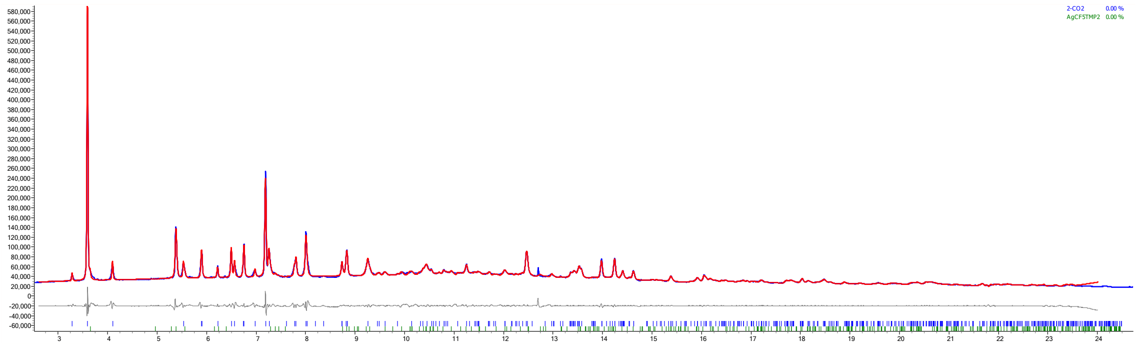

Supplementary Figure 69. Observed (blue) and calculated (red) profiles and difference plot [I_{obs}–I_{calc}] (grey) of the Pawley refinement of Pattern 13 at 22.18 bar CO_2 (T = 295 K) ($3.0 \leq 2\theta \leq 24.0$ °; d_{min} = 2.03 Å) (R_{wp} = 4.141, R_{wp}' = 11.877) for $\mathbf{2}^{\mathbf{CO2}}$. Minor-phase impurity phase of [$Ag_4(O_2C(CF_2)_3CF_3)_4(TMP)_2$] is also fitted by Pawley methods.

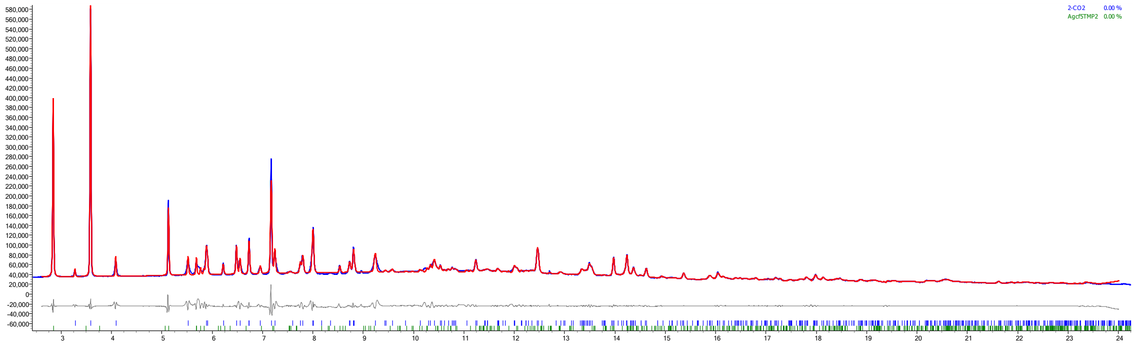

Supplementary Figure 70. Observed (blue) and calculated (red) profiles and difference plot [I_{obs}–I_{calc}] (grey) of the Pawley refinement of Pattern 14 at 49.23 bar CO_2 (T = 295 K) ($3.0 \leq 2\theta \leq 24.0$ °; d_{min} = 2.03 Å) (R_{wp} = 4.312, R_{wp}' = 11.803) for $\mathbf{2}^{CO2}$. Minor-phase impurity phase of [$Ag_4(O_2C(CF_2)_3CF_3)_4(TMP)_2$] is also fitted by Pawley methods.

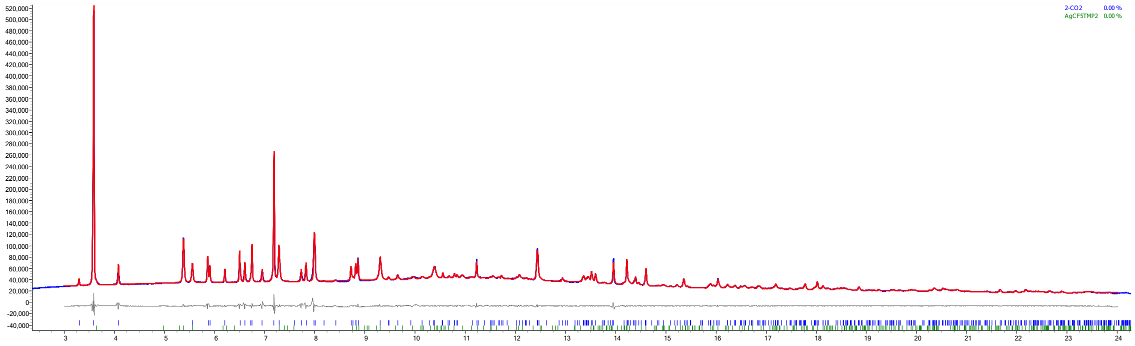

Supplementary Figure 71. Observed (blue) and calculated (red) profiles and difference plot [I_{obs}–I_{calc}] (grey) of the Pawley refinement of Pattern 15 at 22.28 bar CO_2 (T = 295 K) ($3.0 \leq 2\theta \leq 24.0$ °; d_{min} = 2.03 Å) (R_{wp} = 2.432, R_{wp}' = 11.011) for $\mathbf{2}^{CO2}$. Minor-phase impurity phase of [$Ag_4(O_2C(CF_2)_3CF_3)_4(TMP)_2$] is also fitted by Pawley methods.

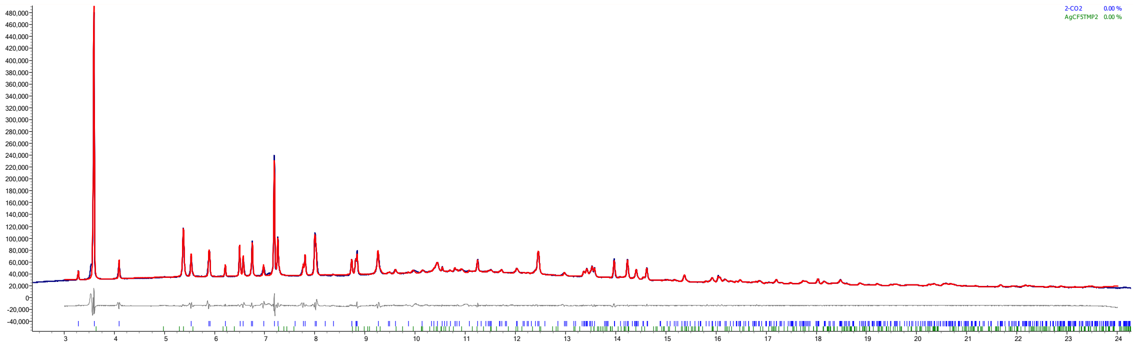

Supplementary Figure 72. Observed (blue) and calculated (red) profiles and difference plot [I_{obs}–I_{calc}] (grey) of the Pawley refinement of Pattern 16 at 18.55 bar CO_2 (T = 295 K) ($3.0 \leq 2\theta \leq 24.0$ °; d_{min} = 2.03 Å) (R_{wp} = 3.249, R_{wp}' = 14.012) for $\mathbf{2}^{CO2}$. Minor-phase impurity phase of [$Ag_4(O_2C(CF_2)_3CF_3)_4(TMP)_2$] is also fitted by Pawley methods.

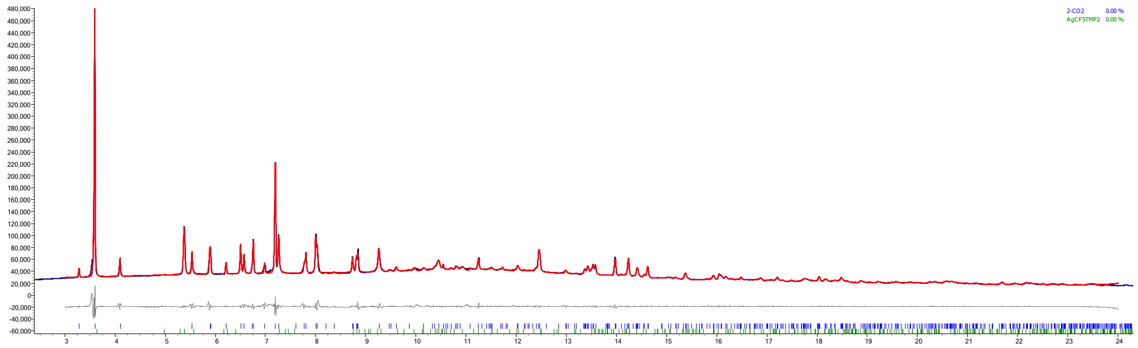

Supplementary Figure 73. Observed (blue) and calculated (red) profiles and difference plot [I_{obs}–I_{calc}] (grey) of the Pawley refinement of Pattern 17 at 16.62 bar CO_2 (T = 295 K) (3.0 ≤ 2θ ≤ 24.0 °; d_{min} = 2.03 Å) (R_{wp} = 3.246, R_{wp}' = 13.689) for $\mathbf{2}^{CO2}$. Minor-phase impurity phase of $[Ag_4(O_2C(CF_2)_3CF_3)_4(TMP)_2]$ is also fitted by Pawley methods.

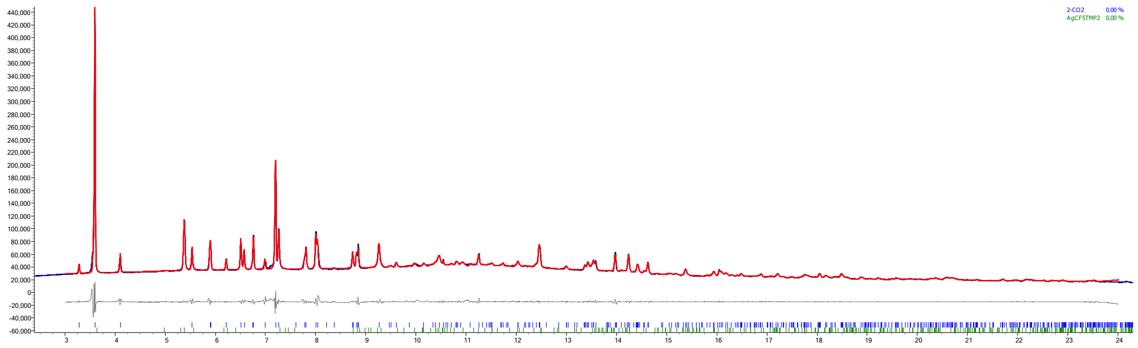

Supplementary Figure 74. Observed (blue) and calculated (red) profiles and difference plot [I_{obs}–I_{calc}] (grey) of the Pawley refinement of Pattern 18 at 14.32 bar CO_2 (T = 295 K) (3.0 ≤ 2θ ≤ 24.0 °; d_{min} = 2.03 Å) (R_{wp} = 3.132, R_{wp}' = 12.921) for $\mathbf{2}^{CO2}$. Minor-phase impurity phase of $[Ag_4(O_2C(CF_2)_3CF_3)_4(TMP)_2]$ is also fitted by Pawley methods.

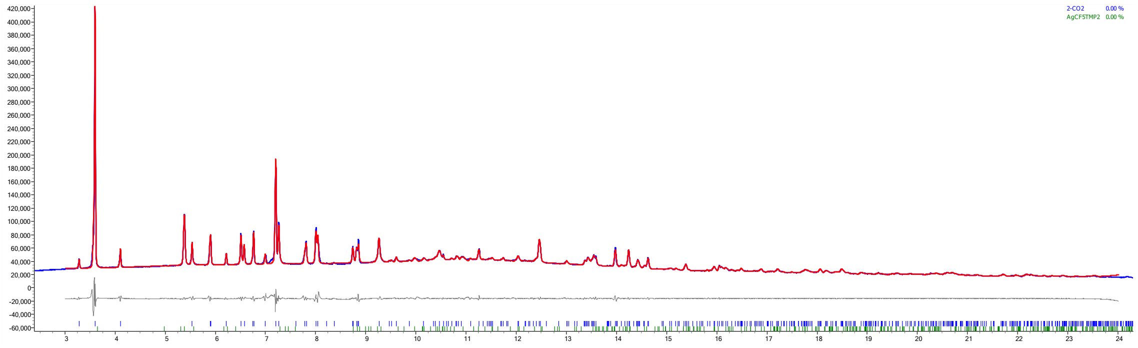

Supplementary Figure 75. Observed (blue) and calculated (red) profiles and difference plot [I_{obs}–I_{calc}] (grey) of the Pawley refinement of Pattern 19 at 12.55 bar CO_2 (T = 295 K) (3.0 ≤ 2θ ≤ 24.0 °; d_{min} = 2.03 Å) (R_{wp} = 2.992, R_{wp}' = 11.634) for $\mathbf{2}^{CO2}$. Minor-phase impurity phase of $[Ag_4(O_2C(CF_2)_3CF_3)_4(TMP)_2]$ is also fitted by Pawley methods.

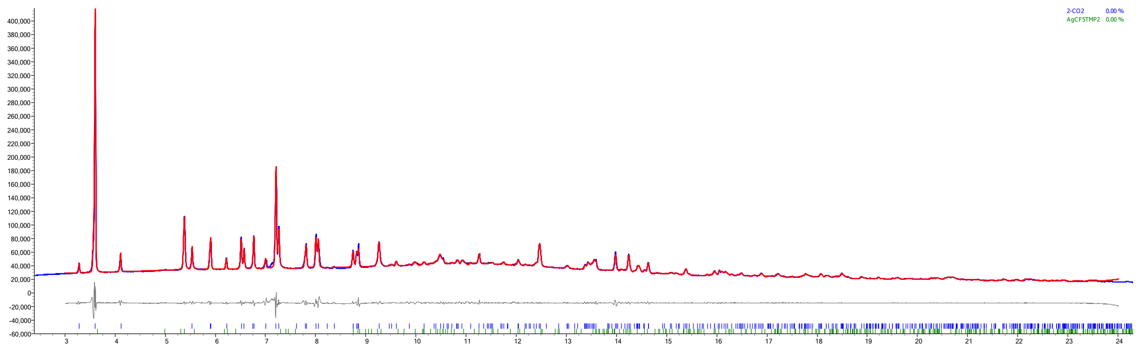

Supplementary Figure 76. Observed (blue) and calculated (red) profiles and difference plot [I_{obs}–I_{calc}] (grey) of the Pawley refinement of Pattern 20 at 10.33 bar CO_2 (T = 295 K) (3.0 ≤ 2θ ≤ 24.0 °; d_{min} = 2.03 Å) (R_{wp} = 2.980, R_{wp}' = 11.099) for $\mathbf{2}^{\mathbf{CO2}}$. Minor-phase impurity phase of [$Ag_4(O_2C(CF_2)_3CF_3)_4(TMP)_2$] is also fitted by Pawley methods.

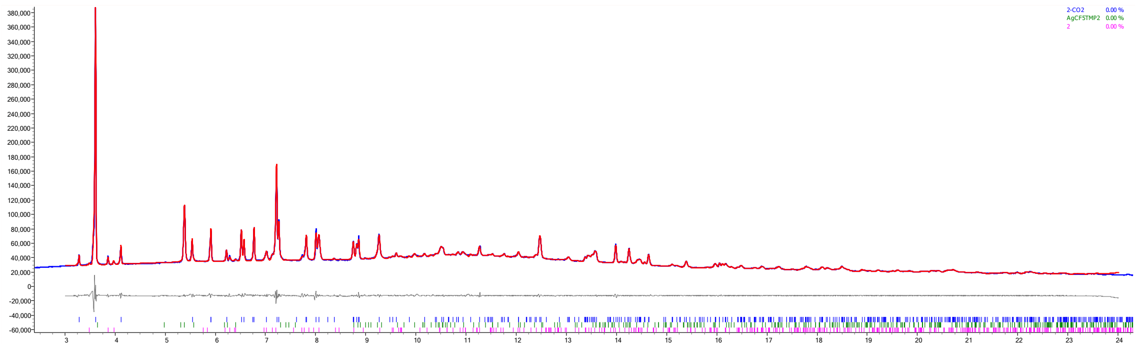

Supplementary Figure 77. Observed (blue) and calculated (red) profiles and difference plot [I_{obs}–I_{calc}] (grey) of the Pawley refinement of Pattern 21 at 7.92 bar CO_2 (T = 295 K) (3.0 ≤ 2θ ≤ 24.0 °; d_{min} = 2.03 Å) (R_{wp} = 2.274, R_{wp}' = 7.271) for mixed phase of $\mathbf{2}^{\mathbf{HT}}$ and $\mathbf{2}^{\mathbf{CO2}}$. Minor-phase impurity phase of [$Ag_4(O_2C(CF_2)_3CF_3)_4(TMP)_2$] is also fitted by Pawley methods.

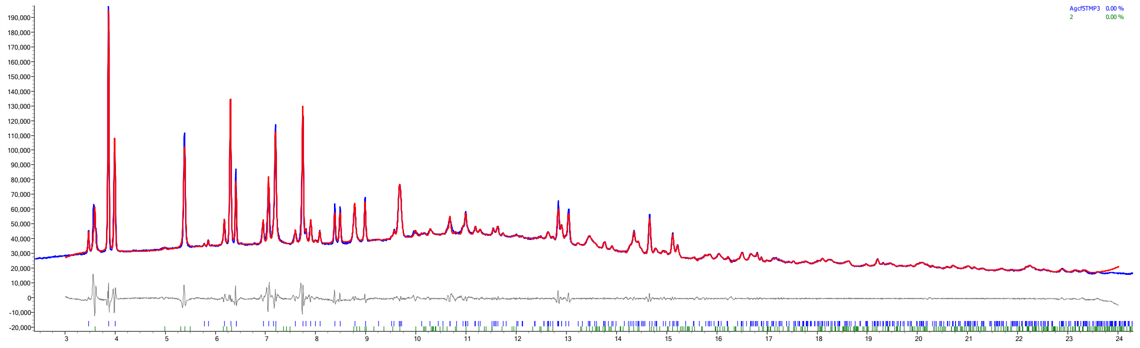

Supplementary Figure 78. Observed (blue) and calculated (red) profiles and difference plot [I_{obs}–I_{calc}] (grey) of the Pawley refinement of Pattern 22 at 5.05 bar CO_2 (T = 295 K) (3.0 ≤ 2θ ≤ 24.0 °; d_{min} = 2.03 Å) (R_{wp} = 3.212, R_{wp}' = 14.821) for $\mathbf{2}^{\mathbf{HT}}$. Minor-phase impurity phase of [$Ag_4(O_2C(CF_2)_3CF_3)_4(TMP)_2$] is also fitted by Pawley methods.

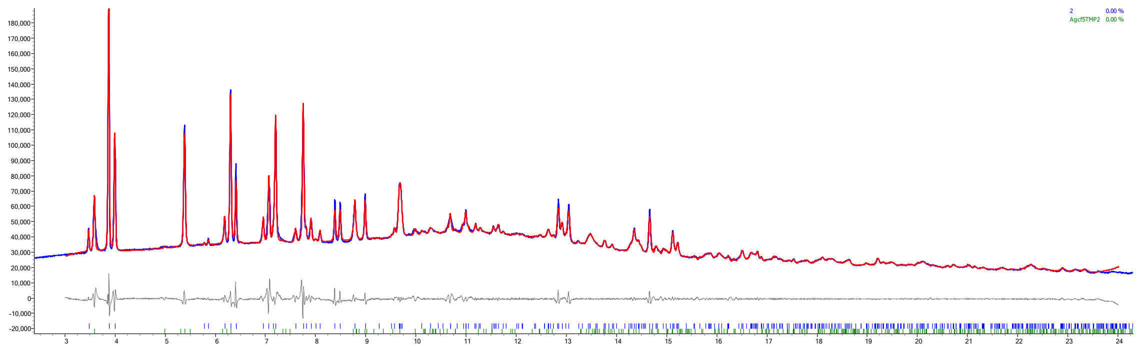

Supplementary Figure 79. Observed (blue) and calculated (red) profiles and difference plot [$I_{obs}–I_{calc}$] (grey) of the Pawley refinement of Pattern 23 at 1.4 bar CO_2 (T = 295 K) ($3.0 \leq 2\theta \leq 24.0$ °; d_{min} = 2.03 Å) (R_{wp} = 2.976, R_{wp}' = 14.022) for $\mathbf{2^{HT}}$. Minor-phase impurity phase of [$Ag_4(O_2C(CF_2)_3CF_3)_4(TMP)_2$] is also fitted by Pawley methods.

4.3 *In situ* PXRD study of coordination polymer 4 exposure to CO_2 gas pressure

The structural changes associated with CO_2 absorption were explored for coordination polymer **4** using PXRD (λ= 0.826179(1) Å) at beamline I11 at Diamond Light Source.[S13] 10s scans were collected using a PSD detector, during which the capillary was oscillated about its axis through an angle of approx. 35 °. All data were collected at room temperature (295 K) at each in a sequence of CO_2 gas pressures (CO_2: 0, 1.13, 10.25, 14.54, 19.99, 24.40, 30.01, 49.90, 28.60, 25.05, 20.10, 10.31, 1.04 bar), with 30 minutes intervals in between measurements for equilibration of the structure along the capillary at each pressure. The powder patterns were indexed using *TOPAS*.[S10] A one or two-phase refinement was implemented in *TOPAS*, which comprised a Pawley refinement[S11] using the unit cell parameters of **4**, $\mathbf{4_B^{CO2}}$ or $\mathbf{4_C^{CO2}}$ (depending on the pressure for the CO_2 gas uptake. Unit cell parameters at each gas pressure are provided in Supplementary Table 16 and summarized in Supplementary Figure 80. Fits for individual PXRD patterns are provided in Supplementary Figures 81-94.

An initial PXRD scan under vacuum at 298 K show the presence of coordination polymer **4**. The CO_2 pressure was subsequently increased to 1.13, 10.25, 14.54 and 19.99 bars and new scans were collected at room temperature. Pawley refinement confirmed the presence of coordination polymer **4** across this range of pressures. The CO_2 pressure was increased to 24.40 bars and Pawley refinement confirmed the presence of a mixture of coordination polymers **4** and $\mathbf{4_B^{CO2}}$. The pressure was further increased to 30.01 bar and a new pattern was collected at room temperature. Pawley refinement confirmed the presence exclusively of coordination polymer $\mathbf{4_B^{CO2}}$. The designation of the latter being made based on the marked increase in unit cell volume relative to **4** (Supplementary Table 16). The CO_2 pressure was further increased to 49.90 bar and a new pattern was collected at room temperature. Pawley refinement confirmed the presence of coordination polymer $\mathbf{4_C^{CO2}}$. The designation of the latter being made based on the marked increase in unit cell volume relative to **4** and $\mathbf{4_B^{CO2}}$ (Supplementary Table 16). A sequence of pressure reductions was then undertaken to explore the reversibility of the processes via CO_2 desorption. A sequence of scans was measured at room temperature at CO_2 pressures of 28.60 and 25.05. Pawley refinement of the patterns at 28.60 and 25.05 bar CO_2 showed the reformation of coordination polymer $\mathbf{4_B^{CO2}}$, confirming the reversibility of the phase change. Then, the CO_2 pressure was further reduced to 20.10 and Pawley refinement confirmed the presence of a mixture of coordination polymers **4** and $\mathbf{4_B^{CO2}}$. Then, the pressure was further decreased to 10.31 and 1.04 bar and a series of scans were measured at room temperature. Full desorption of the CO_2 gas was confirmed by

Pawley refinement of patterns measured at 10.31 and 1.04 bar of CO_2 at room temperature, which showed the presence of coordination polymer **4**.

Supplementary Table 16. Unit values from Pawley refinement for compounds **4** and $\mathbf{4_B}^{\mathbf{CO2}}$ under CO_2 gas pressure at 295 K

Pressure (bar)	*Compound (z)*	*a (Å)*	*b (Å)*	*c (Å)*	*α (°)*	*β (°)*	*γ (°)*	*V (Å³)*	*V (Å³)/z*
0	**4** (1)	8.620(2)	14.801(3)	16.795(4)	111.535(5)	103.588(8)	90.867(8)	1925.2(7)	1925.2(7)
1.13	**4** (1)	8.6157(8)	14.834(1)	16.923(2)	111.575(6)	105.987(9)	90,754(3)	1924.1(3)	1924.1(3)
10.25	**4** (1)	8.6187(4)	14.782(1)	16.994(1)	112.071(3)	105.876(4)	91.066(2)	1921.2(2)	1921.2(2)
14.54	**4** (1)	8.6154(5)	14.851(1)	16.812(1)	111.494(5)	103.415(6)	91.057(4)	1934.1(3)	1934.1(3)
19.99	**4** (1)	8.6310(5)	14.7883(8)	16.8204(8)	111.699(2)	103.500(4)	91.085(2)	1926.5(2)	1926.5(2)
24.40	**4** (1) (and $\mathbf{4_B}^{\mathbf{CO2}}$)	8.622(2)	14.823(4)	16.866(4)	111.44(2)	103.39(2)	91.013(2)	1939.4(9)	1939.4(9)
30.01	$\mathbf{4_B}^{\mathbf{CO2}}$ (2)	16.982(3)	15.392(2)	16.781(2)	97.24(1)	72.41(1)	90.58(2)	4146(1)	2073(1)
49.90	$\mathbf{4_C}^{\mathbf{CO2}}$ (4)	17.229(3)	19.684(4)	28.649(5)	96.613(8)	78.162(9)	107.35(1)	9061(3)	2265(1)
28.60	$\mathbf{4_B}^{\mathbf{CO2}}$ (2)	17.0360(9)	15.3835(7)	16.8005(8)	97.178(4)	71.960(6)	90.679(5)	4152.0(4)	2076(1)
25.05	$\mathbf{4_B}^{\mathbf{CO2}}$ (2)	17,000(1)	15.373(1)	16.744(1)	97.054(6)	72.180(7)	90.523(7)	4132.7(6)	2066(1)
20.10	**4** (1) (and $\mathbf{4_B}^{\mathbf{CO2}}$)	8.6292(5)	14.788(1)	16.848(1)	111,812(3)	103.537(5)	91,104(5)	1927.2(2)	1927.2(2)
10.31	**4** (1)	8.6227(6)	14.833(1)	16.802(1)	111,490(4)	103.493(7)	91,044(5)	1931.7(3)	1931.7(3)
1.04	**4** (1)	8.6275(3)	14.7595(6)	16.8203(6)	111.658(2)	103.561(3)	91.081(2)	1921.9(1)	1921.9(1)

Overall, the *in-situ* powder X-ray diffraction study shows **4** → $\mathbf{4_B}^{\mathbf{CO2}}$ and $\mathbf{4_B}^{\mathbf{CO2}}$ → $\mathbf{4_C}^{\mathbf{CO2}}$ transition between 24.4-30 and 30-49.9 bar CO_2 pressure (Supplementary Table 16, Supplementary Figure 80). The unit cell volumes of $\mathbf{4_B}^{\mathbf{CO2}}$ and $\mathbf{4_C}^{\mathbf{CO2}}$ are more than twice or quadruple, respectively, that of the initial phase **4** as the asymmetric unit of $\mathbf{4_B}^{\mathbf{CO2}}$ and $\mathbf{4_C}^{\mathbf{CO2}}$ contains twice or quadruple, respectively, as much of the polymer as that of **4**. The expansion due to CO_2 adsorption into the crystals is more effectively quantified by considering the change in volume per formula unit, $\Delta V/Z = 134$ Å^3 from p_{CO2} = 24.4 bar to $p_{CO2} = 30$ bar and $\Delta V/Z = 326$ Å^3 from p_{CO2} = 24.4 bar to $p_{CO2} = 49.9$ bar.

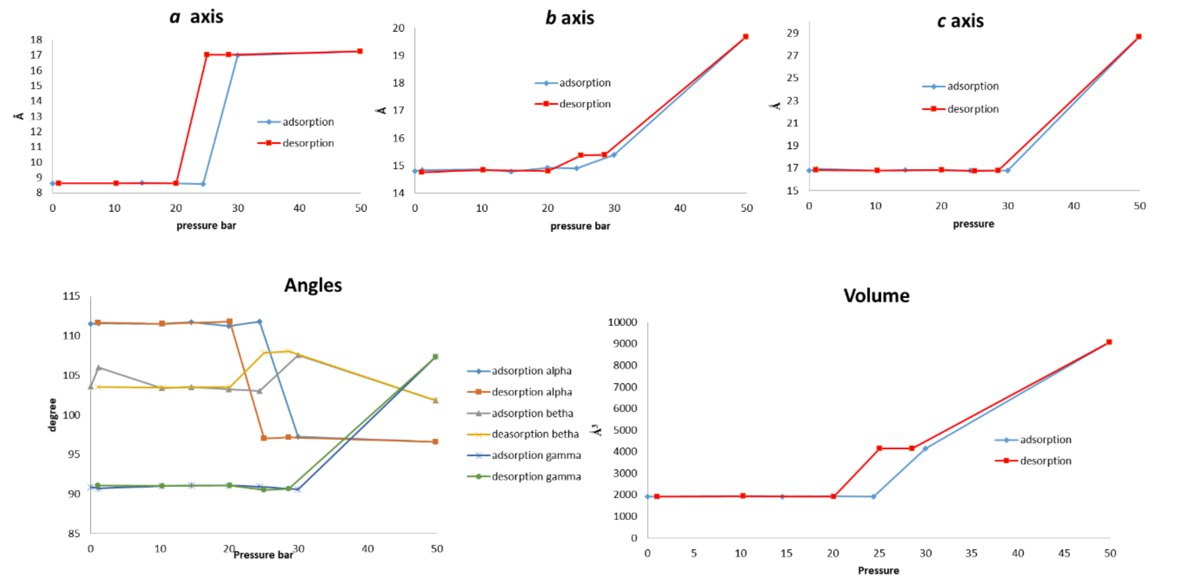

Supplementary Figure 80. Unit cell values from Pawley refinement of PXRD patterns for coordination polymer **4** (**4**, **4**$_B^{CO2}$ or **4**$_C^{CO2}$ depending on CO_2 gas pressure). PXRD data were collected sequentially at CO_2 gas pressures of 0, 1.13, 10.25, 14.54, 19.99, 24.40, 30.01, 49.90, 28.60, 25.05, 20.10, 10.31, 1.04 bar at room temperature

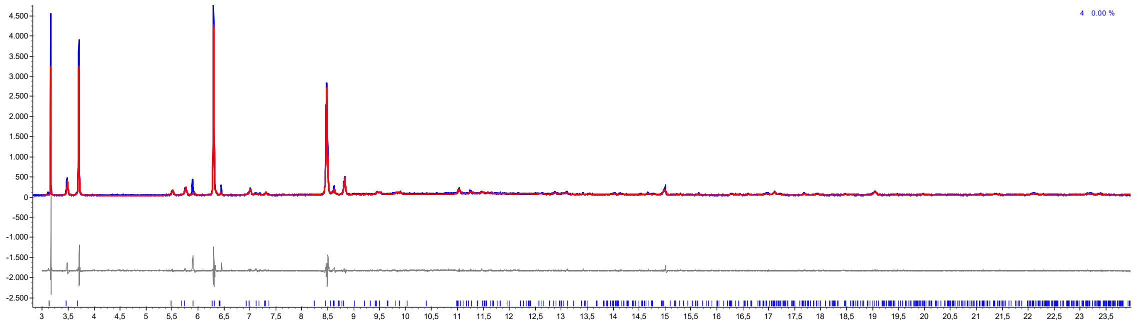

Supplementary Figure 81. Observed (blue) and calculated (red) profiles and difference plot [I_{obs}–I_{calc}] (grey) of the Pawley refinement of Pattern 1 at 10^{-6} mbar CO_2 (T = 295 K) ($3.0 \leq 2\theta \leq 24.0$ °; d_{min} = 2.03 Å) (R_{wp} = 16.728, R_{wp}' = 26.698).

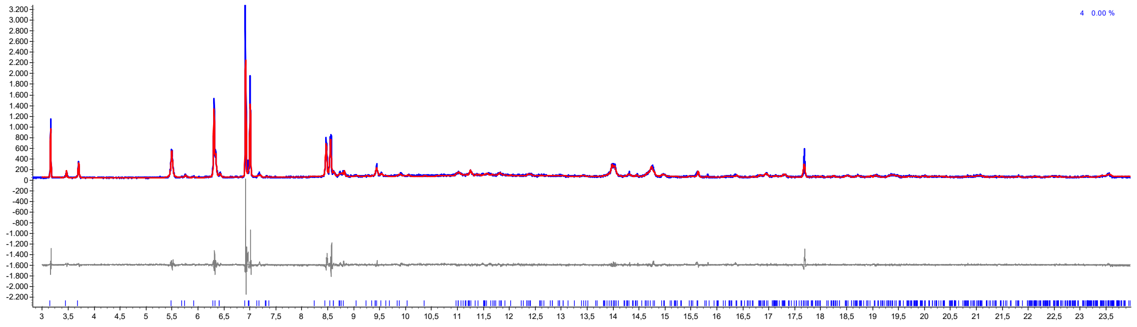

Supplementary Figure 82. Observed (blue) and calculated (red) profiles and difference plot [I_{obs}-I_{calc}] (grey) of the Pawley refinement of Pattern 2 at 1.13 mbar CO_2 (T = 295 K) ($3.0 \leq 2\theta \leq 24.0$ °; d_{min} = 2.03 Å) (R_{wp} = 15.563, R_{wp}' = 31.576).

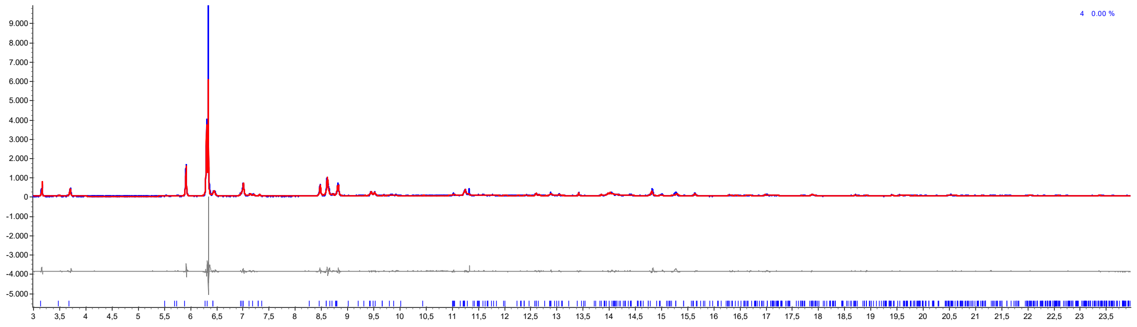

Supplementary Figure 83. Observed (blue) and calculated (red) profiles and difference plot [I_{obs}-I_{calc}] (grey) of the Pawley refinement of Pattern 3 at 10.25 bar CO_2 (T = 295 K) ($3.0 \leq 2\theta \leq 24.0$ °; d_{min} = 2.03 Å) (R_{wp} = 16.878, R_{wp}' = 27.294).

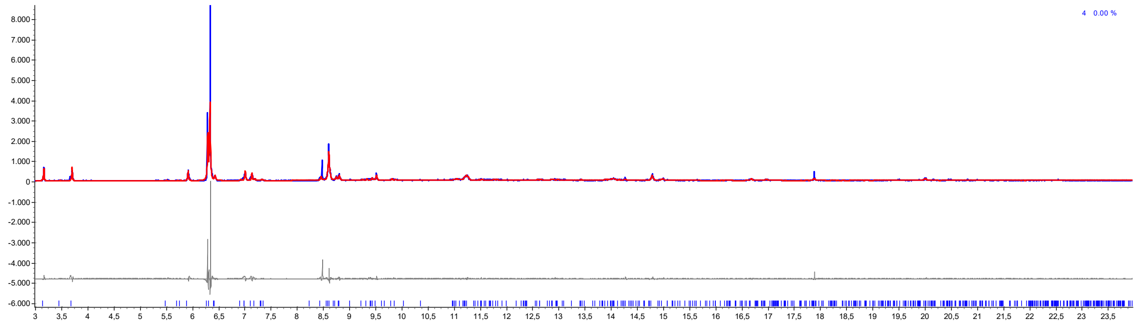

Supplementary Figure 84. Observed (blue) and calculated (red) profiles and difference plot [I_{obs}-I_{calc}] (grey) of the Pawley refinement of Pattern 4 at 14.54 bar CO_2 (T = 295 K) (3.0 ≤ 2θ ≤ 24.0 °; d_{min} = 2.03 Å) (R_{wp} = 20.257, R_{wp}' = 40.226).

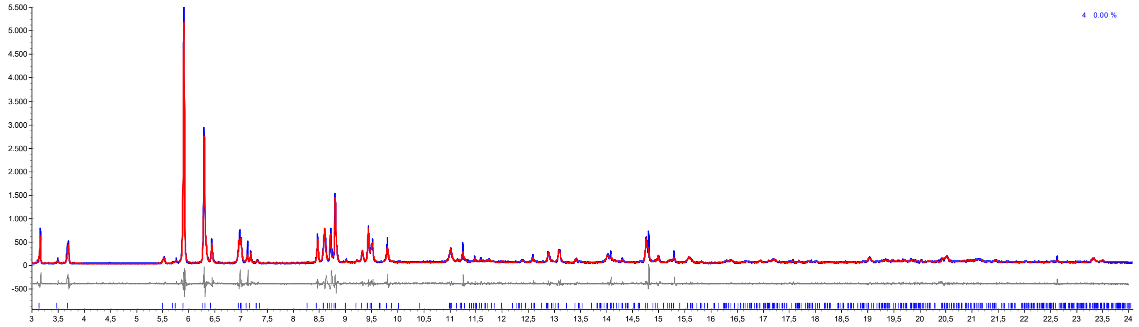

Supplementary Figure 85. Observed (blue) and calculated (red) profiles and difference plot [I_{obs}-I_{calc}] (grey) of the Pawley refinement of Pattern 5 at 19.99 bar CO_2 (T = 295 K) (3.0 ≤ 2θ ≤ 24.0 °; d_{min} = 2.03 Å) (R_{wp} = 14.261, R_{wp}' = 24.325).

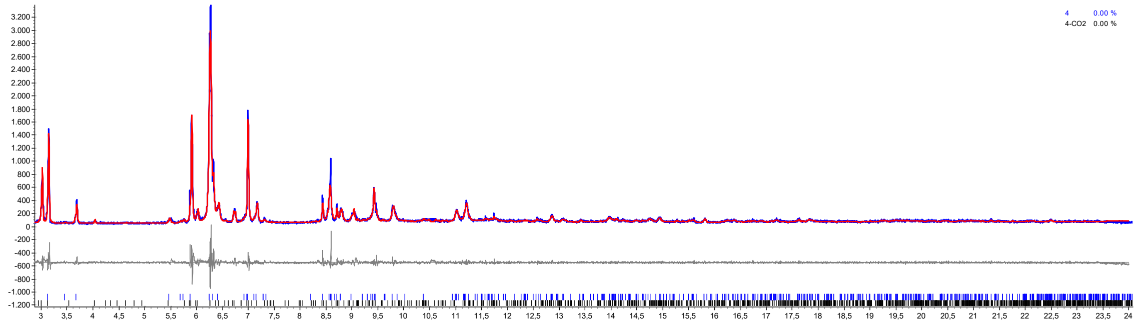

Supplementary Figure 86. Observed (blue) and calculated (red) profiles and difference plot [I_{obs}-I_{calc}] (grey) of the Pawley refinement of Pattern 6 at 24.40 bar CO_2 (T = 295 K) (3.0 ≤ 2θ ≤ 24.0 °; d_{min} = 2.03 Å) (R_{wp} = 12.133, R_{wp}' = 21.174).

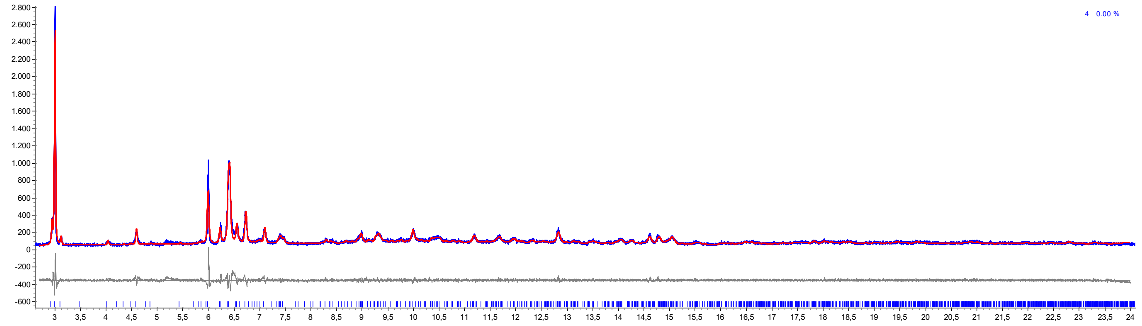

Supplementary Figure 87. Observed (blue) and calculated (red) profiles and difference plot [I_{obs}-I_{calc}] (grey) of the Pawley refinement of Pattern 7 at 30.01 bar CO_2 (T = 295 K) ($3.0 \leq 2\theta \leq 24.0$ °; d_{min} = 2.03 Å) (R_{wp} = 11.821, R_{wp}' = 19.268).

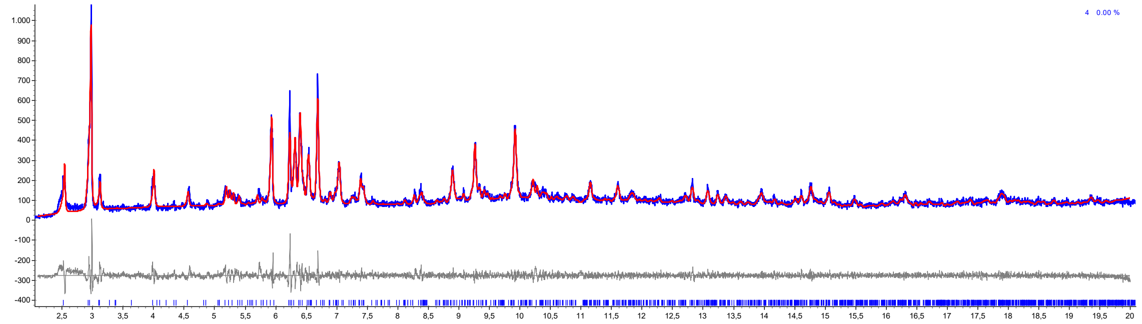

Supplementary Figure 88. Observed (blue) and calculated (red) profiles and difference plot [I_{obs}-I_{calc}] (grey) of the Pawley refinement of Pattern 8 at 49.90 bar CO_2 (T = 295 K) ($3.0 \leq 2\theta \leq 24.0$ °; d_{min} = 2.03 Å) (R_{wp} = 11.704, R_{wp}' = 24.910).

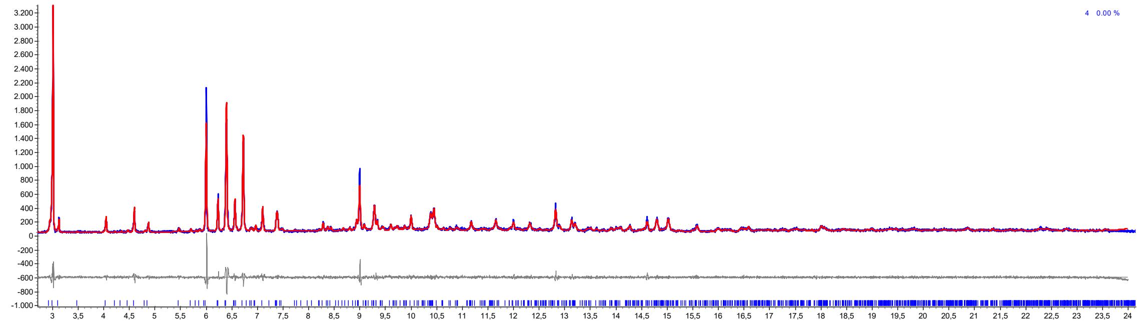

Supplementary Figure 89. Observed (blue) and calculated (red) profiles and difference plot [I_{obs}-I_{calc}] (grey) of the Pawley refinement of Pattern 9 at 28.60 bar CO_2 (T = 295 K) ($3.0 \leq 2\theta \leq 24.0$ °; d_{min} = 2.03 Å) (R_{wp} = 11.548, R_{wp}' = 17.373).

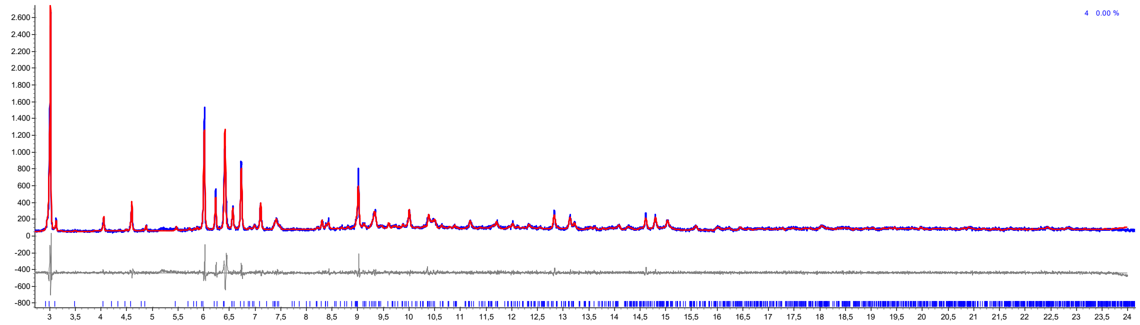

Supplementary Figure 90. Observed (blue) and calculated (red) profiles and difference plot [I_{obs}-I_{calc}] (grey) of the Pawley refinement of Pattern 10 at 25.05 bar CO_2 (T = 295 K) (3.0 ≤ 2θ ≤ 24.0 °; d_{min} = 2.03 Å) (R_{wp} = 11.539, R_{wp}' = 17.363).

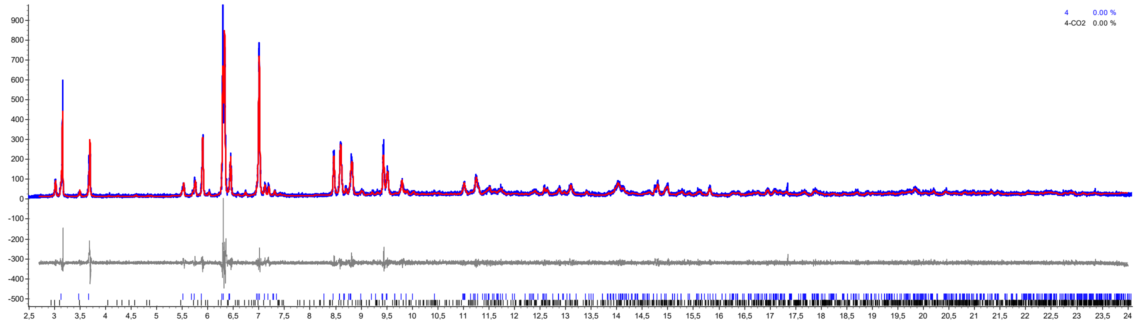

Supplementary Figure 91. Observed (blue) and calculated (red) profiles and difference plot [I_{obs}-I_{calc}] (grey) of the Pawley refinement of Pattern 11 at 20.10 bar CO_2 (T = 295 K) (3.0 ≤ 2θ ≤ 24.0 °; d_{min} = 2.03 Å) (R_{wp} = 16.717, R_{wp}' = 25.469).

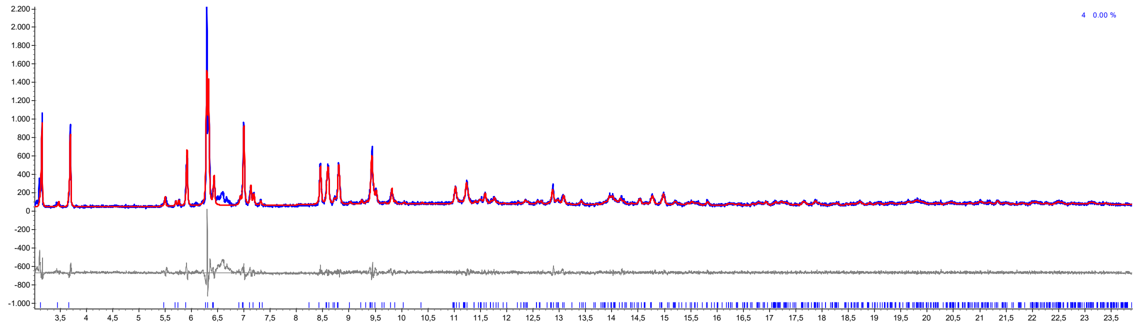

Supplementary Figure 92. Observed (blue) and calculated (red) profiles and difference plot [I_{obs}-I_{calc}] (grey) of the Pawley refinement of Pattern 12 at 10.31 bar CO_2 (T = 295 K) (3.0 ≤ 2θ ≤ 24.0 °; d_{min} = 2.03 Å) (R_{wp} = 14.615, R_{wp}' = 30.923).

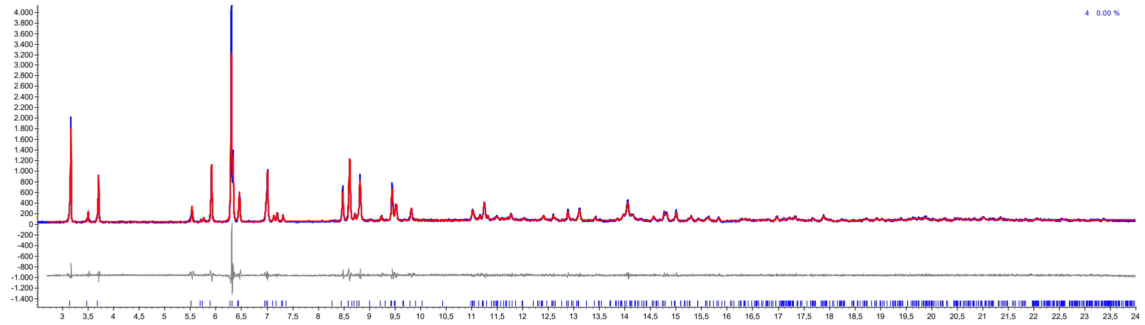

Supplementary Figure 93. Observed (blue) and calculated (red) profiles and difference plot [I_{obs}-I_{calc}] (grey) of the Pawley refinement of Pattern 13 at 1.04 bar CO_2 (T = 295 K) (3.0 ≤ 2θ ≤ 24.0 °; d_{min} = 2.03 Å) (R_{wp} = 12.479, R_{wp}' = 22.589).

4.4. *In situ* PXRD study of coordination polymer 5 exposure to CO_2 and CH_4 gas pressure

The structural changes associated with CO_2 and CH_4 adsorption were explored for coordination polymer **5** using PXRD (λ= 0.826179(1) Å) at beamline I11 at Diamond Light Source.[S13] 10 s scans were collected using a PSD detector, during which the capillary was oscillated about its axis through an angle of approx. 35 °. Data were collected at room temperature (295 K) at each in a sequence of CO_2 gas pressures, (CO_2: 0, 1.02, 9.89, 14.89, 20.17, 24.24, 28.99, 38.53, 48.37, 49.41, 20.71, 15.60, 9.72 and 1.04 bar). In a separate experiment, data were collected at room temperature (295 K) and 180 K at each in a sequence of CH_4 gas pressures, (295 K, air; 180 K, CH_4: 4.99, 9.63, 14.24, 18.63, 14.19, 10.14, 5.23 and 1.05 bar). Intervals of 30 mins between measurements were included for equilibration of the structure along the capillary at each pressure. The powder patterns were indexed using *TOPAS*.[S10] For the study under CO_2 gas atmospheres, a one or two-phase refinement was implemented in *TOPAS*, which comprised a Pawley refinement[S11] using the unit cell parameters of **5** and $\mathbf{5_B^{CO2}}$ (depending on the pressure for the CO_2 gas uptake). Unit cell parameters at each gas pressure are provided in Supplementary Table 17. Fits for individual PXRD patterns are provided in Supplementary Figures 94-107. For the investigation of CH_4 adsorption, an initial PXRD scan on coordination polymer at room temperature in air shows the presence of coordination polymer **5** through fitting by Pawley refinement. The capillary was cooled to 180 K and the air was evacuated for 20 mins. The CH_4 pressure was set to 4.99 bar and a scan was collected suggesting the formation of a low-temperature polymorph of **5** ($\mathbf{5^{LT}}$). The designation of $\mathbf{5^{LT}}$ was made based on the marked decrease in unit cell volume relative to **5** (Supplementary Table 18). A one or two-phase refinement was implemented in *TOPAS*, which comprised a Pawley refinement[S11] using the unit cell parameters of **5** and $\mathbf{5^{LT}}$ (depending on the temperature). Unit cell parameters at each gas pressure are provided in Supplementary Table 18. Fits for individual PXRD patterns are provided in Supplementary Figures 108-117.

4.4.1 *In situ* PXRD study of coordination polymer 5 exposure to CO_2 gas under pressure

An initial PXRD scan under vacuum and 295 K confirmed the presence of coordination polymer **5** through Pawley refinement. The CO_2 pressure was subsequently increased to 1.02, 9.89, 14.89, 20.17, 24.24, 28.99, 38.53 and 48.37 bar and new scans were collected at room temperature. Pawley refinement showed only the presence of coordination polymer **5** with a very small increase in unit cell volume over this pressure range ($\Delta V = \Delta V/Z$ = 24.2(2) Å^3). The CO_2 pressure was gradually increased to 49.41 bar and a new pattern collected at room temperature. Pawley refinement confirmed the presence of a new form of CO_2-containing coordination polymer, designated $\mathbf{5_B^{CO2}}$ based on the marked increase in unit cell volume relative to **5** (Supplementary Table 17). The volume of the new phase $\mathbf{5_B^{CO2}}$ is more than four times the volume of the initial phase **5** due to a change in symmetry from Z = 1 (**5**) to Z = 4 ($\mathbf{5_B^{CO2}}$).

The increase in volume per formula unit is $\Delta V/Z$ = 235(8) Å^3 (approx. 11%). A sequence of pressure reductions was then undertaken to explore the reversibility of the processes via CO_2 desorption. PXRD scans were measured at room temperature at CO_2 pressures of 20.71, 15.60, 9.72 and 1.04 bar. Pawley refinement of the pattern at 20.71 bar CO_2 and showed the reformation of coordination polymer **5**, confirming the reversibility of the transformation. Pawley refinement for measurements throughout this final pressure range confirmed the presence only of coordination polymer **5**.

Supplementary Table 17. Unit cell values of compound **5** obtained from Pawley refinements of PXRD data in the CO_2 adsorption experiment at 295 K

Pressure (bar)	*Compound (Z)*	*a (Å)*	*b (Å)*	*c (Å)*	*α (°)*	*β (°)*	*γ (°)*	*V (Å³)*	*V/Z (Å³)*
0	**5** (1)	8.6138(4)	14.7410(7)	17.9381(7)	110.734(4)	101.983(4)	90.570(2)	2075.1(2)	2075.1(2)
1.02	**5** (1)	8.5449(7)	14.819(1)	17.772(2)	110.421(7)	100,873(7)	90.995(8)	2062.6(4)	2062.6(4)
9.89	**5** (1)	8.6156(3)	14.7646(7)	17.9598(6)	110.727(2)	101.907(3)	90.600(2)	2082.0(2)	2082.0(2)
14.89	**5** (1)	8.6090(5)	14.809(1)	18.1951(1)	110.385(5)	105.415(7)	90,497(4)	2087.1(3)	2087.1(3)
20.17	**5** (1)	8.6191(4)	14.7662(9)	18.2763(9)	110.708(3)	105.763(4)	90.620(3)	2084.5(2)	2084.5(2)
24.24	**5** (1)	8.6218(4)	14.7674(7)	18.2743(8)	110.729(3)	105.597(4)	90.634(2)	2086.8(2)	2086.8(2)
28.99	**5** (1)	8.6192(4)	14.7978(8)	18.2808(8)	110.756(4)	105.530(4)	90.641(3)	2091.6(2)	2091.6(2)
38.53	**5** (1)	8.6277(5)	14.8013(8)	18.2998(9)	110.742(3)	105.553(4)	90.706(3)	2096.5(2)	2096.5(2)
48.37	**5** (1)	8.6268(4)	14.8016(8)	18.3259(9)	110.779(3)	105.520(5)	90.739(3)	2099.3(2)	2099.3(2)
49.41	$\mathbf{5_B}^{CO2}$ (4)	23.339(8)	20.327(9)	20.28(1)	86.38(2)	103.45(3)	89.88(3)	9339(8)	2334(8)
20.71	**5** (1)	8.6245(3)	14.7592(7)	18.274(1)	110.716(4)	105.671(4)	90.681(2)	2085.8(2)	2085.8(2)
15.60	**5** (1)	8.6160(6)	14.800(1)	18.195(1)	110.525(5)	105.346(7)	90.625(4)	2086.8(3)	2086.8(3)
9.72	**5** (1)	8.6250(4)	14.7470(8)	18.2531(8)	110.716(3)	105.662(4)	90.585(2)	2081.5(2)	2081.5(2)
1.04	**5** (1)	8.6086(4)	14.764(1)	18.157(1)	110.354(4)	105.519(5)	90.490(3)	2075.6(2)	2075.6(2)

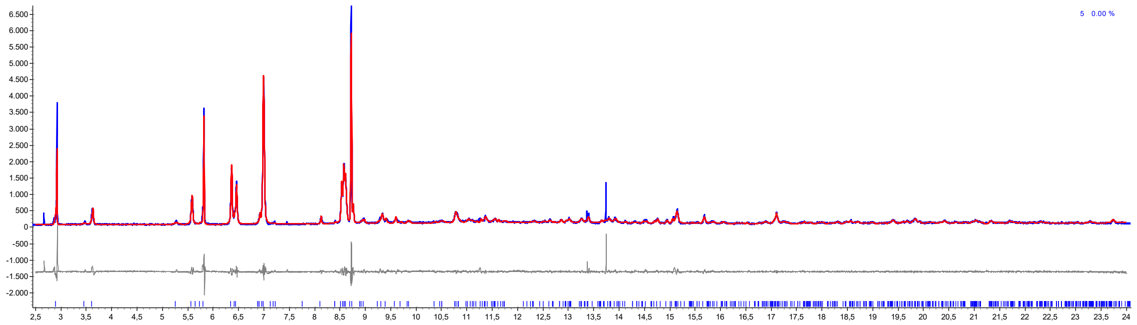

Supplementary Figure 94. Observed (blue) and calculated (red) profiles and difference plot [I_{obs}-I_{calc}] (grey) of the Pawley refinement of Pattern 1 at 10^{-6} mbar (T = 295 K) ($2.5 \leq 2\theta \leq 24.0$ °; d_{min} = 2.03 Å) (R_{wp} = 12.479, R_{wp}' = 22.589).

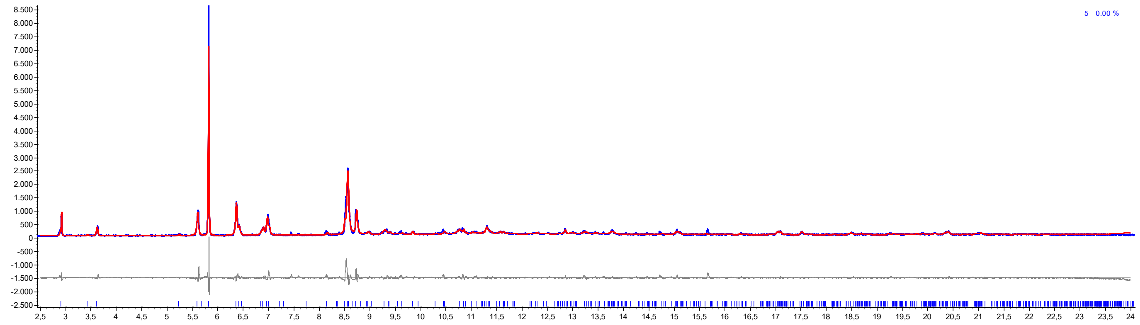

Supplementary Figure 95. Observed (blue) and calculated (red) profiles and difference plot [I_{obs}-I_{calc}] (grey) of the Pawley refinement of Pattern 2 at 1.02 bar CO_2 (T = 295 K) ($2.5 \leq 2\theta \leq 24.0$ °; d_{min} = 2.03 Å) (R_{wp} = 14.622, R_{wp}' = 27.027).

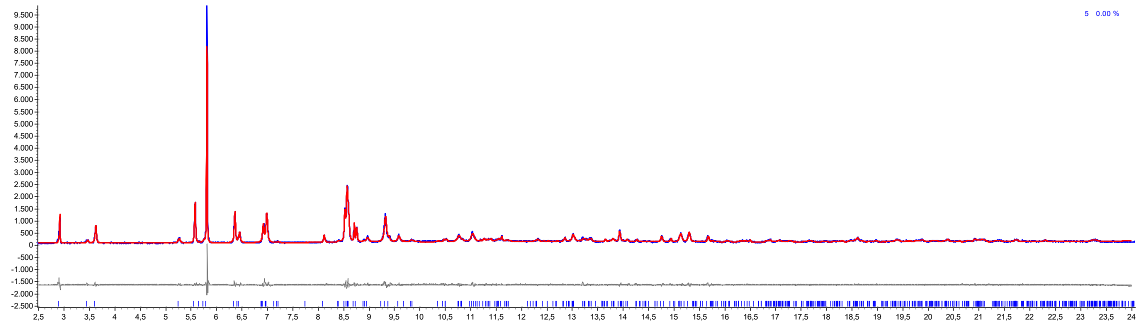

Supplementary Figure 96. Observed (blue) and calculated (red) profiles and difference plot [I_{obs}-I_{calc}] (grey) of the Pawley refinement of Pattern 3 at 9.89 bar CO_2 (T = 295 K) ($2.5 \leq 2\theta \leq 24.0$ °; d_{min} = 2.03 Å) (R_{wp} = 10.010, R_{wp}' = 19.507).

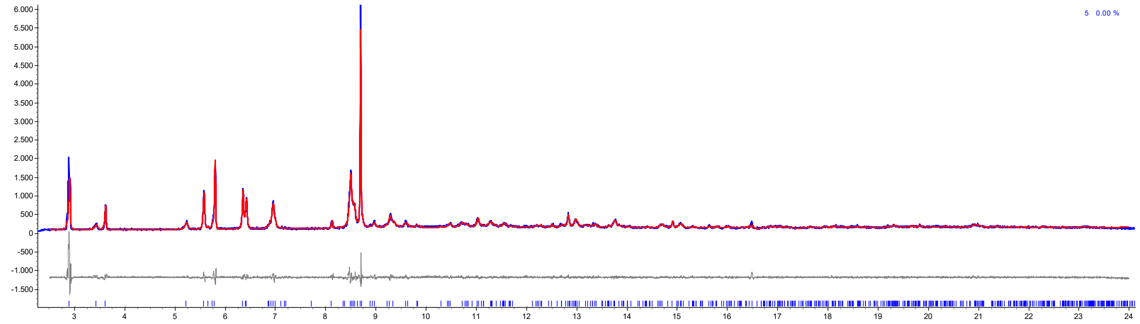

Supplementary Figure 97. Observed (blue) and calculated (red) profiles and difference plot [I_{obs}-I_{calc}] (grey) of the Pawley refinement of Pattern 4 at 14.89 bar CO_2 (T = 295 K) ($2.5 \leq 2\theta \leq 24.0$ °; d_{min} = 2.03 Å) (R_{wp} = 11.028, R_{wp}' = 23.387).

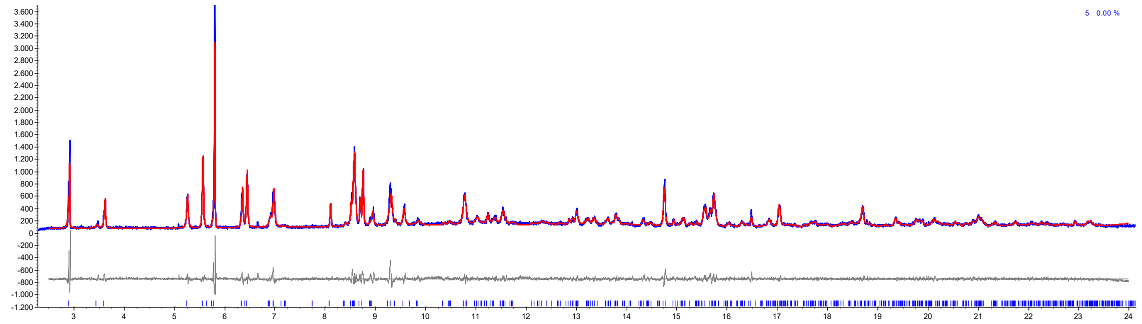

Supplementary Figure 98. Observed (blue) and calculated (red) profiles and difference plot [I_{obs}-I_{calc}] (grey) of the Pawley refinement of Pattern 5 at 20.17 bar CO_2 (T = 295 K) ($2.5 \leq 2\theta \leq 24.0$ °; d_{min} = 2.03 Å) (R_{wp} = 11.202, R_{wp}' = 20.077).

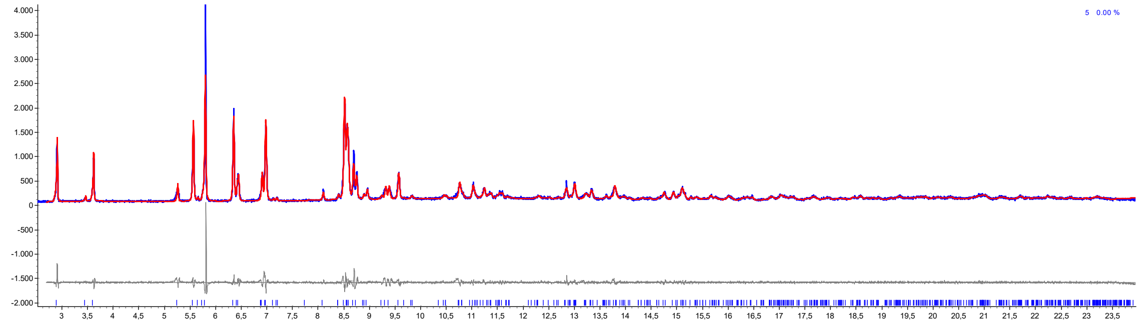

Supplementary Figure 99. Observed (blue) and calculated (red) profiles and difference plot [I_{obs}-I_{calc}] (grey) of the Pawley refinement of Pattern 6 at 24.24 bar CO_2 (T = 295 K) ($2.5 \leq 2\theta \leq 24.0$ °; d_{min} = 2.03 Å) (R_{wp} = 10.768, R_{wp}' = 22.514).

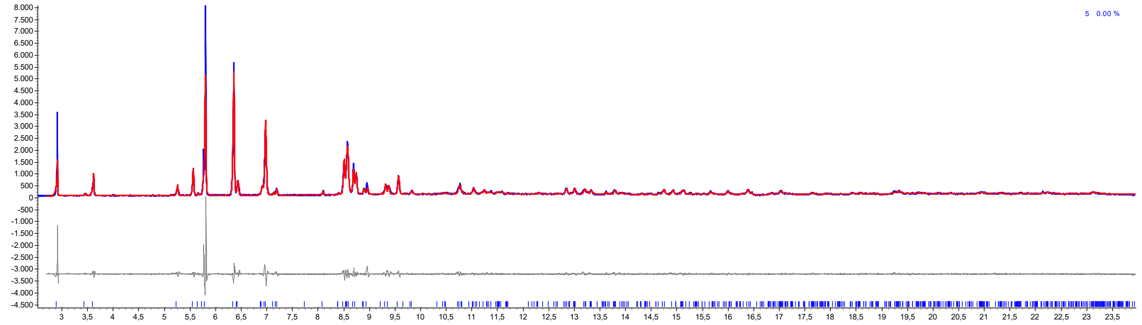

Supplementary Figure 100. Observed (blue) and calculated (red) profiles and difference plot [I_{obs}-I_{calc}] (grey) of the Pawley refinement of Pattern 7 at 28.99 bar CO_2 (T = 295 K) ($2.5 \leq 2\theta \leq 24.0$ °; d_{min} = 2.03 Å) (R_{wp} = 13.972, R_{wp}' = 27.100).

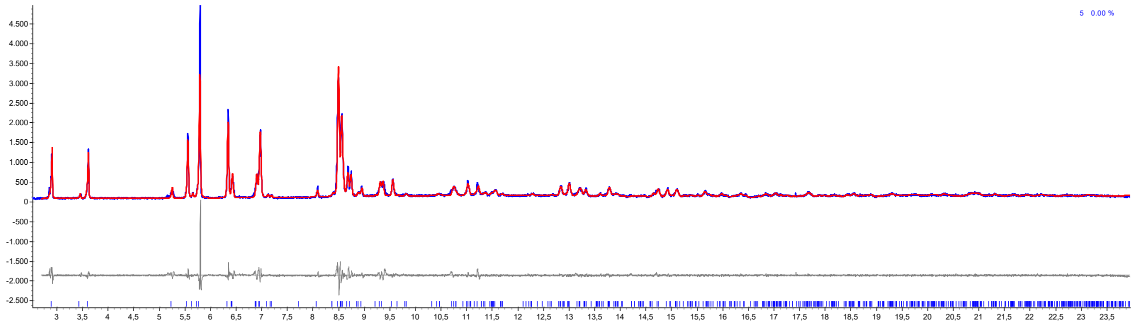

Supplementary Figure 101. Observed (blue) and calculated (red) profiles and difference plot [I_{obs}-I_{calc}] (grey) of the Pawley refinement of Pattern 8 at 38.35 bar CO_2 (T = 295 K) ($2.5 \leq 2\theta \leq 24.0$ °; d_{min} = 2.03 Å) (R_{wp} = 11.146, R_{wp}' = 23.005).

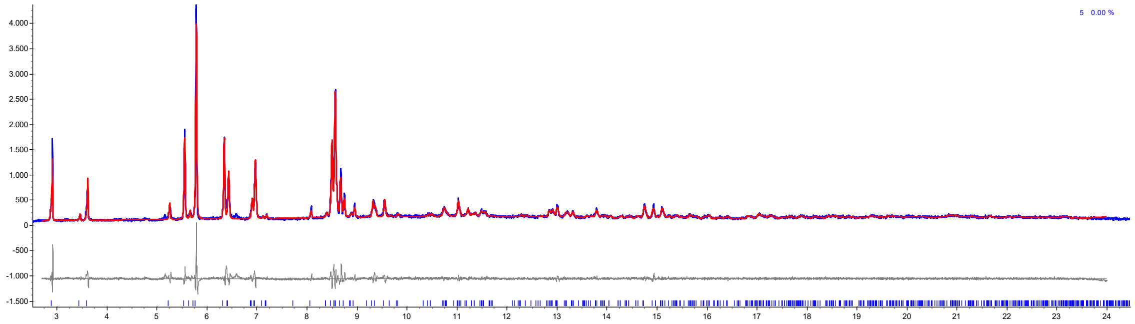

Supplementary Figure 102. Observed (blue) and calculated (red) profiles and difference plot [I_{obs}-I_{calc}] (grey) of the Pawley refinement of Pattern 9 at 48.37 bar CO_2 (T = 295 K) ($2.5 \leq 2\theta \leq 24.0$ °; d_{min} = 2.03 Å) (R_{wp} = 10.544, R_{wp}' = 25.470).

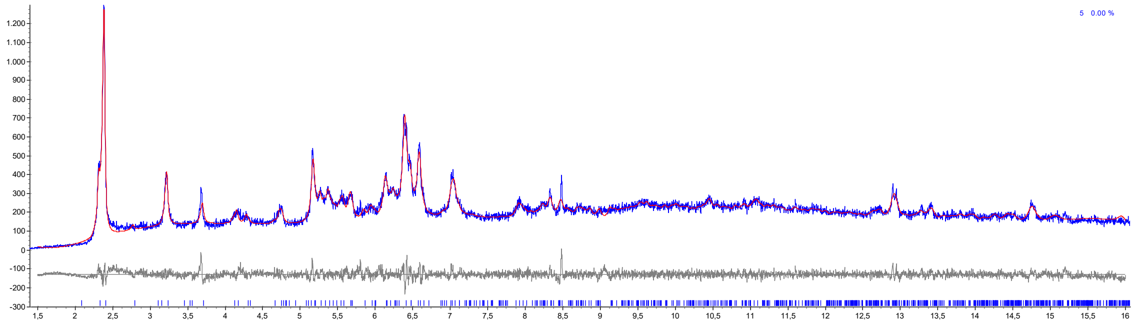

Supplementary Figure 103. Observed (blue) and calculated (red) profiles and difference plot [I_{obs}-I_{calc}] (grey) of the Pawley refinement of Pattern 10 at 49.41 bar CO_2 (T = 295 K) ($2.5 \leq 2\theta \leq 24.0$ °; d_{min} = 2.03 Å) (R_{wp} = 8.0523, R_{wp}' = 16.662).

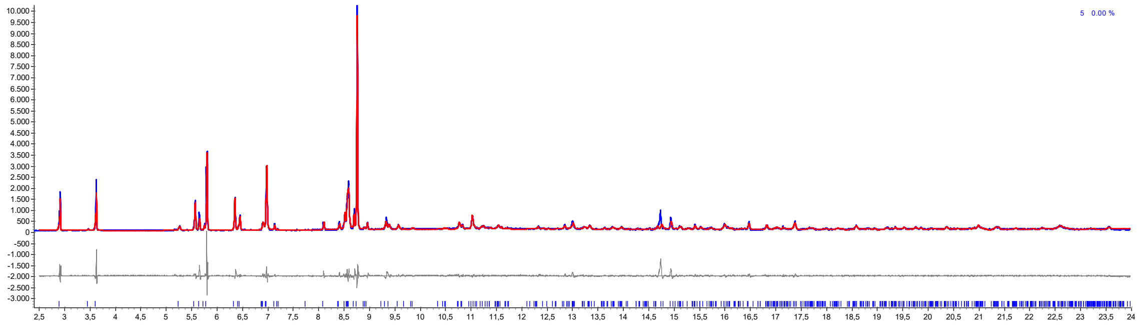

Supplementary Figure 104. Observed (blue) and calculated (red) profiles and difference plot [I_{obs}-I_{calc}] (grey) of the Pawley refinement of Pattern 11 at 20.71 bar CO_2 (T = 295 K) (2.5 ≤ 2θ ≤ 24.0 °; d_{min} = 2.03 Å) (R_{wp} = 15.388, R_{wp}' = 29.389).

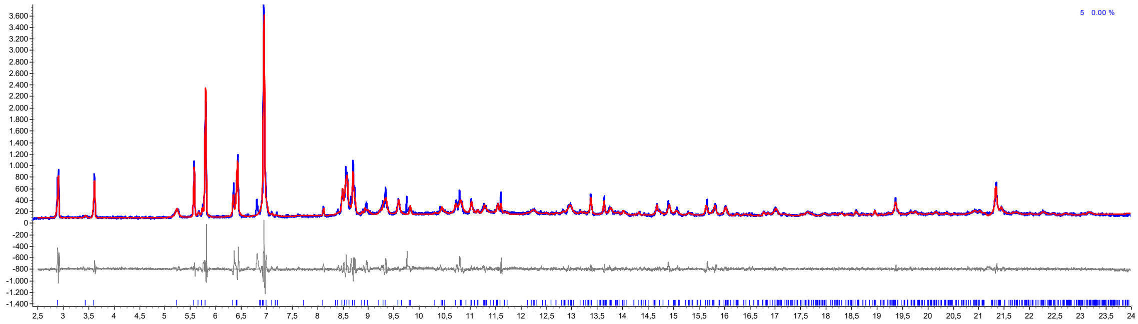

Supplementary Figure 105. Observed (blue) and calculated (red) profiles and difference plot [I_{obs}-I_{calc}] (grey) of the Pawley refinement of Pattern 12 at 15.60 bar CO_2 (T = 295 K) (2.5 ≤ 2θ ≤ 24.0 °; d_{min} = 2.03 Å) (R_{wp} = 12.518, R_{wp}' = 27.373).

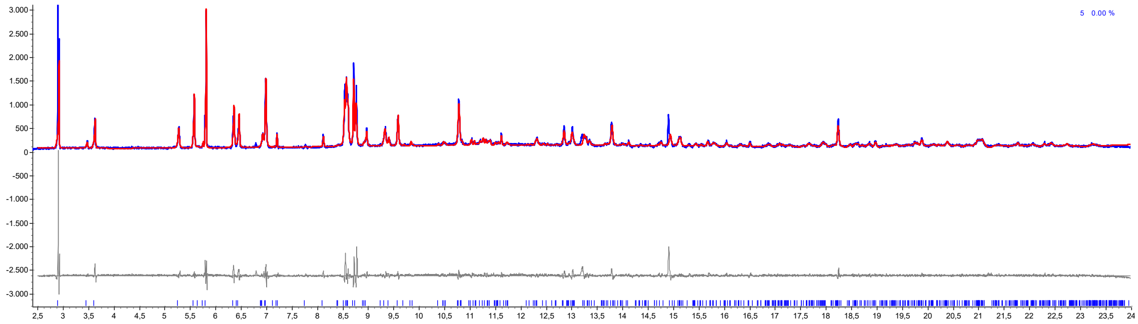

Supplementary Figure 106. Observed (blue) and calculated (red) profiles and difference plot [I_{obs}-I_{calc}] (grey) of the Pawley refinement of Pattern 13 at 9.72 bar CO_2 (T = 295 K) (2.5 ≤ 2θ ≤ 24.0 °; d_{min} = 2.03 Å) (R_{wp} = 15.263, R_{wp}' = 32.182).

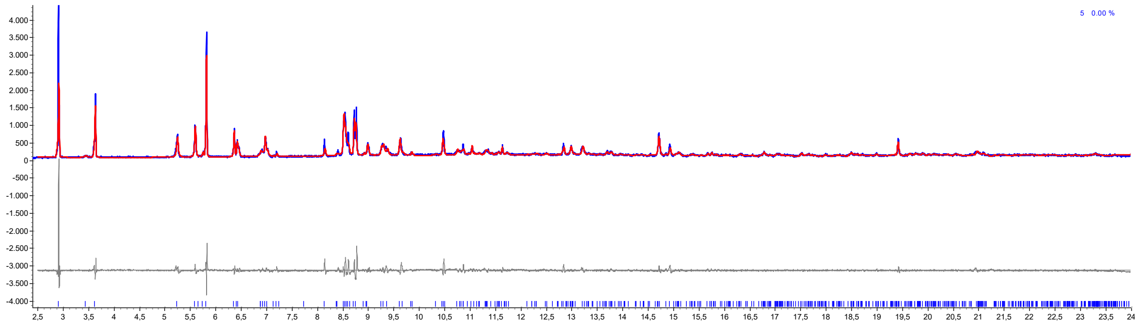

Supplementary Figure 107. Observed (blue) and calculated (red) profiles and difference plot [I_{obs}-I_{calc}] (grey) of the Pawley refinement of Pattern 14 at 1.04 bar CO_2 (T = 295 K) (2.5 ≤ 2θ ≤ 24.0 °; d_{min} = 2.03 Å) (R_{wp} = 14.510, R_{wp}' = 32.569).

4.4.2 *In situ* PXRD study of coordination polymer 5 exposure to CH_4 gas pressure

The *in situ* CH_4 gas sorption powder X-ray diffraction experiment shows a transformation from **5** to $\mathbf{5}^{LT}$ between 295-180 K (Supplementary Table 18). The volume of the new phase $\mathbf{5}^{LT}$ (180 K, 4.99 bar CH_4) is considerably smaller ($\Delta V = -104.4(2)$ Å^3) than the volume of the initial phase **5**, suggesting a thermal contraction upon lowering the temperature. Upon stepwise increase in gas pressure (to p_{CH4} = 18.63 bar) and subsequent stepwise reduction in pressure (to p_{CH4} = 1.05 bar), Pawley fitting of the patterns shows that there is little change in unit cell parameters and volume ($\Delta V < 9$ Å^3) (See Supplementary Table 18 and Supplementary Figures 108-117).

Supplementary Table 18. Unit cell values of compound **5** obtained from Pawley refinements of PXRD data in the CH_4 adsorption experiment at 180 K

Temperature (K)	gas	*Pressure (bar)*	*Compound*	*a (Å)*	*b (Å)*	*c (Å)*	*α (°)*	*β (°)*	*γ (°)*	*V (Å³)*
295	air	1.00	**5**	8.6236(3)	14.7253(6)	17.9628(8)	110.8632(2)	101.945(4)	90.499(2)	2076.8(2)
180	CH_4	4.99	$\mathbf{5}^{LT}$	8.6494(3)	14.2915(7)	17.0482(8)	106.132(3)	102.628(4)	90.491(2)	1972.4(2)
180	CH_4	9.63	$\mathbf{5}^{LT}$	8.6441(6)	14.353(2)	17.066(2)	106.179(8)	102.740(8)	90.513(5)	1980.3(4)
180	CH_4	14.24	$\mathbf{5}^{LT}$	8.6453(3)	14.2936(7)	17.0428(7)	106.140(3)	102.613(4)	90.469(2)	1971.1(2)
180	CH_4	18.63	$\mathbf{5}^{LT}$	8.6425(4)	14.338(1)	17.050(1)	106.233(5)	102.605(5)	90.492(3)	1976.6(2)
180	CH_4	14.19	$\mathbf{5}^{LT}$	8.6476(3)	14.2862(6)	17.0490(7)	106.140(3)	102.616(4)	90,503(2)	1971.4(1)
180	CH_4	10.14	$\mathbf{5}^{LT}$	8.6435(4)	14.335(1)	17.056(1)	106.236(5)	102.622(7)	90.527(3)	1977.0(3)
180	CH_4	5.23	$\mathbf{5}^{LT}$	8.6476(3)	14.2909(7)	17.0475(8)	106.176(3)	102.609(4)	90.511(2)	1971.5(2)
180	CH_4	1.05	$\mathbf{5}^{LT}$	8.6432(3)	14.3422(9)	17.064(1)	106.264(5)	102.618(6)	90.544(3)	1978.6(2)

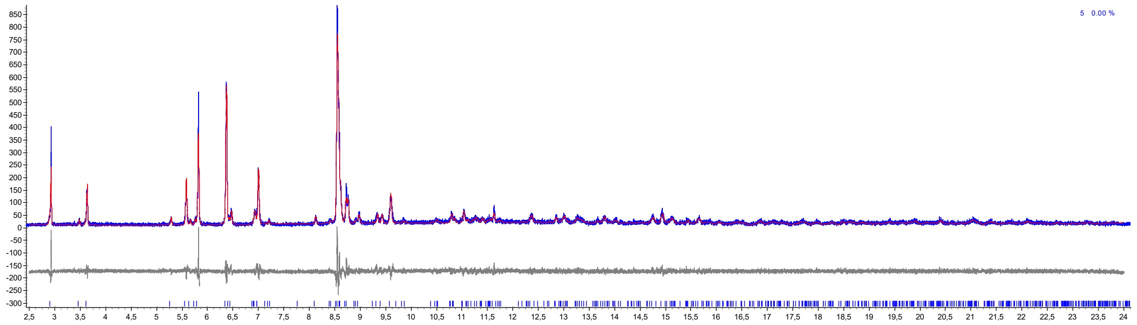

Supplementary Figure 108. Observed (blue) and calculated (red) profiles and difference plot [I_{obs}-I_{calc}] (grey) of the Pawley refinement of Pattern 1 at 1.00 bar air (T = 295 K) (2.5 ≤ 2θ ≤ 24.0 °; d_{min} = 2.03 Å) (R_{wp} = 18.904, R_{wp}' = 33.686).

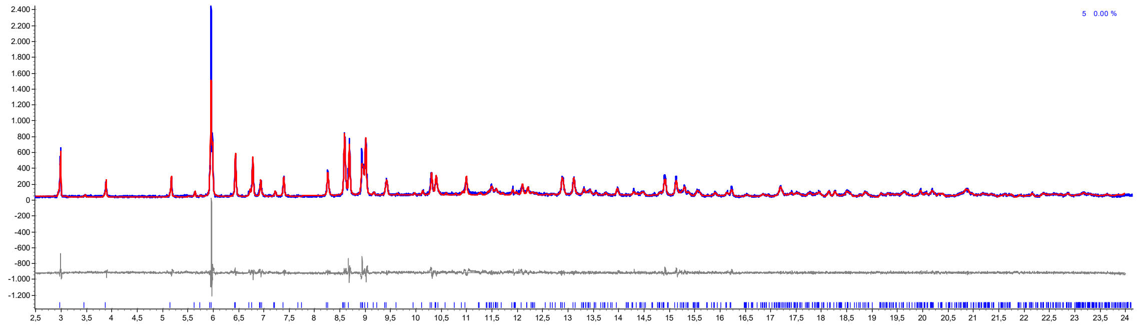

Supplementary Figure 109. Observed (blue) and calculated (red) profiles and difference plot [I_{obs}-I_{calc}] (grey) of the Pawley refinement of Pattern 2 at 4.99 bar CH_4 (T = 180 K) (2.5 ≤ 2θ ≤ 24.0 °; d_{min} = 2.03 Å) (R_{wp} = 14.052, R_{wp}' = 25.092).

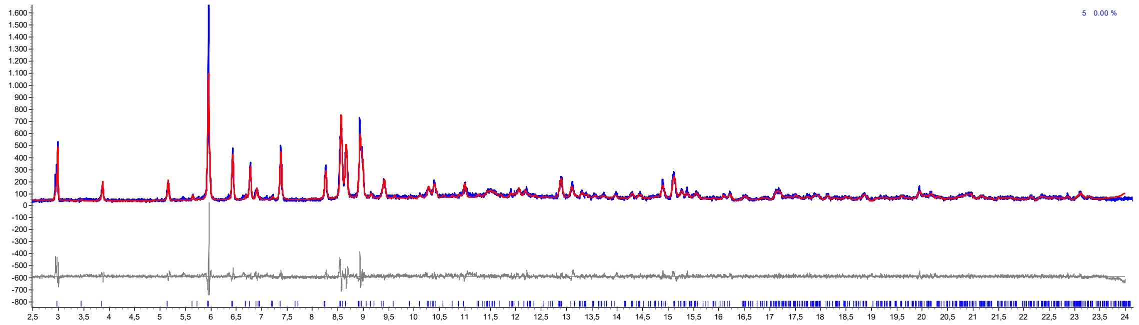

Supplementary Figure 110. Observed (blue) and calculated (red) profiles and difference plot [I_{obs}-I_{calc}] (grey) of the Pawley refinement of Pattern 3 at 9.36 bar CH_4 (T = 180 K) (2.5 ≤ 2θ ≤ 24.0 °; d_{min} = 2.03 Å) (R_{wp} = = 14.687, R_{wp}' = 24.786).

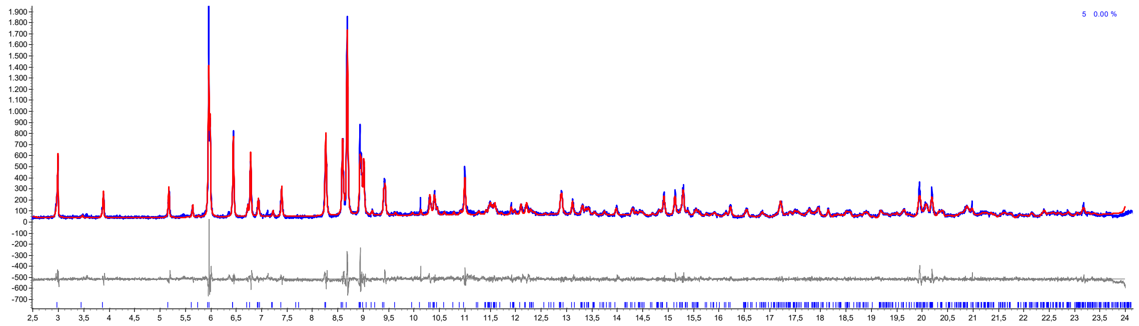

Supplementary Figure 111. Observed (blue) and calculated (red) profiles and difference plot [I_{obs}-I_{calc}] (grey) of the Pawley refinement of Pattern 4 at 14.24 bar CH_4 (T = 180 K) (2.5 ≤ 2θ ≤ 24.0 °; d_{min} = 2.03 Å) (R_{wp} = = 14.063, R_{wp}' = 24.945).

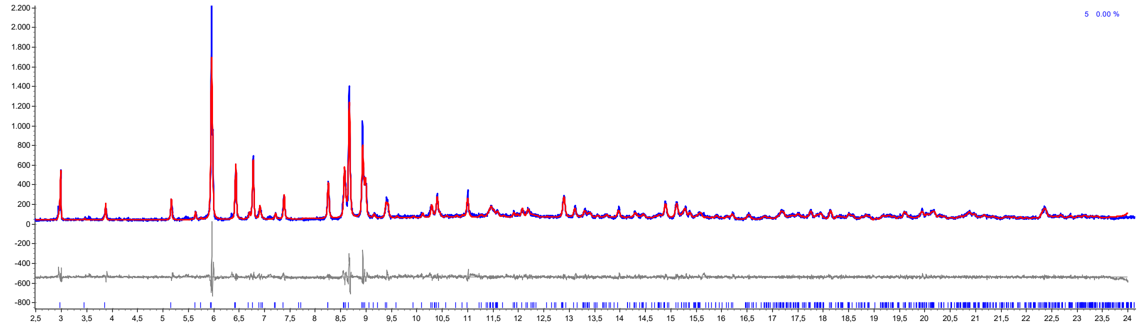

Supplementary Figure 112. Observed (blue) and calculated (red) profiles and difference plot [I_{obs}-I_{calc}] (grey) of the Pawley refinement of Pattern 5 at 18.63 bar CH_4 (T = 180 K) ($2.5 \leq 2\theta \leq 24.0$ °; d_{min} = 2.03 Å) (R_{wp} = = 13.028, R_{wp}' = 22.290).

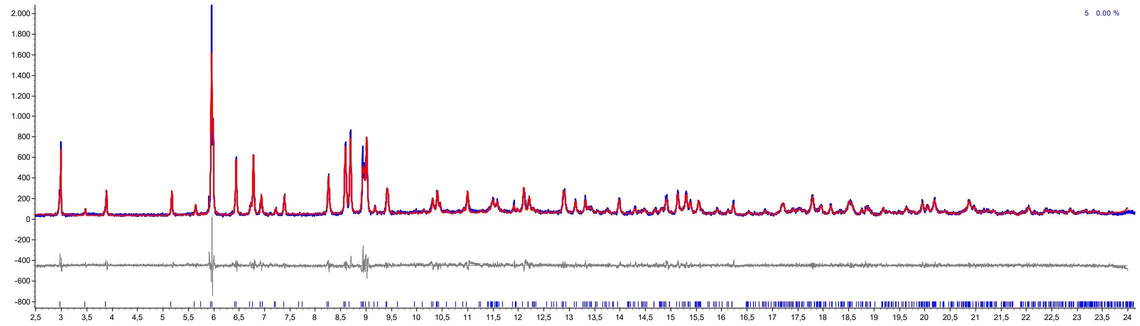

Supplementary Figure 113. Observed (blue) and calculated (red) profiles and difference plot [I_{obs}-I_{calc}] (grey) of the Pawley refinement of Pattern 6 at 14.19 bar CH_4 (T = 180 K) ($2.5 \leq 2\theta \leq 24.0$ °; d_{min} = 2.03 Å) (R_{wp} = = 13.158, R_{wp}' = 22.671).

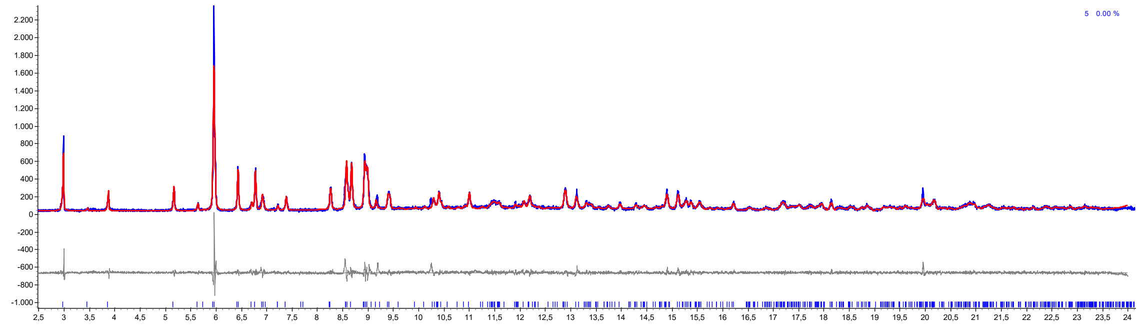

Supplementary Figure 114. Observed (blue) and calculated (red) profiles and difference plot [I_{obs}-I_{calc}] (grey) of the Pawley refinement of Pattern 7 at 10.14 bar CH_4 (T = 180 K) ($2.5 \leq 2\theta \leq 24.0$ °; d_{min} = 2.03 Å) (R_{wp} = = 13.797, R_{wp}' = 21.690).

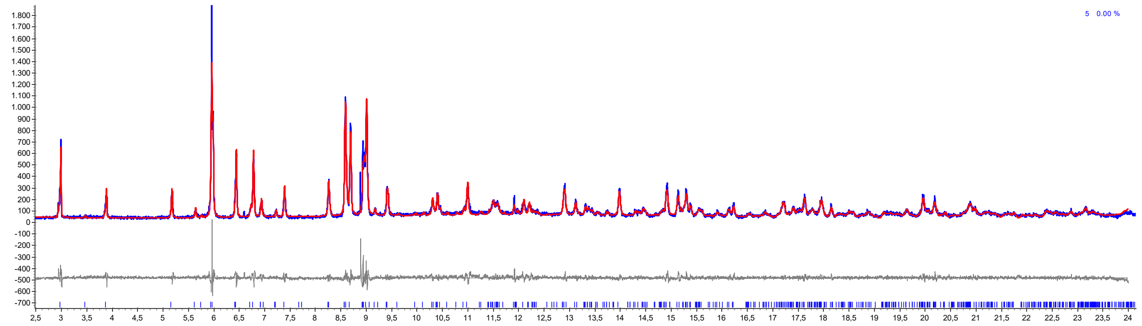

Supplementary Figure 115. Observed (blue) and calculated (red) profiles and difference plot [I_{obs}-I_{calc}] (grey) of the Pawley refinement of Pattern 8 at 5.23 bar CH_4 (T = 180 K) ($2.5 \leq 2\theta \leq 24.0$ °; d_{min} = 2.03 Å) (R_{wp} = = 12.976, R_{wp}' = 22.237).

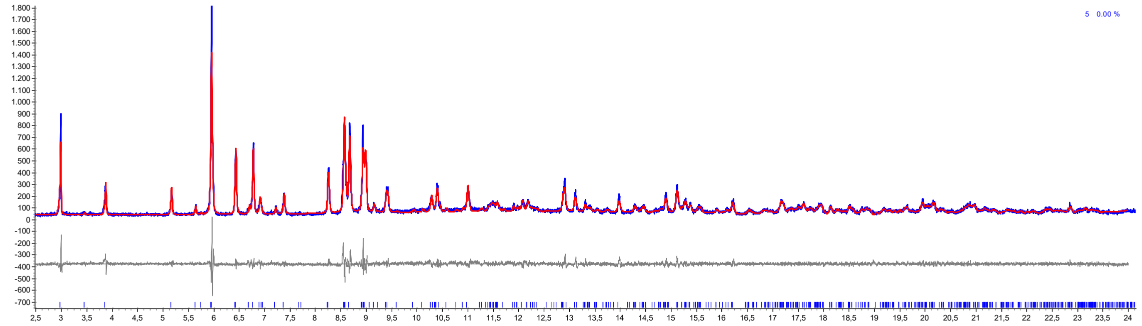

Supplementary Figure 116. Observed (blue) and calculated (red) profiles and difference plot [I_{obs}-I_{calc}] (grey) of the Pawley refinement of Pattern 9 at 1.05 bar CH_4 (T = 180 K) ($2.5 \leq 2\theta \leq 24.0$ °; d_{min} = 2.03 Å) (R_{wp} = = 12.370, R_{wp}' = 22.237).

5. Gas sorption for coordination polymers 1-5

5.1 N_2 adsorption isotherms for coordination polymers 1, 2, 4 and 5

Volumetric adsorption measurements were undertaken using a Micromeritics ASAP2420 Accelerated Surface Area and Porosimetry System. The samples were degassed under vacuum overnight prior to analysis, performed using nitrogen adsorption at 77 K (99.99 % nitrogen adsorbate). A total of 49 points were taken on the adsorption branch and 30 on the desorption branch. The results shown in Supplementary Figure 117, for the coordination polymers with the two longest and shortest perfluor0alkyl chains (i.e. **1**, **2**, **4** and **5**), demonstrate that the materials are non-porous.

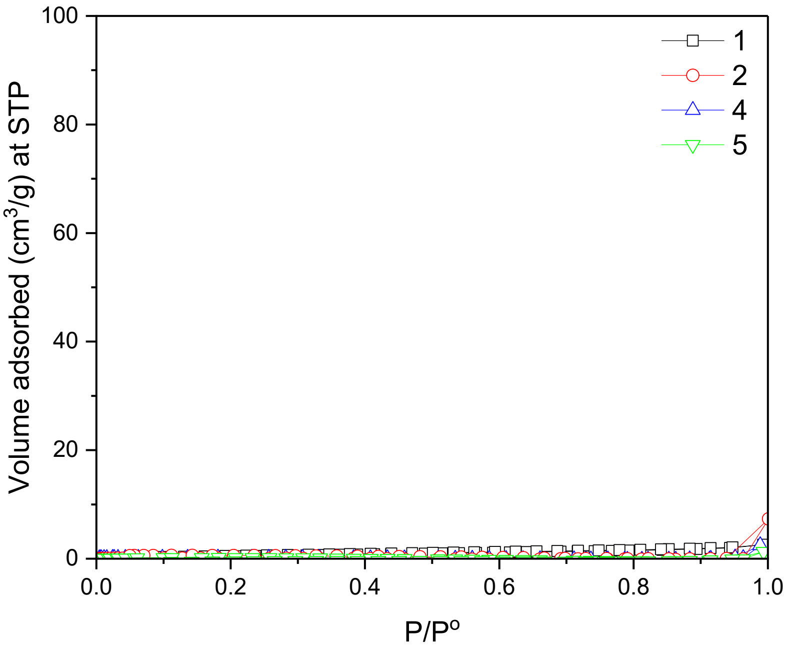

Supplementary Figure 117. N_2 adsorption isotherms for coordination polymers **1**, **2**, **4** and **5** at 77 K. Adsorption and desorption branches overlap.

5.2 CO_2 and CH_4 adsorption isotherms for coordination polymers 1-5

Gravimetric adsorption measurements were recorded using an Intelligent Gravimetric Analyser (IGA) model 003 supplied by Hiden Isochema Ltd. The balance and pressure control system of the instrument are fully thermostatted to 0.1 K and the microbalance has a weighing resolution of 0.2 µg. Samples (**1-5**) were outgassed until they reached a constant mass, at a pressure of < 10^{-6} mbar at 273 K. During measurements the pressure of the gas was gradually increased over ≈ 15 s avoiding disruption to the microbalance. Pressure control used a 0 - 2 MPa pressure transducer, with an accuracy of 0.02 MPa. The pressure was maintained at the set point by active computer control. The mass uptake was measured as a function of time and the approach to equilibrium monitored in real time with a computer algorithm. After equilibrium was established, the pressure of gas in the system was increased to the next set pressure value and the subsequent uptake was measured until equilibrium was re-established. Pressure steps in the range 0 − 20 bar were used to obtain CO_2 isotherms for all compounds at 273 K. Additional CO_2 isotherms were measured for samples **1-3** and **5** at 253 K and for compound **4** at 258 and 263 K. Pressure steps in the range 0 − 10 bar were used to obtain CH_4 isotherms for compounds **1** and **2** at 273 K. Further mixed gas isotherms (90/10 or 80/20 CO_2/CH_4) were measured (0 − 10 bar) for compounds **1** and **2** at 273 K in order to investigate selectivity between the two gases. During carbon dioxide, methane and carbon dioxide/methane mixture adsorption the sample temperature was maintained using a thermostirrer, and was constantly monitored throughout the duration of the experiment. All measurements were made using task-specific software, supplied by Hiden Isochema Ltd. CO_2 isotherms are shown in Supplementary Figures 118-133. CH_4 isotherms are shown in Supplementary Figures 134 and 135. Mixed CO_2/CH_4 isotherms are shown in Figure 4.

5.2.1 CO_2 adsorption isotherms for coordination polymer 1

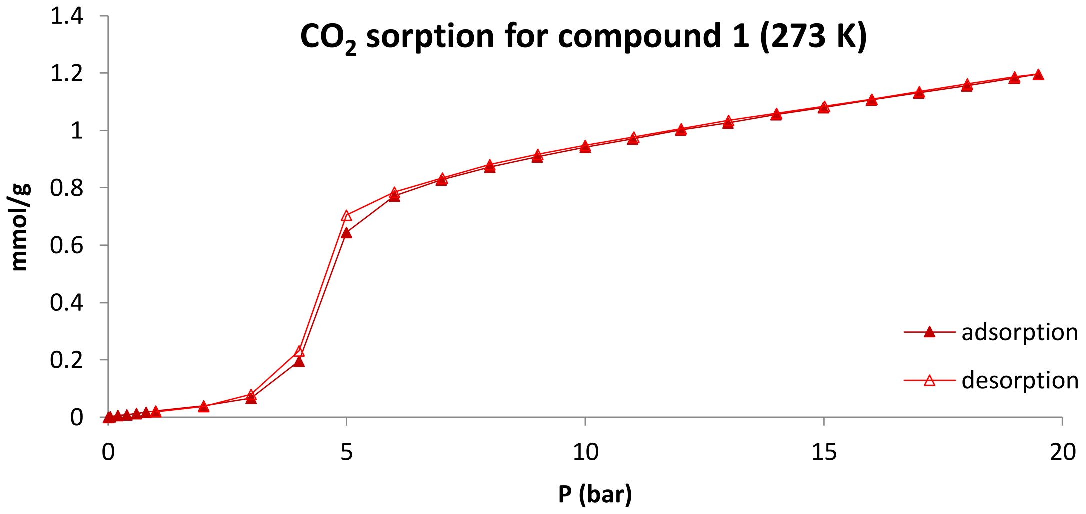

Supplementary Figure 118. CO_2 adsorption isotherms for coordination polymer **1** at 273 K. Adsorption data dark red filled triangles and desorption data in light red open triangles.

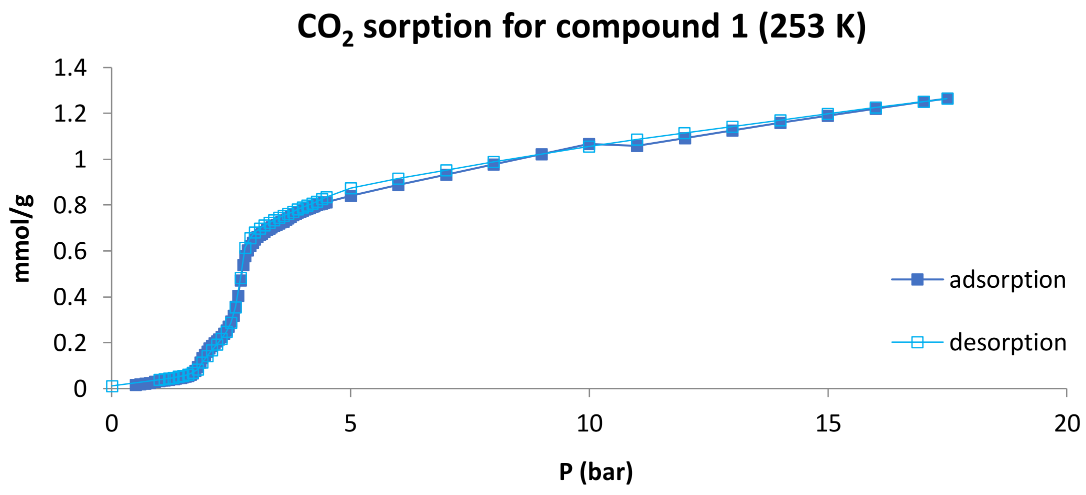

Supplementary Figure 119. CO_2 adsorption isotherms for coordination polymer **1** collected at 253 K. Adsorption data dark blue filled triangles and desorption data in light blue open squares.

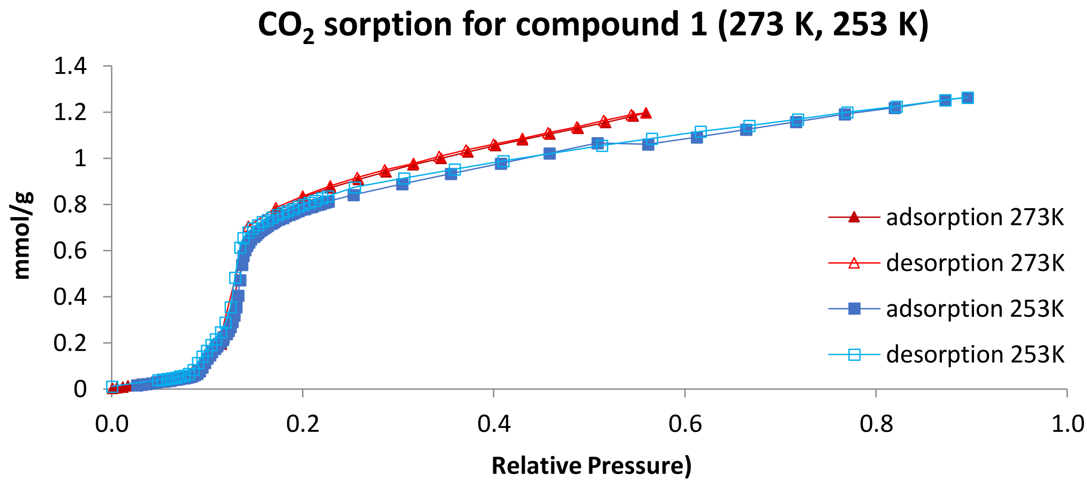

Supplementary Figure 120. CO_2 adsorption isotherms as a function of relative pressure (p/p_0) for coordination polymer **1**. At 273 K: adsorption data dark red filled triangles and desorption data pale red open triangles. At 253 K: adsorption data dark blue filled squares and desorption data light blue open squares.

5.2.2 CO_2 adsorption isotherms for coordination polymer 2

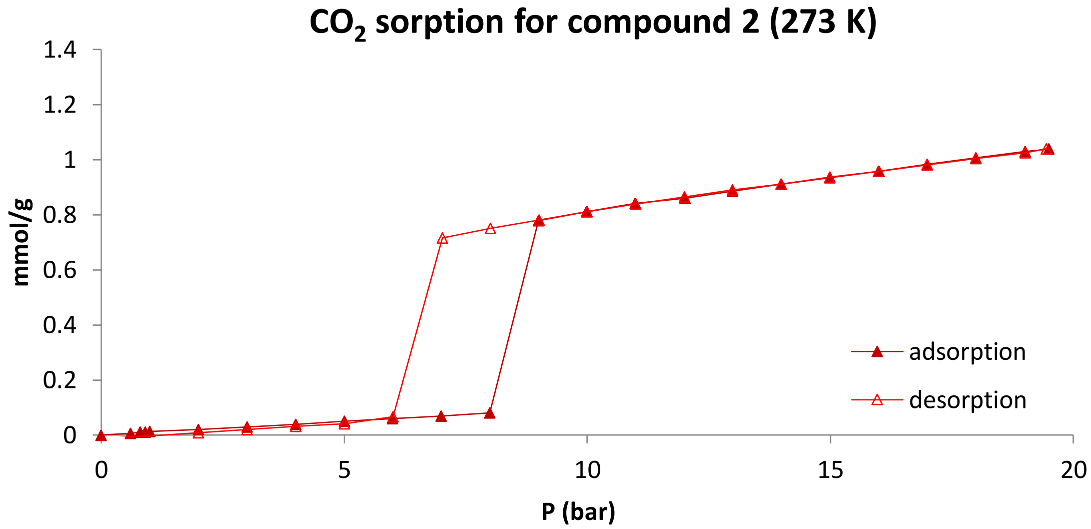

Supplementary Figure 121. CO_2 adsorption isotherms for coordination polymer **2** at 273 K. Adsorption data dark red filled triangles and desorption data in light red open triangles.

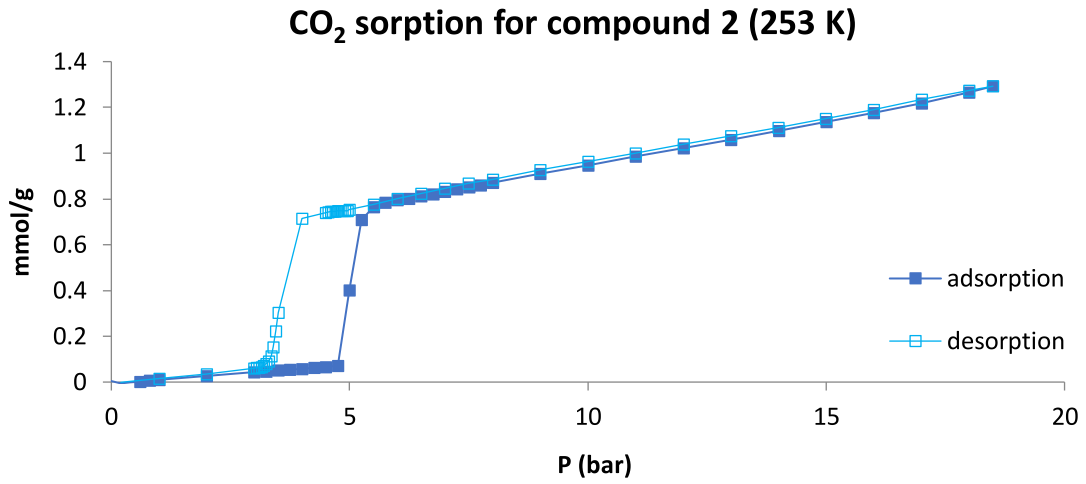

Supplementary Figure 122. CO_2 adsorption isotherms for coordination polymer **2** collected at 253 K. Adsorption data dark blue filled triangles and desorption data in light blue open triangles.

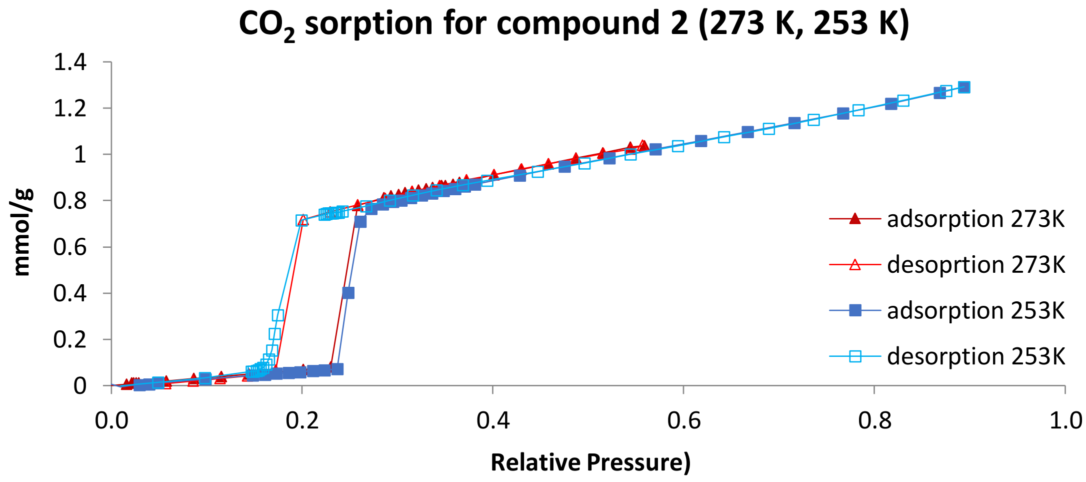

Supplementary Figure 123. CO_2 adsorption isotherms as a function of relative pressure (p/p_0) for coordination polymer **2**. At 273 K: adsorption data dark red filled triangles and desorption data light red open triangles. At 253 K: adsorption data dark blue filled squares and desorption data light blue open squares.

5.2.3 CO_2 adsorption isotherms for coordination polymer 3

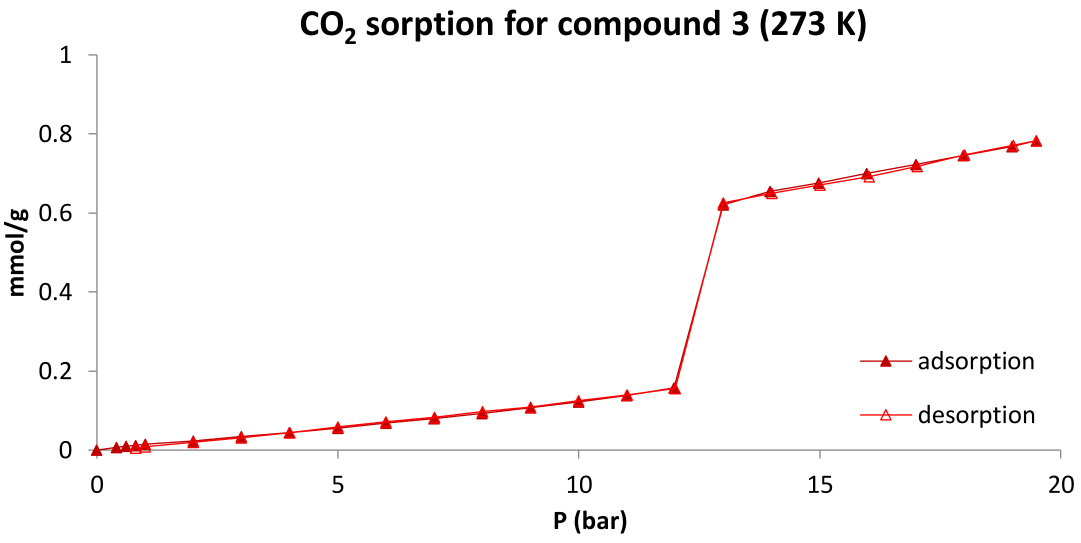

Supplementary Figure 124. CO_2 adsorption isotherms for coordination polymer **3** at 273 K. Adsorption data dark red filled triangles and desorption data in light red open triangles.

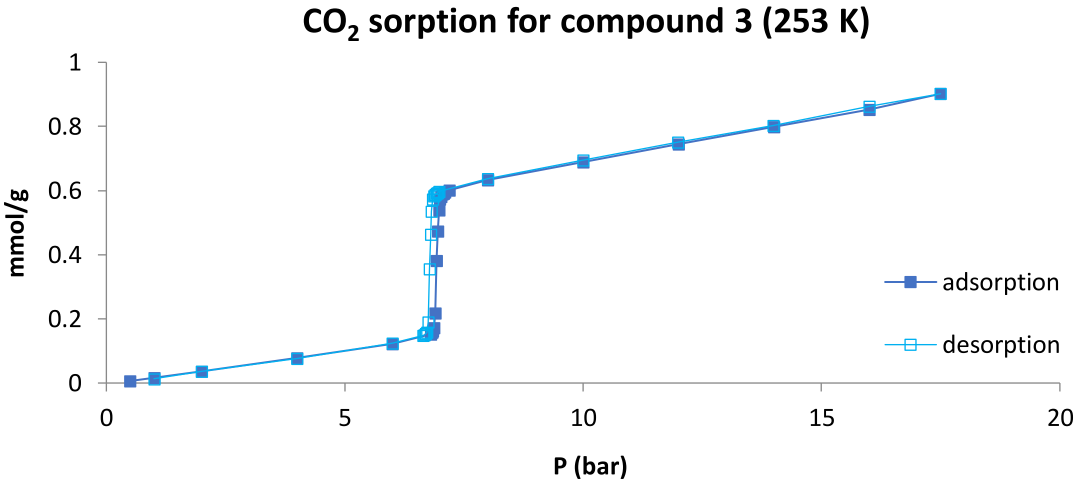

Supplementary Figure 125. CO_2 adsorption isotherms for coordination polymer **3** collected at 253 K. Adsorption data dark blue filled triangles and desorption data in light blue open triangles.

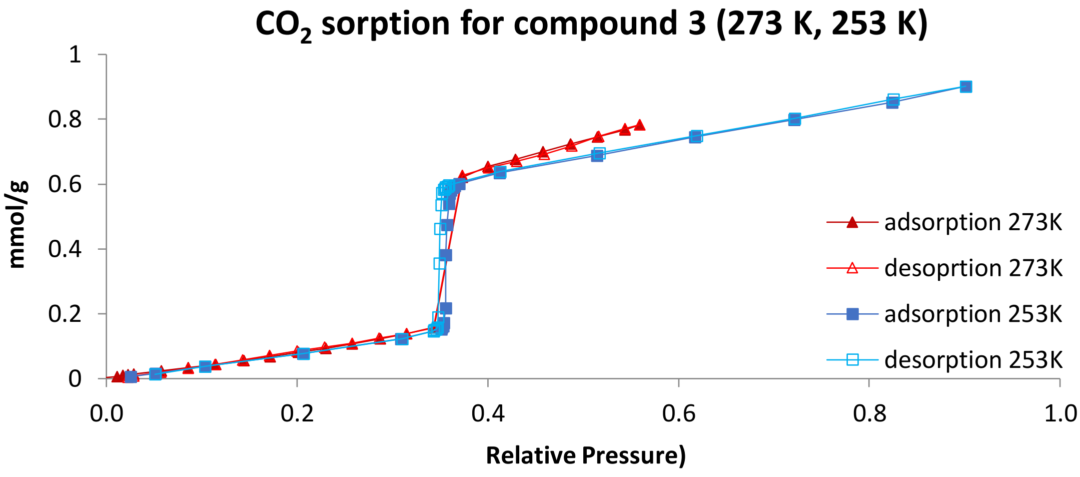

Supplementary Figure 126. CO_2 adsorption isotherms as a function of relative pressure (p/p_0) for coordination polymer **3**. At 273 K: adsorption data dark red filled triangles and desorption data light red open triangles. At 253 K: adsorption data dark blue filled squares and desorption data light blue open squares.

5.2.4 CO_2 adsorption isotherms for coordination polymer 4

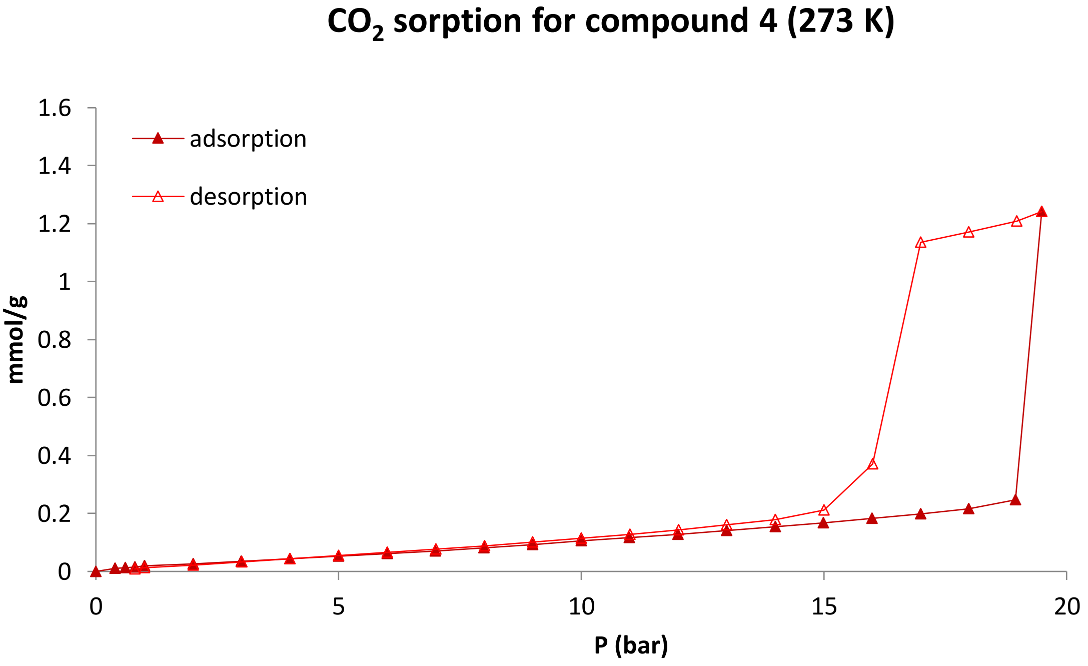

Supplementary Figure 127. CO_2 adsorption isotherms for coordination polymer **4** collected at 273 K. Adsorption data dark red filled triangles and desorption data in light red open triangles.

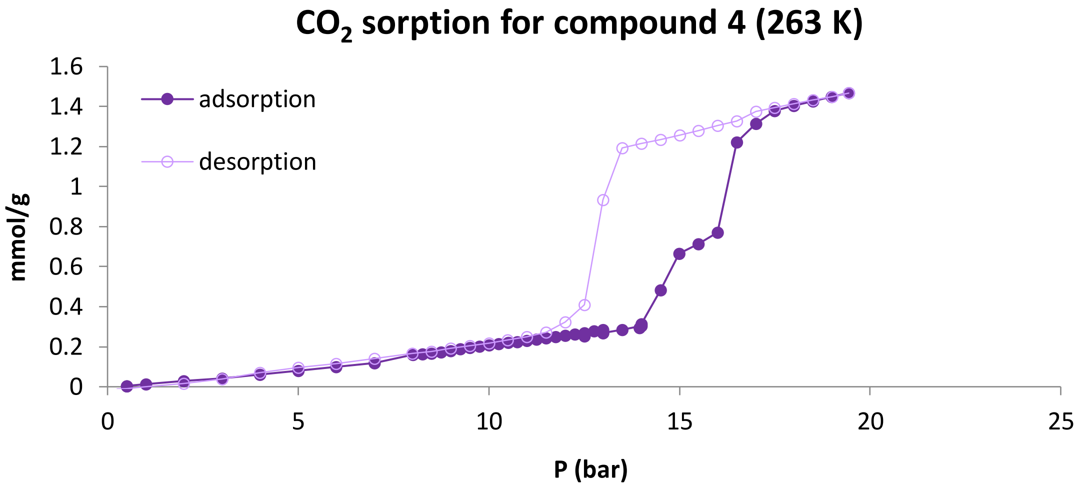

Supplementary Figure 128. CO_2 adsorption isotherms for coordination polymer **4** collected at 263 K. Adsorption data deep purple filled circles and desorption data in light purple open circles.

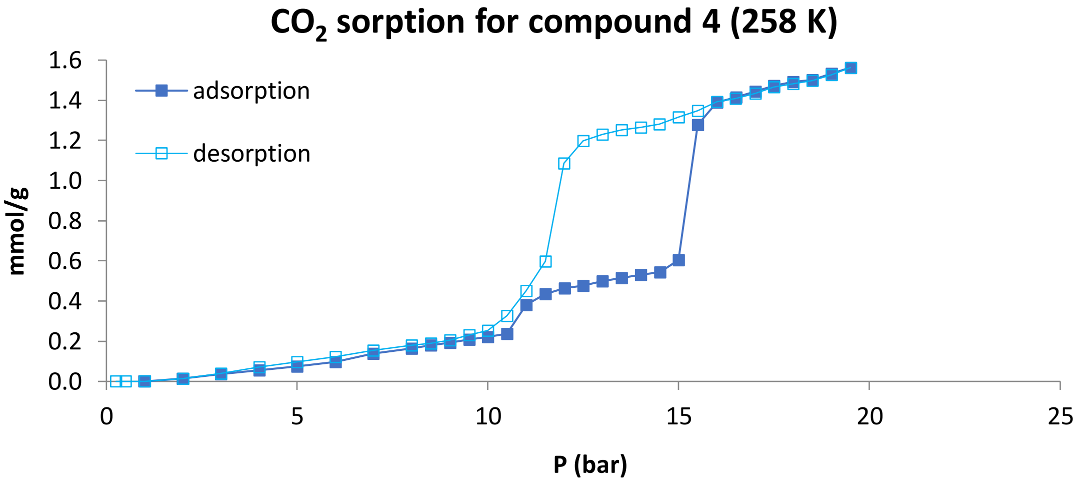

Supplementary Figure 129. CO_2 adsorption isotherms for coordination polymer **4** collected at 258 K. Adsorption data dark blue filled squares and desorption data in light blue open squares.

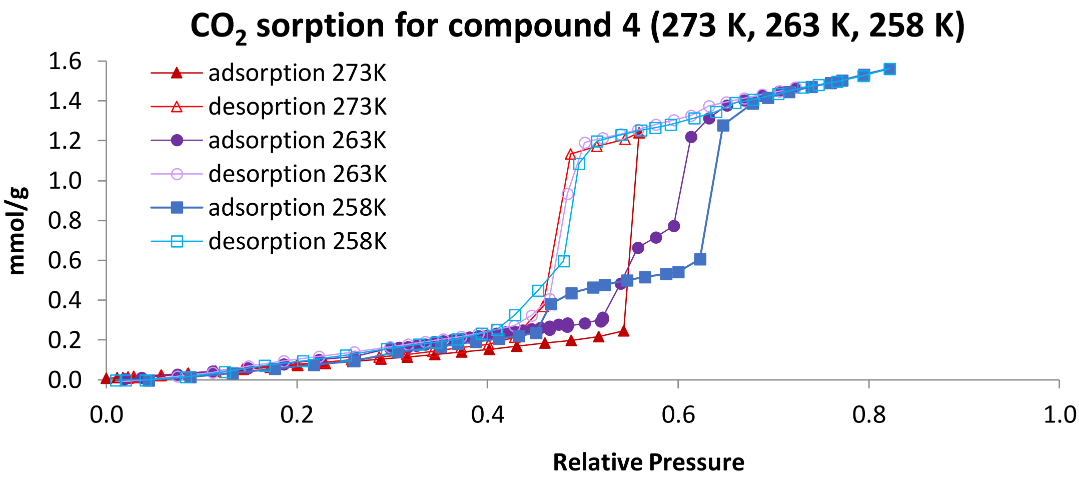

Supplementary Figure 130. CO_2 adsorption isotherms as a function of relative pressure (p/p_0) for coordination polymer **4**. At 273 K: adsorption data dark red filled triangles and desorption data light red open triangles. At 263 K: adsorption data deep purple filled circles and desorption data light purple open circles. At 258 K: adsorption data dark blue filled squares and desorption data light blue open squares.

5.2.5 CO_2 adsorption isotherms for coordination polymer 5

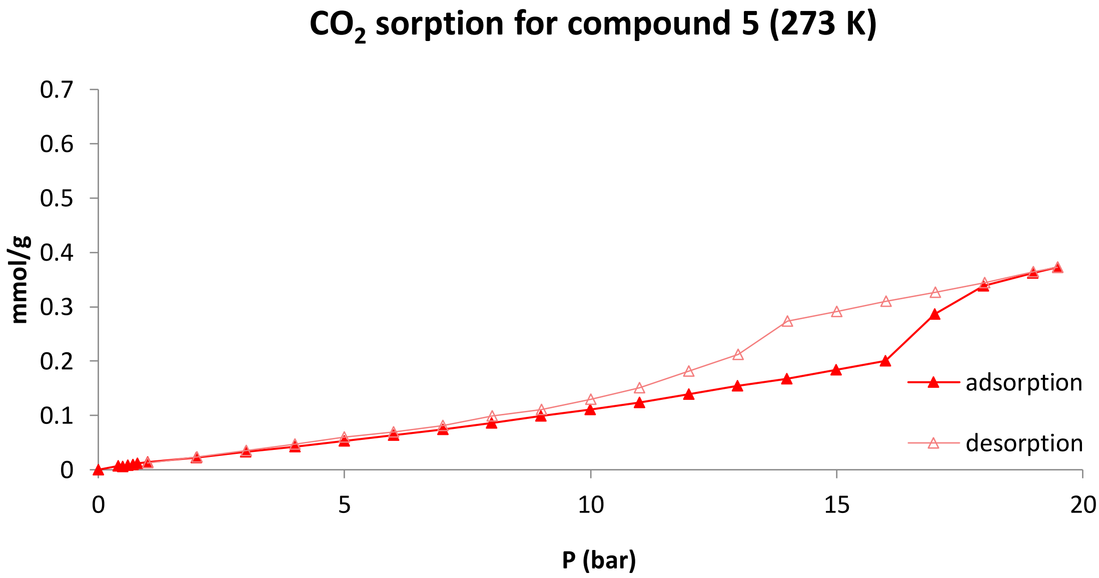

Supplementary Figure 131. CO_2 adsorption isotherms for coordination polymer **5** at 273 K. Adsorption data dark red filled triangles and desorption data in light red open triangles.

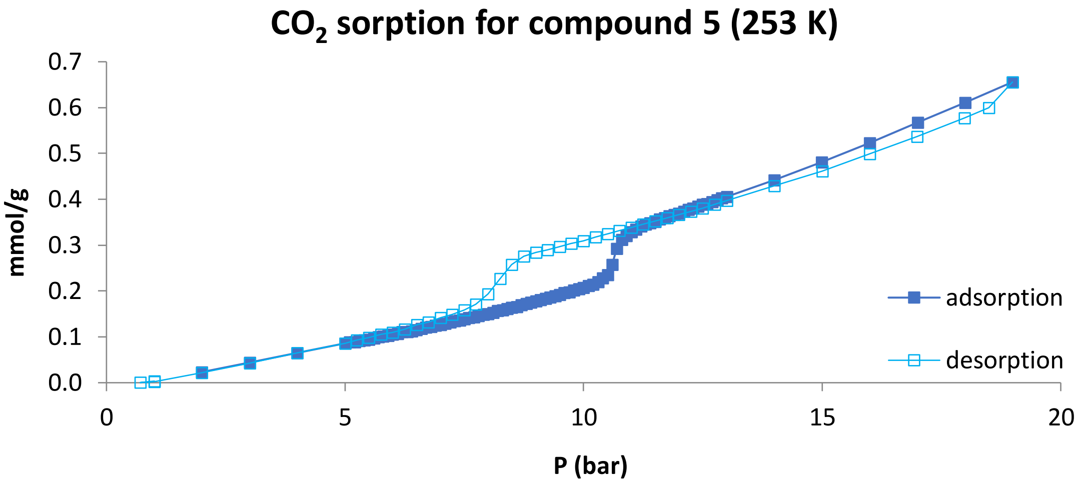

Supplementary Figure 132. CO_2 adsorption isotherms for coordination polymer **5** collected at 253 K. Adsorption data dark blue filled squares and desorption data in light blue open squares.

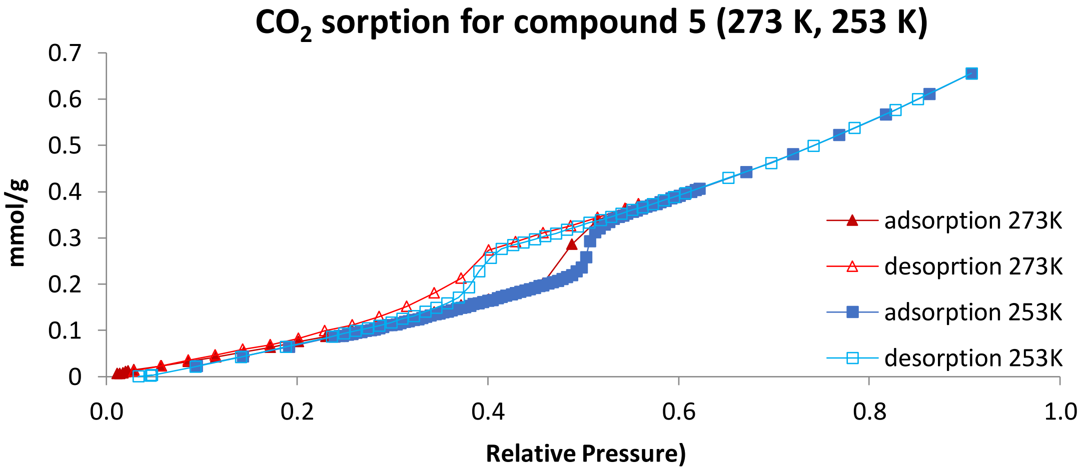

Supplementary Figure 133. CO_2 adsorption isotherms as a function of relative pressure (p/p_0) for coordination polymer **5**. At 273 K: adsorption data dark red filled triangles and desorption data light red open triangles. At 253 K: adsorption data dark blue filled squares and desorption data light blue open squares.

5.2.6 CH_4 adsorption isotherm for coordination polymer 1

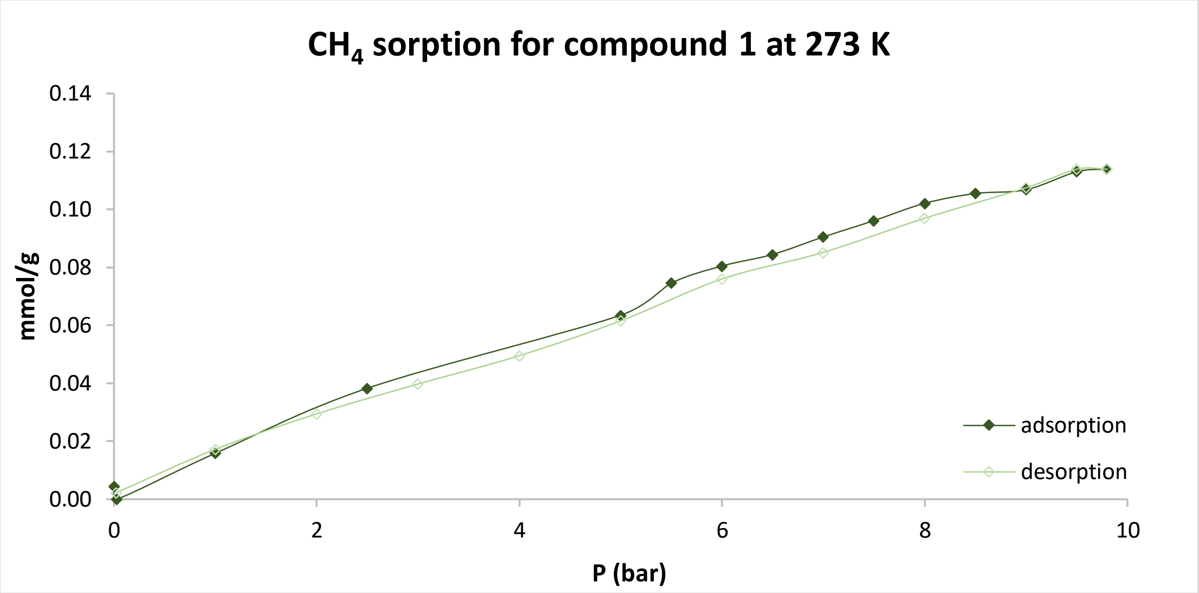

Supplementary Figure 134. CH_4 adsorption isotherm for coordination polymer **1** at 273 K. Adsorption data dark green filled diamonds and desorption data in light green open diamonds.

5.2.7 CH_4 adsorption isotherm for coordination polymer 2

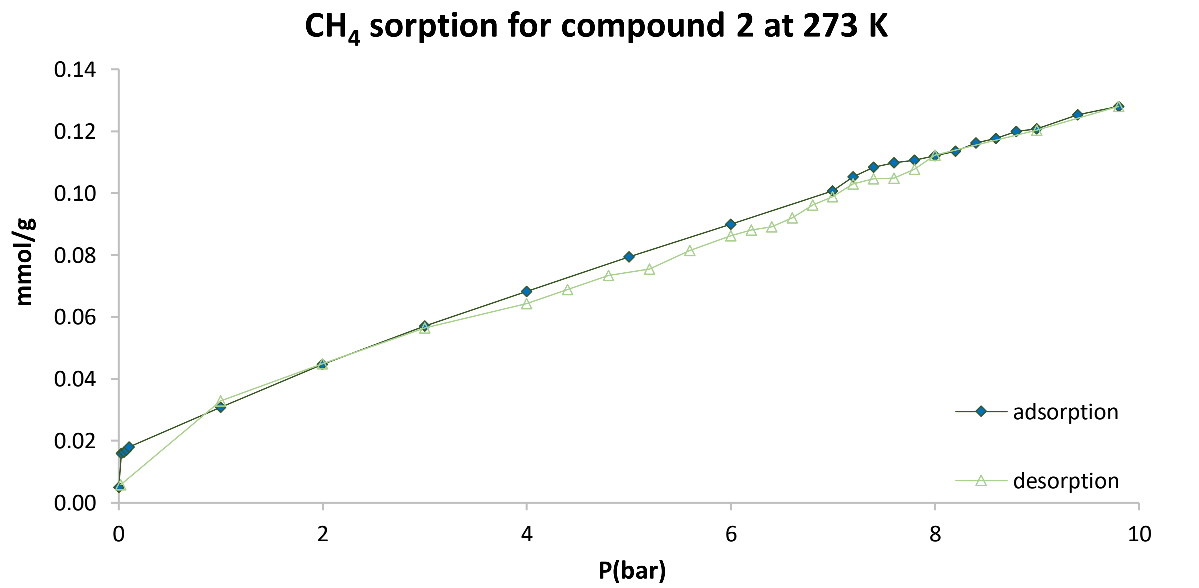

Supplementary Figure 135. CH_4 adsorption isotherm for coordination polymer **2** at 273 K. Adsorption data dark green filled diamonds and desorption data in light green open diamonds.

6. CheckCIF A and B alerts

32 CIFs for the single-crystal structure determinations are provided as part of the Supplementary Information. A full list of alerts and responses are included in the CIFs.

Typical Level A alerts include

"Structure Contains Solvent Accessible VOIDS"

This is a common alert for porous materials such as MOFs when not all of the void contents, which is often disordered solvent molecules, is able to be modelled.

"_diffrn_measured_fraction_theta_full value Low"

Strongest reflections were treated as detector overloads and were removed from the data. As a consequence only 91% of the data remained.

OR

Geometric restrictions on the goniometer-gas cell set up only allowed to collect 95% of the data to 0.83 Å of resolution.

"The value of sine(theta_max)/wavelength is less than 0.550. Calculated sin(theta_max)/wavelength = 0.4999"

No diffraction was found beyond 1Å of resolution. So the data was trimmed accordingly.

"Isotropic non-H Atoms in Main Residue(s) - F1 F2 F3 F4 F5 F6 etc."

(Fluorine) atoms were refined isotropically in order to maximize the data/parameter ratio

Typical Level B alerts include

"Missing FCF Refl Between Thmin & STh/L= 0.600"

Strongest reflections were treated as detector overloads and were removed from the data. As consequence only 91% of the data remained.

"Low Bond Precision on C-C Bonds".

Disorder in the perfluoroalkyl chains lowered the precision in the C-C bonds

"Large Average Ueq of Residue Including O3"

Oxygen corresponding to the CO2 has a large Ueq because the CO2 is located in an open space and has more freedom to move.

7. Displacement ellipsoid plots for single-crystal structures

Crystal structures of **1-MeOH** (CCDC 903747), **1** (polymorph $\mathbf{1_A}^{\mathbf{HT}}$ (CCDC 903751)), $\mathbf{1_A}^{\mathbf{LT}}$ (CCDC 1044595), $\mathbf{1_B}^{\mathbf{HT}}$ (CCDC 1044597) and $\mathbf{1_B}^{\mathbf{LT}}$ (CCDC 1044596), **2-EtOH** (CCDC 654182) and **2** (polymorph $\mathbf{2}^{\mathbf{LT}}$ (CCDC 654183) have been previously reported. CCDC deposition numbers of displacement ellipsoid plots showing the asymmetric unit are presented in Supplementary Table 19 for all crystal structures reported for the first time in this study, for which crystal data are provided in Supplementary Tables 1-11. Where disorder is present, all modelled components of the disorder are shown.

Supplementary Table 19. CCDC deposition numbers and displacement ellipsoid plots

Compound	CCDC deposition no.	Displacement ellipsoid plot (50 % probability)
**1_A^{HT}** **(298 K, 2 bar CO_2)**	2329010	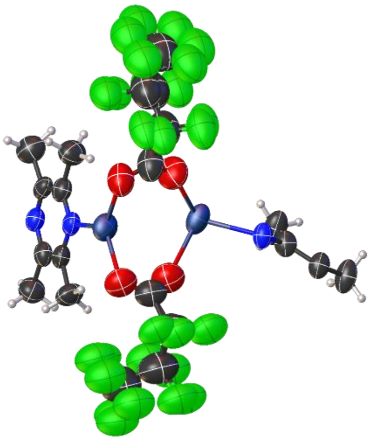
**1^{CO2}** **(253 K, 10 bar CO_2)**	2329012	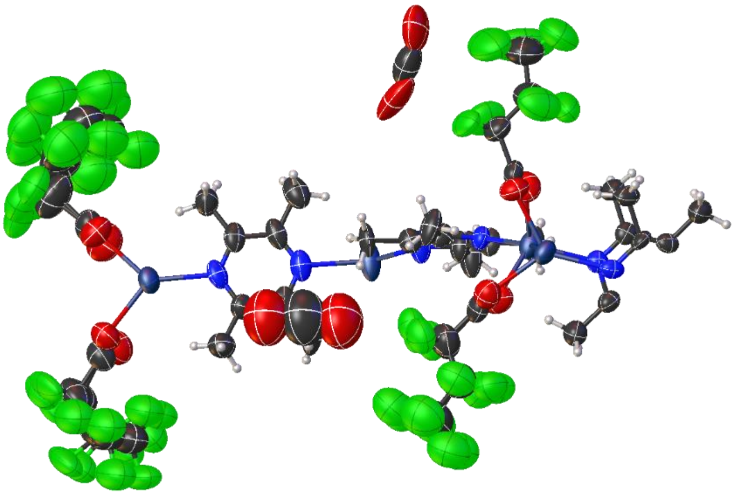
**1^{CO2}** **(232 K, 10 bar CO_2)**	2329013	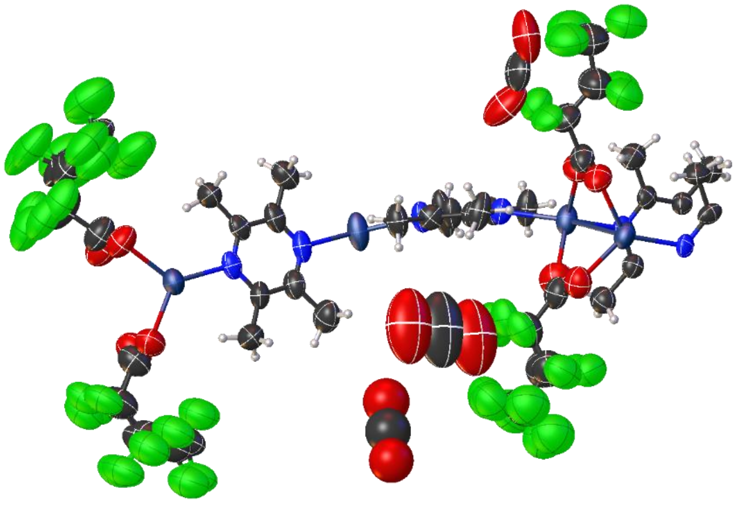

**2^{HT} (298 K, vacuum)**	2329019	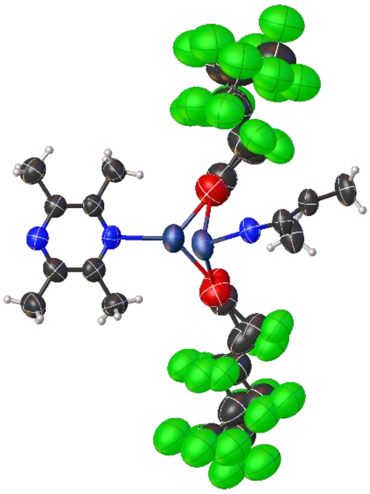
**2^{HT} (273 K, 10 bar CO_2)**	2329018	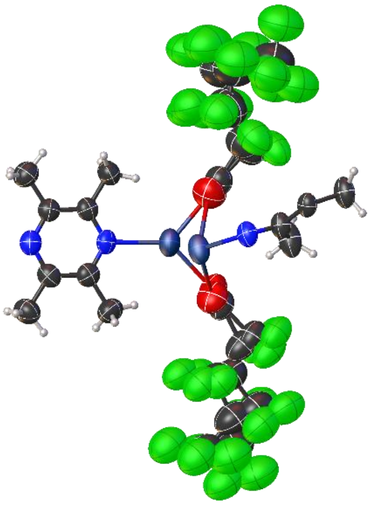
**2^{HT} (240 K, 10 bar CO_2)**	2329017	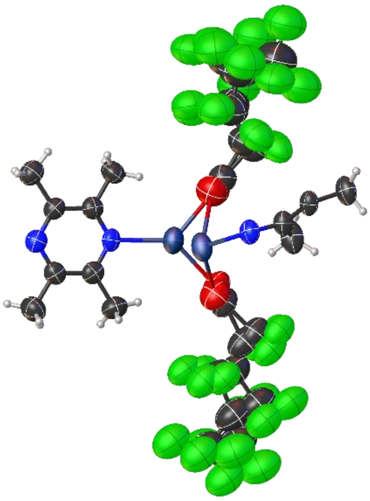

2^{CO2} (215 K, 10 bar CO_2)	2329015	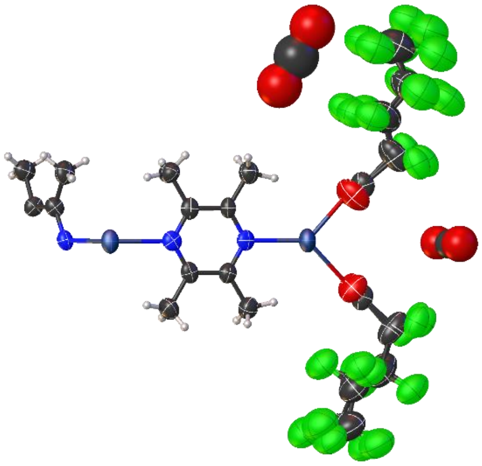
2^{CO2} (200 K, 10 bar CO_2)	2329016	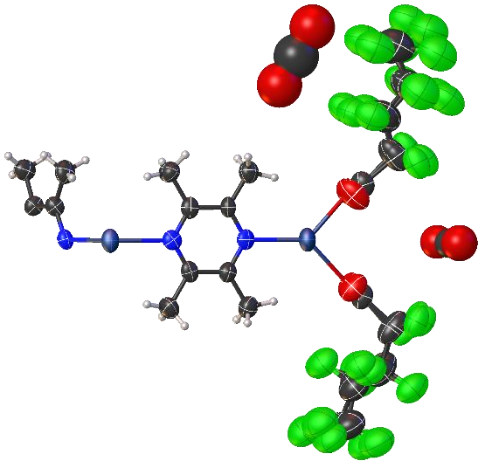
2^{HT} (298 K, vacuum)_2	2329020	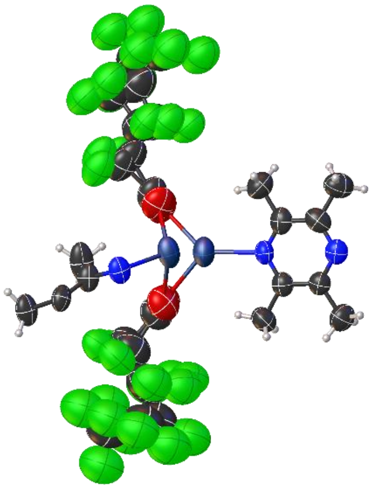

**3-EtOH**<br><br>**100 K**	2329007	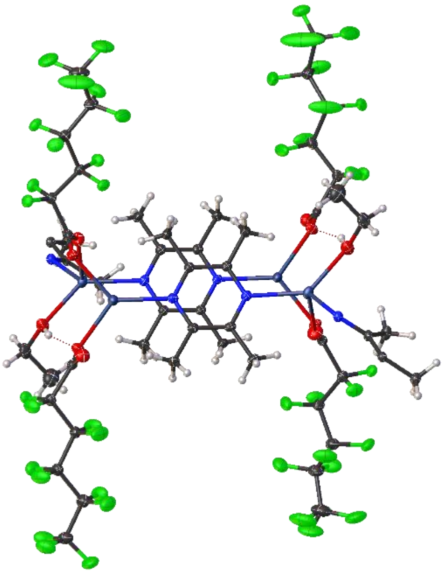
**3^{LT}**<br><br>**100 K**	2329008	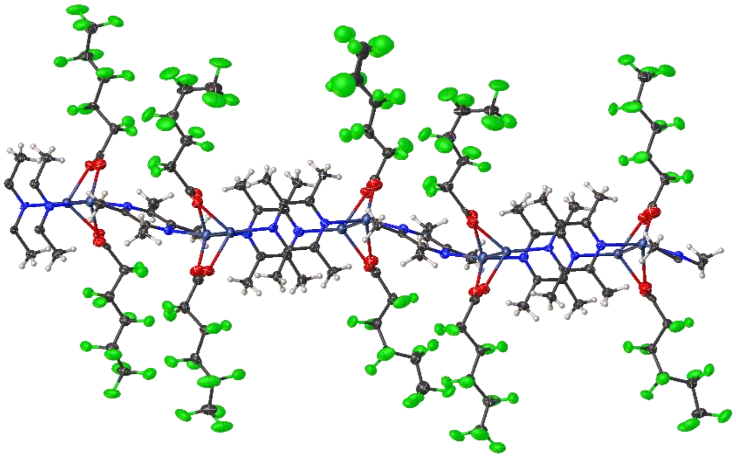
**3^{HT}**<br><br>**(343 K, vacuum)**	2329025	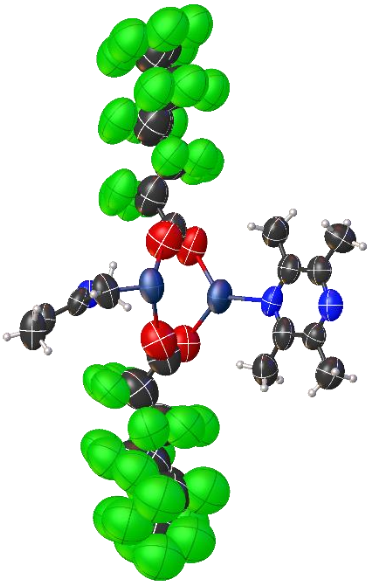

**3**[HT] **(298 K, 10 bar CO_2)**	2329022	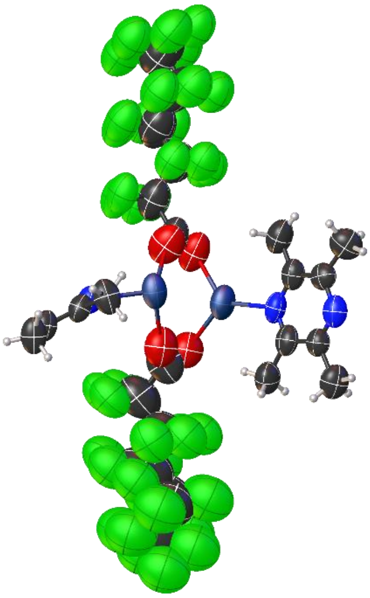
**3**[HT] **(250 K, 10 bar CO_2)**	2329024	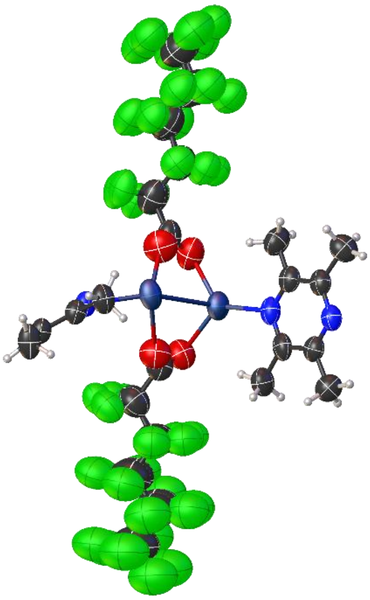

**3_A^{CO2}** (230 K, 10 bar CO_2)	2329023	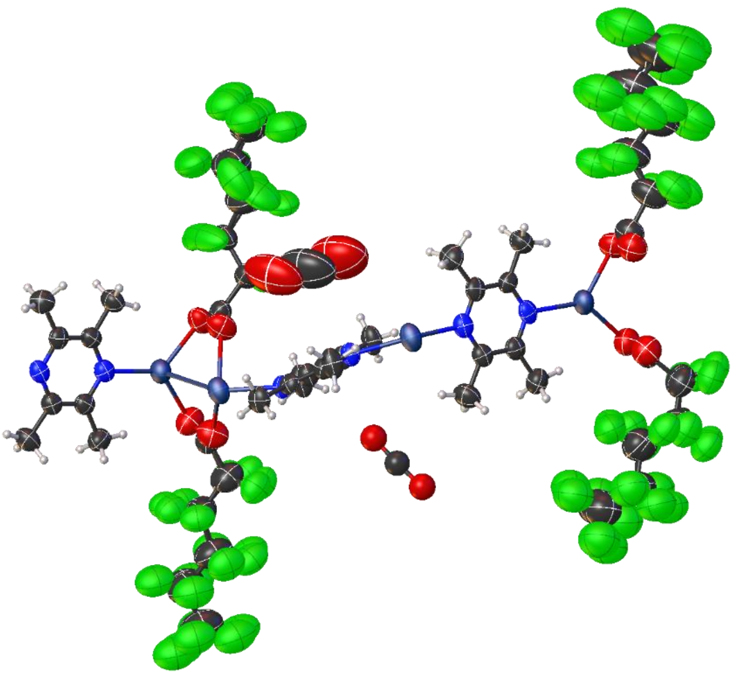
**3_A^{CO2}** (215 K, 10 bar CO_2)	2329026	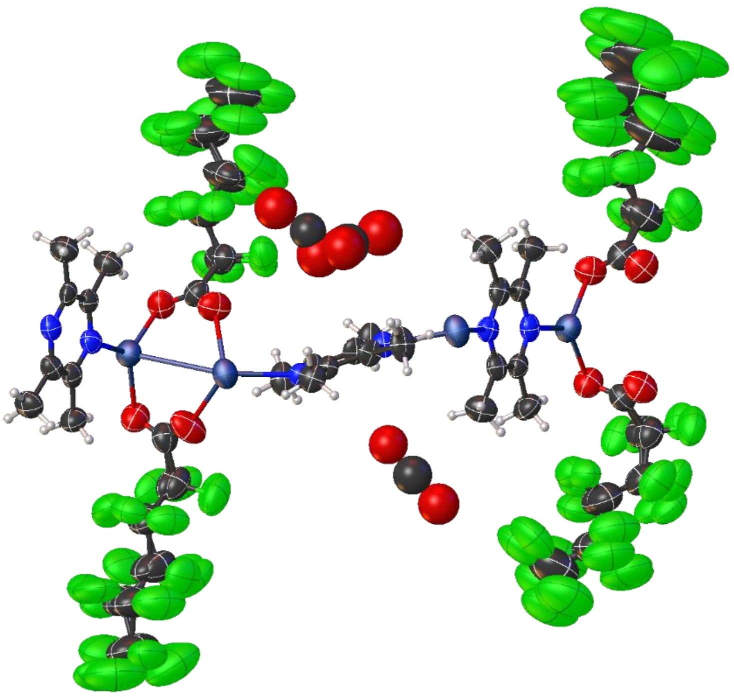

<table>
<tr><td>3_B^{CO2}

(200 K, 10 bar CO₂)</td><td>2329027</td><td>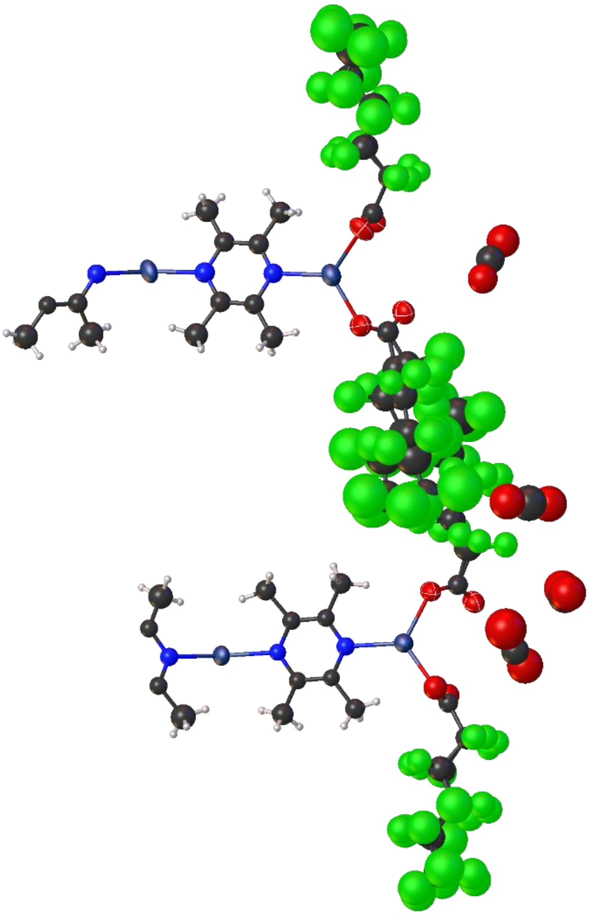</td></tr>
<tr><td>3^{HT}

(298 K, 1 bar CO₂)</td><td>2329021</td><td>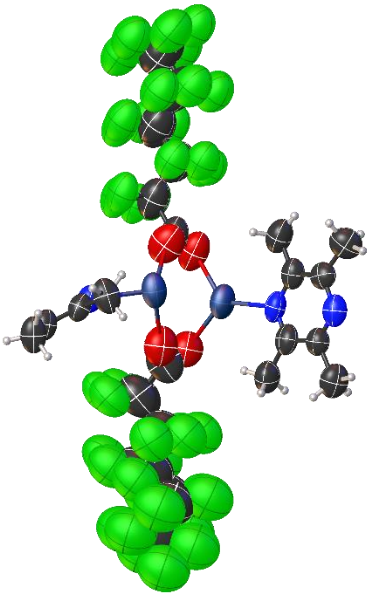</td></tr>
</table>

**4-MeOH**<br><br>**100 K**	2329006	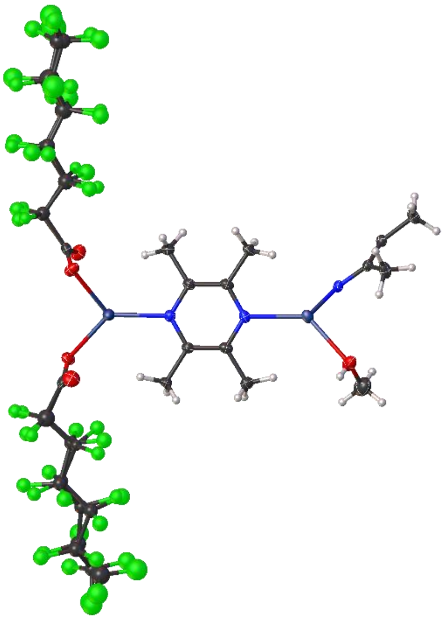
**4**<br><br>**(273 K, vacuum)**	2329028	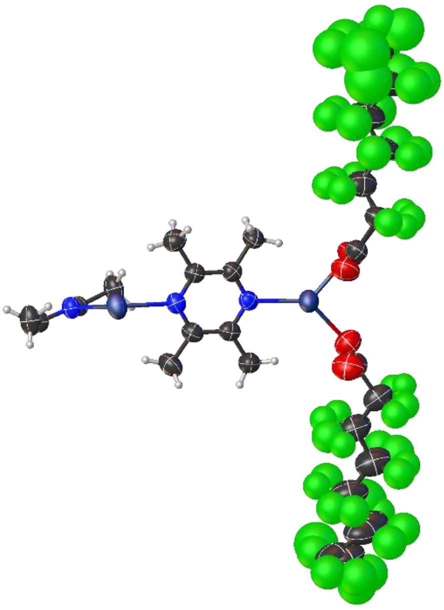

**4**<br><br>**(273 K, 10 bar CO_2)**	2329032	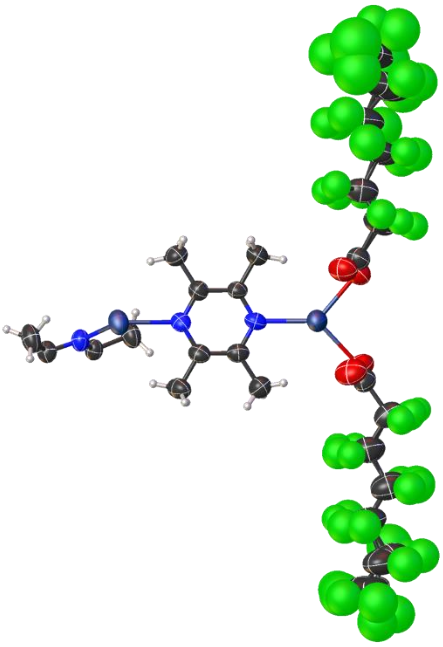
**4^{CO2}**<br><br>**(240 K, 10 bar CO_2)**	2329034	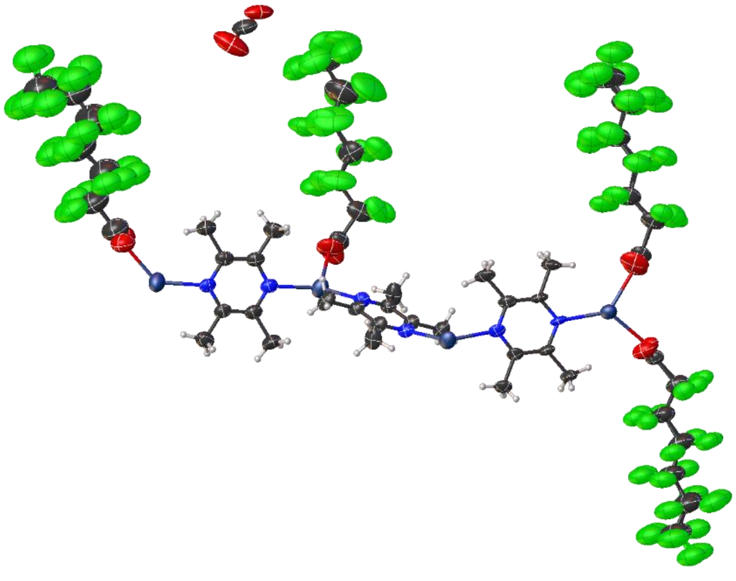

**4^{CO2}** **(230 K, 10 bar CO_2)**	2329029	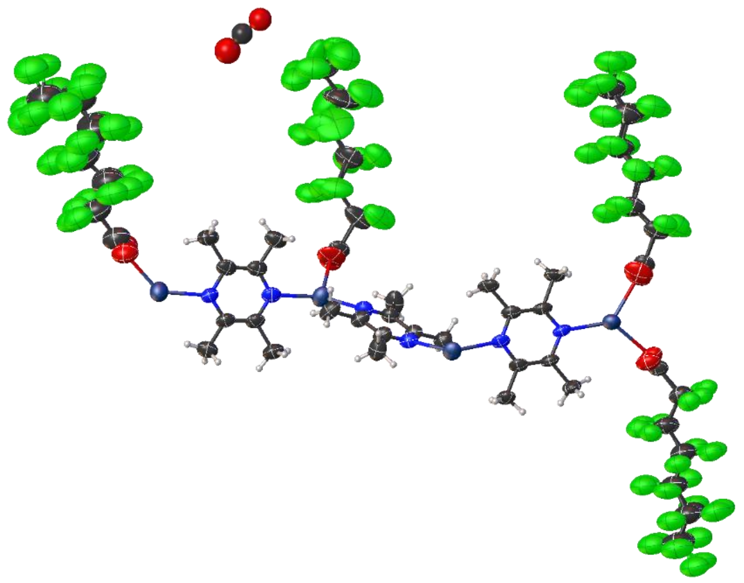
**4^{CO2}** **(215 K, 10 bar CO_2)**	2329033	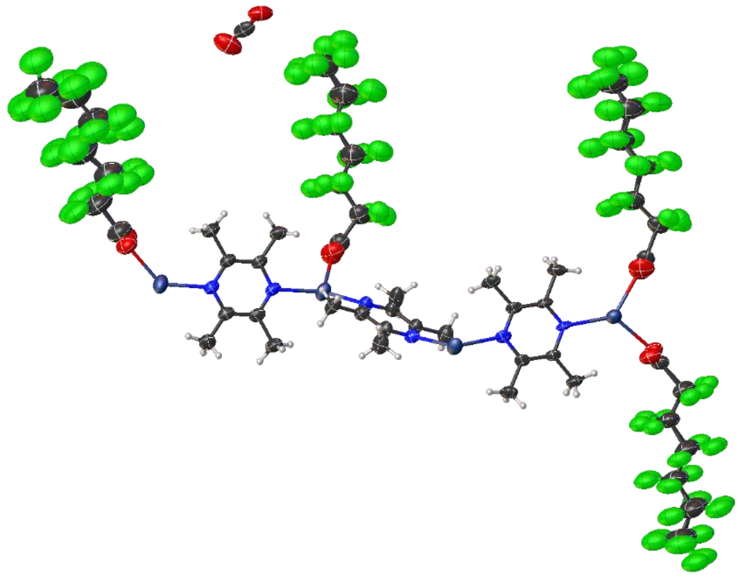

**4**<br><br>**(273 K, vacuum)_2**	2329031	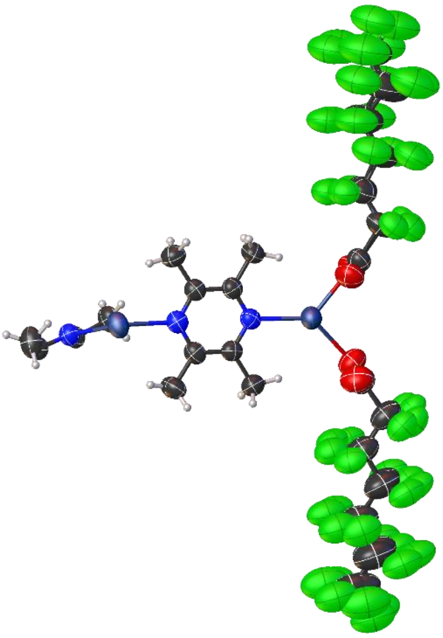
**4^{CO2}**<br><br>**(200 K, 10 bar CO_2)**	2329030	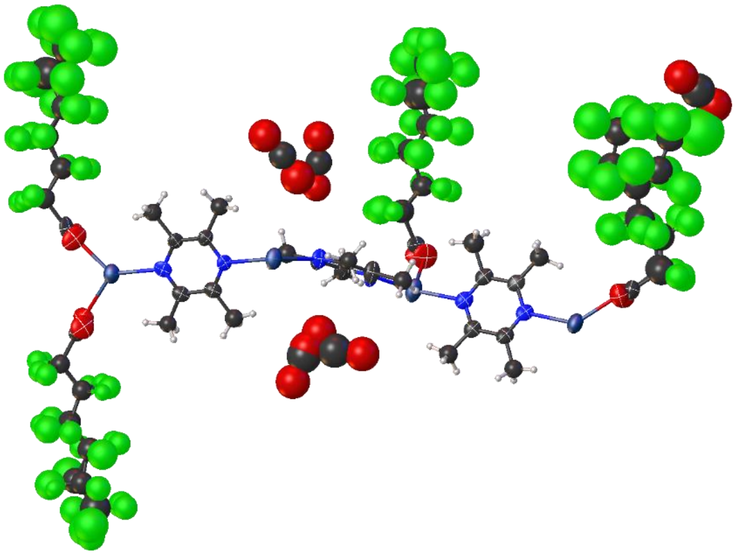

**5-MeOH**<br><br>**100 K**	2329009	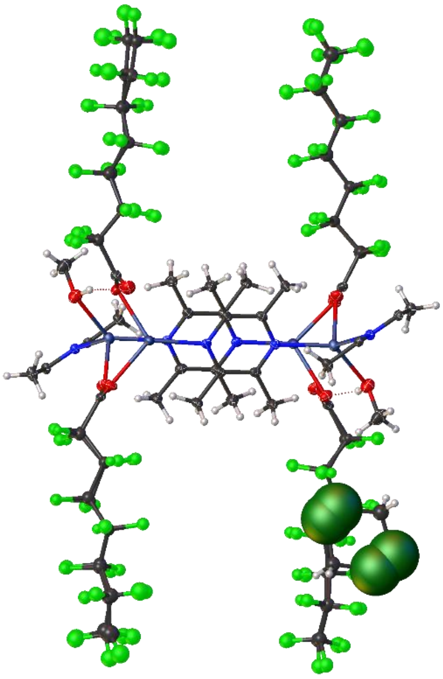
**5**<br><br>**(295 K, vacuum)**	2329038	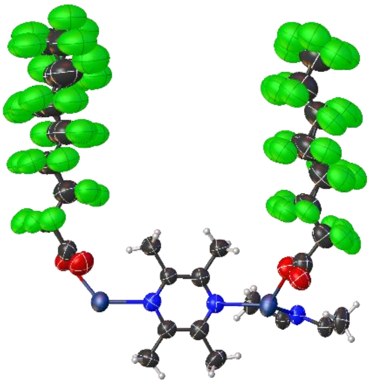
**5**<br><br>**(295 K, 10 bar CO_2)**	2329037	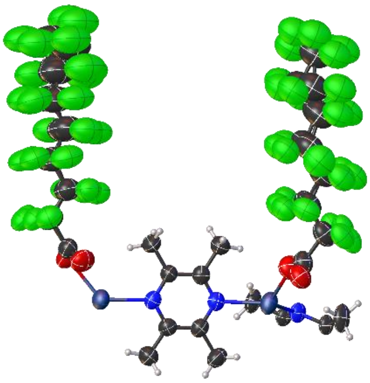

**5** (250 K, 10 bar CO_2)	2329036	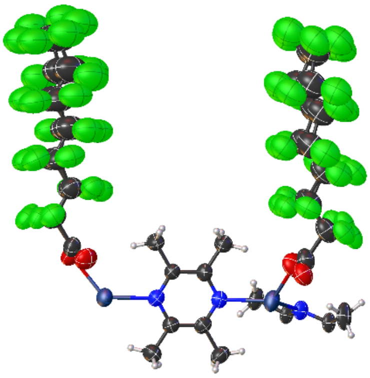
**5** (230 K, 10 bar CO_2)	2329035	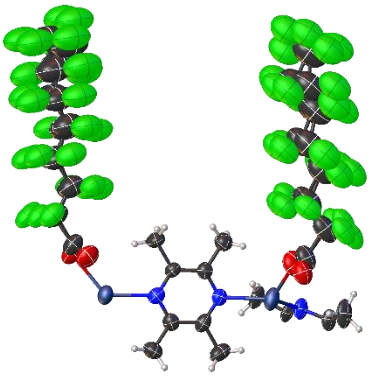
**5^{CO2}** (200 K, 10 bar CO_2)	2329039	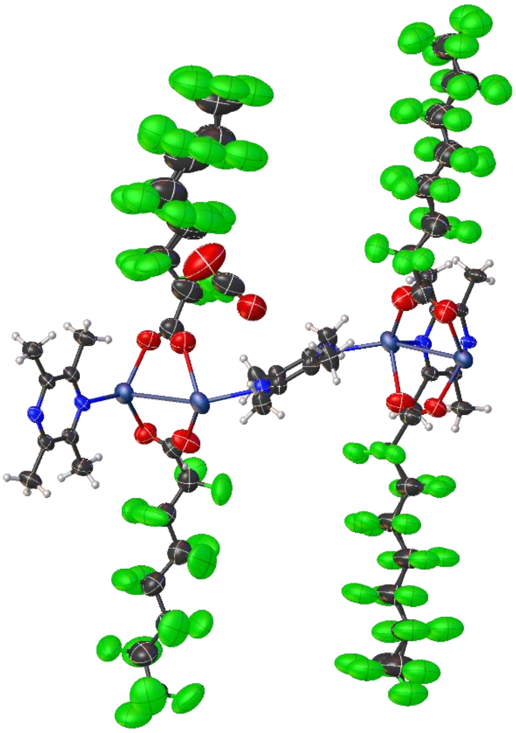

8. References

S1. Hill, A. H. A new gas system for automated in situ powder diffraction studies at the European Synchrotron Radiation Facility. *J. Appl. Cryst.* **46**, 570–572 (2013).

S2. Nowell, H., Barnett, S. A., Christensen, K. E., Teat, S. J. & Allan, D. R. I19, the small-molecule single-crystal diffraction beamline at Diamond Light Source. *J Synchrotron Radiat.* **19**, 435–441 (2012).

S3. Vitórica-Yrezábal, I. J., Libri, S., Loader, J. R., Mínguez Espallargas, G., Hippler, M., Fletcher, A. J., Thompson, S. P., Warren, J. E., Musumeci, D., Ward, M. D. & Brammer, L. Coordination Polymer Flexibility Leads to Polymorphism and Enables a Crystalline Solid–Vapour Reaction: A Multi-technique Mechanistic Study. *Chem. Eur. J.* **21**, 8799–8811 (2015).

S4. Libri, S., Mahler, M., Mínguez Espallargas, G., Singh, D. C. N. G., Soleimannejad, J., Adams, H., Burgard, M. D., Rath, N. P., Brunelli, M. & Brammer, L. Ligand Substitution within Nonporous Crystals of a Coordination Polymer: Elimination from and Insertion into Ag-O Bonds by Alcohol Molecules in a Solid–Vapor Reaction. *Angew. Chem. Int. Ed.* **47**, 1693–1697 (2008).

S5. (a) Sheldrick, G. M. SADABS, empirical absorption correction program[S5b] based upon the method of Blessing;[S5c] (b) Krause, L., Herbst-Irmer, R., Sheldrick, G. M. & Stalke, D. Comparison of silver and molybdenum microfocus X-ray sources for single-crystal structure determination. *J. Appl. Cryst.* **48**, 3–10 (2015); (c) Blessing, R. H. An empirical correction for absorption anisotropy. *Acta Crystallogr.* **A51**, 33–38 (1995).

S6. Dolomanov, O. V., Bourhis, L. J., Gildea, R. J., Howard, J. A. K. & Puschmann, H. OLEX2: a complete structure solution, refinement and analysis program. *J. Appl. Cryst.* **42**, 339–341 (2009).

S7. Sheldrick, G. M. Crystal structure refinement with SHELXL. *Acta. Crystallogr.* **A71**, 3–8 (2015).

S8. Fitch, A. N. The high resolution powder diffraction beam line at ESRF. *J. Res. Natl. Inst. Stand. Technol.* **109**, 133–142 (2004).

S9. Thompson, S. P., Parker, J. E., Marchal, J., Potter, J., Birt, A., Yuan, F., Fearn, R. D., Lennie, A. R., Street S. R. & Tang, C. C., Fast X-ray powder diffraction on I11 at Diamond. *J. Synchrotron Rad.* **18**, 637–648 (2011).

S10. (a) Coelho, A. A., *TOPAS-Academic*, ver. 4.1, 2007; see http://www.topas-academic.net; (b) Coelho, A. A. *TOPAS* and *TOPAS-Academic*: an optimization program integrating computer algebra and crystallographic objects written in C++. *J. Appl. Crystallogr.* **51,** 210–218 (2018); (c) Coelho, A. A., Evans, J., Evans, I., Kern, A., Parsons, S. The TOPAS symbolic computation system. *Powder Diffr.* **26**, S22–S25 (2011).

S11. Pawley, G. S. Unit-cell refinement from powder diffraction scans. *J. Appl. Cryst.* **14**, 357–361 (1981).

S12. Rietveld, H. M. A profile refinement method for nuclear and magnetic structures. *J. Appl. Cryst.* **2**, 65–71 (1969).

S13. Thompson, S. P., Parker, J. E., Potter, J. Hill, T. P., Birt, A. Cobb, T. M., Yuan, F. & Tang, C. C. Beamline I11 at Diamond: A new instrument for high-resolution powder diffraction. *Rev. Sci. Instrum.* **80**, 075107 (2009).
